# Synthesis of 4′-Thionucleoside Analogues Bearing a C2′ Stereogenic All-Carbon Quaternary Center

**DOI:** 10.3390/molecules29071647

**Published:** 2024-04-06

**Authors:** Carla Eymard, Amarender Manchoju, Abir Almazloum, Starr Dostie, Michel Prévost, Mona Nemer, Yvan Guindon

**Affiliations:** 1 Bioorganic Chemistry Laboratory, Institut de Recherches Cliniques de Montréal (IRCM), Montréal, QC H2W 1R7, Canada; carla.eymard@ircm.qc.ca (C.E.); amarender947@gmail.com (A.M.); starr.dostie@ircm.qc.ca (S.D.); 2 Department of Chemistry, Université de Montréal, Montréal, QC H3C 3J7, Canada; 3 Department of Biochemistry, Microbiology and Immunology, University of Ottawa, Ottawa, ON K1N 6N5, Canada; mazloumabir@gmail.com (A.A.); mnemer@uottawa.ca (M.N.)

**Keywords:** thionucleoside analogues, quaternary stereocenter, synthesis, acyclic approach, S_N_2-like cyclization, kinetic resolution

## Abstract

The design of novel 4′-thionucleoside analogues bearing a C2′ stereogenic all-carbon quaternary center is described. The synthesis involves a highly diastereoselective Mukaiyama aldol reaction, and a diastereoselective radical-based vinyl group transfer to generate the all-carbon stereogenic C2′ center, along with different approaches to control the selectivity of the *N*-glycosidic bond. Intramolecular S_N_2-like cyclization of a mixture of acyclic thioaminals provided analogues with a pyrimidine nucleobase. A kinetic bias favoring cyclization of the 1′,2′-*anti* thioaminal furnished the desired β-D-4′-thionucleoside analogue in a 7:1 ratio. DFT calculations suggest that this kinetic resolution originates from additional steric clash in the S_N_2-like transition state for 1′,4′-*trans* isomers, causing a significant decrease in their reaction rate relative to 1′,4′-*cis* counterparts. *N*-glycosylation of cyclic glycosyl donors with a purine nucleobase enabled the formation of novel 2-chloroadenine 4′-thionucleoside analogues. These proprietary molecules and other derivatives are currently being evaluated both in vitro and in vivo to establish their biological profiles.

## 1. Introduction

Natural nucleosides and nucleotides are involved in a plethora of biological processes including metabolism, cell signalling, and replication, all of which are often disrupted in various pathological conditions. Consequently, significant efforts have been dedicated to the development, synthesis, and investigation of nucleoside analogues with the aim of restoring normal cellular or organ homeostasis. These analogues act by competing with natural nucleosides with improved binding to targeted enzymes or receptors with some level of selectivity. This approach has led to the discovery of clinically important antiviral and anticancer agents [1,2]. Modifications of the furanose ring have been extensively studied, and new synthetic approaches are continuously being developed. Substituting the endocyclic oxygen with a sulfur provides 4′-thionucleoside analogues. The enhanced metabolic stability of 4′-thionucleosides or nucleotides towards phosphorylases, phosphatases, and hydrolysis justifies these structural alterations [3]. The presence of a larger and less electronegative heteroatom in the ring can lead to subtle changes in the anomeric effect and the conformation of the ring structure [4]. Consequently, these modifications may influence the biological behavior of a nucleoside compared to its 4′-thionucleoside counterpart.

**Scheme 1:**
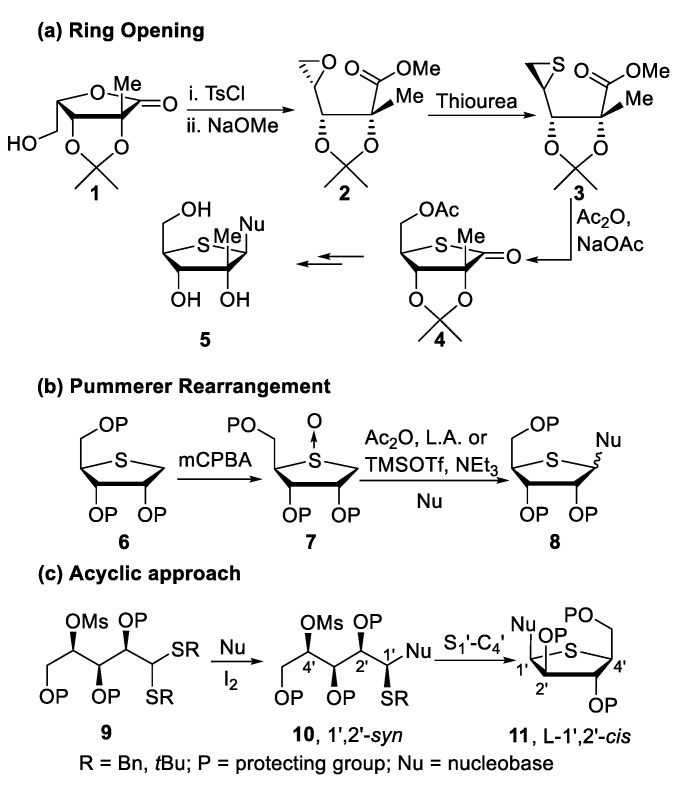
Synthesis of 4′-thionucleosides using (**a**) a ring-opening strategy, (**b**) a Pummerer rearrangement or (**c**) intramolecular cyclization of an acyclic thioaminal.

The synthesis of 4′-thionucleosides still requires improvement and the development of novel approaches, an objective pursued herein. The approaches for the synthesis of 4′-thionucleosides have been divided into three main categories (Scheme 1) [3,5]. The first involves a ring-opening strategy, exemplified by a synthesis from Liotta’s laboratory [6] involving the conversion of 2,3-acetonide protected lactone **1** to terminal thioepoxide **3**. The opening of the thioepoxide with sodium acetate generates thiofuranoside **4** with the subsequent formation of thionucleoside **5** (Scheme 1a). A second category involves Pummerer-type reactions with oxidation of tetrahydrothiophenes (**6**) followed by nucleobase addition onto in situ-generated thionium ions [7]. A third approach consists of an intramolecular cyclization of thioaminals (**10**) formed from stereoselective nucleobase addition onto acyclic dithioacetals **9**.

**Scheme 2:**
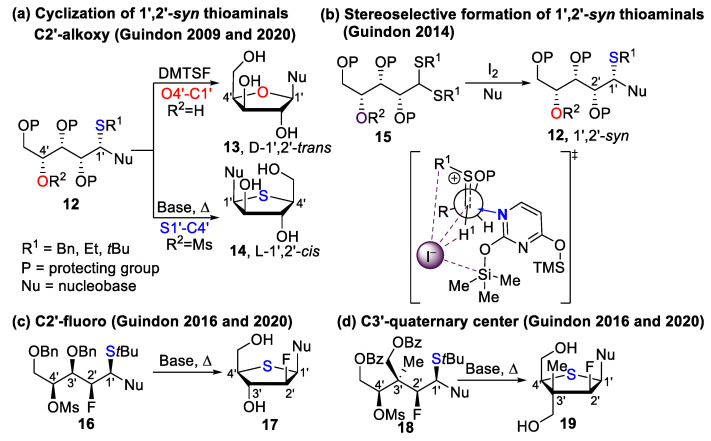
(**a**) Acyclic approach for the intramolecular cyclization of 1′,2′-*syn* thioaminals leading to either 1′,2′-*trans* furanosides through an O4′-to-C1′ cyclization or 1′,2′-*cis* 4′-thioanalogues through a S1′-C4′ cyclization [9,10]. (**b**) Transition state for the stereoselective addition of the silylated nucleobase [11]. (**c**) Acyclic approach for the synthesis of C2′ F 4′-thiofuranosides in addition to those bearing an all-carbon quaternary center at C3′ (**d**) [10,12].

Over the last decade, inspired by the acyclic strategy pioneered by Liotta’s synthesis of AZT [8], we have dedicated substantial efforts to developing a novel and complementary acyclic approach (Scheme 1c) for synthesizing nucleosides and 4′-thionucleoside analogues. From a conceptual standpoint, our approach takes advantage of a cyclization of an acyclic precursor already containing the nucleobase and a thioether at C1′ that may serve as a leaving group to give the corresponding nucleoside following an O4′-to-C1′ cyclization (Scheme 2a). Alternatively, when a leaving group is installed at C4′, the C1′ thioether may serve as a nucleophile resulting in 4′-thionucleosides through a S1′-to-C4′ cyclization. We demonstrated that both intramolecular cyclizations involve S_N_2-like nucleophilic displacements. In the O4′-to-C1′ displacement, the stereochemistry of the thioether at C1′ is inverted, whereas in the S1′-to-C4′ cyclization, the stereochemistry of the thioether at C1′ remains unaltered. Both cyclizations are very robust considering that a change in the C2′ and C3′ stereochemistry results in high levels of stereoselectivity and yield, regardless of the steric tension generated in the newly formed furanoside ring [9].

In the early stages of developing our acyclic strategy for nucleoside synthesis, both the 1′,2′-*syn* and *anti* thioaminals were prepared as a mixture from the corresponding dithioacetals. In presence of a C2 oxygen, activation at low temperatures with Me_2_S(SMe)BF_4_ or I_2_ resulted in a significant increase in 1′,2′-*syn* product formation [9,11]. DFT transition state calculations indicated that this selectivity could be attributed to the preferred addition opposite to the R group and the counteranion of thiocarbenium intermediates having the C2-alkoxy group gauche to the thioether moiety (Scheme 2b) [11]. With this approach, 4′-thionucleosides bearing a C2′-alkoxy or fluoride have been successfully synthesized in both the L- and D-series (Scheme 2a,c) [9,10,12].

Our interest in investigating acyclic carbon-centered free radicals and their reactivity in atom transfer reactions has enabled the generation of all-carbon stereogenic quaternary centers. These centers have been successfully incorporated at the C3′ or C2′ positions of furanoside scaffolds, leading to the development of novel families of nucleoside or nucleotide analogues (an example of which is depicted in Scheme 2d). The presence of an all-carbon quaternary center is expected to induce a conformational bias favoring a south conformation (DNA-like) when located at C3′ and a north conformation (RNA-like) when at C2′. The presence of the hydroxymethyl group on the quaternary center could act as an extended pharmacophore providing additional binding to proximal entities. Alternatively, nucleoside analogues bearing a C2′ or C3′ all-carbon quaternary center will not be recognized by enzymes or receptors susceptible to steric hinderance at these positions. These novel nucleosides bearing a quaternary center at C3′ have shown activities against gemcitabine-resistant KRAS mutated pancreatic cell lines [13,14]. C2′ analogues showed inhibition of SARS-CoV-2 RNA dependant RNA polymerase (RdRp), the causal virus of COVID-19 [15], while others have shown great promise as cardioprotective agents for the treatment of heart failure [16].

**Scheme 3:**
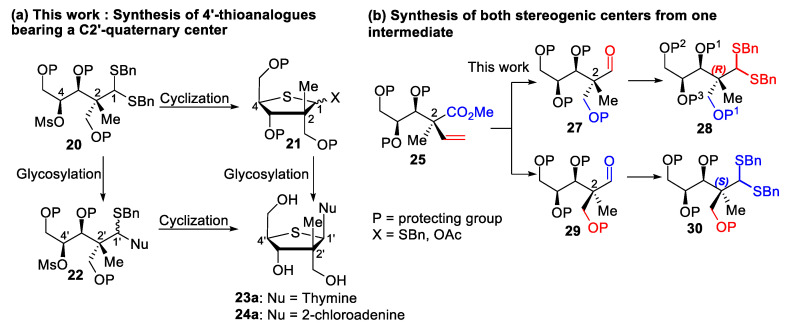
(**a**) This work focuses on the synthesis of 4′-thionucleoside analogues bearing a C2′ all-carbon quaternary center formed from intermediate **28.** (**b**) Potential to reach both C2′ stereochemistries.

Herein, we report the synthesis of 4′-thioanalogues bearing a C2′ all-carbon stereogenic center (Scheme 3a). From the onset, intrinsic challenges were recognized using our acyclic approach; namely, the C2′ quaternary center could hinder the desired cyclization. Our efforts towards the synthesis of the targeted 4′-thionucleosides using the acyclic approach with a pyrimidine nucleobase will be presented, in addition to a complementary cyclic approach, to access purine-bearing 4′-thioanalogues (Scheme 3a).

The formation of the C2′ all-carbon stereogenic center resulting from a vinyl atom transfer provides a single isomer **25**. This key intermediate can efficiently provide access to both the dithioacetals **28** or **30** with opposite stereochemistries at the C2′ quaternary center by derivatizing either the alkene or the ester moiety towards the required oxidation state for the C1′ anomeric center (Scheme 3b).

## 2. Results and Discussion

### 2.1. Acyclic Approach for the Synthesis of 4′-Thionucleoside Analogues

**Scheme 4:**
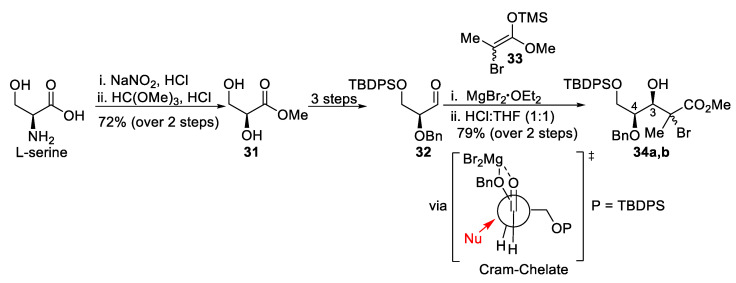
Formation of methylesters **34a,b** from a Cram-Chelate controlled Mukaiyama aldol reaction. ^‡^: The symbol is used to denote transition states.

The synthesis of the targeted novel 4′-thioanalogues required the construction of the key dithioacetal bearing the C2 all-carbon quaternary center. Following the literature procedures, aldehyde **32** was prepared in five steps from L-serine (Scheme 4) [17,18,19]. Aiming to generate a 3,4-*syn* diol, aldehyde **32** was engaged in a Cram-Chelate controlled Mukaiyama aldol reaction in the presence of a mixture of tetrasubstituted enoxysilanes **33** [20], and MgBr_2_·OEt_2_, a bidentate Lewis acid. The desired 3,4-*syn* products **34a,b** were formed in a >20:1 ratio with a 1:1 mixture of C2 bromides. The relative 3,4 stereochemistry was confirmed after removal of the silyl ether and lactonization (see Supplementary Information for further details). No efforts were invested in controlling the C2-selectivity as the generated tertiary bromides **34a,b** lead to a common radical species in the subsequent radical-based reaction.

**Scheme 5:**
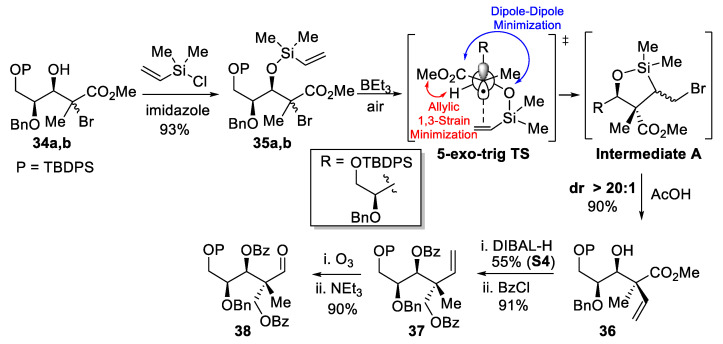
All-carbon quaternary center formation from a free-radical-based atom transfer reaction. ^‡^: The symbol is used to denote transition states.

The installation of vinyldimethylsilane on secondary alcohols **34a,b** provided a mixture of the corresponding silyl ethers **35a,b** (Scheme 5). This mixture was then subjected to a free-radical-based atom transfer reaction, using triethylborane as the initiator. Cyclization through the preferred 5-exo-trig diastereoselective transition state with carbon-carbon bond formation from the bottom face of the radical intermediate resulted in **intermediate A**, which was subsequently treated with AcOH for exclusive formation (>20:1) of methylester **36** [21]. DIBAL-H reduction of the methylester, benzoylation of the two alcohols, and ozonolysis of the vinyl moiety, provided aldehyde **38** in excellent yield.

The formation of the requisite dithioacetal was first attempted with t-butylthiol, but this only resulted in cyclized products **41a,b** (Table 1). Despite varying the equivalents of Lewis acid used (entries 1 and 2), debenzylation and cyclization was favored over the formation of dithioacetal **39**, presumably due to steric congestion. Using the less hindered benzyl mercaptan, dithioacetal **40** was formed in excellent yield (entry 3). 

Subsequent removal of the C4 benzyl ether from dithioacetal **40** proved to be difficult using boron-based Lewis acids, including Me_2_BBr [22], providing a mere 15% yield when using BCl_3_ (see Section 3). Reversing the order of these reactions was therefore considered. Hydrogenolysis of the C4-benzyl ether moiety of aldehyde **38** provided lactols **42a,b** in excellent yield (Table 2). Thioacetylation with BF_3_·OEt_2_ gave a mixture of cyclic thioacetals **44a,b** (entry 1), while SnCl_4_ pushed the equilibrium to provide 44% of the targeted dithioacetal **43** (entry 2). The use of TiCl_4_ then gave an excellent 80% yield (entry 3).

The addition of silylated thymine to C4-mesylated dithioacetal **45** in the presence of iodine resulted in a 1:1 mixture of thioaminals **46a,b** at room temperature or 50 °C (Table 3, entries 3 and 4), while lower temperatures allowed for a modest increase in formation of the 1′,2′-*anti* thioaminal **46a** (entries 1 and 2). The marginal stereoselectivities observed contrasted with the high 1′,2′-*syn* induction for the introduction of a nucleobase at C1 in the presence of an electron-withdrawing group adjacent to the dithioacetal. Nonetheless, the formation of these thioaminals provided the opportunity to examine the following cyclization step while exploring strategies to improve selectivity.

The two thioaminals were cyclized separately using NaI in the presence of 2,6-lutidine at reflux (Table 4). 1′,2′-*anti* thioaminal **46a** reacted accordingly to give the β-D-anomer **47a** in excellent yield (entry 1). The cyclization results were strikingly different for the 1′,2′-*syn* isomer **46b**, which yielded a low amount of α-D-anomer **47b** with recovery of starting material and a secondary product (**48**) isolated in 25% yield (entry 2). To confirm this difference in reactivity under identical conditions, a 1:1 mixture of thioaminals **46a,b** was submitted to the cyclization conditions (entry 3). A 7:1 ratio in favor of β-anomer **47a** was obtained, confirming the faster cyclization of the 1′,2′-*anti* isomer, and indicating the potential for developing a kinetic resolution strategy to address the absence of induction in nucleobase coupling to dithioacetals not bearing an electron-withdrawing group at the C2 position.

**Scheme 6:**
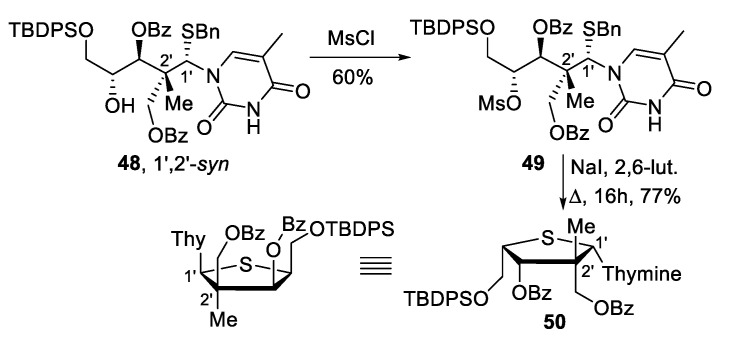
Cyclization of thioaminal **49**.

The secondary product **48** seemingly originated from displacement of the C4′-mesylate in 1′,2′-*syn* thioaminal **46b** with traces of water. To determine its structure, the C4′ hydroxyl of **48** was mesylated and treated with NaI in 2,6-lutidine (Scheme 6). Interestingly, the cyclization was very efficient, with the β-L-anomer **50** being the only isolated product in 77% yield.

### 2.2. DFT Computational Study

**Scheme 7:**
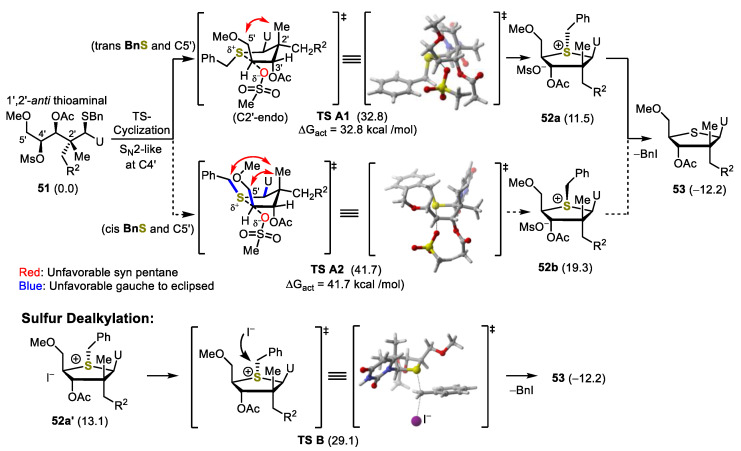
Relative Gibbs free energy (kcal/mol) calculated in Gaussian 16 [23] at the M06-2x/6-31G* [24,25] level of theory in 2,6-lutidine using the continuum solvation model (PCM) [26]. Cyclization of 1′,2′-*anti* thioaminal **51** as a model of **46a**. U = Uracil, ∆G_act_ corresponds to the TS free energy minus the lowest acyclic thioaminal **51** free energy. Otherwise, all free energies are arbitrarily referenced to the lowest energy acyclic conformer of compound **51** at 423.2 K. CYLview structures for TS are shown [27]. ^‡^: The symbol is used to denote transition states.

**Scheme 8:**
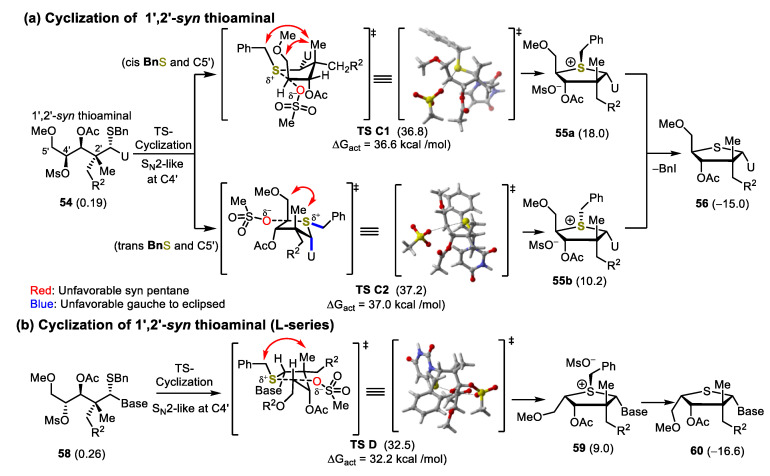
Relative Gibbs free energy (kcal/mol) profile in 2,6-lutidine using the continuum solvation model (PCM) for (**a**) cyclization of 1′,2′-*syn* thioaminal **54** as a model of **46b**, and (**b**) cyclization of 1′,2′-*syn* thioaminal **58** as a model of **49**. U = Uracil, ∆G_act_ corresponds to the TS free energy difference with the lowest corresponding acyclic thioaminal free energy **54** and **58**. Otherwise, all free energies are arbitrarily referenced to the lowest energy acyclic conformer of compound **51** at 423.2 K (refer to Scheme 7). CYLview structures for TS are shown [27]. ^‡^: The symbol is used to denote transition states.

With the aim of identifying the principle steric and electronic factors influencing the rates of cyclizations in thioaminals having different relative stereochemistries, we examined model compounds **51**, **54** and **58** through DFT calculations (Scheme 7 and Scheme 8). The calculated energy landscape was first explored with **51** and was consistent with rate-limiting intramolecular displacement of the C4′-mesylate, generating sulfonium intermediate **52a** (**TS A1**, 32.8 kcal/mol) with the -SBn chain in the bottom position, trans to the C5′ center. Dealkylation in the presence of iodide (i.e., **TS B**, Scheme 7) would then furnish product **53** through **TS B** (29.1 kcal/mol). **TS A2**, with the -SBn occupying the upper position, exhibited significantly higher energy (41.7 kcal/mol) due to a severe steric clash between the -SBn chain and both the C5′ and base substituents. A noteworthy observation is that the conformation of the examined 4′-thiofuranoside shows lower energy with the C2′-endo envelop having the C3′-OAc oriented in the pseudo-axial conformation, perpendicular to the axis of the S_N_2-like bond breaking and bond forming. This could be rationalized by favorable stabilization achieved through the orientation of the best C3′-H3′ and C3′-C2′ sigma donors towards the C4′ center, as confirmed by NBO analysis. This orientation could also relieve electrostatic repulsion by distancing the leaving group (-OMs) and the C3′ acetate group. Even in **TS A2**, which experiences severe strain on the upper face with the C2′-Me axial, the C2′-endo conformation at 41.7 kcal/mol is preferable to the corresponding C2′-exo TS, where the top face C2′-Me is in a pseudo-equatorial position, with an energy of 44.9 kcal/mol (see Supplementary Information).

Consistent with the observed slower cyclization for the 1′,2′-*syn* thioaminal **46b** (model compound **54**, Scheme 8a), the calculated TS energies for the cyclization through **TS C1** (36.8 kcal/mol) with the benzyl group up or **TS C2** (37.2 kcal/mol) with the benzyl group down are significantly higher in energy than the lowest **TS A1** for the 1′,2′-*anti* thioaminal (32.8 kcal/mol, Scheme 7). Both **TS C1** and **TS C2** suffer, respectively, from either additional syn pentane interactions (SBn and C2′-Me moiety) or from an additional gauche interaction (Uracil and SBn). In **TS C2**, the nucleobase is also forced to occupy a less favorable pseudo-axial position. The acyclic precursor **54** minima was found to be slightly higher than for **51**, leading to a predicted activation energy of 36.6 kcal/mol and therefore slower kinetics for 1′,4′-*trans* thiofuranoside formation. Interestingly, previous cyclizations of thioaminals with 1′,4′-*cis* and 1′,4′-*trans* stereochemistries not bearing the C2′ quaternary group displayed similar rates of reactivity [9]. The syn-pentane steric clashes, therefore, seem to impact more severely the reactivity of the isomers leading to 1′,4′-*trans* thionucleosides. This was further confirmed in the formation of L-1′,4′-*cis* thiofuranoside **60**. This cyclization was observed experimentally to progress readily (Scheme 6 and Scheme 8b), in accordance with a TS energy (**TS D**, 32.5 kcal/mol) comparable to **TS A1**. The starting thioaminal **58** was 0.26 kcal/mol higher than **51**, therefore leading to a calculated ∆G_act_ of 32.2 kcal/mol.

**Scheme 9:**
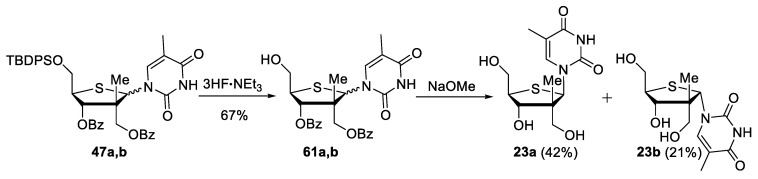
Synthesis of final 4′-thionucleoside analogues bearing a thymine nucleobase.

These studies shed light on why the S1′-to-C4′ cyclization can lead to a kinetic resolution favoring formation of the biologically relevant 1′,4′-*cis* thionucleoside. In the context of generating the targeted analogues presented here, deprotection of the primary silyl group of a mixture of 4′-thionucleosides **47a,b** using 3HF·NEt_3_ provided an inseparable mixture of anomers **61a,b**. Following benzoate removal with NaOMe, the final molecules **23a** and **23b** were isolated in 42% and 21%, respectively (Scheme 9).

### 2.3. Cyclic Approach for the Synthesis of 4′-Thionucleoside Analogues

**Scheme 10:**
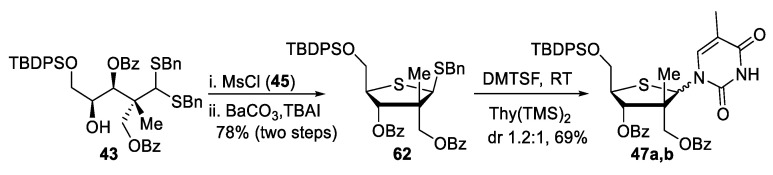
Synthesis of thymine-bearing 4′-thionucleoside analogues from glycosylation of cyclic thiofuranoside **62**.

As discussed in Scheme 3, a second approach was considered to access such 4′-thioanalogues, in which the thiofuranoside was formed prior to addition of the nucleobase. After mesylation of dithioacetal **43**, treatment with TBAI in the presence of a base provided thiobenzylfuranoside **62** (Scheme 10) [28]. Glycosylation using silylated thymine in the presence of DMTSF provided a 1.2:1 ratio of thioanalogues **47a,b**.

A similar cyclic strategy was used to prepare purine derivatives, the synthesis of which was difficult using the acyclic approach. The addition of 2-chloroadenine was investigated, as the presence of a halogen at the two position of the nucleobase renders analogues, such as Clofarabine, more stable to deamination, a major mechanism of metabolic clearance in vivo [29]. Similar to the addition of thymine, the activation of thiofuranoside **62** with DMTSF followed by the addition of 2-chloroadenine or 2,6-dichloropurine resulted in a mixture of compounds with the major products identified as a 1:1 mixture of N9-β:α anomers (results not shown). The nucleobase addition with thiofuranosides **63a,b** bearing an anomeric acetate was next investigated (Table 5).

**Scheme 11:**
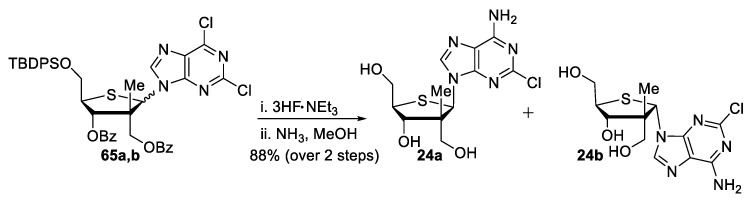
Synthesis of final 4′-thionucleoside analogues bearing a 2-chloroadenine nucleobase.

Similar to glycosylation of thiofuranoside **62**, the addition of 2-chloroadenine to **63a,b** resulted in a mixture of compounds with the major products identified as a 1:1 mixture of N9-β:α anomers **64a,b** at RT or 84 °C (entries 1 and 2). However, a 5:1 ratio of N9-products **65a,b** in favor of the desired β-anomer was obtained with 2,6-dichloropurine using DBU and TMSOTf at 84 °C versus a 1:1 ratio at room temperature (entries 3 and 4), indicative of a thermodynamic equilibrium favoring the β-anomer. The removal of the C5′-silyl ether of anomers **65a,b** followed by debenzoylation and displacement of the 6-chloro moiety with ammonia provided the corresponding 6-amino derivatives **24a** and **24b** (Scheme 11).

In conclusion, nucleoside analogue synthesis is a research field of great interest in medicinal chemistry. The intramolecular cyclization of acyclic thioaminals has been used to synthesize nucleosides as well as 4′-thioanalogues. Herein, this approach was evaluated in the context of a novel family of 4′-thionucleosides bearing a quaternary stereogenic center at C2′. The challenge of this study resides in the S_N_2-like S1′-to-C4′ cyclization combined with stereoselective formation of the desired thioaminal typically dependent on the presence of an electron-withdrawing group at C2′, which in this case is absent. The lack of diastereoselectivity for thioaminal formation turned out to be significant, with a modest stereoselectivity (1.4:1) favoring the 1′,2′-*anti* isomer. Interestingly, an original solution arose from these challenges. A kinetic bias favoring cyclization of the 1′,2′-*anti* thioaminal was observed, with the desired β-anomer being obtained in a 7:1 ratio. DFT calculations suggest that this kinetic resolution favors the 1′,4′-*cis* product due to significant steric clashes arising in the S_N_2 TS of the 1′,4′-*trans* isomer. These unfavorable interactions increase the activation energy, resulting in a slower rate of cyclization as compared to the corresponding 1′,4′-*cis* isomers. An alternative approach, in which the nucleobase was added onto an already formed thiofuranoside, allowed for the synthesis of novel 2-chloroadenine 4′-thionucleoside analogues. These proprietary molecules and other derivatives are currently being evaluated both in vitro and in vivo for their biological profiles, more specifically in the context of cardioprotection. These novel nucleoside scaffolds could potentially also find interesting applications in synthetic vaccine development.

## 3. Materials and Methods

### 3.1. General Information—Synthesis

All reactions requiring anhydrous conditions were carried out under an atmosphere of nitrogen or argon in flame-dried glassware using standard syringe techniques. All anhydrous solvents were dried with 3 Å molecular sieves prior to use. The 3 Å molecular sieves (1–2 mm beads) were activated by being heated at 180 °C for 48 h under vacuum prior to being added to new bottles of solvent purged with nitrogen. Commercially available reagents were used as received. Flash chromatography was performed on silica gel 60 (0.040–0.063 mm) using forced flow (flash chromatography) of the indicated solvent system or an automated flash purification system. Analytical thin-layer chromatography (TLC) was carried out on precoated (0.25 mm) silica gel aluminum plates. Visualization was performed with short-wavelength UV and/or revealed with potassium permanganate solutions. ^1^H NMR spectra were recorded at room temperature at 500 MHz and ^13^C were recorded at 126 MHz. The data are reported as follows: chemical shift in parts per million referenced to residual solvent (CDCl_3_ δ 7.26 ppm, CD_3_OD δ 3.31 ppm), multiplicity (s = singlet, d = doublet, dd = doublet of doublets, ddd = doublet of doublets of doublets, t = triplet, td = triplet of doublets, m = multiplet, app = apparent), coupling constants (hertz), and integration. ^13^C{^1^H}MR spectra were recorded at room temperature using 126 MHz. The data are reported as follows: chemical shift in parts per million referenced to residual solvent (CDCl_3_ δ 77.16 ppm, CD_3_OD δ 49.00 ppm). Infrared spectra were recorded on a Fourier-transform infrared spectrophotometer with a single-reflection diamond ATR module, and signals were reported in cm^−1^. Mass spectra were recorded through electrospray ionization positive-ion mode. A Hybrid Quadrupole-Orbitrap mass analyzer was used for HRMS measurements. Optical rotations were measured at room temperature from the sodium D line (589 nm) using CHCl_3_ as solvent unless otherwise noted, and calculated using the following formula: [α]_D_ = (100)α_obs_/(l·c)), where c = (g of substrate/100 mL of solvent) and l = 1 dm. Diol **31**, methylester **S1** and aldehyde **32** were prepared using previously reported procedures [17,18,19].

### 3.2. Experimental Synthetic Procedures

(–)-Methyl (*S*)-2-(benzyloxy)-3-((tert-butyldiphenylsilyl)oxy)propanoate (**S2**).


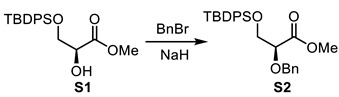


To a solution of alcohol **S1** [18] (46.2 g, 1.00 equiv, 129 mmol) in anhydrous DMF (0.57 L, 0.23 M) at 0 °C, BnBr (30.6 mL, 2.00 equiv, 258 mmol) and NaH (5.67 g, 1.10 equiv, 142 mmol, 60% oil dispersion) were added. The reaction mixture was stirred at room temperature for 3 h. After cooling to 0 °C, water was added. The aqueous layer was extracted (3× with EtOAc and the combined organic layers were washed with brine, dried over MgSO_4_, filtered, and concentrated under reduced pressure. Purification by flash chromatography (Hexanes/EtOAc) provided methyl ester **S2** (57.1 g, 98% yield). ^1^H NMR and optical rotation correlate with the previously reported data for this compound [19]. [α]_D_^25^ −17 (*c* 2.9, CHCl_3_); literature value [19] [α]_D_^23^ −19.0 (c = 1.5, CHCl_3_); ^1^H NMR (500 MHz, CDCl_3_) δ 7.71–7.64 (m, 4H), 7.44–7.28 (m, 11H), 4.75 (d, *J* = 11.9 Hz, 1H), 4.53 (d, *J* = 11.9 Hz, 1H), 4.14 (t, *J* = 5.1 Hz, 1H), 3.99–3.91 (m, 2H), 3.75 (s, 3H), 1.04 (s, 9H) ppm.

(+)-Methyl (2*S*,3*R*,4*S*)-4-(benzyloxy)-2-bromo-5-((tert-butyldiphenylsilyl)oxy)-3-hydroxy-2-methylpentanoate (**34a**) and (+)-Methyl (2*R*,3*R*,4*S*)-4-(benzyloxy)-2-bromo-5-((tert-butyldiphenylsilyl)oxy)-3-hydroxy-2-methylpentanoate (**34b**). To a stirred solution of aldehyde **32** [19] (206 mg, 1.00 equiv, 0.492 mmol) in CH_2_Cl_2_ (0.74 mL, 0.66 M) at −78 °C, MgBr_2_·OEt_2_ (635 mg, 5.00 equiv, 2.46 mmol) was added. The reaction mixture was stirred for 15 min at −78 °C followed by slow addition of crude methyl ((2-bromo-1-methoxyprop-1-en-1-yl)oxy)trimethylsilane **33** [20] (0.19 mL, 2.0 equiv, 0.98 mmol). The reaction mixture was stirred at −78 °C for 1 h. A mixture of 1N HCl/THF (1:1, *v*/*v*, 2.80 mL) was added followed by gradual warming to room temperature with stirring for 1 h. The aqueous layer was extracted (3×) with Et_2_O and the combined organic layers were washed with a saturated solution of NaHCO_3_, dried over MgSO_4_, filtered, and concentrated under reduced pressure. ^1^H NMR of the crude reaction indicated a >20:1 ratio of 3,4-*syn*:3,4-*anti* products with a 1:1 mixture of C2-bromides **34a** and **34b**. Purification by flash chromatography (Hexanes/EtOAc) allowed for the two C2 diastereomers to be separated providing **34a** (126 mg) and **34b** (101 mg) for a combined yield of 79%. The 3,4-*syn* and C2 stereochemistry were assigned from lactonization of **34a** (see below and Supplementary Information). **34a**: R_f_ = 0.46 (Hexanes/EtOAc, 80:20); [α]_D_^25^ + 11 (c 3.0, CH_2_Cl_2_); formula: C_30_H_37_BrO_5_Si; MW: 585.61 g/mol; IR (neat) ν_max_ 3533, 3070, 2999, 2953, 2858, 1742, 1472, 1249, 1112 cm^−1^; ^1^H NMR (500 MHz, CDCl_3_) δ 7.72–7.67 (m, 4H), 7.48–7.42 (m, 2H), 7.42–7.38 (m, 4H), 7.33–7.27 (m, 3H), 7.22–7.19 (m, 2H), 4.65 (d, *J* = 10.9 Hz, 1H), 4.41 (d, *J* = 10.9 Hz, 1H), 4.29 (d, *J* = 9.7 Hz, 1H), 3.97 (appt, *J* = 6.1 Hz, 1H), 3.86–3.84 (m, 2H), 3.67 (s, 3H), 3.41 (d, *J* = 9.7 Hz, 1H), 1.86 (s, 3H), 1.08 (s, 9H) ppm; ^13^C NMR (126 MHz, CDCl_3_) δ 171.3, 137.5, 135.80 (2C), 135.77 (2C), 133.3, 133.1, 130.02, 129.99, 128.5 (2C), 128.14 (2C), 128.10, 128.0 (2C), 127.9 (2C), 76.8, 74.8, 72.8, 63.8, 61.6, 53.2, 27.0 (3C), 23.5, 19.3 ppm; HRMS (ESI) *m*/*z*: calcd for C_30_H_38_BrO_5_Si [M+H]^+^ 585.1666, found 585.1660 (–1.03 ppm). **34b**: R_f_ = 0.95 (Hexanes/EtOAc 50:50); [α]_D_^25^ + 9.2 (c 2.4, CH_2_Cl_2_); formula: C_30_H_37_BrO_5_Si; MW: 585.6100 g/mol; IR (neat) ν_max_ 3552, 3070, 2953, 2932, 2858, 1737, 1472, 1428, 1261, 1209, 1112 cm^−1^; ^1^H NMR (500 MHz, CDCl_3_) δ 7.75–7.71 (m, 4H), 7.48 (dd, *J* = 7.3, 1.3 Hz, 2H), 7.46–7.42 (m, 4H), 7.36–7.29 (m, 3H), 7.27–7.24 (m, 2H), 4.61 (d, *J* = 10.9 Hz, 1H), 4.42 (d, *J* = 11.0 Hz, 1H), 4.19 (d, *J* = 10.4 Hz), 4.05–4.01 (m, 1H), 3.87–3.84 (m, 2H), 3.54 (s, 3H), 3.19 (d, *J* = 10.3 Hz, 1H), 2.08 (s, 3H), 1.12 (s, 9H) ppm; ^13^C NMR (126 MHz, CDCl_3_) δ 170.8, 137.7, 135.8 (2C), 135.7 (2C), 133.3, 133.2, 130.0, 129.96, 128.4 (2C), 128.0 (2C), 127.94 (4C), 127.91, 77.5, 76.9, 72.9, 65.7, 63.3, 53.3, 27.2, 27.0 (3C), 19.3 ppm; HRMS (ESI) *m*/*z*: calcd for C_30_H_38_BrO_5_Si [M+H]^+^ 585.1666, found 585.1666 (0 ppm).

(+)-(3*S*,4*R*,5*S*)-5-(benzyloxy)-3-bromo-4-hydroxy-3-methyltetrahydro-2H-pyran-2-one (**S3**).


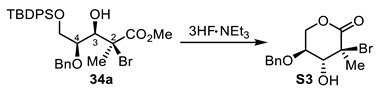


To a stirred solution of methyl ester **34a** (72 mg, 1.0 equiv, 0.12 mmol) in THF (0.49 mL, 0.25 M) at 0 °C, 3HF·NEt_3_ (0.047 mL, 2.30 equiv, 0.283 mmol) was added. The reaction mixture was stirred at room temperature for 16 h and then quenched by addition of a saturated solution of NaHCO_3_. The aqueous layer was extracted (3×) with EtOAc and the combined organic layers were washed with brine, dried over MgSO_4_, filtered, and concentrated under reduced pressure. Purification by flash chromatography (Hexanes/EtOAc) provided lactone **S3** (22 mg, 57% yield). The 3,4 and C2 stereochemistry were determined by the relevant nuclear Overhauser effect (nOe) enhancements, as depicted in the SI. R_f_ = 0.66 (Hexanes/EtOAc, 50:50); [α]_D_^25^ +33 (*c* 2.2, CH_2_Cl_2_); formula: C_13_H_15_O_4_Br; MW: 315.1630 g/mol; IR (neat) ν_max_ 3451, 2925, 2869, 1742, 1455, 1389, 1282, 1228, 1090 cm^−1^; ^1^H NMR (500 MHz, CDCl_3_) δ 7.41–7.32 (m, 5H), 4.71 (d, *J* = 11.7 Hz, 1H), 4.69 (d, *J* = 11.7 Hz, 1H), 4.66 (dd, *J* = 12.2, 5.6 Hz, 1H), 4.19 (dd, *J* = 12.2, 5.7 Hz, 1H), 3.96 (dt, *J* = 7.1, 5.6 Hz, 1H), 3.59 (dd, *J* = 7.0, 5.5 Hz, 1H), 2.67 (d, *J* = 5.8 Hz, 1H), 2.05 (s, 3H) ppm; ^13^C NMR (126 MHz, CDCl_3_) δ 168.1, 137.2, 128.9 (2C), 128.5, 128.1 (2C), 76.2, 75.8, 73.0, 68.8, 60.1, 26.3 ppm; HRMS (ESI) *m*/*z*: calcd for C_13_H_16_O_4_Br [M+H]^+^ 315.0226, found 315.0226 (0 ppm).

(–)-Methyl (2*S*,3*R*,4*S*)-4-(benzyloxy)-2-bromo-5-((tert-butyldiphenylsilyl)oxy)-3-((dimethyl (vinyl)silyl)oxy)-2-methylpentanoate (**35a**). To a stirred solution of alcohol **34a** (56 mg, 1.0 equiv, 0.096 mmol) in CH_2_Cl_2_ (0.24 mL, 0.40 M) at 0 °C, imidazole (22 mg, 3.4 equiv, 0.33 mmol) was added, followed by chloro(dimethyl)vinylsilane (0.022 mL, 1.5 equiv, 0.14 mmol). The reaction mixture was stirred at room temperature for 19 h and then quenched by the addition of water. The aqueous layer was extracted (3×) with CH_2_Cl_2_ and the combined organic layers were washed with water, dried over MgSO_4_, filtered, and concentrated under reduced pressure. Purification by flash chromatography (Hexanes/EtOAc) provided **35a** (53 mg, 83% yield). R_f_ = 0.93 (Hexanes/EtOAc, 30:70); [α]_D_^25^ −8.7 (*c* 4.9, CH_2_Cl_2_); formula: C_34_H_45_BrO_5_Si_2_; MW: 669.8030 g/mol; IR (neat) ν_max_ 3070, 3049, 2999, 2953, 2858, 1742, 1454, 1252, 1127 cm^−1^; ^1^H NMR (500 MHz, CDCl_3_) δ 7.73–7.66 (m, 5H), 7.47–7.43 (m, 2H), 7.43–7.36 (m, 4H), 7.25–7.18 (m, 4H), 6.06 (dd, *J* = 20.4, 14.9 Hz, 1H), 5.88 (dd, *J* = 14.9, 3.8 Hz, 1H), 5.66 (dd, *J* = 20.4, 3.8 Hz, 1H), 4.66 (d, *J* = 1.6 Hz, 1H), 4.45 (d, *J* = 11.7 Hz, 1H), 4.41 (d, *J* = 11.7 Hz, 1H), 4.09 (ddd, *J* = 7.3, 5.9, 1.6 Hz, 1H), 3.77 (dd, *J* = 10.7, 5.9 Hz, 1H), 3.74 (s, 3H), 3.72 (dd, *J* = 10.5, 7.1 Hz, 1H), 1.94 (s, 3H), 1.09 (s, 9H), 0.15 (s, 3H), 0.12 (s, 3H) ppm; ^13^C NMR (126 MHz, CDCl_3_) δ 171.6, 138.5, 137.9, 136.0 (2C), 135.8 (2C), 133.5, 133.4, 133.0, 130.0, 129.9, 128.3 (2C), 127.89 (4C), 127.85 (2C), 127.5, 78.5, 74.7, 72.6, 63.6, 62.3, 53.2, 27.1 (3C), 22.6, 19.3, −1.1, −1.3 ppm; HRMS (ESI) *m*/*z*: calcd for C_34_H_46_BrO_5_Si_2_ [M+H]^+^ 669.2062, found 669.2058 (−0.60 ppm).

(+)-Methyl (2*R*,3*R*,4*S*)-4-(benzyloxy)-2-bromo-5-((tert-butyldiphenylsilyl)oxy)-3-((dimethyl (vinyl)silyl)oxy)-2-methylpentanoate (**35b**). To a stirred solution of alcohol **34b** (40 mg, 1.0 equiv, 0.068 mmol) in CH_2_Cl_2_ (0.17 mL, 0.40 M) at 0 °C, imidazole (16 mg, 3.4 equiv, 0.23 mmol) was added, followed by chloro(dimethyl)vinylsilane (0.016 mL, 1.5 equiv, 0.10 mmol). The reaction mixture was stirred at room temperature for 19 h and then quenched by the addition of water. The aqueous layer was extracted (3×) with CH_2_Cl_2_ and the combined organic layers were washed with water, dried over MgSO_4_, filtered, and concentrated under reduced pressure. Purification by flash chromatography (Hexanes/EtOAc) provided **35b** (20 mg, 44% yield). R_f_ = 0.96 (Hexanes/EtOAc, 30:70); [α]_D_^25^ +4.2 (*c* 2.0, CH_2_Cl_2_); formula: C_34_H_45_BrO_5_Si_2_; MW: 669.8030 g/mol; IR (neat) ν_max_ 3070, 3032, 2953, 2858, 1739, 1472, 1254, 1154, 1110 cm^−1^; ^1^H NMR (500 MHz, CDCl_3_) 7.69–7.63 (m, 5H), 7.47–7.42 (m, 2H), 7.42–7.36 (m, 5H), 7.25–7.23 (m, 1H), 7.16–7.13 (m, 2H), 6.20 (dd, *J* = 20.5, 14.9 Hz, 1H), 5.94 (dd, *J* = 14.9, 3.7 Hz, 1H), 5.71 (dd, *J* = 20.5, 3.7 Hz, 1H), 4.58 (d, *J* = 2.6 Hz, 1H), 4.41 (d, *J* = 11.4 Hz, 1H), 4.24 (d, *J* = 11.4 Hz, 1H), 3.78–3.69 (m, 2H), 3.62 (s, 3H), 3.58 (td, *J* = 6.0, 2.6 Hz, 1H), 1.92 (s, 3H), 1.07 (s, 9H), 0.29 (s, 3H), 0.25 (s, 3H) ppm; ^13^C NMR (126 MHz, CDCl_3_) δ 171.0, 137.9, 137.8, 135.8 (2C), 135.7 (2C), 133.39, 133.36, 133.3, 130.01, 129.95, 128.33 (2C), 128.29 (2C), 127.91 (2C), 127.90 (2C), 127.7, 80.2, 77.1, 73.0, 66.7, 63.8, 53.0, 27.0 (3C), 24.3, 19.3, −0.8, −1.3 ppm; HRMS (ESI) *m*/*z*: calcd for C_34_H_46_BrO_5_Si_2_ [M+H]^+^ 669.2062, found 669.2052 (–1.49 ppm).

(+)-Methyl(2*S*,3*S*,4*S*)-4-(benzyloxy)-5-((tert-butyldiphenylsilyl)oxy)-3-hydroxy-2-methyl-2-vinylpentanoate (**36**). To a stirred solution of C2-bromo esters **35a,b** (58 mg, 1.0 equiv, 0.087 mmol) in toluene (1.5 mL, 0.060 M) at 0 °C, BEt_3_ (0.17 mL, 2.0 equiv, 0.17 mmol, 1.0 M solution in hexanes) was added over 1 h in an open-air system. The reaction mixture was stirred for 1 h at 0 °C followed by the addition of acetic acid (10 μL, 2.0 equiv, 0.17 mmol) and MeOH (1.5 mL), with gradual warming to room temperature and stirring for an additional 30 min. The mixture was concentrated under reduced pressure. ^1^H NMR analysis of the crude mixture indicated a >20:1 diastereomeric ratio. Purification by flash chromatography (Hexanes/EtOAc) provided methyl ester **36** (26 mg, 57% yield). The 90% yield shown in Scheme 5 was obtained on a larger 9 g scale. R_f_ = 0.22 (Hexanes/EtOAc, 90:10); [α]_D_^25^ +7.8 (*c* 1.6, MeOH); formula: C_32_H_40_O_5_Si; MW: 532.7520 g/mol; IR (neat) ν_max_ 3561, 2952, 2858, 1732, 1456, 1192 cm^−1^; ^1^H NMR (500 MHz, CDCl_3_) δ 7.68–7.65 (m, 4H), 7.46–7.41 (m, 2H), 7.40–7.36 (m, 4H), 7.31–7.24 (m, 3H), 7.22–7.20 (m, 2H), 6.33 (dd, *J* = 17.7, 11.0 Hz, 1H), 5.24 (d, *J* = 11.1 Hz, 1H), 5.18 (d, *J* = 17.7 Hz, 1H), 4.55 (d, *J* = 10.9 Hz, 1H), 4.32 (d, *J* = 10.9 Hz, 1H), 3.85–3.76 (m, 3H), 3.59–3.54 (m, 1H), 3.45 (s, 3H), 3.20 (d, *J* = 10.2 Hz, 1H), 1.39 (s, 3H), 1.05 (s, 9H) ppm; ^13^C NMR (126 MHz, CDCl_3_) δ 175.6, 138.9, 138.0, 135.8 (4C), 133.5, 133.3, 129.94, 129.91, 128.4 (2C), 128.0 (2C), 127.90 (2C), 127.89 (2C), 127.81, 115.0, 78.0, 76.9, 72.8, 63.6, 52.1, 51.7, 27.0 (3C), 19.4, 19.3 ppm; HRMS (ESI) *m*/*z*: calcd for C_32_H_41_O_5_Si [M+H]^+^ 533.2718, found 533.2720 (+0.38 ppm).

(+)-(2*R*,3*S*,4*S*)-4-(benzyloxy)-5-((tert-butyldiphenylsilyl)oxy)-2-methyl-2-vinylpentane-1,3-diol (**S4**).


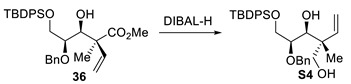


To a stirred solution of methyl ester **36** (63 mg, 1.0 equiv, 0.12 mmol) in CH_2_Cl_2_ (0.91 mL, 0.13 M) at −78 °C, DIBAl-H (1 M in Hexanes, 0.47 mL, 4.0 equiv, 0.47 mmol) was added dropwise. The reaction mixture was stirred at −40 °C for 3 h and then quenched by the addition of a saturated aqueous solution of potassium sodium tartrate at −78 °C, followed by gradual warming to room temperature and stirring for 1 h. The aqueous layer was extracted (3×) with EtOAc and the combined organic layers were washed with brine, dried over MgSO_4_, filtered, and concentrated under reduced pressure. Purification by flash chromatography (Hexanes/EtOAc) provided diol **S4** (33 mg, 55% yield). R_f_ = 0.60 (Hexanes/EtOAc, 50:50); [α]_D_^25^ +18 (c 2.2, CH_2_Cl_2_); formula: C_31_H_40_O_4_Si; MW: 504.7420 g/mol; IR (neat) ν_max_ 3453, 3070, 3032, 2858, 1456, 1112 cm^−1^; ^1^H NMR (500 MHz, CDCl_3_) δ 7.71–7.63 (m, 4H), 7.47–7.42 (m, 2H), 7.41–7.36 (m, 4H), 7.33–7.27 (m, 3H), 7.24–7.22 (m, 2H), 6.06 (dd, *J* = 17.8, 11.0 Hz, 1H), 5.16 (dd, *J* = 11.0, 1.5 Hz, 1H), 5.11 (dd, *J* = 17.7, 1.6 Hz, 1H), 4.53 (d, *J* = 11.0 Hz, 1H), 4.38 (d, *J* = 11.0 Hz, 1H), 3.78 (dd, *J* = 8.6, 4.6 Hz, 1H), 3.75 (dd, *J* = 8.7, 3.6 Hz, 1H), 3.72 (d, *J* = 7.8 Hz, 1H), 3.63 (dd, *J* = 11.0, 7.8 Hz, 1H), 3.59 (dd, *J* = 10.9, 4.8 Hz, 1H), 3.51 (appt, *J* = 5.9 Hz, 1H), 3.05 (d, *J* = 8.1 Hz, 1H), 2.61 (dd, *J* = 7.8, 4.9 Hz, 1H), 1.07 (s, 9H), 1.02 (s, 3H) ppm; ^13^C NMR (126 MHz, CDCl_3_) δ 140.4, 137.7, 135.77 (2C), 135.76 (2C), 133.3, 133.2, 130.02, 130.00, 128.5 (2C), 128.1 (2C), 128.0, 127.9 (4C), 114.5, 77.1, 76.7, 72.6, 69.8, 63.9, 45.5, 27.0 (3C), 19.3, 19.0 ppm; HRMS (ESI) *m*/*z*: calcd for C_31_H_40_NaO_4_Si [M+Na]^+^ 527.2588, found 527.2587 (–0.19 ppm).

(–)-(2*R*,3*S*,4*S*)-4-(benzyloxy)-5-((tert-butyldiphenylsilyl)oxy)-2-methyl-2-vinylpentane-1,3-diyl dibenzoate (**37**). To a stirred solution of diol **S4** (115 mg, 1.00 equiv, 0.228 mmol) in CH_2_Cl_2_ (1.6 mL, 0.15 M) at room temperature, DMAP (2.8 mg, 0.10 equiv, 0.023 mmol) and pyridine (0.11 mL, 6.0 equiv, 1.4 mmol) were added. The mixture was cooled to 0 °C and BzCl (0.079 mL, 3.0 equiv, 0.68 mmol) was added slowly. The reaction mixture was warmed to room temperature for 16 h. After cooling to 0 °C, ethylenediamine (0.038 mL, 2.5 equiv, 0.57 mmol) was added and stirred for 30 min at 0 °C. Upon warming to room temperature, the aqueous layer was extracted (3×) with EtOAc and the combined organic layers were washed with brine, dried over MgSO_4_, filtered, and concentrated under reduced pressure. Purification by flash chromatography (Hexanes/EtOAc) provided alkene **37** (148 mg, 91% yield). R_f_ = 0.88 (Hexanes/EtOAc, 50:50); [α]_D_^25^ −35 (*c* 7.4, CH_2_Cl_2_); formula: C_45_H_48_O_6_Si; MW: 712.9580 g/mol; IR (neat) ν_max_ 3070, 2930, 2857, 1719, 1264, 1106 cm^−1^; ^1^H NMR (500 MHz, CDCl_3_) δ 8.12–8.03 (m, 4H), 7.70–7.62 (m, 4H), 7.59–7.52 (m, 2H), 7.43 (dd, *J* = 13.0, 7.7 Hz, 5H), 7.40–7.35 (m, 5H), 7.31–7.26 (m, 4H), 7.25–7.23 (m, 1H), 6.22 (dd, *J* = 17.7, 11.0 Hz, 1H), 5.67 (apps, 1H), 5.24–5.17 (m, 2H), 4.54 (d, *J* = 11.4 Hz, 1H), 4.49 (d, *J* = 11.5 Hz, 1H), 4.41 (d, *J* = 11.1 Hz, 1H), 4.31 (d, *J* = 11.1 Hz, 1H), 3.87–3.76 (m, 2H), 3.67 (appdd, *J* = 9.8, 6.3 Hz, 1H), 1.30 (s, 3H), 1.07 (s, 9H) ppm; ^13^C NMR (126 MHz, CDCl_3_) δ 166.5, 165.8, 140.1, 138.3, 135.8 (2C), 135.7 (2C), 133.4, 133.2, 133.1, 133.0, 130.4, 130.2, 130.0 (2C), 129.84, 129.77 (2C), 129.69, 128.50 (2C), 128.48 (2C), 128.3 (2C), 127.84 (2C), 127.77 (2C), 127.75 (2C), 127.6, 115.4, 78.3, 75.0, 72.6, 68.9, 63.1, 44.7, 26.9 (3C), 19.3, 19.2 ppm; HRMS (ESI) *m*/*z*: calcd for C_45_H_48_NaO_6_Si [M+Na]^+^ 735.3112, found 735.3124 (+ 1.63 ppm).

(–)-(2*R*,3*S*,4*S*)-4-(benzyloxy)-5-((tert-butyldiphenylsilyl)oxy)-2-formyl-2-methylpentane-1,3-diyl dibenzoate (**38**). To a stirred solution of alkene **37** (148 mg, 1.00 equiv, 0.208 mmol) in CH_2_Cl_2_ (22 mL, 0.010 M) at −78 °C, O_3_ was added and bubbled under vacuum until the reaction mixture turned blue (about 30 min). The solution was then purged with nitrogen to remove excess ozone. Following the addition of NEt_3_ (0.029 mL, 1.0 equiv, 0.21 mmol), the reaction was stirred for 1 h while warming to room temperature. After filtering over MgSO_4_, the mixture was concentrated under reduced pressure. Purification by flash chromatography (Hexanes/EtOAc) provided aldehyde **38** (113 mg, 76% yield). R_f_ = 0.86 (Hexanes/EtOAc, 50:50); [α]_D_^25^ −20 (*c* 5.6, CH_2_Cl_2_); formula: C_44_H_46_O_7_Si; MW: 714.9300 g/mol; IR (neat) ν_max_ 2931, 2857, 1721, 1263, 1106 cm^−1^; ^1^H NMR (500 MHz, CDCl_3_) δ 10.02 (s, 1H), 8.11 (d, *J* = 7.6 Hz, 2H), 8.01 (d, *J* = 7.6 Hz, 2H), 7.73–7.67 (m, 2H), 7.65–7.61 (m, 1H), 7.61–7.54 (m, 2H), 7.51–7.46 (m, 4H), 7.44–7.40 (m, 4H), 7.39–7.27 (m, 6H), 7.25–7.22 (m, 2H), 5.93 (d, *J* = 1.6 Hz, 1H), 4.56 (d, *J* = 11.3 Hz, 1H), 4.46 (appt, *J* = 11.8 Hz, 2H), 4.37 (d, *J* = 11.3 Hz, 1H), 3.86–3.83 (m, 1H), 3.81 (dd, *J* = 10.2, 5.2 Hz, 1H), 3.68 (dd, *J* = 9.9, 7.9 Hz, 1H), 1.31 (s, 3H), 1.08 (s, 9H) ppm; ^13^C NMR (126 MHz, CDCl_3_) δ 200.6, 166.1, 165.5, 137.1, 135.68 (2C), 135.67 (2C), 133.5, 133.2, 133.1, 132.8, 130.2 (2C), 130.0, 129.9, 129.8 (2C), 129.7, 129.5, 128.7 (2C), 128.49 (2C), 128.42 (2C), 128.4 (2C), 128.1, 128.0 (2C), 127.8 (2C), 77.4, 75.1, 72.6, 65.7, 61.4, 52.9, 26.9 (3C), 19.2, 15.2 ppm; HRMS (ESI) *m*/*z*: calcd for C_44_H_47_O_7_Si [M+H]^+^ 715.3086, found 715.3076 (–1.40 ppm).

((3*R*,4*S*,5*S*)-4-(benzoyloxy)-5-(((tert-butyldiphenylsilyl)oxy)methyl)-2-(tert-butylthio)-3-methyltetrahydrofuran-3-yl)methyl benzoate (**41a,b**). To a stirred solution of aldehyde **38** (31 mg, 1.0 equiv, 0.04 mmol) in CH_2_Cl_2_ (0.43 mL, 0.10 M) at −60 °C, *tert*-butylthiol (19 μL, 4.0 equiv, 0.17 mmol) and boron trifluoride diethyletherate (7 μL, 1.3 equiv, 0.06 mmol) were added. The reaction mixture was stirred at −60 °C for 2 h followed by the addition of NEt_3_ (24 μL, 4.0 equiv, 0.17 mmol) with additional stirring for 15 min at −60 °C. A saturated solution of NaHCO_3_ was added and the crude mixture was warmed to room temperature. The aqueous layer was extracted (3×) with CH_2_Cl_2_ and the combined organic layers were washed with brine, dried over MgSO_4_, filtered, and concentrated under reduced pressure. Purification by flash chromatography (Hexanes/EtOAc) provided **41a,b** (27 mg, 91% yield) as a 5:1 mixture in favor of the α-L-anomer. R_f_ = 0.89 (Hexanes/EtOAc, 50:50); formula: C_41_H_48_O_6_SSi; MW: 696.9740 g/mol; IR (neat) ν_max_ 2958, 2930, 2858, 1722, 1266, 1108 cm^−1^; ^1^H NMR (500 MHz, CDCl_3_) δ 8.12–8.08 (m, 2H), 8.02–7.99 (m, 2H), 7.98–7.93 (m, 4H), 7.62–7.60 (m, 3H), 7.58–7.51 (m, 9H), 7.45–7.37 (m, 10H), 7.35 (d, *J* = 7.6 Hz, 2H), 7.34–7.27 (m, 4H), 7.14 (t, *J* = 7.7 Hz, 4H), 5.87 (d, *J* = 4.3 Hz, 1H, major), 5.85 (d, *J* = 4.9 Hz, 1H, minor), 5.41 (s, 1H, major), 5.21 (s, 1H, minor), 4.65 (ddd, *J* = 7.8, 5.9, 4.2 Hz, 1H, major), 4.55–4.50 (m, 1H, minor), 4.44 (d, *J* = 11.2 Hz, 1H, major), 4.41–4.37 (m, 2H, minor), 4.30 (d, *J* = 11.2 Hz, 1H, major), 4.01–3.96 (m, 1H, minor), 3.82–3.78 (m, 3H, 2× major and 1× minor), 1.47 (s, 3H, minor), 1.38 (s, 12H, major), 1.35 (s, 9H, minor), 0.92 (s, 18H, major and minor) ppm; ^13^C NMR (126 MHz, CDCl_3_) δ 166.4 (major), 165.5 (minor), 165.0 (minor), 165.0 (major), 135.73 (2C), 135.69 (2C), 135.56 (2C), 135.54 (2C), 133.32, 133.30, 133.24, 133.15, 133.11, 133.07, 133.04, 130.2, 130.1, 129.9 (2C), 129.81 (4C), 129.79 (2C), 129.76, 129.7, 129.63, 129.57 (2C), 128.61 (2C), 128.59 (2C), 128.5 (2C), 128.4 (2C), 127.82 (2C), 127.77 (2C), 127.65 (2C), 127.60 (2C), 90.3 (minor), 86.6 (major), 81.6 (minor), 80.1 (major), 78.2 (major), 77.0 (minor), 65.7 (major), 65.6 (minor), 62.4 (minor), 61.6 (major), 51.3 (minor), 50.9 (major), 43.7 (minor), 43.6 (major), 31.8 (major, 3C), 31.7 (minor, 3C), 26.9 (minor, 3C), 26.7 (major, 3C), 22.4 (minor), 19.11 (minor), 19.09 (major), 17.6 (major) ppm, due to overlapping carbon signals in the aromatic region, 2 peaks are hidden; HRMS (ESI) *m*/*z*: calcd for C_41_H_49_O_6_SSi [M+H]^+^ 697.3014, found 697.3009 (−0.72 ppm).

(–)-(2*R*,3*S*,4*S*)-4-(benzyloxy)-2-(bis(benzylthio)methyl)-5-((tert-butyldiphenylsilyl)oxy)-2-methylpentane-1,3-diyl dibenzoate (**40**). To a stirred solution of aldehyde **38** (40 mg, 1.0 equiv, 0.056 mmol) in CH_2_Cl_2_ (0.56 mL, 0.10 M) at −60 °C, benzyl mercaptan (27 μL, 4.0 equiv, 0.22 mmol) and boron trifluoride diethyletherate (9.0 μL, 1.3 equiv, 0.073 mmol) were added. The reaction mixture was stirred at −60 °C for 2 h, followed by the addition of NEt_3_ (31 μL, 4.0 equiv, 0.22 mmol) with an additional 15 min of stirring at −60 °C. A saturated solution of NaHCO_3_ was added and the crude was warmed to room temperature. The aqueous layer was extracted (3×) with CH_2_Cl_2_ and the combined organic layers were washed with brine, dried over MgSO_4_, filtered, and concentrated under reduced pressure. Purification by flash chromatography (Hexanes/EtOAc) provided dithioacetal **40** (46 mg, 87% yield). R_f_ = 0.57 (Hexanes/EtOAc, 80:20); [α]_D_^25^ −11 (*c* 4.6, CH_2_Cl_2_); formula: C_58_H_60_O_6_S_2_Si; MW: 945.3170 g/mol; IR (neat) ν_max_ 3065, 2930, 2857, 1721, 1494, 1266, 1176, 1108 cm^−1^; ^1^H NMR (500 MHz, CDCl_3_) δ 8.06 (dd, *J* = 8.3, 1.2 Hz, 2H), 7.93 (dd, *J* = 8.3, 1.2 Hz, 2H), 7.64 (dd, *J* = 8.0, 1.3 Hz, 2H), 7.60–7.51 (m, 4H), 7.44 (t, *J* = 7.8 Hz, 2H), 7.36 (t, *J* = 7.9 Hz, 3H), 7.30 (t, *J* = 7.4 Hz, 3H), 7.26–7.17 (m, 14H), 7.16–7.14 (m, 3H), 6.07 (d, *J* = 2.0 Hz, 1H), 4.70 (d, *J* = 11.8 Hz, 1H), 4.61 (d, *J* = 11.8 Hz, 1H), 4.48 (d, *J* = 11.7 Hz, 1H), 4.35 (d, *J* = 11.7 Hz, 1H), 3.96 (td, *J* = 6.4, 1.8 Hz, 1H), 3.84 (s, 1H), 3.80 (d, *J* = 12.9 Hz, 1H), 3.77 (dd, *J* = 10.6, 5.8 Hz, 1H), 3.73 (t, *J* = 12.6 Hz, 2H), 3.68 (d, *J* = 12.7 Hz, 1H), 3.61 (dd, *J* = 10.6, 6.6 Hz, 1H), 1.34 (s, 3H), 1.02 (s, 9H) ppm; ^13^C NMR (126 MHz, CDCl_3_) δ 166.3, 165.6, 138.4, 137.63, 137.56, 135.8 (2C), 135.7 (2C), 133.4, 133.2, 133.1, 133.0, 130.21, 130.20 (2C), 130.18, 129.9 (2C), 129.73, 129.67, 129.4 (4C), 128.63 (2C), 128.56 (2C), 128.51 (2C), 128.47 (2C), 128.3 (2C), 127.8 (2C), 127.7 (2C), 127.4 (3C), 127.3, 127.2, 78.7, 74.5, 72.2, 67.8, 63.2, 58.6, 48.6, 37.5, 37.4, 26.9 (3C), 19.7, 19.2 ppm; HRMS (ESI) *m*/*z*: calcd for C_58_H_60_NaO_6_S_2_Si [M+Na]^+^ 967.3493, found 967.3483 (–1.03 ppm).

(+)-(2*R*,3*S*,4*S*)-2-(bis(benzylthio)methyl)-5-((tert-butyldiphenylsilyl)oxy)-4-hydroxy-2-methylpentane-1,3-diyl dibenzoate (**43**). To a stirred solution of C4-protected dithioacetal **40** (38 mg, 1.0 equiv, 0.040 mmol) in CH_2_Cl_2_ (0.40 mL, 0.10 M) at −78 °C, boron trichloride (1.0 M in DCM, 52 μL, 1.30 equiv, 0.052 mmol) was added. The reaction mixture was stirred at −78 °C for 1 h followed by the addition of boron trichloride (1.30 equiv). After stirring for another hour at −78 °C, a third addition of boron trichloride (1.30 equiv) was carried out. After one additional hour at −78 °C, the reaction was quenched by the addition of methanol, warmed to room temperature, and was then concentrated under reduced pressure. The aqueous layer was extracted (3×) with CH_2_Cl_2_ and the combined organic layers were washed with water, dried over MgSO_4_, filtered, and concentrated under reduced pressure. Purification by flash chromatography (Hexanes/EtOAc) provided dithioacetal **43** (5 mg, 15% yield). R_f_ = 0.45 (Hexanes/EtOAc, 80:20); [α]_D_^25^ +15 (*c* 0.3, CH_2_Cl_2_); formula: C_51_H_54_O_6_S_2_Si; MW: 855.1920 g/mol; IR (neat) ν_max_ 3505, 3066, 2929, 2857, 1722, 1268, 1177 cm^−1^; ^1^H NMR (500 MHz, CDCl_3_) δ 8.01 (dd, *J* = 8.3, 1.2 Hz, 2H), 7.92 (dd, *J* = 8.3, 1.2 Hz, 2H), 7.58–7.52 (m, 6H), 7.41 (dt, *J* = 10.1, 7.9 Hz, 5H), 7.32 (t, *J* = 7.4 Hz, 2H), 7.25–7.21 (m, 8H), 7.19–7.14 (m, 5H), 5.77 (d, *J* = 0.8 Hz, 1H), 4.71 (d, *J* = 11.6 Hz, 1H), 4.60 (d, *J* = 11.6 Hz, 1H), 4.00–3.95 (m, 1H), 3.94 (s, 1H), 3.80 (d, *J* = 13.0 Hz, 1H), 3.74 (t, *J* = 12.3 Hz, 2H), 3.71 (d, *J* = 12.7 Hz, 1H), 3.56 (dd, *J* = 10.2, 5.3 Hz, 1H), 3.52 (dd, *J* = 10.2, 7.0 Hz, 1H), 2.52 (d, *J* = 5.7 Hz, 1H), 1.32 (s, 3H), 0.96 (s, 9H) ppm; ^13^C NMR (126 MHz, CDCl_3_) δ 166.3, 165.6, 137.53, 137.51, 135.68 (2C), 135.65 (2C), 133.3, 133.1, 133.00, 132.98, 130.21, 130.17 (2C), 129.9 (2C), 129.83, 129.81, 129.3 (4C), 128.66 (2C), 128.64 (2C), 128.57 (2C), 128.55 (2C), 127.8 (5C), 127.4, 127.3, 74.4, 70.0, 67.4, 65.9, 57.7, 48.4, 37.5, 37.3, 26.9 (3C), 19.31, 19.25 ppm; HRMS (ESI) *m*/*z*: calcd for C_51_H_54_NaO_6_S_2_Si [M+Na]^+^ 877.3023, found 877.3014 (–1.03 ppm).

((3*R*,4*S*,5*S*)-4-(benzoyloxy)-5-(((tert-butyldiphenylsilyl)oxy)methyl)-2-hydroxy-3-methyltetrahydrofuran-3-yl)methyl benzoate (**42a,b**). To a stirred solution of aldehyde **38** (50 mg, 1.0 equiv, 0.070 mmol) in THF:*i*PrOH (3:1, 3.5 mL, 0.020 M) at room temperature, palladium (10 wt.%) on activated carbon (30 mg, 0.40 equiv, 0.028 mmol) was added. The reaction mixture was degassed and flushed using a hydrogen-filled balloon. After stirring for 16 h, the reaction mixture was filtered through Celite^®^, washed with MeOH, and concentrated under reduced pressure. Purification by flash chromatography (Hexanes/EtOAc) provided a mixture of lactols **42a,b** (36 mg, 82% yield) as a 3:1 mixture in favor of the β-L-anomer. R_f_ = 0.80 (Hexanes/EtOAc, 50:50); formula: C_37_H_40_O_7_Si; MW: 624.8050 g/mol; ^1^H NMR (500 MHz, CDCl_3_) δ 7.99 (ddd, *J* = 7.1, 3.7, 2.1 Hz, 4H), 7.98–7.92 (m, 4H), 7.63–7.57 (m, 3H), 7.56–7.51 (m, 6H), 7.50–7.47 (m, 3H), 7.40 (dtd, *J* = 16.8, 8.8, 5.7 Hz, 8H), 7.34–7.30 (m, 8H), 7.19 (dt, *J* = 15.2, 7.6 Hz, 4H), 5.81 (d, *J* = 4.9 Hz, 1H, minor), 5.78 (d, *J* = 5.4 Hz, 1H, major), 5.52 (s, 1H, minor), 5.20 (s, 1H, major), 4.67 (q, *J* = 5.9 Hz, 1H, minor), 4.64–4.61 (m, 1H, major), 4.60 (d, *J* = 11.2 Hz, 1H, major), 4.49 (d, *J* = 11.2 Hz, 1H, major), 4.45 (d, *J* = 11.2 Hz, 1H, minor), 4.36 (d, *J* = 11.1 Hz, 1H, minor), 3.93 (dd, *J* = 10.5, 7.1 Hz, 1H, major), 3.84 (dd, *J* = 10.4, 7.0 Hz, 1H, minor), 3.80 (dt, *J* = 10.1, 4.8 Hz, 2H, major and minor), 1.39 (s, 3H, minor), 1.32 (s, 3H, major), 0.95 (s, 9H, minor), 0.94 (s, 9H, major) ppm, labile protons were not observed due to exchange; ^13^C NMR (126 MHz, CDCl_3_) δ 166.5 (major), 166.4 (minor), 165.34 (minor), 165.31 (major), 135.8 (2C), 135.70 (2C), 135.64 (2C), 135.60 (2C), 133.5, 133.4, 133.2, 133.14, 133.13, 133.11, 133.0, 132.9, 130.03, 129.95 (2C), 129.89, 129.85, 129.82, 129.75 (2C), 129.74, 129.70 (2C), 129.61 (2C), 129.57, 128.7 (2C), 128.6 (2C), 128.51 (2C), 128.48 (2C), 127.8 (2C), 127.79 (2C), 127.72 (2C), 127.68 (2C), 103.2 (major), 100.9 (minor), 81.8 (major), 79.1 (minor), 78.8 (minor), 77.1 (major), 66.2 (minor), 64.5 (major), 62.7 (major), 62.0 (minor), 51.2 (major), 50.5 (minor), 26.79 (major, 3C), 26.75 (minor, 3C), 20.0 (major), 19.14 (minor), 19.09 (major), 16.0 (minor) ppm, due to overlapping carbon signals in the aromatic region 2 peaks are hidden; HRMS (ESI) *m*/*z*: calcd for C_37_H_40_NaO_7_Si [M+Na]^+^ 647.2435, found 647.2427 (–1.24 ppm).

((3*R*,4*S*,5*S*)-4-(benzoyloxy)-2-(benzylthio)-5-(((tert-butyldiphenylsilyl)oxy)methyl)-3-methyltetrahydrofuran-3-yl)methyl benzoate (**44a,b**). To a stirred solution of lactols **42a,b** (36 mg, 1.0 equiv, 0.058 mmol) in CH_2_Cl_2_ (0.50 mL, 0.11 M) at −60 °C, benzyl mercaptan (27 μL, 4.0 equiv, 0.23 mmol) and boron trifluoride diethyletherate (18 μL, 2.5 equiv, 0.14 mmol) were added. The reaction mixture was stirred at −40 °C for 2 h. The reaction was quenched by the addition of triethylamine (32 μL, 4.0 equiv, 0.23 mmol) at −60 °C, followed by stirring for 15 min. After the addition of a saturated solution of NaHCO_3_, the crude was warmed to room temperature. The aqueous layer was extracted (3×) with CH_2_Cl_2_ and the combined organic layers were washed with brine, dried over MgSO_4_, filtered, and concentrated under reduced pressure. Purification by flash chromatography (Hexanes/EtOAc) provided **44a,b** (35 mg, 83% yield) as a 2:1 mixture in favor of the α-L-anomer. R_f_ = 0.47 (Hexanes/EtOAc, 80:20); formula: C_44_H_46_O_6_SSi; MW: 730.9910 g/mol; IR (neat) ν_max_ 3069, 2931, 2857, 1724, 1270 cm^−1^; ^1^H NMR (500 MHz, CDCl_3_) δ 8.12 (d, *J* = 7.1 Hz, 2H), 7.92 (d, *J* = 8.4 Hz, 2H), 7.89 (d, *J* = 7.1 Hz, 4H), 7.67–7.63 (m, 6H), 7.59–7.48 (m, 7H), 7.46–7.40 (m, 6H), 7.37 (t, *J* = 5.7 Hz, 6H), 7.32 (d, *J* = 6.1 Hz, 5H), 7.29–7.21 (m, 6H), 7.18 (t, *J* = 7.9 Hz, 6H), 5.87 (d, *J* = 4.9 Hz, 1H, minor), 5.79 (d, *J* = 4.4 Hz, 1H, major), 5.25 (s, 1H, major), 4.97 (s, 1H, minor), 4.69–4.59 (m, 2H, major and minor), 4.38 (d, *J* = 3.7 Hz, 2H, minor), 4.35 (d, *J* = 11.0 Hz, 1H, major), 4.17 (d, *J* = 11.2 Hz, 1H, major), 4.09–4.01 (m, 1H, minor), 3.89 (s, 2H), 3.84 (appd, *J* = 8.2 Hz, 5H), 1.38 (s, 3H, major), 1.36 (s, 3H, minor), 0.97 (s, 9H, major), 0.94 (s, 9H, minor) ppm; ^13^C NMR (126 MHz, CDCl_3_) δ 166.25 (major), 166.24 (minor), 165.1 (major), 165.0 (minor), 138.0, 137.7, 135.739 (2C), 135.733 (2C), 135.62 (2C), 135.56 (2C), 133.4, 133.34, 133.33, 133.25, 133.22, 133.21, 133.17, 133.069, 133.066, 130.2, 129.9, 129.83 (4C), 129.82 (4C), 129.79 (2C), 129.7, 129.62, 129.61, 129.552, 129.548, 129.225, 129.214 (2C), 128.7 (2C), 128.63 (2C), 128.58 (4C), 128.5 (2C), 128.4 (2C), 127.8 (2C), 127.7 (2C), 127.6 (2C), 127.3 (2C), 127.1, 91.0 (minor), 88.8 (major), 82.1 (minor), 79.7 (major), 79.0 (major), 76.7 (minor), 66.1 (major), 64.8 (minor), 62.6 (minor), 61.6 (major), 51.5 (minor), 50.8 (major), 35.7 (major), 35.4 (minor), 26.8 (major, 3C), 26.7 (minor, 3C), 22.3 (minor), 19.2 (major), 19.1 (minor), 18.0 (major) ppm; HRMS (ESI) *m*/*z*: calcd for C_44_H_46_NaO_6_SSi [M+Na]^+^ 753.2676, found 753.2662 (–1.86 ppm).

(+)-(2*R*,3*S*,4*S*)-2-(bis(benzylthio)methyl)-5-((tert-butyldiphenylsilyl)oxy)-4-hydroxy-2-methylpentane-1,3-diyl dibenzoate (**43**). To a stirred solution of lactols **42a,b** (273 mg, 1.00 equiv, 0.437 mmol) in CH_2_Cl_2_ (4.4 mL, 0.10 M) at −40 °C, benzyl mercaptan (0.25 mL, 4.8 equiv, 2.1 mmol) and TiCl_4_ (1.2 mL, 2.6 equiv, 1.2 mmol, 1M DCM) were added. The reaction mixture was stirred at −20 °C for 4 h. The reaction was quenched by the addition of triethylamine (0.3 mL, 4.8 equiv, 2.1 mmol) at −60 °C followed by stirring for 15 min. After the addition of a saturated solution of NaHCO_3_, the mixture was warmed to room temperature. The aqueous layer was extracted (3×) with CH_2_Cl_2_ and the combined organic layers were washed with brine, dried over MgSO_4_, filtered, and concentrated under reduced pressure. Purification by flash chromatography (Hexanes/EtOAc) provided **43** (299 mg, 80% yield), which corresponds to the same product obtained from C4-OBn deprotection of dithioacetal **40,** as reported above.

(2*R*,3*S*,4*S*)-2-(bis(benzylthio)methyl)-5-((tert-butyldiphenylsilyl)oxy)-2-methyl-4-((methylsulfonyl)oxy)pentane-1,3-diyl dibenzoate (**45**). To a stirred solution of dithioacetal **43** (434 mg, 1.00 equiv, 0.507 mmol) in pyridine (8.7 mL, 0.06 M) at 0 °C, methanesulfonyl chloride (80 μL, 2.0 equiv, 1.0 mmol) was added. The reaction mixture was stirred at room temperature for 3 h. The reaction was concentrated and then diluted with CH_2_Cl_2_. The aqueous layer was extracted (3×) with CH_2_Cl_2_ and the combined organic layers were washed with HCl (0.1N), a saturated solution of NaHCO_3_, and with brine, dried over MgSO_4_, filtered, and concentrated under reduced pressure. C4-mesylated dithioacetal **45** was used without further purification. ^1^H NMR (500 MHz, CDCl_3_) δ 8.01 (dd, *J* = 8.3, 1.3 Hz, 2H), 7.94 (dd, *J* = 8.3, 1.2 Hz, 2H), 7.64–7.51 (m, 7H), 7.45 (t, *J* = 7.8 Hz, 2H), 7.40–7.28 (m, 7H), 7.20 (dd, *J* = 10.6, 4.9 Hz, 4H), 7.17–7.13 (m, 3H), 7.12 (d, *J* = 4.4 Hz, 3H), 6.01 (d, *J* = 3.2 Hz, 1H), 5.30–5.26 (m, 1H), 4.68 (d, *J* = 12.0 Hz, 1H), 4.39 (d, *J* = 11.9 Hz, 1H), 4.00 (dd, *J* = 11.2, 7.0 Hz, 1H), 3.89 (s, 1H), 3.79 (d, *J* = 12.8 Hz, 1H), 3.73 (dd, *J* = 11.3, 5.4 Hz, 1H), 3.71 (d, *J* = 12.8 Hz, 1H), 3.62 (s, 2H), 2.96 (s, 3H), 1.39 (s, 3H), 1.00 (s, 9H) ppm.

Preparation of silylated thymine. To a suspension of thymine (0.80 g, 1.0 equiv., 6.4 mmol) in HMDS (4.0 mL, 3.0 equiv., 19 mmol) under inert atmosphere, (NH_4_)_2_SO_4_ (18 mg, 0.022 equiv., 0.14 mmol) was added. The reaction mixture was refluxed until a clear solution was obtained (3 h). Upon cooling to room temperature, the solution was placed under high vacuum for approximately 1 h to remove excess HMDS. A 0.78 M solution of the silylated nucleobase was made in CH_2_Cl_2_.

(+)-(2*R*,3*S*,4*S*)-2-((*R*)-(benzylthio)(5-methyl-2,4-dioxo-3,4-dihydropyrimidin-1(2H)-yl)methyl)-5-((tert-butyldiphenylsilyl)oxy)-2-methyl-4-((methylsulfonyl)oxy)pentane-1,3-diyl dibenzoate (**46a**) and (–)-(2*R*,3*S*,4*R*)-2-((*S*)-(benzylthio)(5-methyl-2,4-dioxo-3,4-dihydropyrimidin-1(2H)-yl) methyl)-5-((tert-butyldiphenylsilyl)oxy)-2-methyl-4-((methylsulfonyl)oxy)pentane-1,3-diyl dibenzoate (**46b**). To a stirred solution of crude C4-Ms dithioacetal **45** (200 mg, 1.00 equiv, 0.214 mmol) in anhydrous CH_2_Cl_2_ (1.1 mL, 0.20 M), silylated thymine (0.82 mL, 3.0 equiv, 0.64 mmol, 0.78 M in CH_2_Cl_2_) was added. The resulting solution was cooled to 0 °C and iodide (114 mg, 2.10 equiv, 0.450 mmol) was added, followed by stirring at room temperature for 3 h. After cooling to 0 °C, the reaction was quenched by theaddition of a saturated solution of Na_2_S_2_O_3_ and dissolved in EtOAc. The aqueous layer was extracted (3×) with EtOAc and the combined organic layers were washed with brine, dried over MgSO_4_, filtered, and concentrated under reduced pressure. ^1^H NMR analysis of the crude mixture indicated a 1.1:1 ratio of thioaminals. Purification by flash chromatography (Hexanes/EtOAc) provided thioaminals **46a,b** (154 mg, 77% yield over 2 steps). A pure fraction of each isomer was obtained for characterization. **46a (1′,2′-*anti*)**: R_f_ = 0.17 (Hexanes/EtOAc, 60:40); [α]_D_^25^ +53 (*c* 0.9, CH_2_Cl_2_); formula: C_50_H_54_N_2_O_10_S_2_Si; MW: 935.1910 g/mol; IR (neat) ν_max_ 3182, 3032, 2931, 2857, 1724, 1683, 1264, 1105 cm^−1^; ^1^H NMR (500 MHz, CDCl_3_) δ 8.09 (dd, *J* = 8.3, 1.2 Hz, 2H), 7.69 (ddd, *J* = 7.8, 6.4, 1.4 Hz, 4H), 7.62 (ddd, *J* = 16.1, 5.5, 4.3 Hz, 2H), 7.54 (dd, *J* = 8.0, 1.3 Hz, 2H), 7.47 (ddd, *J* = 14.3, 7.5, 2.2 Hz, 4H), 7.41 (dt, *J* = 13.7, 6.7 Hz, 3H), 7.30 (dd, *J* = 9.5, 6.2 Hz, 3H), 7.21–7.14 (m, 7H), 6.22 (s, 1H), 6.14 (d, *J* = 1.9 Hz, 1H), 5.45–5.40 (m, 1H), 4.69 (s, 2H), 4.05 (dd, *J* = 10.5, 5.3 Hz, 1H), 3.82–3.76 (m, 2H), 3.71 (dd, *J* = 10.5, 8.3 Hz, 1H), 3.30 (s, 3H), 1.69 (s, 3H), 1.26 (s, 3H), 1.08 (s, 9H) ppm; ^13^C NMR (126 MHz, CDCl_3_) δ 165.9, 165.3, 162.4, 151.2, 137.8, 136.5, 135.7 (2C), 135.6 (2C), 133.9, 133.5, 132.6, 132.5, 130.3 (2C), 130.1, 129.9, 129.25, 129.24 (2C), 129.0, 128.9 (2C), 128.8 (2C), 128.64 (2C), 128.58(2C), 128.0 (2C), 127.8 (2C), 127.6, 110.2, 77.5, 73.3, 66.6, 66.0, 62.3, 48.5, 39.4, 37.1, 26.8 (3C), 19.3, 16.2, 12.7 ppm; HRMS (ESI) *m*/*z*: calcd for C_50_H_55_N_2_O_10_S_2_Si [M+H]^+^ 935.3062, found 935.3049 (–1.39 ppm). **46b (1′,2′-*syn*)**: R_f_ = 0.77 (Hexanes/EtOAc, 30:70); [α]_D_^25^ −8 (*c* 0.3, CH_2_Cl_2_); formula: C_50_H_54_N_2_O_10_S_2_Si; MW: 934.1910 g/mol; IR (neat) ν_max_ 2955, 2922, 2852, 1726, 1686, 1261, 1107 cm^−1^; ^1^H NMR (500 MHz, CDCl_3_) δ 8.05 (d, *J* = 7.1 Hz, 2H), 7.96 (d, *J* = 7.1 Hz, 2H), 7.68 (s, 1H), 7.62 (t, *J* = 7.4 Hz, 1H), 7.55 (d, *J* = 6.6 Hz, 2H), 7.50 (q, *J* = 7.1 Hz, 4H), 7.39–7.34 (m, 4H), 7.32–7.28 (m, 3H), 7.27 (d, *J* = 3.4 Hz, 1H), 7.18 (t, *J* = 7.5 Hz, 2H), 6.99 (dt, *J* = 15.1, 7.4 Hz, 4H), 6.91 (t, *J* = 7.2 Hz, 1H), 6.13 (s, 1H), 5.80 (d, *J* = 2.9 Hz, 1H), 5.21 (ddd, *J* = 7.8, 5.0, 3.1 Hz, 1H), 4.56 (d, *J* = 12.8 Hz, 1H), 4.10 (d, *J* = 12.9 Hz, 1H), 4.01 (dd, *J* = 11.4, 7.6 Hz, 1H), 3.75 (dd, *J* = 11.4, 5.0 Hz, 1H), 3.53 (d, *J* = 14.2 Hz, 1H), 3.49 (d, *J* = 14.2 Hz, 1H), 2.95 (s, 3H), 1.50 (s, 3H), 1.33 (s, 3H), 0.96 (s, 9H) ppm; ^13^C NMR (126 MHz, CDCl_3_) δ 165.6, 165.2, 162.6, 151.4, 138.0, 136.5, 135.54 (2C), 135.50 (2C), 133.8, 133.5, 132.5, 132.4, 130.12 (2C), 130.09, 130.05 (3C), 129.2, 129.1, 128.8 (2C), 128.7 (2C), 128.6 (2C), 128.4 (2C), 128.0 (2C), 127.9 (2C), 127.3, 111.0, 79.7, 70.9, 65.5, 65.2, 64.2, 47.8, 39.5, 37.1, 26.9 (3C), 19.3, 15.1, 12.4 ppm; HRMS (ESI) *m*/*z*: calcd for C_50_H_54_N_2_NaO_10_S_2_Si [M+Na]^+^ 957.2881, found 957.2884 (+0.31 ppm).

(–)-(2*R*,3*S*,4*R*)-2-((*S*)-(benzylthio)(5-methyl-2,4-dioxo-3,4-dihydropyrimidin-1(2H)-yl)methyl)-5-((tert-butyldiphenylsilyl)oxy)-4-hydroxy-2-methylpentane-1,3-diyl dibenzoate (**48**). To a 1.1:1 mixture of thioaminals **46a,b** (266 mg, 1.00 equiv, 0.284 mmol) in a high-pressure flask, anhydrous 2,6-lutidine (2.9 mL, 0.10 M), and sodium iodide (426 mg, 10.0 equiv, 2.84 mmol) were added. The reaction mixture was stirred at 160 °C for 16 h in a sand bath. After cooling to room temperature, the volatiles were removed under reduced pressure. ^1^H NMR analysis of the crude mixture indicated a 7:1 ratio of β:α thiofuranosides **47a,b,** along with unreacted 1′,2′-*syn* thioaminal **46b** and side-product **48**. Purification by flash chromatography (Hexanes/EtOAc) provided thiofuranoside **47a** (106 mg, 49% yield), **47b** (8 mg, 5%), 1′,2′-*syn* thioaminal **46b** (94 mg, 35%) and side-product **48** (14 mg, 6%). Thiofuranosides **47a,b** corresponded to those characterized below for thymine addition onto the cyclic thiofuranoside **62**. **48**: R_f_ = 0.84 (Hexanes/EtOAc, 30:70); [α]_D_^25^ −27 (*c* 3.3, CH_2_Cl_2_); formula: C_49_H_52_N_2_O_8_SSi; MW: 857.1060 g/mol; IR (neat) ν_max_ 3069, 2930, 2857, 1679, 1260, 1105 cm^−1^; ^1^H NMR (500 MHz, CDCl_3_) 8.17 (s, 1H), 8.11 (dd, *J* = 8.4, 1.3 Hz, 2H), 7.93 (dd, *J* = 8.4, 1.3 Hz, 2H), 7.62–7.57 (m, 5H), 7.50 (dd, *J* = 8.1, 1.4 Hz, 2H), 7.47–7.43 (m, 2H), 7.43–7.35 (m, 5H), 7.30 (t, *J* = 7.1 Hz, 2H), 7.15–7.11 (m, 4H), 7.09 (d, *J* = 8.2 Hz, 2H), 6.34 (s, 1H), 5.63 (d, *J* = 8.5 Hz, 1H), 4.71 (d, *J* = 12.0 Hz, 1H), 4.50 (d, *J* = 12.1 Hz, 1H), 4.11–4.00 (m, 1H), 3.88 (d, *J* = 8.3 Hz, 1H), 3.70–3.66 (m, 1H), 3.66–3.60 (m, 2H), 3.50 (d, *J* = 14.0 Hz, 1H), 1.71 (s, 3H), 1.20 (s, 3H), 1.00 (s, 9H) ppm; ^13^C NMR (126 MHz, CDCl_3_) δ 166.2, 165.4, 162.8, 151.9, 138.4, 136.8, 135.7 (2C), 135.6 (2C), 133.5, 133.4, 132.9, 132.7, 130.0 (2C), 129.93 (2C), 129.85, 129.83, 129.7, 129.5, 128.68 (2C), 128.66 (2C), 128.6 (2C), 128.5 (2C), 127.8 (2C), 127.7 (2C), 127.4, 110.9, 72.5, 71.8, 66.8, 66.0, 65.1, 47.9, 37.6, 26.9 (3C), 19.2, 16.0, 12.7 ppm; HRMS (ESI) *m*/*z*: calcd for C_49_H_52_N_2_NaO_8_SSi [M+Na]^+^ 879.3106, found 879.3111 (+0.57 ppm).

(–)-(2*R*,3*S*,4*R*)-2-((*S*)-(benzylthio)(5-methyl-2,4-dioxo-3,4-dihydropyrimidin-1(2H)-yl)methyl)-5-((tert-butyldiphenylsilyl)oxy)-2-methyl-4-((methylsulfonyl)oxy)pentane-1,3-diyl dibenzoate (**49**). To a stirred solution of alcohol **48** (20 mg, 1.0 equiv, 0.023 mmol) in pyridine (0.4 mL, 0.06 M) at 0 °C, MsCl (4 μL, 2 equiv, 0.05 mmol) was added dropwise. The reaction mixture was stirred at room temperature for 3 h. After completion of the reaction, the mixture was concentrated under reduced pressure, diluted in CH_2_Cl_2_, washed with a solution of HCl (0.1N), a saturated solution of NaHCO_3_, and with brine, dried over MgSO_4_, filtered, and concentrated under reduced pressure. Purification by flash chromatography (Hexanes/EtOAc) provided the pure product **49** (13 mg, 60% yield). R_f_ = 0.55 (Hexanes/EtOAc, 30:70); [α]_D_^25^ −18 (*c* 1.0, CH_2_Cl_2_); formula: C_50_H_54_N_2_O_10_S_2_Si; MW: 935.1910 g/mol; IR (neat) ν_max_ 3177, 3070, 2932, 2857, 1726, 1688, 1261, 1105 cm^−1^; ^1^H NMR (500 MHz, CDCl_3_) δ 8.03 (dd, *J* = 7.3, 1.1 Hz, 2H), 7.94 (dd, *J* = 7.3, 1.2 Hz, 2H), 7.76 (s, 1H), 7.59 (dd, *J* = 8.0, 1.2 Hz, 4H), 7.53 (dd, *J* = 6.8, 1.2 Hz, 2H), 7.48–7.42 (m, 4H), 7.39 (dd, *J* = 14.1, 6.9 Hz, 1H), 7.33 (dd, *J* = 14.8, 7.3 Hz, 3H), 7.28 (d, *J* = 7.4 Hz, 1H), 7.25 (d, *J* = 8.2 Hz, 2H), 6.98 (d, *J* = 4.6 Hz, 4H), 6.94–6.89 (m, 1H), 6.10 (s, 1H), 5.94 (d, *J* = 2.6 Hz, 1H), 5.20 (dt, *J* = 7.6, 2.8 Hz, 1H), 4.40 (d, *J* = 12.6 Hz, 1H), 4.16 (d, *J* = 12.6 Hz, 1H), 3.95 (dd, *J* = 12.0, 3.2 Hz, 1H), 3.83 (dd, *J* = 12.0, 7.6 Hz, 1H), 3.50 (d, *J* = 14.2 Hz, 1H), 3.41 (d, *J* = 14.2 Hz, 1H), 2.98 (s, 3H), 1.45 (s, 3H), 1.13 (s, 3H), 0.99 (s, 9H) ppm; ^13^C NMR (126 MHz, CDCl_3_) δ 165.7, 164.9, 162.5, 151.4, 137.8, 136.5, 135.7 (2C), 135.6 (2C), 133.8, 133.6, 132.4, 132.3, 130.3, 130.2 (2C), 130.1, 129.9 (2C), 129.1, 129.0, 128.9 (2C), 128.7 (2C), 128.51 (2C), 128.45 (2C), 128.1 (2C), 128.0 (2C), 127.4, 111.2, 83.0, 74.5, 65.9, 65.2, 63.4, 47.4, 39.2, 37.1, 26.9 (3C), 19.2, 15.6, 12.3 ppm; HRMS (ESI) *m*/*z*: calcd for C_50_H_55_N_2_O_10_S_2_Si [M+H]^+^ 935.3062, found 935.3069 (+0.75 ppm).

(–)-((2*S*,3*R*,4*S*,5*S*)-4-(benzoyloxy)-5-(((tert-butyldiphenylsilyl)oxy)methyl)-3-methyl-2-(5-methyl-2,4-dioxo-3,4-dihydropyrimidin-1(2H)-yl)tetrahydrothiophen-3-yl)methyl benzoate (**50**). To a stirred solution of C4′-OMs thioaminal **49** (13 mg, 1.0 equiv, 0.014 mmol) in a high-pressure flask, 2,6-lutidine (0.13 mL, 0.11 M) and NaI (22 mg, 10 equiv, 0.14 mmol) were added. The reaction mixture was stirred at 160 °C for 16 h in a sand bath. After completion of the reaction, the mixture was concentrated under reduced pressure. Purification by flash chromatography (Hexanes/EtOAc) provided the β-L-thiofuranoside **50** (8 mg, 77% yield). R_f_ = 0.64 (Hexanes/EtOAc, 30:70); [α]_D_^25^ −65 (*c* 0.6, CH_2_Cl_2_); formula: C_42_H_44_N_2_O_7_SSi; MW: 748.9660 g/mol; IR (neat) ν_max_ 3194, 3070, 2931, 2857, 1725, 1691, 1274, 1263, 1107 cm^−1^; ^1^H NMR (500 MHz, CDCl_3_) δ 8.02 (s, 1H), 7.98 (d, *J* = 1.3 Hz, 1H), 7.93 (dd, *J* = 8.1, 1.5 Hz, 2H), 7.77 (dd, *J* = 8.2, 1.5 Hz, 2H), 7.64 (dd, *J* = 8.1, 1.5 Hz, 2H), 7.60 (t, *J* = 7.5 Hz, 1H), 7.53 (dd, *J* = 7.8, 1.1 Hz, 2H), 7.45–7.33 (m, 9H), 7.27 (t, *J* = 7.4 Hz, 2H), 6.30 (s, 1H), 5.84 (d, *J* = 4.3 Hz, 1H), 4.28–4.23 (m, 2H), 4.22–4.12 (m, 2H), 3.81 (dd, *J* = 9.6, 7.4 Hz, 1H), 1.85 (s, 3H), 1.54 (s, 3H), 1.00 (s, 9H) ppm; ^13^C NMR (126 MHz, CDCl_3_) δ 166.0, 165.4, 162.9, 151.1, 138.3, 135.8 (2C), 135.6 (2C), 133.9, 133.3, 132.8, 132.7, 130.1, 130.0, 129.8 (2C), 129.6 (2C), 129.4, 129.0, 128.9 (2C), 128.5 (2C), 127.92 (2C), 127.90 (2C), 110.5, 80.0, 68.3, 64.0, 63.1, 55.8, 53.9, 26.8 (3C), 23.3, 19.3, 13.0 ppm; HRMS (ESI) *m*/*z*: calcd for C_42_H_45_N_2_O_7_SSi [M+H]^+^ 749.2711, found 749.2694 (–2.27 ppm).

((3*R*,4*S*,5*R*)-4-(benzoyloxy)-5-(hydroxymethyl)-3-methyl-2-(5-methyl-2,4-dioxo-3,4-dihydropyrimidin-1(2H)-yl)tetrahydrothiophen-3-yl)methyl benzoate (**61a,b**). To a stirred solution of C5′-protected thiofuranosides **47a,b** (241 mg, 1.00 equiv, 0.322 mmol) in anhydrous THF (1.3 mL, 0.25 M) at 0 °C, 3HF·NEt_3_ (0.13 mL, 2.5 equiv, 0.80 mmol) was added. The reaction mixture was stirred at room temperature for 18 h. After dilution with EtOAc, a saturated solution of NaHCO_3_ was added and the mixture was concentrated under reduced pressure. Purification by flash chromatography (Hexanes/EtOAc) provided **61a,b** (110 mg, 67% yield) as a 5:1 mixture in favor of the β-D-anomer. R_f_ = 0.49 and 0.53 (CH_2_Cl_2_/MeOH, 90:10); formula: C_26_H_26_N_2_O_7_S; MW: 510.5610 g/mol; ^1^H NMR (500 MHz, CDCl_3_) δ 9.62 (s, 1H, α-anomer), 9.51 (s, 1H, β-anomer), 8.60 (s, 1H, β-anomer), 8.24 (d, *J* = 8.3 Hz, 2H, β-anomer), 8.04 (d, *J* = 8.4 Hz, 2H, β-anomer), 7.90 (dd, *J* = 13.9, 8.3 Hz, 4H, α-anomer), 7.74 (s, 1H, α-anomer), 7.64–7.58 (m, 5H, α-anomer), 7.58–7.52 (m, 2H, β-anomer and α-anomer), 7.46 (t, *J* = 7.2 Hz, 4H, β-anomer), 7.39 (dt, *J* = 22.1, 7.4 Hz, 1H, β-anomer), 6.54 (s, 1H, β-anomer), 6.36 (s, 1H, α-anomer), 5.66 (d, *J* = 9.5 Hz, 1H, β-anomer), 5.52 (d, *J* = 3.9 Hz, 1H, α-anomer), 4.70 (d, *J* = 11.2 Hz, 1H, β-anomer), 4.64 (d, *J* = 11.2 Hz, 1H, β-anomer), 4.53 (d, *J* = 11.4 Hz, 1H, α-anomer), 4.40 (d, *J* = 11.5 Hz, 1H, α-anomer), 4.04 (appt, *J* = 3.3 Hz, 2H, α-anomer), 3.99 (s, 1H, α-anomer), 3.97–3.89 (m, 2H, β-anomer), 3.85 (appt, *J* = 6.7 Hz, 1H, α-anomer), 3.67 (appd, *J* = 9.5 Hz, 1H, β-anomer), 3.45 (s, 1H, β-anomer), 2.00 (s, 3H, β-anomer), 1.74 (s, 3H, α-anomer), 1.55 (s, 3H, α-anomer), 1.15 (s, 3H, β-anomer) ppm; ^13^C NMR (126 MHz, CDCl_3_) δ 166.71, 166.67, 165.9, 165.8, 164.0, 163.6, 151.5, 151.4, 138.0 (β-anomer), 137.8 (α-anomer), 134.3, 134.1, 133.5, 133.3, 130.0 (4C), 129.7 (2C), 129.6, 129.5, 129.2 (2C), 128.9 (2C), 128.7 (2C), 128.60 (2C), 128.57 (2C), 128.47, 128.36, 111.4 (β-anomer), 111.0 (α-anomer), 81.8 (α-anomer), 77.1 (β-anomer), 68.0 (α-anomer), 66.2 (β-anomer), 64.2 (2C, α-anomer), 62.7 (β-anomer), 59.9 (β-anomer), 56.5 (α-anomer), 54.3 (α-anomer), 53.7 (β-anomer), 50.8 (β-anomer), 23.1 (α-anomer), 17.3 (β-anomer), 12.9 (β-anomer), 12.8 (α-anomer) ppm; HRMS (ESI) *m*/*z*: calcd for C_26_H_26_N_2_NaO_7_S [M+Na]^+^ 533.1353, found 533.1356 (+0.56 ppm).

(+)-1-((2*R*,3*R*,4*S*,5*R*)-4-hydroxy-3,5-bis(hydroxymethyl)-3-methyltetrahydrothiophen-2-yl)-5-methylpyrimidine-2,4(1H,3H)-dione (**23a**) and (+)-1-((2*S*,3*R*,4*S*,5*R*)-4-hydroxy-3,5-bis(hydroxymethyl)-3-methyltetrahydrothiophen-2-yl)-5-methylpyrimidine-2,4(1H,3H)-dione (**23b**). To a stirred solution of 2′,3′-protected thiofuranosides 61a,b (24 mg, 1.0 equiv, 0.047 mmol) in MeOH (0.24 mL, 0.20 M), a solution of NaOMe (11 μL, 1.0 equiv, 0.047 mmol, 4.4 M in MeOH) was added. The reaction mixture was stirred at room temperature for 3 h. After the addition of formic acid until a neutral pH was reached, the mixture was concentrated under reduced pressure. Purification by C18 reverse-phase flash chromatography (H_2_O/MeOH) provided thiofuranosides **23a** (6 mg, 42% yield) and **23b** (3 mg, 21% yield). **23a**: R_f_= 0.13 (DCM/MeOH, 90:10); [α]^25^_D_ +23 (*c* 0.25, MeOH); formula: C_12_H_18_N_2_O_5_S; MW: 302.3450 g/mol; IR (neat) ν_max_ 3376, 2925, 1683, 1470 cm^−1^; ^1^H NMR (500 MHz, CD_3_OD): δ 8.44 (s, 1H), 6.17 (s, 1H), 4.04 (d, *J* = 9.6 Hz, 1H), 3.95 (dd, *J* = 11.9, 3.6 Hz, 1H), 3.90 (dd, *J* = 11.8, 2.6 Hz, 1H), 3.77 (d, *J* = 11.3 Hz, 1H), 3.70 (d, *J* = 11.3 Hz, 1H), 3.34 (ddd, *J* = 9.5, 3.6, 2.6 Hz, 1H), 1.90 (d, *J* = 1.2 Hz, 3H), 0.98 (s, 3H) ppm, labile protons were not observed due to exchange_;_ ^13^C NMR (126 MHz, CD_3_OD) δ 166.3, 153.0, 140.8, 110.9, 78.7, 65.0, 64.1, 61.0, 55.5, 54.6, 17.3, 12.5 ppm; HRMS (ESI) *m*/*z*: calcd for C_12_H_18_N_2_NaO_5_S [M+Na]^+^ 325.0829, found 325.0840 (+3.4 ppm). 23b: R_f_= 0.13 (DCM/MeOH, 90:10); [α]^25^_D_ +18 (*c* 0.18, MeOH); formula: C_12_H_18_N_2_O_5_S; MW: 302.3450 g/mol; IR (neat) ν_max_ 3368, 2926, 1682, 1468 cm^−1^; ^1^H NMR (500 MHz, CD_3_OD): δ 8.16 (d, *J* = 1.2 Hz, 1H), 6.01 (s, 1H), 3.96 (d, *J* = 5.1 Hz, 1H), 3.91 (dd, *J* = 10.9, 5.7 Hz, 1H), 3.87–3.82 (m, 1H), 3.62 (dd, *J* = 10.9, 7.5 Hz, 1H), 3.59 (d, *J* = 2.8 Hz, 2H), 1.89 (d, *J* = 1.2 Hz, 3H), 1.23 (s, 3H) ppm, labile protons were not observed due to exchange_;_ ^13^C NMR (126 MHz, CD_3_OD) δ 166.3, 153.5, 141.9, 110.1, 82.2, 68.8, 65.8, 63.4, 59.1, 55.4, 22.9, 12.6. ppm; HRMS (ESI) *m*/*z*: calcd for C_12_H_18_N_2_NaO_5_S [M+Na]^+^ 325.0829, found 325.0841 (+3.7 ppm).

(–)-((2*R*,3*R*,4*S*,5*R*)-4-(benzoyloxy)-2-(benzylthio)-5-(((tert-butyldiphenylsilyl)oxy)methyl)-3-methyltetrahydrothiophen-3-yl)methyl benzoate (**62**). To a solution of crude C4-OMs dithioacetal **45** (432 mg, 1.00 equiv, 0.463 mmol) in pyridine (4.3 mL, 0.10 M), tetrabutylammonium iodide (188 mg, 1.10 equiv, 0.509 mmol) and barium carbonate (112 mg, 1.23 equiv, 0.569 mmol) were added [28]. The reaction mixture was stirred at 80 °C for 3 h. After cooling to room temperature, the volatiles were removed under reduced pressure. Purification by flash chromatography (Hexanes/EtOAc) provided the pure product **62** (271 mg, 78% yield over two steps). R_f_ = 0.61 (Hexanes/EtOAc, 80:20); [α]_D_^25^ −56 (*c* 1.9, CH_2_Cl_2_); formula: C_44_H_46_O_5_S_2_Si; MW: 747.0520 g/mol; IR (neat) ν_max_ 3069, 2931, 2857, 1723, 1265, 1108 cm^−1^; ^1^H NMR (500 MHz, CDCl_3_) δ 7.92 (dd, *J* = 8.4, 1.3 Hz, 2H), 7.83 (dd, *J* = 8.4, 1.3 Hz, 2H), 7.66 (ddd, *J* = 8.1, 2.6, 1.5 Hz, 4H), 7.59–7.53 (m, 2H), 7.43–7.29 (m, 15H), 5.70 (d, *J* = 6.1 Hz, 1H), 4.55 (d, *J* = 11.0 Hz, 1H), 4.41 (s, 1H), 4.31 (d, *J* = 11.0 Hz, 1H), 4.03 (dd, *J* = 10.4, 5.7 Hz, 1H), 3.90 (d, *J* = 6.0 Hz, 2H), 3.87–3.81 (m, 1H), 3.65 (dt, *J* = 7.8, 5.9 Hz, 1H), 1.29 (s, 3H), 1.02 (s, 9H) ppm; ^13^C NMR (126 MHz, CDCl_3_) δ 166.3, 165.1, 137.3, 135.84 (2C), 135.75 (2C), 133.4, 133.3, 133.2, 129.9 (2C), 129.83, 129.82, 129.7 (2C), 129.5, 129.3 (2C), 128.7 (2C), 128.6 (2C), 128.5 (2C), 127.82 (2C), 127.78 (2C), 127.4, 81.1, 67.3, 66.4, 55.1, 52.9, 52.8, 37.3, 26.9 (3C), 19.3, 18.7 ppm, due to overlapping carbon signals in the aromatic region 2 peaks are hidden; HRMS (ESI) *m*/*z*: calcd for C_44_H_46_NaO_5_S_2_Si [M+Na]^+^ 769.2448, found 769.2453 (+0.65 ppm).

((3*R*,4*S*,5*R*)-4-(benzoyloxy)-5-(((tert-butyldiphenylsilyl)oxy)methyl)-3-methyl-2-(5-methyl-2,4-dioxo-3,4-dihydropyrimidin-1(2H)-yl)tetrahydrothiophen-3-yl)methyl benzoate (**47a,b**). To a stirred solution of thiofuranoside **62** (32 mg, 1.0 equiv, 0.043 mmol) in anhydrous DCE (0.43 mL, 0.10 M) at room temperature, silylated thymine (0.78 M in MeCN, 0.16 mL, 3.0 equiv, 0.13 mmol) was added. The resulting solution was cooled to 0 °C and dimethyl(methylthio)sulfonium tetrafluoroborate (34 mg, 4.0 equiv, 0.17 mmol) was added. The reaction mixture was stirred at room temperature for 3 h. After cooling to 0 °C, the reaction was quenched by the addition of H_2_O and dissolved in EtOAc. The aqueous layer was extracted (3×) with EtOAc and the combined organic layers were washed with brine, dried over MgSO_4_, filtered, and concentrated under reduced pressure. ^1^H NMR analysis of the crude mixture indicated a 1.2:1 ratio of nucleosides. Purification by flash chromatography (Hexanes/EtOAc) provided a mixture of products **47a,b** (22 mg, 69% yield) in a 1.3:1 (β:α) ratio. R_f_ = 0.79 (Hexanes/EtOAc, 30:70); formula: C_42_H_44_N_2_O_7_SSi; MW: 748.9660 g/mol; IR (neat) ν_max_ 3190, 3069, 2930, 2857, 1720, 1686, 1263, 1104 cm^−1^; ^1^H NMR (500 MHz, CDCl_3_) δ 8.35 (s, 1H), 8.33 (s, 1H), 8.23 (dd, *J* = 8.3, 1.1 Hz, 2H), 7.98 (dd, *J* = 8.3, 1.2 Hz, 2H), 7.89 (ddd, *J* = 14.0, 8.3, 1.2 Hz, 3H), 7.77 (s, 1H, β-anomer), 7.74 (d, *J* = 1.2 Hz, 1H, α-anomer), 7.71–7.64 (m, 8H), 7.64–7.51 (m, 5H), 7.50–7.35 (m, 15H), 7.35–7.28 (m, 5H), 6.56 (s, 1H, β-anomer), 6.27 (s, 1H, α-anomer), 5.65 (d, *J* = 9.8 Hz, 1H, β-anomer), 5.42 (d, *J* = 3.8 Hz, 1H, α-anomer), 4.61 (d, *J* = 11.3 Hz, 1H, β-anomer), 4.54 (d, *J* = 11.3 Hz, 1H, β-anomer), 4.47 (d, *J* = 11.4 Hz, 1H, α-anomer), 4.32 (d, *J* = 11.4 Hz, 1H, α-anomer), 4.19 (dd, *J* = 10.2, 5.2 Hz, 1H, α-anomer), 4.07–4.01 (m, 2H, β-anomer and α-anomer), 3.90 (dd, *J* = 10.9, 6.4 Hz, 1H, β-anomer), 3.84 (ddd, *J* = 11.3, 7.4, 4.4 Hz, 2H, β-anomer and α-anomer), 1.80 (d, *J* = 1.1 Hz, 3H, α-anomer), 1.76 (d, *J* = 0.9 Hz, 3H, β-anomer), 1.41 (s, 3H, α-anomer), 1.09 (s, 3H, β-anomer), 1.07 (s, 9H, α-anomer), 1.05 (s, 9H, β-anomer) ppm; ^13^C NMR (126 MHz, CDCl_3_) δ 166.7, 165.9, 165.4, 165.1, 163.3, 163.2, 151.10, 151.09, 138.1, 137.0, 135.9 (2C), 135.7 (4C), 135.6 (2C), 133.99, 133.96, 133.54, 133.50, 133.1, 133.0, 132.8, 132.7, 130.12, 130.10, 130.07 (2C), 130.06, 130.04, 130.02 (2C), 129.7 (2C), 129.62 (2C), 129.61, 129.4, 128.92, 128.87 (2C), 128.82 (2C), 128.76, 128.73 (2C), 128.6 (2C), 127.99 (2C), 127.96 (2C), 127.95 (2C), 127.92 (2C), 111.7, 110.8, 81.3 (α-anomer), 77.6 (β-anomer), 68.3 (α-anomer), 66.4 (α-anomer), 66.1 (β-anomer), 64.3 (α-anomer), 64.2 (β-anomer), 62.2 (β-anomer), 57.7 (β-anomer), 54.5, 53.2, 51.7 (α-anomer), 27.0 (β-anomer, 3C), 26.9 (α-anomer, 3C), 23.1 (α-anomer), 19.5 (β-anomer), 19.4 (α-anomer), 17.3 (β-anomer), 12.9 (α-anomer), 12.8 (β-anomer) ppm; HRMS (ESI) *m*/*z*: calcd for C_42_H_45_N_2_NaO_7_SSi [M+Na]^+^ 771.2531, found 771.2534 (+0.39 ppm).

(2*R*,3*S*,4*R*)-5-acetoxy-4-((benzoyloxy)methyl)-2-(((tert-butyldiphenylsilyl)oxy)methyl)-4-methyltetrahydrothiophen-3-yl benzoate (**63a,b**). To a stirred solution of thiofuranoside **62** (156 mg, 1.00 equiv, 0.209 mmol) in acetic acid (1.6 mL, 0.13 M), mercury acetate (133 mg, 2.00 equiv, 0.418 mmol) was added. After stirring at room temperature for 2 h, the mixture was concentrated under reduced pressure. Purification by flash chromatography (Hexanes/EtOAc) provided **63a,b** (129 mg, 91% yield) as a 3:1 mixture in favor of the β-anomer. R_f_ = 0.43 and 0.40 (Hexanes/EtOAc, 80:20); formula: C_39_H_42_O_7_SSi; MW: 682.2421 g/mol; ^1^H NMR (500 MHz, CDCl_3_) δ 8.06–8.05 (m, 4H), 8.04 (d, *J* = 1.2 Hz, 4H), 8.02 (d, *J* = 1.1 Hz, 1H), 8.00 (d, *J* = 1.4 Hz, 1H), 7.97 (d, *J* = 1.2 Hz, 1H), 7.95 (d, *J* = 1.4 Hz, 1H), 7.69 (t, *J* = 1.3 Hz, 1H), 7.68 (dd, *J* = 3.3, 1.5 Hz, 2H), 7.67 (d, *J* = 1.6 Hz, 1H), 7.64–7.63 (m, 2H), 7.63–7.61 (m, 2H), 7.60–7.57 (m, 5H), 7.56 (dd, *J* = 3.0, 1.7 Hz, 1H), 7.47–7.42 (m, 5H), 7.41–7.38 (m, 1H), 7.38–7.34 (m, 4H), 7.33–7.27 (m, 4H), 6.13 (s, 1H, β-anomer), 6.06 (s, 1H, α-anomer), 5.84 (d, *J* = 9.0 Hz, 1H, β-anomer), 5.46 (d, *J* = 2.7 Hz, 1H, α-anomer), 4.64 (d, *J* = 11.3 Hz, 1H, β-anomer), 4.61 (d, *J* = 11.5 Hz, 1H, β-anomer), 4.58 (d, *J* = 11.0 Hz, 1H, α-anomer), 4.49 (d, *J* = 11.0 Hz, 1H, α-anomer), 4.09 (dd, *J* = 10.1, 5.8 Hz, 1H, α-anomer), 3.99 (ddd, *J* = 8.6, 5.7, 2.8 Hz, 1H, α-anomer), 3.90 (dd, *J* = 10.7, 4.7 Hz, 1H, β-anomer), 3.84–3.81 (m, 1H, α-anomer), 3.79 (dd, *J* = 10.7, 6.6 Hz, 1H, β-anomer), 3.66 (ddd, *J* = 9.1, 6.5, 4.7 Hz, 1H, β-anomer), 2.15 (s, 3H, α-anomer), 2.11 (s, 3H, β-anomer), 1.31 (s, 3H, α-anomer), 1.28 (s, 3H, β-anomer), 1.05 (s, 9H, α-anomer), 1.00 (s, 9H, β-anomer) ppm; ^13^C NMR (126 MHz, CDCl_3_) δ 170.2 (β-anomer), 170.1 (α-anomer), 166.5 (β-anomer), 166.3 (α-anomer), 165.5 (β-anomer), 165.3 (α-anomer), 135.9 (2C), 135.80 (2C), 135.77 (2C), 135.73 (2C), 133.7, 133.6, 133.4, 133.3, 133.2, 133.1, 133.04, 133.01, 130.0 (2C), 129.94, 129.92, 129.87 (2C), 129.84 (2C), 129.81, 129.79 (2C), 129.73 (2C), 129.69, 129.3 (2C), 128.7 (4C), 128.64, 128.62, 127.88 (2C), 127.85 (2C), 127.80 (2C), 127.75 (2C), 86.5 (α-anomer), 81.4 (α-anomer), 81.3 (β-anomer), 79.1 (β-anomer), 77.4 (α-anomer), 66.2 (β-anomer), 65.6 (β-anomer), 64.9 (α-anomer), 57.4 (α-anomer), 53.5 (α-anomer), 52.5 (β-anomer), 50.9 (β-anomer), 26.9 (3C, α-anomer), 26.8 (3C, β-anomer), 21.7 (α-anomer), 21.40 (β-anomer), 21.37 (α-anomer), 19.34 (α-anomer), 19.28 (β-anomer), 17.0 (β-anomer) ppm, due to overlapping carbon signals in the aromatic region 2 peaks are hidden; HRMS (ESI) *m*/*z*: calcd for C_39_H_42_NaO_7_SSi [M+Na]^+^ 705.2313, found 705.2290 (−3.26 ppm).

(–)-((2*R*,3*S*,4*R*,5*R*)-5-(6-amino-2-chloro-9H-purin-9-yl)-4-((benzoyloxy)methyl)-2-(((tert-butyldiphenylsilyl)oxy)methyl)-4-methyltetrahydrothiophen-3-yl benzoate (**64a**) and (–)-(2*R*,3*S*,4*R*,5*S*)-5-(6-amino-2-chloro-9H-purin-9-yl)-4-((benzoyloxy)methyl)-2-(((tert-butyldiphenylsilyl)oxy)methyl)-4-methyltetrahydrothiophen-3-yl benzoate (**64b**). To a suspension of 2-chloroadenine (23 mg, 2.0 equiv, 0.14 mmol) in anhydrous DCE (0.67 mL, 0.20 M), BSA (0.11 mL, 6.5 equiv, 0.44 mmol) was added. The reaction mixture was refluxed at 84 °C until a clear solution was obtained. After cooling to −10 °C, the mixture was added to a solution of thiofuranosides **63a,b** (46 mg, 1.0 equiv, 0.067 mmol) in anhydrous DCE (0.67 mL, 0.10 M), followed by dropwise addition of TMSOTf (25 μL, 2.0 equiv, 0.14 mmol). The resulting solution was stirred at 84 °C for 2 h. The crude was dissolved in EtOAc, and a saturated solution of NaHCO_3_ was added. The aqueous layer was extracted (3×) with EtOAc and the combined organic layers were washed with brine, dried over MgSO_4_, filtered, and concentrated under reduced pressure. ^1^H NMR analysis of the crude mixture indicated a 1.0:1.1 ratio of β:α N9-thionucleosides. Purification by flash chromatography (Hexanes/EtOAc) provided thionucleosides **64a** (13 mg, 24% yield) and **64b** (23 mg, 43% yield). **64a**: R_f_ = 0.86 (Hexanes/EtOAc, 30:70); [α]_D_^25^ −33 (*c* 0.8, CH_2_Cl_2_); formula: C_42_H_42_ClN_5_O_5_SSi; MW: 792.4230 g/mol; IR: (neat) ν_max_ 3323, 3171, 2958, 2859, 1727, 1644, 1263, 1108 cm^−1^; ^1^H NMR (500 MHz, CDCl_3_) δ 8.58 (s, 1H), 8.37 (d, *J* = 7.4 Hz, 2H), 8.00 (d, *J* = 7.6 Hz, 2H), 7.69 (t, *J* = 6.9 Hz, 4H), 7.63 (dd, *J* = 14.0, 7.2 Hz, 2H), 7.57 (t, *J* = 7.5 Hz, 2H), 7.47 (t, *J* = 7.7 Hz, 2H), 7.44–7.35 (m, 4H), 7.29 (t, *J* = 7.4 Hz, 2H), 6.42 (s, 1H), 5.99 (s, 2H), 5.97 (d, *J* = 9.7 Hz, 1H), 4.68 (d, *J* = 11.1 Hz, 1H), 4.63 (d, *J* = 11.1 Hz, 1H), 3.99 (dd, *J* = 11.1, 3.1 Hz, 1H), 3.90 (dd, *J* = 11.1, 5.5 Hz, 1H), 3.84 (m, 1H), 1.11 (s, 9H), 0.88 (s, 3H) ppm; ^13^C NMR (126 MHz, CDCl_3_) δ 166.8, 165.2, 156.3, 154.6, 151.9, 141.0, 135.9 (2C), 135.7 (2C), 134.0, 133.7, 132.7, 132.4, 130.2 (2C), 130.12, 130.10, 130.0 (2C), 129.5, 129.0 (2C), 128.82 (2C), 128.80, 128.1 (2C), 128.0 (2C), 118.2, 77.1, 66.0, 63.5, 60.3, 53.7, 51.2, 27.0 (3C), 19.3, 17.5 ppm; HRMS (ESI) *m*/*z*: calcd for C_42_H_43_ClN_5_O_5_SSi [M+H]^+^ 792.2437, found 792.2434 (–0.38 ppm). 64b: R_f_ = 0.74 (Hexanes/EtOAc, 30:70); [α]_D_^25^ −4 (*c* 0.7, CH_2_Cl_2_); formula: C_42_H_42_ClN_5_O_5_SSi; MW: 792.4230 g/mol; IR (neat) ν_max_ 3320, 3168, 3071, 2931, 2858, 1725, 1266, 1110 cm^−1^; ^1^H NMR (500 MHz, CDCl_3_) δ 8.38 (s, 1H), 7.91–7.87 (m, 2H), 7.84 (dd, *J* = 8.1, 1.0 Hz, 2H), 7.68 (ddd, *J* = 7.8, 3.7, 1.4 Hz, 4H), 7.60 (t, *J* = 7.4 Hz, 1H), 7.53 (t, *J* = 7.5 Hz, 1H), 7.48–7.33 (m, 10H), 6.19 (s, 1H), 5.82 (s, 2H), 5.53 (d, *J* = 4.4 Hz, 1H), 4.34 (d, *J* = 11.7 Hz, 1H), 4.21–4.13 (m, 3H), 3.91–3.86 (m, 1H), 1.48 (s, 3H), 1.06 (s, 9H) ppm; ^13^C NMR (126 MHz, CDCl_3_) δ 165.9, 165.2, 156.0, 154.6, 151.7, 141.2, 135.9 (2C), 135.8 (2C), 134.0, 133.5, 133.0, 132.8, 130.1 (2C), 129.9 (2C), 129.7 (2C), 129.2, 128.9 (2C), 128.8, 128.7 (2C), 128.0 (2C), 127.9 (2C), 117.9, 81.3, 65.9, 65.5, 64.0, 57.1, 54.4, 26.9 (3C), 22.5, 19.4 ppm; HRMS (ESI) *m*/*z*: calcd for C_42_H_43_ClN_5_O_5_SSi [M+H]^+^ 792.2437, found 792.2433 (−0.51 ppm).

((3*R*,4*S*,5*R*)-4-(benzoyloxy)-5-(((tert-butyldiphenylsilyl)oxy)methyl)-2-(2,6-dichloro-9H-purin-9-yl)-3-methyltetrahydrothiophen-3-yl)methyl benzoate (**65a,b**). To a stirred solution of thiofuranosides **63a,b** (33 mg, 1.0 equiv, 0.048 mmol) in anhydrous MeCN (2.8 mL, 0.25 M), 2,6-dichloropurine (10 mg, 1.1 equiv, 0.053 mmol) was added. The resulting solution was cooled to −10 °C and DBU (22 μL, 3.0 equiv, 0.15 mmol) was added, followed by dropwise addition of TMSOTf (36 μL, 4.0 equiv, 0.19 mmol). The reaction mixture was stirred at room temperature for 16 h. The crude was dissolved in EtOAc, and a saturated solution of NaHCO_3_ was added. The aqueous layer was extracted (3×) with EtOAc and the combined organic layers were washed with brine, dried over MgSO_4_, filtered, and concentrated under reduced pressure. ^1^H NMR analysis of the crude mixture indicated a 1.3:1 ratio of β:α N9-thionucleosides. Purification by flash chromatography (Hexanes/EtOAc) provided a mixture of thionucleosides **65a,b** (34 mg, 87% yield) in a 1.3:1 ratio in favor of the β-anomer. R_f_ = 0.31 (Hexanes/EtOAc, 80:20); formula: C_42_H_40_Cl_2_N_4_O_5_SSi; MW: 811.8500 g/mol; ^1^H NMR (500 MHz, CDCl_3_) δ 8.91 (s, 1H, β-anomer), 8.69 (s, 1H, α-anomer), 8.33 (d, *J* = 7.0 Hz, 2H, β-anomer), 8.01 (d, *J* = 7.1 Hz, 2H, β-anomer), 7.88 (d, *J* = 7.1 Hz, 2H, α-anomer), 7.81 (d, *J* = 7.2 Hz, 2H, α-anomer), 7.71–7.66 (m, 9H), 7.65–7.61 (m, 2H), 7.59–7.54 (m, 4H), 7.48 (td, *J* = 7.9, 3.3 Hz, 4H), 7.44–7.35 (m, 11H), 7.29 (t, *J* = 7.3 Hz, 2H), 6.48 (s, 1H, β-anomer), 6.23 (s, 1H, α-anomer), 5.98 (d, *J* = 9.6 Hz, 1H, β-anomer), 5.56 (d, *J* = 4.3 Hz, 1H, α-anomer), 4.71 (d, *J* = 11.2 Hz, 1H, β-anomer), 4.64 (d, *J* = 11.1 Hz, 1H, β-anomer), 4.30 (d, *J* = 11.7 Hz, 1H, α-anomer), 4.22–4.19 (m, 2H, α-anomer), 4.19 (d, *J* = 11.8 Hz, 1H, α-anomer), 4.00 (dd, *J* = 11.0, 3.1 Hz, 1H, β-anomer), 3.95–3.85 (m, 3H), 1.51 (s, 3H, α-anomer), 1.12 (s, 9H, β-anomer), 1.07 (s, 9H, α-anomer), 0.89 (s, 3H, β-anomer) ppm; ^13^C NMR (126 MHz, CDCl_3_) δ 166.6 (β-anomer), 165.7 (α-anomer), 165.2 (α-anomer), 165.1 (β-anomer), 153.6, 153.44, 153.42 (β-anomer), 153.3 (α-anomer), 152.5, 152.2, 146.2 (α-anomer), 145.9 (β-anomer), 135.9 (2C), 135.8 (2C), 135.7 (2C), 134.11, 134.08, 133.8, 133.7, 132.9, 132.7, 132.6, 132.3, 131.1 (β-anomer), 130.8 (α-anomer), 130.18, 130.14, 130.13, 130.11, 130.10 (2C), 130.0 (2C), 129.8 (2C), 129.6 (2C), 129.4, 129.04 (2C), 128.95 (2C), 128.90, 128.85 (2C), 128.76 (2C), 128.64, 128.60, 128.1 (2C), 127.99 (2C), 127.98 (2C), 127.96 (2C), 81.4 (α-anomer), 76.9 (β-anomer), 66.4 (α-anomer), 65.9 (β-anomer), 65.8 (α-anomer), 63.8 (α-anomer), 63.4 (β-anomer), 61.1 (β-anomer), 57.6 (α-anomer), 54.7 (α-anomer), 53.7 (β-anomer), 51.5 (β-anomer), 27.0 (3C, β-anomer), 26.9 (3C, α-anomer), 22.6 (α-anomer), 19.34 (α-anomer), 19.29 (β-anomer), 17.5 (β-anomer) ppm; HRMS (ESI) *m*/*z*: calcd for C_42_H_41_Cl_2_N_4_O_5_SSi [M+H]^+^ 811.1939, found 811.1938 (−0.12 ppm).

(+)-((2*R*,3*S*,4*R*,5*R*)-5-(6-Amino-2-chloro-9H-purin-9-yl)-3-hydroxy-4-methyltetrahydrothiophene-2,4-diyl)dimethanol (**24a**) and (+)-((2*R*,3*S*,4*R*,5*S*)-5-(6-Amino-2-chloro-9H-purin-9-yl)-3-hydroxy-4-methyltetrahydrothiophene-2,4-diyl)dimethanol (**24b**). To a mixture of thionucleosides **65a,b** (226 mg, 1.00 equiv, 0.278 mmol) in anhydrous THF (1.1 mL, 0.25 M) at 0 °C, 3HF.NEt_3_ (0.11 mL, 2.5 equiv, 0.69 mmol) was added. The reaction mixture was stirred at room temperature for 16 h. After dilution with EtOAc, a saturated solution of NaHCO_3_ was added and the mixture was concentrated under reduced pressure. The reaction mixture was passed through a pad of silica allowing for a ^1^H NMR of each C5′-OH product to be obtained. Major isomer: R_f_ = 0.61 (CH_2_Cl_2_/MeOH, 90:10); formula: C_26_H_22_Cl_2_N_4_O_5_S; MW: 573.4450 g/mol; ^1^H NMR (500 MHz, CDCl_3_) δ 9.79 (s, 1H), 8.37 (d, *J* = 8.1 Hz, 2H), 7.76 (d, *J* = 8.2 Hz, 2H), 7.65 (t, *J* = 6.8 Hz, 1H), 7.62–7.55 (m, 3H), 7.43 (t, *J* = 7.2 Hz, 2H), 6.47 (s, 1H), 5.95 (d, *J* = 9.5 Hz, 1H), 5.08 (apps, 1H), 4.77 (d, *J* = 11.1 Hz, 1H), 4.69 (d, *J* = 11.1 Hz, 1H), 4.20 (dd, *J* = 12.6, 6.0 Hz, 1H), 4.04 (dd, *J* = 12.5, 4.9 Hz, 1H), 3.84 (appd, *J* = 9.5 Hz, 1H), 0.95 (s, 3H) ppm; HRMS (ESI) *m*/*z*: calcd for C_26_H_23_Cl_2_N_4_O_5_S [M+H]^+^ 573.0761, found 573.0750 (−1.75 ppm). Minor isomer: R_f_ = 0.55 (CH_2_Cl_2_/MeOH, 90:10); formula: C_26_H_22_Cl_2_N_4_O_5_S; MW: 573.4450 g/mol; ^1^H NMR (500 MHz, CDCl_3_) δ 8.71 (s, 1H), 7.94 (d, *J* = 7.9 Hz, 2H), 7.76 (d, *J* = 8.0 Hz, 2H), 7.62 (t, *J* = 7.4 Hz, 1H), 7.56 (t, *J* = 7.4 Hz, 1H), 7.51–7.45 (m, 2H), 7.43–7.39 (m, 2H), 6.30 (s, 1H), 5.62 (d, *J* = 5.1 Hz, 1H), 4.44 (d, *J* = 11.8 Hz, 1H), 4.32 (d, *J* = 11.8 Hz, 1H), 4.19 (appq, *J* = 5.7 Hz, 1H), 4.08 (dd, *J* = 11.4, 5.4 Hz, 1H), 3.95–3.89 (m, 1H), 2.78 (s, 1H), 1.62 (s, 3H) ppm; HRMS (ESI) *m*/*z*: calcd for C_26_H_22_Cl_2_N_4_NaO_5_S [M+Na]^+^ 595.0580, found 595.0573 (−1.18 ppm). To a mixture of C5′-alcohols (34 mg, 1.0 equiv, 59 umol) in anhydrous MeOH (1.5 mL, 0.040 M) in a high-pressure flask, NH_3_ was bubbled until saturation. The reaction mixture was warmed to 80 °C for 48 h. The mixture was then concentrated under reduced pressure. Purification by flash chromatography (Hexanes/EtOAc/MeOH) provided the pure products 24a and 24b (18 mg, 88% yield over two steps). **24a**: R_f_= 0.25 (DCM/MeOH, 9:1); [α]^25^_D_ +45 (c 0.11, MeOH); IR (neat) ν_max_ 3316, 2881, 2509, 2327, 1630 cm^−1^; formula: C_12_H_16_ClN_5_O_3_S; MW: 345.8020 g/mol; ^1^H NMR (500 MHz, CD_3_OD): δ 8.65 (s, 1H), 6.00 (s, 1H), 4.32 (apps, 1H), 4.05 (dd, *J* = 11.3, 4.6 Hz, 1H), 3.99 (dd, *J* = 11.5, 2.9 Hz, 1H), 3.84 (s, 2H), 3.45 (ddd, *J* = 9.7, 5.0, 2.8 Hz, 1H), 0.74 (s, 3H) ppm, OH and NH_2_ signals are missing due to exchange_;_ ^13^C NMR (126 MHz, CD_3_OD) δ 158.2, 155.3, 152.3, 143.2, 118.8, 78.9, 64.5, 62.8, 62.3, 55.5, 54.7, 17.5 ppm; HRMS (ESI) *m*/*z*: calcd for C_12_H_17_ClN_5_O_3_S [M+H]^+^ 346.0735; found 346.0740 (+1.44 ppm). **24b**: R_f_= 0.15 (DCM/MeOH, 9:1); [α]^25^_D_ +4 (c 0.8, MeOH) IR (neat) ν_max_ 3346, 2931, 2384, 1615 cm^−1^; formula: C_12_H_16_ClN_5_O_3_S; MW: 345.8020 g/mol; ^1^H NMR (500 MHz, CD_3_OD): δ 8.56 (s, 1H), 5.90 (s, 1H), 4.03 (d, *J* = 5.1 Hz, 1H), 3.97 (m, 2H), 3.67 (dd, *J* = 12.7, 9.3 Hz, 1H), 3.55 (d, *J* = 11.2 Hz, 1H), 3.47 (d, *J* = 11.2 Hz, 1H), 1.29 (s, 3H); OH and NH_2_ signals are missing due to exchange_;_ ^13^C NMR (126 MHz, CD_3_OD) δ 158.0, 155.1, 152.4, 144.0, 118.4, 82.0, 66.6, 65.7, 63.2, 59.0, 55.2, 22.2 ppm; HRMS (ESI) *m*/*z*: calcd for C_12_H_17_ClN_5_O_3_S [M+H]^+^ 346.0735; found 346.0731 (–1.16 ppm).

### 3.3. General Information—DFT Calculations

Quantum mechanics calculations were conducted in Gaussian 16 [23] using the M06-2X [24,25] density functional in conjunction with the 6-31G* basis set, the LANLDZpd [30,31] effective core potential for Iodide, and using the polarizable continuum solvation model for 2,6-lutidine (PCM) [26]. Frequency calculations were carried out on all optimized geometries to distinguish minima (no imaginary frequencies) or transition structures (one imaginary frequency). The geometry and transition state optimizations (Berny algorithm) were achieved with tight SCF convergence and an ultrafine integral. The different conformations of the rotamers and the C2′-endo and C2′-exo ring conformations were evaluated. Using the molecular mechanics force field (MMFF94), a systematic conformational search considering all rotable bonds was performed for **51**, **54**, and **58**. The best candidates for each search where then reoptimized using the reported DFT method above, to identify the lowest energy structure. The energies presented in this paper and the Supporting Information are all from fully optimized structures. Orbital stabilization energies were obtained from the NBO theory (version 7) implemented in Gaussian 16. Gibbs free energies of activation (DDG^‡^) correspond to the energy difference between the lowest TS energy and lowest thioaminal bearing the C4′-OMs group. All energies in kcal/mol are presented relative to **51** lowest minima.

## Figures and Tables

**Table 1:** Dithioacetal formation from aldehyde **38**.

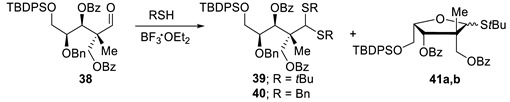				
Entry	R	Conditions ^a^	Yield (%) Dithioacetal ^b^	Yield 41a,b ^b^ (%)
1	*t*Bu	BF_3_·OEt_2_ (2.5 equiv.), 5 h	0	84
2	*t*Bu	BF_3_·OEt_2_ (1.3 equiv.), 2 h	0	91
3	Bn	BF_3_·OEt_2_ (1.3 equiv.), 2 h	87	0

^a^ Reaction conditions: RSH (4.0 equiv.), L.A., −60 °C, DCM (0.1 M). ^b^ Isolated yields.

**Table 2:** Dithioacetal formation from lactols **42a,b**.

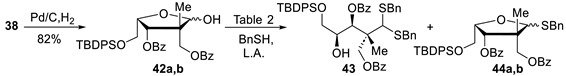				
Entry	Lewis Acid (Equiv.)	Conditions ^a^	Yield 43 ^b^ (%)	Yield 44a,b ^b^ (%)
1	BF_3_·OEt_2_ (2.5)	2 h, −40 °C	0	87
2	SnCl_4_ (1.1)	16 h, −78 °C	44	46
3	TiCl_4_ (2.6)	4 h, −20 °C	80	0

^a^ Reaction conditions: BnSH (4.0 equiv.), L.A., DCM (0.1 M). ^b^ Isolated yields.

**Table 3:** Thioaminal Formation.

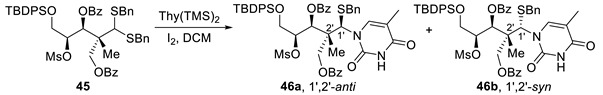			
Entry	Temperature ^a^	Ratio (46a:46b) ^b^	Yield ^c^ (%)
1	−20 °C	1.4:1	57
2	0 °C	1.2:1	76
3	RT	1.1:1	77
4	50 °C	1.0:1	82

^a^ Reaction conditions: Thy(TMS)_2_ (3.0 equiv.), I_2_ (2.1 equiv.), DCM (0.2 M). ^b^ Determined by ^1^H NMR analysis of the crude reaction mixture. ^c^ Isolated yields.

**Table 4:** Thioaminal Cyclization.

Entry	Thioaminal	Ratio (47a:47b) ^a,b^	Yield ^c^
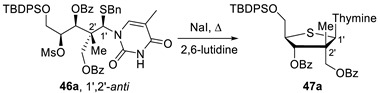			
1	**46a** (1′,2′-*anti*)	>20:1	**47a** (94%)
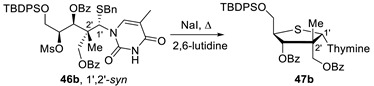			
2	**46b** (1′,2′-*syn*)	1:>20	**47b** (17%); **48** (25%); (43% Recovered SM **46b**)
3	**46a:46b** (1.1:1)	7:1	**47a** (49%); **47b** (5%); 48 (6%); (35% Recovered SM **46b**)

^a^ Reaction conditions: NaI (3 or 10 equiv.), 2,6-lutidine (0.1 M), reaction in high-pressure flask in 160 °C sand bath, 16 h. ^b^ Determined by ^1^H NMR analysis of crude reaction mixture. ^c^ Isolated yields.

**Table 5:** Formation of 4′-thioanalogues bearing a purine nucleobase.

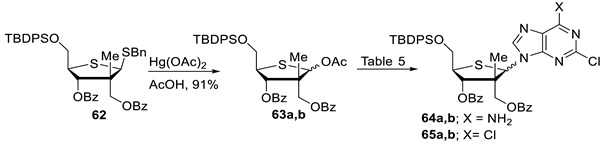				
Entry	Conditions	Temp	Ratio a,b ^a^	Yield (%) ^b^
1	2-chloroadenine, BSA, TMSOTf, DCE, 5 h	RT	**64a,b** 1.0:1.3	68
2	2-chloroadenine, BSA, TMSOTf, DCE, 2 h	84 °C	**64a,b** 1.0:1.1	67
3	2,6-dichloropurine, TMSOTf, DBU, MeCN, 16 h	RT	**65a,b** 1.3:1.0	88
4	2,6-dichloropurine, TMSOTf, DBU, MeCN, 16 h	84 °C	**65a,b** 5:1	84

^a^ Determined by ^1^H NMR analysis of the crude reaction mixture. ^b^ Isolated yields.

## Data Availability

The data presented in this study are available within the paper and the Supporting Information.

## Supplementary Materials

The following supporting information can be downloaded at: https://www.mdpi.com/article/10.3390/molecules29071647/s1, Stereochemical Proofs including HSQC, HMBC and nOe correlations; DFT free energy profile data; ^1^H, ^13^C and 2D NMR spectra.
