# Supplementary material for: Synthesis of 4′-Thionucleoside Analogues Bearing a C2′ Stereogenic All-Carbon Quaternary Center
Source: Molecules. 2024 Apr 6;29(7):1647. doi: 10.3390/molecules29071647 (PMC11013827; doi:10.3390/molecules29071647)

Synthesis of 4'-Thionucleoside Analogues Bearing a C2' Stereogenic All-Carbon Quaternary Center

Carla Eymard [1,2], Amarender Manchoju [1], Abir Almazloum [3], Starr Dostie [1], Michel Prévost [1,*], Mona Nemer [3], and Yvan Guindon [1,2,3,*]

[1] Bioorganic Chemistry Laboratory, Institut de recherches cliniques de Montréal (IRCM), Montréal, Québec, H2W 1R7, Canada;

[2] Department of Chemistry, Université de Montréal, Montréal, Québec, H3C 3J7, Canada

[3] Department of Biochemistry, Microbiology and Immunology, University of Ottawa, Ottawa, Ontario, K1N 6N5, Canada

* Correspondence: michel.prevost@ircm.qc.ca (M.P.); yvan.guindon@ircm.qc.ca (Y.G.)

Part I. Stereochemical Proofs…………………………………………………...….S2

Part II. DFT Studies…………………………………………………………......S7

Part III. ^{1}H, ^{13}C and 2D NMR spectra……………………………………………S33

Part I. Stereochemical Proofs

The peaks in the ^{1}H NMR spectra were assigned using $^{1}H/^{1}H$ 2D COSY, $^{1}H/^{13}C$ 2D HSQC experiments, chemical shifts, and coupling constants. Formation of the 3,4-*syn* stereochemistry for the Mukaiyama aldol reaction along with the stereochemistry of the C2-bromide is supported by relevant nuclear Overhauser effect (nOe) enhancements (2D NOESY) of lactone **S3** resulting from silyl group deprotection of **34a**. Further support for a 3,4-*syn* aldol reaction is obtained from 2D NOESY effects of the final thioanalogues as shown below.

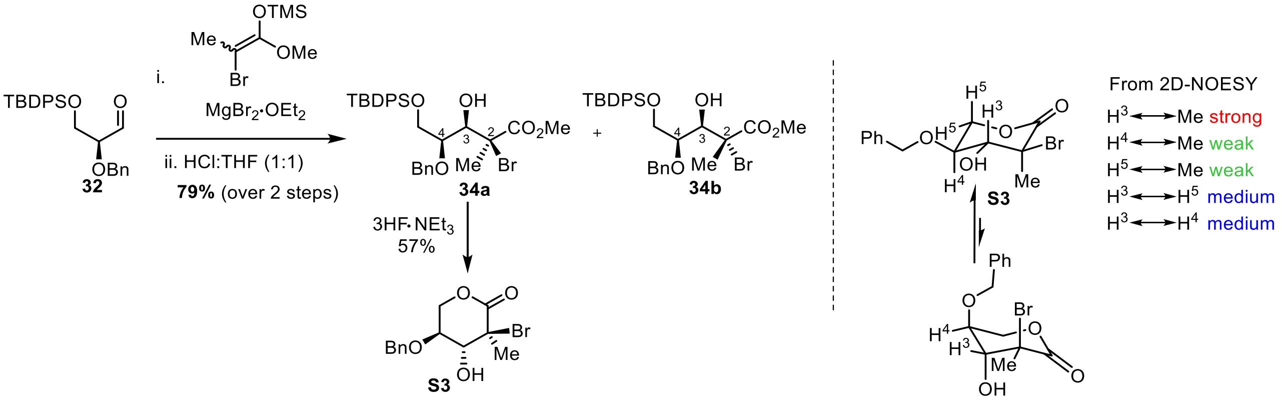

The structure of **34b** as the C2-bromo diastereomer was confirmed from the subsequent vinyl group transfer in which a mixture of dimethylvinylsilanes **35a,b** resulted in formation of only one methylester **36**. The stereochemistry of the C2 all-carbon quaternary center was confirmed in the subsequent cyclized intermediates.

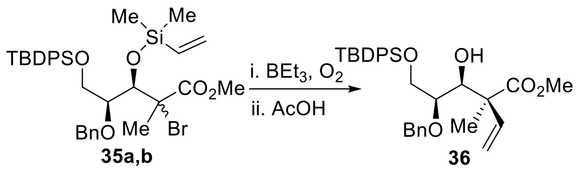

Determining the stereochemistry of the cyclized furanoside and thiofuranoside compounds supports the 3,4-*syn* stereochemistry for the Mukaiyama aldol reaction and the stereochemistry of the C2 all-carbon quaternary center.

NOE confirmed α-L-anomer to be the major isomer for 41a,b

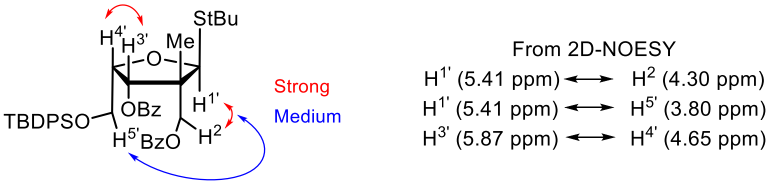

NOE confirmed β-L-anomer to be the major isomer for 42a,b

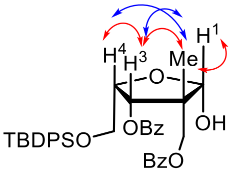

From 2D-NOESY

H^1 (5.20 ppm)	⟷	H of Me (1.32 ppm)
H^3 (5.78 ppm)	⟷	H^4 (4.62 ppm)
H^3 (5.78 ppm)	⟷	H of Me (1.32 ppm)
H^4 (4.62 ppm)	⟷	H of Me (1.32 ppm)
H^1 (5.20 ppm)	⟷	H^3 (5.78 ppm)

Strong
Medium
Weak

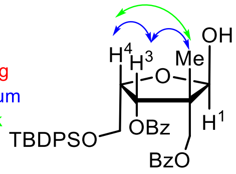

From 2D-NOESY

H^3 (5.81 ppm)	⟷	H^4 (4.67 ppm)
H^3 (5.81 ppm)	⟷	H of Me (1.39 ppm)
H^4 (4.67 ppm)	⟷	H of Me (1.39 ppm)

NOE confirmed α-L-anomer to be the major isomer for 44a,b

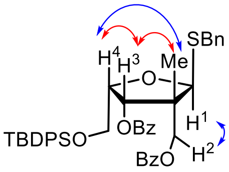

From 2D-NOESY

H^1 (5.25 ppm)	⟷	H^2 (4.17 ppm)
H^3 (5.79 ppm)	⟷	H^4 (4.69 ppm)
H^3 (5.79 ppm)	⟷	H of Me (1.38 ppm)
H^4 (4.69 ppm)	⟷	H of Me (1.38 ppm)

Strong
Medium

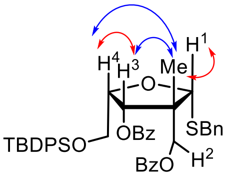

From 2D-NOESY

H^1 (4.97 ppm)	⟷	H of Me (1.36 ppm)
H^3 (5.87 ppm)	⟷	H^4 (4.69 ppm)
H^3 (5.87 ppm)	⟷	H of Me (1.36 ppm)
H^4 (4.69 ppm)	⟷	H of Me (1.36 ppm)

NOE confirmed β-D-anomer for 47a and α-D-anomer for 47b

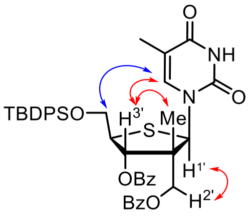

47a

From 2D-NOESY

$H^{1'}$ (6.57 ppm)	⟷	$H^{2'}$ (4.61 ppm)
$H^{3'}$ (5.65 ppm)	⟷	H of Me (1.09 ppm)
$H^{3'}$ (5.65 ppm)	⟷	H of Thy. (7.77 ppm)
$H^{5'}$ (3.90 ppm)	⟷	H of Thy. (7.77 ppm)

Strong
Medium

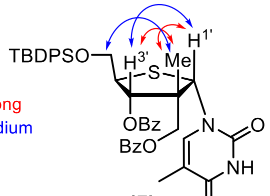

47b

From 2D-NOESY

$H^{1'}$ (6.28 ppm)	⟷	H of Me (1.41 ppm)
$H^{1'}$ (6.28 ppm)	⟷	$H^{3'}$ (5.42 ppm)
$H^{3'}$ (5.42 ppm)	⟷	H of Me (1.41) ppm
$H^{5'}$ (4.24 ppm)	⟷	H of Me (1.41) ppm

NOE confirmed β-L-anomer for 50

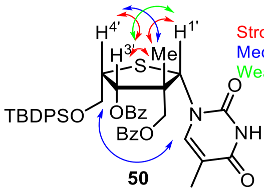

50

Strong
Medium
Weak

From 2D-NOESY

$H^{1'}$ (6.30 ppm)	⟷	H of Me (1.54 ppm)
$H^{3'}$ (5.84 ppm)	⟷	H of Me (1.54 ppm)
$H^{3'}$(5.84 ppm)	⟷	$H^{4'}$ (4.18 ppm)
$H^{4'}$ (4.18 ppm)	⟷	H of Me (1.54 ppm)
$H^{1'}$ (6.30 ppm)	⟷	$H^{3'}$ (5.84 ppm)
$H^{5'}$ (3.81 ppm)	⟷	H of Thy. (8.02 ppm)

NOE confirmed β-D-anomer for 61a and α-D-anomer for 61b

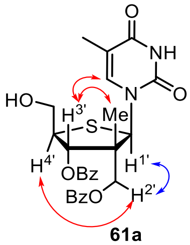

61a

From 2D-NOESY

$H^{1'}$ (6.54 ppm)	⟷	$H^{2'}$ (4.70 ppm)
$H^{3'}$ (5.66 ppm)	⟷	H of Thy. (8.60 ppm)
$H^{3'}$ (5.66 ppm)	⟷	H of Me (1.15 ppm)
$H^{4'}$ (3.67 ppm)	⟷	$H^{2'}$ (4.70 ppm)

Strong
Medium

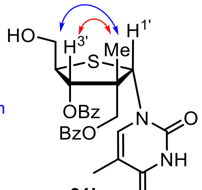

61b

From 2D-NOESY

$H^{3'}$ (5.52 ppm)	⟷	H of Me (1.55 ppm)
$H^{5'}$ (3.85 ppm)	⟷	H of Me (1.55 ppm)

NOE confirmed β-D-anomer for 23a and α-D-anomer for 23b

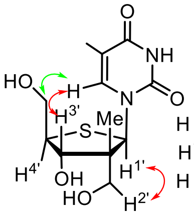

23a

From 2D-NOESY

H of Thy (8.44 ppm)	⟷	$H^{3'}$ (4.04 ppm)
$H^{1'}$ (6.17 ppm)	⟷	$H^{2'}$ (3.77 ppm)
H of Thy (8.44 ppm)	⟷	$H^{5'}$ (3.90 ppm)

Strong
Medium
Weak

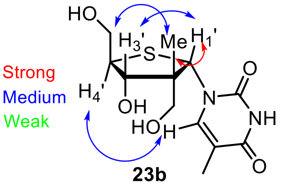

23b

From 2D-NOESY

H of Thy (8.16 ppm)	⟷	$H^{4'}$ (3.86 ppm)
$H^{1'}$ (6.01 ppm)	⟷	$H^{3'}$ (3.96 ppm)
$H^{1'}$ (6.01 ppm)	⟷	H of Me (1.23 ppm)
$H^{5'}$ (3.91 ppm)	⟷	H of Me (1.23 ppm)

NOE confirmed β-D-anomer for 62

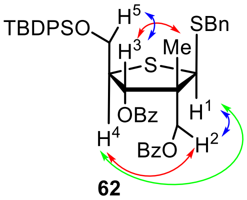

62

Strong
Medium
Weak

From 2D-NOESY

H^1 (4.41 ppm)	⟷	H^2 (4.55 ppm)
H^1 (4.41 ppm)	⟷	H^4 (3.65 ppm)
H^3 (5.70 ppm)	⟷	H of Me (1.29 ppm)
H^3 (5.70 ppm)	⟷	H^5 (4.03 ppm)
H^2 (4.55 ppm)	⟷	H^4 (3.65 ppm)

NOE confirmed β-D-anomer to be the major isomer for 63a,b

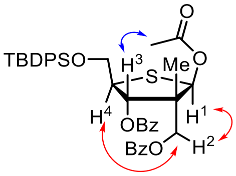

From 2D-NOESY

H^3 (5.84 ppm)	⟷	H of Ac (2.11 ppm)
H^1 (6.13 ppm)	⟷	H^2 (4.64 ppm)
H^4 (3.66 ppm)	⟷	H^2 (4.64 ppm)

Strong
Medium

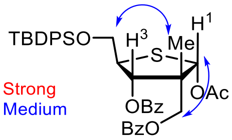

From 2D-NOESY

H^1 (6.06 ppm)	⟷	H^2 (4.49 ppm)
H^5 (4.09 ppm)	⟷	H of Me (1.31 ppm)

The N^9 regiochemistry of the purine ring of the following compounds was determined from HSQC and HMBC experiments. In addition, NOESY experiments confirmed the β- and α-stereochemistry.

NOE confirmed β-D-anomer for 64a

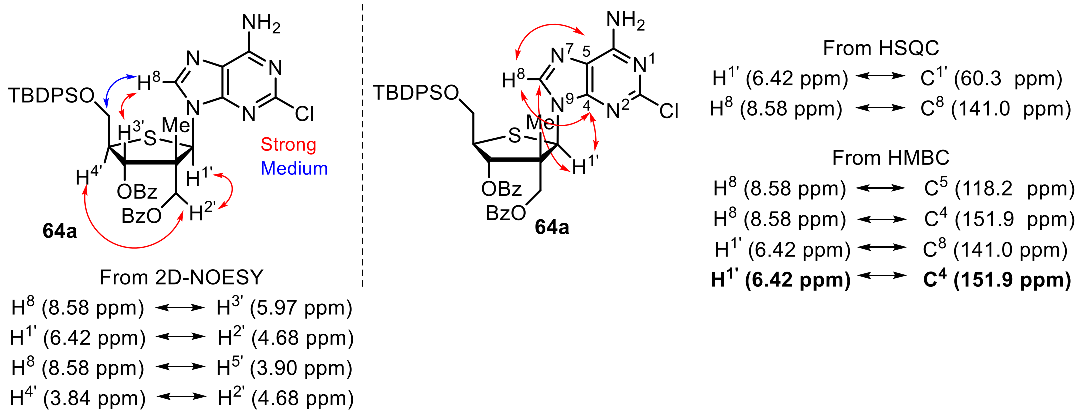

NOE confirmed α-D-anomer for 64b

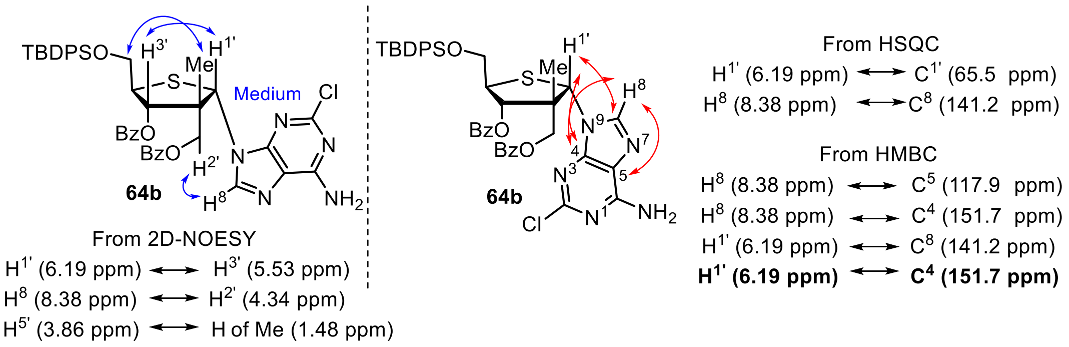

NOE confirmed β-D-anomer for 65a

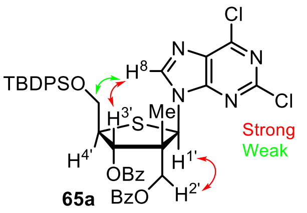

From 2D-NOESY

H$^{1'}$ (6.48 ppm) ⟷ H$^{2'}$ (4.71 ppm)
H^{8} (8.91 ppm) ⟷ H$^{3'}$ (5.98 ppm)
H^{8} (8.91 ppm) ⟷ H$^{5'}$ (4.00 ppm)

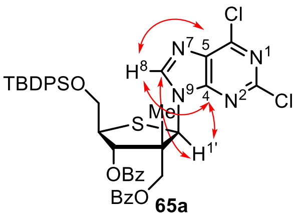

From HSQC

H$^{1'}$ (6.48 ppm) ⟷ C$^{1'}$ (61.1 ppm)
H^{8} (8.91 ppm) ⟷ C^{8} (145.9 ppm)

From HMBC

H^{8} (8.91 ppm) ⟷ C^{5} (131.1 ppm)
H^{8} (8.91 ppm) ⟷ C^{4} (153.42 ppm)
H$^{1'}$ (6.48 ppm) ⟷ C^{8} (145.9 ppm)
H$^{1'}$ (6.48 ppm) ⟷ C^{4} (153.42 ppm)

NOE confirmed α-D-anomer for 65b

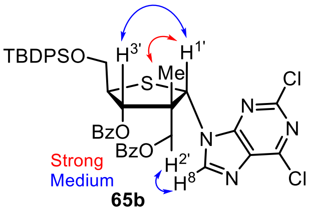

From 2D-NOESY

H$^{1'}$ (6.23 ppm) ⟷ H of Me 1.51ppm)
H$^{1'}$ (6.23 ppm) ⟷ H$^{3'}$ (5.56 ppm)
H^{8} (8.69 ppm) ⟷ H$^{2'}$ (4.30 ppm)

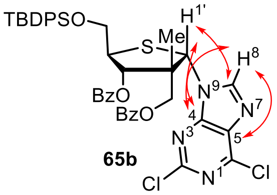

From HSQC

H$^{1'}$ (6.23 ppm) ⟷ C$^{1'}$ (66.4 ppm)
H^{8} (8.69 ppm) ⟷ C^{8} (146.2 ppm)

From HMBC

H^{8} (8.69 ppm) ⟷ C^{5} (130.8 ppm)
H^{8} (8.69 ppm) ⟷ C^{4} (153.3 ppm)
H$^{1'}$ (6.23 ppm) ⟷ C^{8} (146.2 ppm)
H$^{1'}$ (6.23 ppm) ⟷ C^{4} (153.3 ppm)

NOE confirmed β-D-anomer for 24a

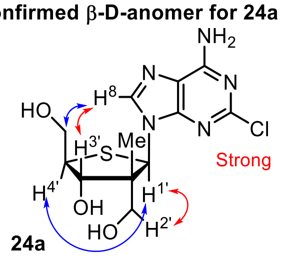

From 2D-NOESY

H$^{1'}$ (6.00 ppm) ⟷ H$^{2'}$ (3.84 ppm)
H^{8} (8.65 ppm) ⟷ H$^{3'}$ (4.32 ppm)
H^{8} (8.65 ppm) ⟷ H$^{5'}$ (4.05 ppm)
H$^{4'}$ (8.65 ppm) ⟷ H$^{1'}$ (6.00 ppm)

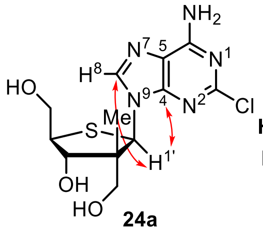

From HMBC

H^{1} (6.00 ppm) ⟷ C^{4} (152.3 ppm)
H^{1} (6.00 ppm) ⟷ C^{8} (143.2 ppm)

NOE confirmed α-D-anomer for 24b

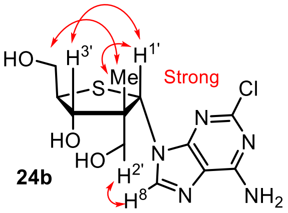

From 2D-NOESY

H$^{1'}$ (5.90 ppm) ⟷ H of Me (1.29 ppm)
H$^{1'}$ (5.90 ppm) ⟷ H$^{3'}$ (4.03 ppm)
H^{8} (8.56 ppm) ⟷ H$^{2'}$ (3.55 ppm)
H$^{5'}$ (3.67 ppm) ⟷ H of Me (1.29 ppm)

Part II. DFT Studies

Free Gibbs Energies (Ha) (1 Hartree = 627.5095 kcal mol–1) at 423.15K

Free energy profile data:

		Relative Gibbs Free Energies	
		(Ha)	(kcal mol-1)
**Data for Scheme 7:**			
**51**	**51** (–2362.966673)	0	0
**TS A1**	**TS A1** (–2362.914405) – **51** (–2362.966673)	0.052268	32.8
**52a** (X= MsO-)	**52a** (–2362.948353) – **51** (–2362.966673)	0.0183200	11.5
**53**	[**53** (–1428.686968) + **BnI** (–282.111711) + **NaOMs** (–825.948793)] – [**NaI** (–173.761283) + **51** (–2362.966673)	– 0.019516	–12.2
**TS A2** (C2'-endo)	**TS A2** (–2362.900222) – **51** (–2362.966673)	0.066451	41.7
**TS A2'** (C2'-exo) *not shown in Scheme, structure in SI below*	**TS A2'** (–2362.895187) – **51** (–2362.966673)	0.071486	44.9
**52b**	**52b** (–2362.935946) – **51** (–2362.966673)	0.030727	19.3
**52a'** (X= I-)	[**52a'** (–1710.758325) + **NaOMs** (–825.948793) – [**NaI** (–173.761283) + **51** (–2362.966673)]	0.020838	13.1
**TS B**	[**TS B** (–1710.758325) + **NaOMs** (–825.948793) – [**NaI** (–173.761283) + **51** (–2362.966673)]	0.04637300	29.1
**Data for Scheme 8a**:			
**54**	**54** (–2362.966364) – **51** (–2362.966673)	0.0003090	0.19
**TS C1**	**TS C1** (–2362.908045) – **51** (–2362.966673)	0.0586280	36.8
	ΔG_{act} = **TS C1** – **54**		36.6
**55a**	**55a** (–2362.937959) – **51** (–2362.966673)	0.0287140	18.0

**56**	[**56** (–1428.691312) + **BnI** (–282.111711) + **NaOMs** (–825.948793)] – [**NaI** (–173.761283) + **51** (–2362.966673)]	–0.0238600	–15.0
**TS C2**	**TS C2** (–2362.907431) – **51** (–2362.966673)	–0.0592420	37.2
	ΔG_{act} = **TS C2** – **54**		37.0
**55b**	**55a** (–2362.950435) – **51** (–2362.966673)	0.0162380	10.2
**Data for Scheme 8b:**			
**58**	**58** (–2362.966256) – **51** (–2362.966673)	0.0004170	0.26
**TS D**	**TS D** (–2362.914899) – **51** (–2362.966673)	0.0517740	32.5
	ΔG_{act} = **TS D**– **58**		32.2
**59**	**59** (–2362.952394) – **51** (–2362.966673)	0.0142790	9.0
**60**	[**56** (–1428.693878) + **BnI** (–282.111711) + **NaOMs** (–825.948793)] – [**NaI** (–173.761283) + **51** (–2362.966673)]	–0.0264260	–16.6

51

SCF energy: – 2363.37489440 hartree
Free energy correction: + 0.408221 hartree
imaginary frequency: none

C	-3.0345010	-0.1292220	0.5096560
C	-1.5453300	-0.5455980	0.4937150
C	-0.9497640	-1.3544620	-0.6833840
C	0.6010780	-1.2880440	-0.4987760
C	-4.1118280	-1.1925800	0.4069240
O	-4.2579640	-1.6407430	-0.9153030
C	-5.2367040	-2.6528500	-1.0194840
C	-1.3857570	-0.7491530	-2.0239640
C	-1.3693400	-2.8311810	-0.6135960
N	2.2150510	1.7346660	0.8152970
C	1.5850690	0.5146640	0.8266660
N	1.1352700	0.0830030	-0.4180010
C	1.3357450	0.8512080	-1.5444980
C	1.9546190	2.0455720	-1.5270730
C	2.4875770	2.5599430	-0.2790830
O	3.1225910	3.5893240	-0.1293320
O	1.4365590	-0.1324950	1.8508220
O	-1.3088630	-1.3042460	1.6878070
C	-1.1025250	-0.5991970	2.8271470
C	-0.6161860	-1.4946350	3.9281800
O	-1.2872170	0.5900960	2.9082190
H	-3.1740280	0.3608400	1.4797350
H	-0.9961340	0.3954850	0.5808170

H	0.8348360	-1.7366050	0.4689090
H	-5.0594510	-0.7594090	0.7591020
H	-3.8429470	-2.0177500	1.0858720
H	-5.3075640	-2.9273300	-2.0724610
H	-4.9546220	-3.5372370	-0.4314020
H	-6.2135290	-2.2933290	-0.6696720
H	-1.2211170	0.3318460	-2.0587930
H	-0.8334870	-1.2204830	-2.8456410
H	-2.4504780	-0.9325130	-2.1794010
H	-1.0397690	-3.3591940	-1.5110660
H	-0.9325880	-3.3269800	0.2574920
H	-2.4555860	-2.9099630	-0.5629630
H	2.5780870	2.0364930	1.7132990
H	0.9849040	0.4057940	-2.4662580
H	2.1012570	2.6190500	-2.4314130
H	-1.1865000	-2.4250340	3.9499940
H	0.4304420	-1.7343970	3.7201460
H	-0.6899870	-0.9755090	4.8825220
O	-1.4005000	2.4688010	-0.5337000
S	-2.8265280	2.3625590	-0.2579410
O	-3.3235450	2.7913660	1.0384820
O	-3.3099320	0.8433420	-0.5245000
C	-3.7468980	3.1184320	-1.5675750
H	-3.4388150	2.6626100	-2.5085420
H	-4.8086100	2.9635760	-1.3792590
H	-3.4987120	4.1805660	-1.5539800
S	1.4987600	-2.2509720	-1.7714630
C	3.0325140	-2.6355290	-0.8469650
H	2.7689820	-3.2604140	0.0109440
H	3.5978210	-3.2593980	-1.5469310
C	3.8572360	-1.4504950	-0.4036770
C	4.1833800	-1.2959280	0.9448540
C	4.3293650	-0.5095330	-1.3247040
C	4.9675690	-0.2230920	1.3684090
H	3.8094790	-2.0124480	1.6713830
C	5.1090200	0.5630780	-0.9048520
H	4.0659300	-0.6124130	-2.3749770
C	5.4318480	0.7090000	0.4449460
H	5.2066270	-0.1147310	2.4219240
H	5.4580580	1.2925940	-1.6295340
H	6.0333700	1.5512450	0.7723820

TS A1
SCF energy: – 2363.31723472 hartree
Free energy correction: + 0.402830 hartree
imaginary frequency: − 523.7i cm–1

C	-1.0364670	-0.2666200	0.8393350
C	-0.2704200	-1.4805040	0.3366710
C	1.2594480	-1.4700320	0.5685830
C	1.7752610	-0.2198340	-0.1834800
C	-1.3086640	-0.0218370	2.3089810
O	-1.1968920	-1.2409920	2.9898040
C	-1.3207950	-1.0738210	4.3858100
C	1.8674880	-2.7274630	-0.0646740
C	1.6097480	-1.4473730	2.0592430

N	5.3163700	-0.1931820	-1.0898710
C	3.9617100	-0.3882640	-1.2228550
N	3.2146210	-0.0069730	-0.1037580
C	3.8239650	0.5467600	1.0050170
C	5.1524610	0.7275580	1.1025400
C	6.0177970	0.3378800	0.0001390
O	7.2285610	0.4420810	-0.0392940
O	3.4575470	-0.8588450	-2.2244280
O	-0.4820330	-1.5080100	-1.0745680
C	-1.1389010	-2.5669690	-1.6125530
C	-1.3298710	-2.3695190	-3.0869900
O	-1.4920540	-3.5214650	-0.9672460
H	-1.5355740	0.3467710	0.1033040
H	-0.7073150	-2.3651690	0.8044820
H	1.5514740	-0.3426420	-1.2438750
H	-2.3203860	0.3947570	2.3923010
H	-0.6037510	0.7138510	2.7205790
H	-1.2411340	-2.0638400	4.8353890
H	-0.5213750	-0.4296510	4.7786340
H	-2.2916480	-0.6331020	4.6460450
H	2.9475480	-2.7510480	0.1106970
H	1.4319890	-3.6216480	0.3915370
H	1.6977200	-2.7590480	-1.1433410
H	2.6917770	-1.5490760	2.1859740
H	1.2883340	-0.5316600	2.5678900
H	1.1281870	-2.2862340	2.5660100
H	5.8711160	-0.4735290	-1.8926770
H	3.1506880	0.8449000	1.8003090
H	5.5983030	1.1699800	1.9819450
H	-0.3772590	-2.1124590	-3.5565710
H	-1.7348860	-3.2801220	-3.5259570
H	-2.0238410	-1.5370180	-3.2257130
O	-3.1807910	-0.3528680	-1.4841490
S	-3.8409240	-0.8460500	-0.2543610
O	-4.7791370	0.1062760	0.3559160
O	-2.8192130	-1.3644530	0.7361740
C	-4.7503000	-2.3088760	-0.7084950
H	-4.0426560	-3.0318550	-1.1167330
H	-5.2327850	-2.7094670	0.1834080
H	-5.4965280	-2.0280190	-1.4529170
S	0.8019490	1.2145720	0.4010700
C	0.1386280	1.8900180	-1.1771020
H	0.9924790	2.2383030	-1.7631000
H	-0.3594820	1.0681610	-1.7013080
C	-0.8192600	3.0006550	-0.8370370
C	-0.3332220	4.2471350	-0.4293510
C	-2.1981660	2.7794860	-0.8865940
C	-1.2164790	5.2622010	-0.0774640
H	0.7397900	4.4199020	-0.3910430
C	-3.0803030	3.7974550	-0.5279430
H	-2.5855130	1.8165590	-1.2159910
C	-2.5925790	5.0371670	-0.1234320
H	-0.8322600	6.2287760	0.2326470
H	-4.1495010	3.6144080	-0.5659050
H	-3.2812000	5.8289320	0.1542610

52a

SCF energy: – 2363.35291611 hartree
Free energy correction: + 0.404563 hartree
imaginary frequency: none

C	1.1959810	1.1959320	0.9098960
C	0.1466340	2.2303180	1.3386390
C	-1.2687060	1.6324790	1.2151030
C	-1.2483180	0.9843070	-0.1869940
C	1.8804340	0.3698790	1.9889030
O	2.7913940	-0.4402060	1.2973260
C	3.3199500	-1.4788320	2.0980110
C	-2.3568400	2.7090830	1.2432450
C	-1.5211680	0.6215520	2.3390080
N	-4.3178710	-0.1423070	-1.7746350
C	-3.2747600	0.6911550	-1.4616720
N	-2.3670140	0.1482830	-0.5434610
C	-2.5207600	-1.1403680	-0.0487490
C	-3.5450680	-1.9343010	-0.3983810
C	-4.5606660	-1.4489330	-1.3184640
O	-5.5424020	-2.0529730	-1.7040260
O	-3.1495960	1.8089070	-1.9262550
O	0.2893810	3.3152260	0.4126660
C	0.1786230	4.5777570	0.9072340
C	0.3219340	5.5892660	-0.1919220
O	-0.0163950	4.8088230	2.0725090
H	1.9821080	1.6885830	0.3296530
H	0.3375000	2.5910530	2.3531880
H	-1.1954770	1.7739040	-0.9396410
H	1.1732320	-0.2406270	2.5603460
H	2.3923170	1.0636440	2.6740270
H	3.9374230	-2.0959900	1.4429810
H	3.9413320	-1.0740500	2.9078280
H	2.5080710	-2.0813370	2.5206410
H	-3.3398200	2.2407340	1.1353410
H	-2.3324720	3.2379030	2.1999560
H	-2.2384570	3.4323530	0.4323070
H	-2.5605500	0.2811100	2.3048790
H	-0.8832820	-0.2668030	2.2910240
H	-1.3673000	1.1110860	3.3057730
H	-4.9932780	0.2402820	-2.4288510
H	-1.7479240	-1.4858150	0.6316990
H	-3.5987950	-2.9446420	-0.0178660
H	-0.5085560	5.4776400	-0.8947270
H	0.3140520	6.5906550	0.2343310
H	1.2502650	5.4151140	-0.7404170
O	0.1243220	-2.1958970	1.7698910
S	0.0482550	-3.3949240	0.8841730
O	0.7135410	-3.1907720	-0.4225790
O	-1.3316120	-3.9168580	0.7765660
C	0.9894450	-4.6601890	1.7259120
H	0.5530310	-4.8208140	2.7124930
H	0.9416360	-5.5777800	1.1381240
H	2.0242350	-4.3275360	1.8182800
S	0.3606010	0.0548860	-0.2744860

C	1.0301680	0.6271030	-1.8859360
H	0.3911420	0.1417870	-2.6294000
H	0.9009110	1.7112320	-1.9395170
C	2.4723870	0.2122050	-1.9880050
C	2.8271090	-1.1394160	-1.9212520
C	3.4607310	1.1879320	-2.1216050
C	4.1664770	-1.5030170	-2.0041700
H	2.0618790	-1.8982640	-1.7681580
C	4.8013230	0.8170460	-2.2086730
H	3.1819250	2.2378020	-2.1676340
C	5.1539540	-0.5279440	-2.1503320
H	4.4413810	-2.5515650	-1.9494940
H	5.5658060	1.5793100	-2.3187730
H	6.1979170	-0.8182450	-2.2142730

53
SCF energy: – 1428.94581238 hartree
Free energy correction: + 0.258845 hartree
imaginary frequency: none

C	-2.0515740	0.8261240	-0.4414740
C	-1.8235610	-0.4490230	0.3902520
C	-0.3501980	-0.5455770	0.8298830
C	0.4202240	-0.1392030	-0.4473410
C	-2.7064650	1.9707770	0.3144070
O	-2.8529210	3.0323920	-0.5937730
C	-3.4047690	4.1802160	0.0120910
C	0.0395030	-1.9653770	1.2495410
C	-0.0998770	0.4046310	2.0054930
N	4.0411610	-0.6965130	-0.4090810
C	2.7065780	-0.9528910	-0.6323350
N	1.8518300	0.0871780	-0.2591730
C	2.3519400	1.2578630	0.2723270
C	3.6630120	1.4754100	0.4845790
C	4.6303400	0.4485870	0.1392940
O	5.8381230	0.5122810	0.2790460
O	2.3140560	-2.0019660	-1.1067500
O	-2.1373450	-1.5463000	-0.4808560
C	-2.7993420	-2.5989840	0.0493340
C	-3.0113210	-3.6662770	-0.9868610
O	-3.1538790	-2.6520520	1.2015720
H	-2.7074200	0.5700950	-1.2765230
H	-2.4927010	-0.4878390	1.2552270
H	0.3487610	-0.9542340	-1.1666690
H	-2.1052830	2.2844560	1.1804620
H	-3.6900050	1.6396930	0.6867850
H	-3.4817530	4.9485610	-0.7581690
H	-4.4042950	3.9726040	0.4175550
H	-2.7636310	4.5452700	0.8258670
H	1.0877750	-1.9837290	1.5645340
H	-0.5761460	-2.2952310	2.0914590
H	-0.0724350	-2.6757010	0.4262930
H	0.9275310	0.3008920	2.3680080
H	-0.2643900	1.4538390	1.7466360
H	-0.7685380	0.1478780	2.8332110
H	4.6702330	-1.4478570	-0.6734950

H	1.6027950	2.0083640	0.4934730
H	4.0193440	2.4089840	0.8961780
H	-2.0407850	-4.0587820	-1.3030300
H	-3.6155550	-4.4675920	-0.5653340
H	-3.5004930	-3.2408710	-1.8658160
S	-0.4296970	1.3352530	-1.1454960

BnI

SCF energy: – 282.17852233 hartree
Free energy correction: + 0.066812 hartree
imaginary frequency: none

C	-3.0122570	1.2069310	-0.2667200
C	-1.7527240	1.2057950	0.3245910
C	-1.1143460	-0.0006270	0.6266810
C	-1.7533870	-1.2063600	0.3233700
C	-3.0129530	-1.2061680	-0.2679310
C	-3.6445120	0.0007020	-0.5650320
H	-3.5011810	2.1489240	-0.4936060
H	-1.2570930	2.1455740	0.5546600
H	-1.2583290	-2.1466710	0.5524960
H	-3.5024090	-2.1476600	-0.4957480
H	-4.6270860	0.0012050	-1.0259110
C	0.2310450	-0.0013080	1.2694010
H	0.4200810	-0.8951250	1.8610350
H	0.4200650	0.8913050	1.8628630
I	1.8426560	0.0001630	-0.2166410

NaOMs

SCF energy: – 825.95319932 hartree
Free energy correction: + 0.004406 hartree
imaginary frequency: none

O	-0.3993750	-0.0272300	-1.2166640
S	0.4577020	-0.1597820	-0.0000190
O	1.3226060	-1.3469700	0.0005210
C	1.5334320	1.2642470	0.0000500
H	0.9209770	2.1663870	-0.0004830
H	2.1554210	1.2280630	0.8950850
H	2.1560020	1.2274670	-0.8945630
O	-0.4000990	-0.0267270	1.2160980
Na	-2.3582980	0.1415030	0.0000300

NaI

SCF energy: – 173.72447705 hartree
Free energy correction: – 0.036806 hartree
imaginary frequency: none

Na	0.0000000	0.0000000	-2.4717370
I	0.0000000	0.0000000	0.5130020

TS A2 (C2'-endo)

SCF energy: – 2363.30296811 hartree
Free energy correction: + 0.402746 hartree

imaginary frequency: − 535.0i cm–1

C	-1.6308480	0.4636390	0.4531870
C	-1.5628040	-1.0363170	0.7090140
C	-0.1996730	-1.6182580	1.1721370
C	0.7848830	-1.2285360	0.0451690
C	-1.4834030	1.5508140	1.4844040
O	-1.1733450	2.7370230	0.7995280
C	-1.2666110	3.8702420	1.6432420
C	-0.3155100	-3.1483480	1.2189690
C	0.0980180	-1.0991330	2.5935550
N	4.0335940	-2.4432610	-1.0769670
C	2.7212570	-2.0390120	-1.1395360
N	2.1481770	-1.7197920	0.1036860
C	2.9006750	-1.7971520	1.2605500
C	4.1853670	-2.1945190	1.2852500
C	4.8574940	-2.5606350	0.0482910
O	6.0084970	-2.9342970	-0.0648500
O	2.1032020	-1.9607890	-2.1809450
O	-1.9170450	-1.6260140	-0.5382200
C	-3.0366320	-2.3923700	-0.5902670
C	-3.3648250	-2.7449170	-2.0095970
O	-3.6595210	-2.7152880	0.3900170
H	-1.8587250	0.7700530	-0.5595640
H	-2.3132910	-1.2956040	1.4593590
H	0.3736230	-1.6579450	-0.8707910
H	-0.7112890	1.3146540	2.2274290
H	-2.4359150	1.6352350	2.0190570
H	-1.0213500	4.7422350	1.0363230
H	-2.2828390	3.9782030	2.0403870
H	-0.5588310	3.7992840	2.4808610
H	0.6247440	-3.5865680	1.5670030
H	-1.1060570	-3.4436560	1.9156360
H	-0.5439640	-3.5650340	0.2347320
H	0.6744120	-1.8311390	3.1641390
H	0.6393350	-0.1512530	2.6166370
H	-0.8395160	-0.9576710	3.1409810
H	4.4514580	-2.6780510	-1.9722250
H	2.3870300	-1.5234520	2.1704510
H	4.7358520	-2.2526400	2.2134480
H	-2.4747280	-3.1121480	-2.5249710
H	-4.1542540	-3.4947860	-2.0263240
H	-3.6948270	-1.8277250	-2.5069580
O	-3.8420670	0.4830170	-1.7778110
S	-4.5249850	0.8082430	-0.5118740
O	-4.9866480	2.1948430	-0.3813020
O	-3.6790550	0.3720540	0.6754750
C	-5.9565520	-0.2471630	-0.3954080
H	-5.6249590	-1.2861460	-0.4146450
H	-6.4705970	-0.0324890	0.5420010
H	-6.6073880	-0.0292510	-1.2433520
S	0.6657660	0.5678750	-0.3566950
C	1.7612710	1.4285300	0.8410540
H	1.1308340	1.9368560	1.5757430
H	2.3710890	0.6793110	1.3509830
C	2.6314140	2.4083510	0.1026000

C	2.1185660	3.6506050	-0.2757150
C	3.9425640	2.0700490	-0.2358210
C	2.9148380	4.5514670	-0.9768270
H	1.0899190	3.8992400	-0.0260750
C	4.7401140	2.9729230	-0.9338380
H	4.3399220	1.0986980	0.0513780
C	4.2274100	4.2147010	-1.3040810
H	2.5111930	5.5161520	-1.2673970
H	5.7609780	2.7066710	-1.1886570
H	4.8490060	4.9182980	-1.8486380

TS A2’ (C2’-exo)

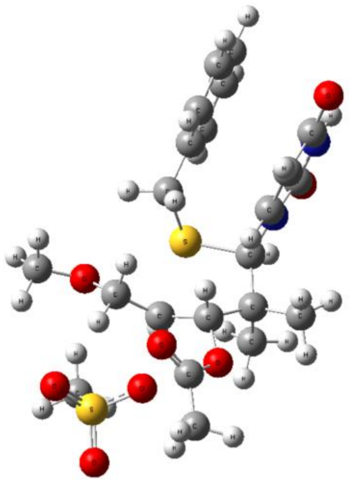

SCF energy: – 2363.30474595 hartree
Free energy correction: + 0.409559 hartree
imaginary frequency: − 525.8i cm–1

C	1.7473330	0.2412120	0.2083900
C	1.1730540	-1.1649260	-0.0803800
C	0.2756040	-1.6981470	1.0645460
C	-0.9838450	-0.8071500	1.2235720
C	1.8504060	1.2510450	-0.8961890
O	1.8299980	2.5304920	-0.3142300
C	2.2454380	3.5304810	-1.2314320
C	1.0511360	-1.6228920	2.3930030
C	-0.1067780	-3.1633650	0.8166660
N	-4.5175910	-1.1935770	0.4339790
C	-3.3895670	-0.9792590	1.1851910
N	-2.1804130	-1.1517460	0.4859440
C	-2.1880180	-1.5132710	-0.8483770
C	-3.3131540	-1.7216850	-1.5523490
C	-4.6104950	-1.5338860	-0.9190250
O	-5.6955130	-1.6459090	-1.4550370
O	-3.4272060	-0.6633810	2.3571140
O	2.1716720	-2.1455310	-0.2800650
C	2.8950740	-2.0196210	-1.4209360
C	4.0855190	-2.9265820	-1.4080120
O	2.5840660	-1.2507770	-2.2976570
H	2.0001320	0.5415940	1.2107730

H	0.6083710	-1.0680110	-1.0142280
H	-1.2894360	-0.8411330	2.2688640
H	1.0238090	1.1200600	-1.6116530
H	2.7801010	1.0768450	-1.4475010
H	2.2031550	4.4829360	-0.7022520
H	3.2686200	3.3329710	-1.5681810
H	1.5723400	3.5678910	-2.0982100
H	0.5442570	-2.2278920	3.1498570
H	2.0647550	-2.0086010	2.2540620
H	1.1227390	-0.6026050	2.7855840
H	-0.8998920	-3.4604550	1.5095520
H	-0.4519790	-3.3517490	-0.2031830
H	0.7625970	-3.7989420	0.9944720
H	-5.3925180	-1.0211520	0.9193180
H	-1.2169010	-1.6276600	-1.3091440
H	-3.2794760	-2.0078790	-2.5941350
H	4.8397890	-2.4505800	-0.7727230
H	3.8320800	-3.8968070	-0.9779130
H	4.4737860	-3.0389630	-2.4189960
O	4.8838390	1.4727580	-0.9802000
S	4.8957500	0.6860950	0.2644520
O	6.1442590	-0.0256800	0.5557010
O	3.7289080	-0.2902470	0.3319640
C	4.5970160	1.8363680	1.5929870
H	3.6619090	2.3599270	1.3808280
H	4.5342720	1.2832960	2.5310050
H	5.4262180	2.5443630	1.6244310
C	-1.3535320	1.5423970	-0.5045400
H	-0.8470070	2.4921320	-0.7169310
H	-1.1558560	0.8777200	-1.3513890
C	-2.8451600	1.7316740	-0.3279080
C	-3.4202630	2.1164540	0.8845290
C	-3.6693770	1.5352470	-1.4408100
C	-4.8004350	2.2742930	0.9874240
H	-2.7954520	2.2665110	1.7601040
C	-5.0476420	1.7058840	-1.3411570
H	-3.2289700	1.2383450	-2.3892980
C	-5.6174750	2.0659270	-0.1218710
H	-5.2363230	2.5565050	1.9404880
H	-5.6749220	1.5369720	-2.2104520
H	-6.6930280	2.1839800	-0.0367640
S	-0.4507720	0.9653220	0.9688920

52b

SCF energy: – 2363.34264812 hartree
Free energy correction: + 0.406702 hartree
imaginary frequency: none

C	-1.2906160	0.7381490	-0.2773890
C	-1.6013100	-0.6849900	0.2100080
C	-0.3817830	-1.2831070	0.9458510
C	0.7523660	-1.0607220	-0.0803840
C	-1.5683250	1.8995030	0.6593530
O	-1.3198380	3.0716990	-0.0801570
C	-1.7140820	4.2321940	0.6275200

C	-0.5529330	-2.7917470	1.1640430
C	-0.2726720	-0.5736120	2.3150960
N	4.3185710	-1.4855330	-0.5558820
C	2.9933640	-1.3350050	-0.8587190
N	2.1347250	-1.2659730	0.2541800
C	2.6325420	-1.4118910	1.5388850
C	3.9406520	-1.5713970	1.7968740
C	4.9100910	-1.5764010	0.7100410
O	6.1167420	-1.6519880	0.8216860
O	2.5663770	-1.2390280	-1.9924310
O	-1.8761990	-1.4229570	-0.9819100
C	-2.9866250	-2.2074210	-0.9599750
C	-3.4450390	-2.5271140	-2.3491980
O	-3.5057970	-2.5720170	0.0646820
H	-1.8782310	0.9131800	-1.1789320
H	-2.4903170	-0.6719790	0.8480210
H	0.5351760	-1.6828030	-0.9514830
H	-0.9582280	1.8868830	1.5731630
H	-2.6225360	1.8122900	0.9567680
H	-1.4913300	5.0883100	-0.0096100
H	-2.7886650	4.2053250	0.8445550
H	-1.1597980	4.3252330	1.5710720
H	0.3338970	-3.2120200	1.6490680
H	-1.4172400	-2.9684900	1.8089420
H	-0.7135240	-3.3182360	0.2204980
H	0.0954760	-1.2554760	3.0853220
H	0.3717760	0.3072790	2.3179710
H	-1.2686110	-0.2560520	2.6389460
H	4.9474150	-1.4714390	-1.3533260
H	1.9015020	-1.4010860	2.3315270
H	4.2990740	-1.6882650	2.8099630
H	-2.5960840	-2.7266860	-3.0052290
H	-4.1269600	-3.3759600	-2.3283920
H	-3.9678780	-1.6386890	-2.7167600
O	-4.1163210	0.3561260	-0.9574350
S	-5.0372570	0.5753410	0.1915630
O	-5.7909760	1.8416570	0.1016640
O	-4.3524130	0.3905550	1.4985420
C	-6.2443520	-0.7380600	0.0873470
H	-5.7173140	-1.6917160	0.1421540
H	-6.9444380	-0.6375530	0.9179020
H	-6.7742190	-0.6474990	-0.8621680
S	0.4680580	0.6485370	-0.8647200
C	1.4585840	1.8396810	0.1242300
H	1.0134070	2.8003930	-0.1526020
H	1.2873100	1.6762920	1.1879690
C	2.9250460	1.7446600	-0.2199880
C	3.3711390	1.7997310	-1.5439330
C	3.8577510	1.6440670	0.8155770
C	4.7329160	1.7390340	-1.8242670
H	2.6557680	1.8774210	-2.3584780
C	5.2216580	1.6052670	0.5341320
H	3.5165260	1.6015870	1.8467190
C	5.6602190	1.6431370	-0.7868710
H	5.0695500	1.7721380	-2.8552870
H	5.9369600	1.5235870	1.3461310

H	6.7215020	1.5963990	-1.0085400

52a'

SCF energy: – 1711.11273199 hartree
Free energy correction: + 0.354407 hartree
imaginary frequency: none

C	-1.3021630	0.9221300	-0.8833030
C	-0.3220210	1.9132990	-1.5273220
C	1.1304160	1.4530290	-1.2967980
C	1.1481840	1.1098130	0.2099440
C	-1.9566290	-0.1450440	-1.7478270
O	-2.8225390	-0.8200100	-0.8782280
C	-3.4807510	-1.9100740	-1.4967430
C	2.1429950	2.5724800	-1.5527380
C	1.4522900	0.2558500	-2.1952060
N	4.2982670	0.6390080	1.9561140
C	3.1673310	1.2744640	1.5131250
N	2.3264650	0.4680050	0.7308270
C	2.6328420	-0.8617280	0.4741210
C	3.7501260	-1.4481320	0.9330940
C	4.6997990	-0.6875790	1.7315520
O	5.7458420	-1.0943420	2.1955450
O	2.9123780	2.4372040	1.7617630
O	-0.5452360	3.1519450	-0.8439500
C	-0.5223680	4.2901260	-1.5909630
C	-0.7554470	5.4937320	-0.7261440
O	-0.3308350	4.2860710	-2.7790960
H	-2.1113620	1.4769300	-0.4004830
H	-0.5326080	2.0411730	-2.5926860
H	1.0182680	2.0322510	0.7802330
H	-1.2317420	-0.8367280	-2.1983550
H	-2.5065300	0.3639430	-2.5549680
H	-4.0959140	-2.3806130	-0.7290550
H	-4.1220700	-1.5609980	-2.3166900
H	-2.7532750	-2.6353900	-1.8793810
H	3.1557850	2.1997350	-1.3722750
H	2.0793770	2.9007960	-2.5936060
H	1.9775750	3.4309430	-0.8966340
H	2.5046040	-0.0245580	-2.0906810
H	0.8537770	-0.6368110	-1.9814920
H	1.2873670	0.5330290	-3.2408210
H	4.9240920	1.2123070	2.5134030
H	1.8987760	-1.4219180	-0.1027160
H	3.9544120	-2.4901370	0.7289910
H	0.0660690	5.5894030	-0.0106170
H	-0.8080340	6.3832920	-1.3509550
H	-1.6807310	5.3710110	-0.1587030
S	-0.3895910	0.1004740	0.4953530
C	-1.1176030	0.9138620	1.9833630
H	-0.4566920	0.6179250	2.8021500
H	-1.0552990	1.9934870	1.8261670
C	-2.5282780	0.4306500	2.1547940
C	-2.7801240	-0.8943450	2.5226900
C	-3.5940100	1.2944140	1.8972810

C	-4.0890060	-1.3488820	2.6297460
H	-1.9513020	-1.5704800	2.7177290
C	-4.9054050	0.8381840	2.0114190
H	-3.3999350	2.3277420	1.6201520
C	-5.1528330	-0.4832380	2.3721820
H	-4.2800190	-2.3783570	2.9136290
H	-5.7304550	1.5151270	1.8163040
H	-6.1740420	-0.8403350	2.4564650
I	0.2575340	-3.5123480	-1.1431170

TS B

SCF energy: – 1711.08638339 hartree
Free energy correction: + 0.353594 hartree
imaginary frequency: − 385.4i cm–1

C	-0.0395890	2.1288570	0.4165080
C	0.7151970	1.7517310	1.6974310
C	2.0832670	1.1391640	1.3467320
C	1.7310260	0.1201070	0.2367030
C	0.0492900	3.5610410	-0.0883560
O	-0.8124020	3.6212860	-1.1943650
C	-0.8244770	4.8951600	-1.8025730
C	2.7110020	0.4036260	2.5341850
C	3.0473250	2.2297860	0.8711670
N	4.3260690	-2.2011810	-0.8623490
C	3.2529840	-1.7251760	-0.1471780
N	2.8518230	-0.4308490	-0.5050660
C	3.5006210	0.2633900	-1.5097510
C	4.5498100	-0.2324730	-2.1875140
C	5.0487640	-1.5638450	-1.8785030
O	5.9859140	-2.1247260	-2.4123480
O	2.7050320	-2.3660310	0.7277730
O	-0.1025220	0.7462360	2.3147500
C	-0.2272720	0.7730900	3.6676940
C	-1.1009100	-0.3526660	4.1384260
O	0.3074150	1.6038060	4.3568040
H	-1.1044260	1.9406510	0.5790630
H	0.8156350	2.6030230	2.3767880
H	1.2377320	-0.7368860	0.6990530
H	1.0683680	3.8552570	-0.3729110
H	-0.2877580	4.2360990	0.7141840
H	-1.5193890	4.8427680	-2.6411610
H	-1.1629900	5.6656800	-1.0975810
H	0.1731630	5.1665590	-2.1722670
H	3.6773240	-0.0172390	2.2401980
H	2.8767100	1.0986400	3.3620710
H	2.0821910	-0.4199200	2.8815850
H	4.0376020	1.8042680	0.6839820
H	2.7194490	2.7291480	-0.0440200
H	3.1560120	2.9897360	1.6508200
H	4.6279960	-3.1369880	-0.6093100
H	3.0904310	1.2412630	-1.7309630
H	5.0298260	0.3321000	-2.9739860
H	-2.0894070	-0.2706630	3.6792080
H	-0.6699110	-1.3070910	3.8243850

H	-1.1859580	-0.3172300	5.2229310
S	0.4950700	0.9347920	-0.8690550
C	-1.2790330	-0.6168130	-0.6258340
H	-0.7824210	-1.2923800	-1.3089420
H	-1.0629290	-0.7056050	0.4328000
C	-2.3876170	0.2150550	-1.0776830
C	-2.6059550	0.4161030	-2.4476920
C	-3.2036490	0.8679180	-0.1417390
C	-3.6196540	1.2642290	-2.8726400
H	-1.9727140	-0.0916440	-3.1700240
C	-4.2194550	1.7127490	-0.5706250
H	-3.0495540	0.6828390	0.9187920
C	-4.4247980	1.9141840	-1.9354720
H	-3.7859380	1.4196930	-3.9332510
H	-4.8538000	2.2093910	0.1556570
H	-5.2179020	2.5746400	-2.2706800
I	-2.7400460	-3.0276310	0.0056550

54

SCF energy: – 2363.37282132 hartree
Free energy correction: + 0.406457`hartree
imaginary frequency: none

C	-3.1550960	-0.2303180	0.2261140
C	-1.6673560	-0.6432030	0.2929290
C	-0.8828300	-1.0513230	-0.9671730
C	0.6516210	-1.0875850	-0.6660140
C	-4.1570950	-1.1848060	-0.4008600
O	-4.1191170	-1.1044090	-1.8012650
C	-5.0261220	-2.0061880	-2.3996310
C	-1.0967210	-0.0004320	-2.0688890
C	-1.2882840	-2.4347390	-1.4946550
N	2.6488480	1.9713600	-1.0201850
C	1.9792400	0.8289990	-1.3923360
N	1.2402860	0.2325890	-0.3708320
C	1.2271030	0.7795470	0.8954280
C	1.8905560	1.9020200	1.2284630
C	2.7174780	2.5681860	0.2412910
O	3.4266860	3.5435000	0.4213680
O	2.0385790	0.3849830	-2.5258840
O	-1.6279430	-1.7444960	1.2166680
C	-1.4203720	-1.4617910	2.5238480
C	-1.3733060	-2.7190420	3.3412650
O	-1.2807120	-0.3360910	2.9399520
H	-3.4540740	-0.1091100	1.2751530
H	-1.1665120	0.2126840	0.7485180
H	1.1249270	-1.3889280	-1.6029340
H	-5.1618410	-0.9191550	-0.0409620
H	-3.9275050	-2.2030160	-0.0479540
H	-4.7719940	-3.0456490	-2.1498130
H	-6.0544830	-1.8050790	-2.0713870
H	-4.9561680	-1.8664250	-3.4787770
H	-0.4395770	-0.2185330	-2.9151220
H	-2.1346300	-0.0271440	-2.4042750
H	-0.8738840	1.0090290	-1.7086750

H	-2.2986110	-2.3932910	-1.9045020
H	-0.6101270	-2.7300800	-2.3030110
H	-1.2423890	-3.1972800	-0.7152620
H	3.2323850	2.3762880	-1.7445390
H	0.6589100	0.2284930	1.6354280
H	1.8545460	2.2977300	2.2335950
H	-1.3040390	-2.4652920	4.3974900
H	-2.2634270	-3.3221370	3.1496800
H	-0.5000470	-3.3041160	3.0381180
O	-1.4963870	2.5697750	0.3578220
S	-2.9360500	2.3667560	0.4144890
O	-3.5795940	2.2870080	1.7155860
O	-3.3204420	1.0421770	-0.4321760
C	-3.7537860	3.5399850	-0.6278610
H	-4.8208110	3.3206940	-0.6246760
H	-3.5559980	4.5269550	-0.2077480
H	-3.3329450	3.4562780	-1.6298380
S	1.1683250	-2.3302450	0.5793980
C	2.8668230	-2.6695360	-0.0187590
H	3.2268870	-3.4372460	0.6740940
H	2.7924970	-3.1304560	-1.0079160
C	3.8196770	-1.4989800	-0.0556230
C	4.4502940	-1.1534890	-1.2525150
C	4.1124130	-0.7646540	1.0982580
C	5.3587510	-0.0961700	-1.2982610
H	4.2200870	-1.7083190	-2.1582160
C	5.0158190	0.2920170	1.0550730
H	3.6125570	-1.0153170	2.0312790
C	5.6436920	0.6289060	-0.1448480
H	5.8361930	0.1623040	-2.2385390
H	5.2237170	0.8612750	1.9560880
H	6.3424470	1.4590320	-0.1784010

TS C1

SCF energy: – 2363.31248363 hartree
Free energy correction: + 0.404438 hartree
imaginary frequency: − 526.9i cm–1

C	0.6500100	-1.2666490	0.6302540
C	1.3537270	-0.0394730	1.1804070
C	0.4929200	1.0397550	1.8804660
C	-0.7167960	1.4545900	0.9924820
C	-0.0325580	-2.3443390	1.4478390
O	-1.0453150	-2.9822610	0.7196960
C	-0.5636660	-3.8359460	-0.3085260
C	1.3890700	2.2464540	2.1889850
C	-0.0715940	0.5133940	3.2097020
N	-0.5508480	4.8408460	-0.3722270
C	-0.8277300	3.8237020	0.5090910
N	-0.4670260	2.5523900	0.0569560
C	0.1017300	2.3761070	-1.1883190
C	0.3634640	3.3945490	-2.0279650
C	0.0384510	4.7570790	-1.6405910
O	0.2250420	5.7626070	-2.2986950
O	-1.3382990	4.0158620	1.5979460

O	1.9719900	0.5545150	0.0401030
C	3.3222660	0.6764770	0.0204870
C	3.7791410	1.1575460	-1.3243580
O	4.0212110	0.4189050	0.9678350
H	0.6827650	-1.3776260	-0.4455130
H	2.1252660	-0.3704910	1.8815660
H	-1.5068000	1.8256590	1.6475220
H	-0.4964150	-1.9295580	2.3434200
H	0.7391090	-3.0517360	1.7711150
H	-1.4352550	-4.3533750	-0.7118530
H	-0.0783460	-3.2743690	-1.1161390
H	0.1556400	-4.5599860	0.0896730
H	0.8043440	3.0228900	2.6893760
H	2.2025520	1.9407730	2.8537890
H	1.8268410	2.6732150	1.2831620
H	-0.4220710	1.3589730	3.8083780
H	-0.9233220	-0.1583630	3.0799090
H	0.6979960	-0.0116120	3.7834550
H	-0.8046870	5.7694370	-0.0498190
H	0.3353470	1.3533520	-1.4550720
H	0.8005920	3.2205160	-3.0012070
H	3.2169280	2.0503450	-1.6115470
H	4.8459530	1.3738110	-1.2941490
H	3.5649930	0.3688960	-2.0523800
O	2.4377920	-1.8299110	-1.8574340
S	2.9417790	-2.7309120	-0.8018830
O	2.6955340	-4.1602030	-1.0269580
O	2.4488100	-2.2724290	0.5634480
C	4.7077890	-2.5127260	-0.7025350
H	4.9249680	-1.4734430	-0.4545490
H	5.0906080	-3.1720520	0.0770940
H	5.1347360	-2.7814710	-1.6697450
S	-1.3769010	-0.0079960	0.1079720
C	-2.8314840	-0.5239370	1.1163300
H	-2.5062690	-1.2672460	1.8469360
H	-3.1822530	0.3707270	1.6371970
C	-3.8931670	-1.0910380	0.2119880
C	-4.0844610	-2.4709360	0.1267440
C	-4.6956520	-0.2359250	-0.5491490
C	-5.0738500	-2.9896520	-0.7066690
H	-3.4523030	-3.1315760	0.7122930
C	-5.6817310	-0.7543540	-1.3811130
H	-4.5457110	0.8394030	-0.4864180
C	-5.8720830	-2.1339890	-1.4611400
H	-5.2194530	-4.0637020	-0.7655360
H	-6.3034280	-0.0834420	-1.9653580
H	-6.6421770	-2.5389100	-2.1101260

55a

SCF energy: – 2363.34258345 hartree
Free energy correction: + 0.404624 hartree
imaginary frequency: none

C	0.7113570	-0.8068080	0.7155410

C	1.1050100	0.5604120	1.2844080
C	-0.1012230	1.2459580	1.9488510
C	-1.3225390	1.0256560	1.0103220
C	0.9493060	-2.0398680	1.5820250
O	0.5074440	-3.1993900	0.9201320
C	1.3961710	-3.6433420	-0.1005220
C	0.1544740	2.7374030	2.1742720
C	-0.4220740	0.5760690	3.2920430
N	-2.8307570	3.9529500	-0.5339370
C	-2.5983060	2.9648420	0.3881380
N	-1.6020240	2.0506560	0.0192160
C	-0.9256050	2.1763960	-1.1831290
C	-1.1760730	3.1635350	-2.0594570
C	-2.1934100	4.1617620	-1.7654440
O	-2.5100440	5.0996650	-2.4689420
O	-3.1959850	2.8858800	1.4450100
O	1.5017500	1.3392870	0.1595980
C	2.6952610	1.9844680	0.1951710
C	2.9676260	2.6236740	-1.1346720
O	3.3936610	2.0253130	1.1748730
H	1.2477170	-0.9268410	-0.2310490
H	1.9518130	0.4537250	1.9684250
H	-2.2358210	0.9471170	1.6021100
H	0.4285410	-1.9938510	2.5413770
H	2.0291860	-2.0642720	1.7663430
H	0.9900390	-4.5839800	-0.4752010
H	1.4665470	-2.9263560	-0.9270380
H	2.4023800	-3.7998370	0.3010570
H	-0.7217250	3.2041140	2.6333350
H	1.0072010	2.8593880	2.8484560
H	0.3777830	3.2572670	1.2394350
H	-1.2044320	1.1415030	3.8062800
H	-0.7803580	-0.4519640	3.1836070
H	0.4653290	0.5577910	3.9306260
H	-3.5468460	4.6243450	-0.2736450
H	-0.1676840	1.4283730	-1.3780550
H	-0.6406910	3.2287220	-2.9962250
H	2.1333240	3.2772670	-1.4062730
H	3.8918430	3.1978970	-1.0855790
H	3.0400150	1.8270440	-1.8810930
O	2.8532660	-0.5943800	-1.7147560
S	3.9319650	-1.1802240	-0.8733100
O	4.3493640	-2.5267660	-1.3115920
O	3.6125750	-1.0963170	0.5751270
C	5.3565940	-0.1218360	-1.0982660
H	5.1267280	0.8678930	-0.7000080
H	6.1947110	-0.5530400	-0.5490660
H	5.5929750	-0.0629640	-2.1615380
S	-1.0726480	-0.6188130	0.1825130
C	-2.1555200	-1.7854550	1.1573370
H	-1.5062250	-2.3627250	1.8138890

H	-2.8088950	-1.1411710	1.7490370
C	-2.9204780	-2.6598450	0.2073600
C	-2.4623020	-3.9464130	-0.0838930
C	-4.0902400	-2.1841090	-0.3910990
C	-3.1776280	-4.7536070	-0.9646820
H	-1.5510490	-4.3052250	0.3849700
C	-4.8000170	-2.9915320	-1.2734400
H	-4.4481020	-1.1834810	-0.1606440
C	-4.3433770	-4.2773390	-1.5605920
H	-2.8234640	-5.7553860	-1.1850100
H	-5.7101940	-2.6201160	-1.7327150
H	-4.8985400	-4.9083370	-2.2472350

56

SCF energy: – 1428.95036069 hartree
Free energy correction: + 0.259049 hartree
imaginary frequency: none

C	1.9218940	0.0529620	-0.3692540
C	1.2002120	0.4248910	0.9386360
C	0.3246990	-0.7445680	1.4231530
C	-0.2764090	-1.4028360	0.1516840
C	3.3997830	-0.2488950	-0.1939270
O	3.9097330	-0.5821820	-1.4598410
C	5.2753780	-0.9311530	-1.4082800
C	-0.7492430	-0.2786320	2.4095850
C	1.2135610	-1.7851210	2.1211930
N	-3.8658420	-0.7389480	-0.2217060
C	-2.7085550	-1.3710480	0.1755380
N	-1.5380930	-0.7905780	-0.3082860
C	-1.5849400	0.3140430	-1.1255840
C	-2.7315070	0.9186220	-1.4931520
C	-4.0033640	0.3966310	-1.0287670
O	-5.1090840	0.8420160	-1.2798500
O	-2.7158380	-2.3534840	0.8960870
O	0.3373390	1.5474650	0.7055640
C	0.9408750	2.7490100	0.5837300
C	-0.0714870	3.8410370	0.3876810
O	2.1396330	2.8855060	0.6377570
H	1.8489600	0.8887040	-1.0701130
H	1.9267080	0.7100050	1.7076910
H	-0.5311590	-2.4411960	0.3634470
H	3.5589960	-1.0743660	0.5168290
H	3.8943400	0.6511240	0.2052300
H	5.5877620	-1.1718880	-2.4251790
H	5.8827720	-0.0977400	-1.0303120
H	5.4361030	-1.8053350	-0.7629240
H	-1.3328930	-1.1357360	2.7548880
H	-0.2711070	0.1876770	3.2770980
H	-1.4259900	0.4529580	1.9636520
H	0.5900920	-2.5976600	2.5062340
H	1.9533390	-2.2220600	1.4459440
H	1.7370600	-1.3295240	2.9672010

H	-4.7231610	-1.1549310	0.1274870
H	-0.6216940	0.6640100	-1.4728640
H	-2.7297870	1.7787690	-2.1477460
H	-0.6609970	3.9537940	1.3016550
H	0.4381450	4.7753190	0.1589960
H	-0.7581490	3.5713090	-0.4184620
S	1.0392850	-1.3887990	-1.1056230

TS C2

SCF energy: – 2363.30766454 hartree
Free energy correction: + 0.400234 hartree
imaginary frequency: − 541.1i cm–1

C	-0.1093760	-1.2523040	0.8602400
C	1.2047870	-0.7632580	1.4614870
C	1.1698000	0.6263430	2.1604240
C	0.3669780	1.6588450	1.3252720
C	-1.1911740	-1.9665670	1.6332860
O	-2.3472900	-1.8932180	0.8451240
C	-3.4682880	-2.4882740	1.4654060
C	2.6029190	1.1314090	2.3649190
C	0.5074720	0.5000980	3.5427950
N	2.1478170	3.9777850	-0.8629120
C	1.5058680	3.5039500	0.2555190
N	1.0190480	2.1925890	0.1367400
C	1.2182680	1.4739000	-1.0309720
C	1.8592130	1.9693850	-2.1039490
C	2.3903890	3.3208090	-2.0763240
O	2.9892680	3.8857410	-2.9711260
O	1.3789120	4.1687730	1.2659630
O	2.1323000	-0.6790210	0.3897430
C	3.2477250	-1.4442190	0.4184660
C	4.0220430	-1.2413080	-0.8530150
O	3.5551720	-2.1446420	1.3484770
H	-0.2733650	-1.1077030	-0.1986030
H	1.5556920	-1.5094920	2.1818270
H	0.1911420	2.5294980	1.9566620
H	-1.3512030	-1.5035570	2.6168890
H	-0.8789930	-3.0040710	1.7952140
H	-4.3135280	-2.3471520	0.7904470
H	-3.3046830	-3.5602280	1.6314310
H	-3.6873800	-2.0041450	2.4262970
H	2.5862890	2.0728520	2.9214270
H	3.1771800	0.3976050	2.9394390
H	3.1142100	1.2999510	1.4144950
H	0.5427520	1.4649130	4.0570860
H	-0.5389210	0.1875090	3.5051620
H	1.0549670	-0.2269130	4.1494230
H	2.5041590	4.9248520	-0.7806490
H	0.8398450	0.4606010	-1.0444570
H	1.9838700	1.3754290	-2.9985780
H	4.3888230	-0.2106400	-0.8866130
H	4.8638260	-1.9316240	-0.8895940
H	3.3523350	-1.3837440	-1.7058690
O	0.8270170	-1.9528700	-1.9743770

S	0.6463610	-3.2242140	-1.2419310
O	-0.5571960	-3.9823370	-1.5982620
O	0.7630650	-2.9946580	0.2604720
C	2.0503220	-4.2623430	-1.5958320
H	2.9582510	-3.7651730	-1.2537040
H	1.9177260	-5.2075600	-1.0689740
H	2.0855230	-4.4285620	-2.6733990
S	-1.2696230	0.8620080	1.0382860
C	-1.8201810	1.3124520	-0.6543190
H	-1.5069960	2.3442460	-0.8346150
H	-1.3322140	0.6564620	-1.3797990
C	-3.3168140	1.1607020	-0.7208320
C	-4.1505810	2.2196830	-0.3545440
C	-3.8791340	-0.0477240	-1.1372860
C	-5.5335120	2.0760110	-0.4126530
H	-3.7128190	3.1596070	-0.0274290
C	-5.2636260	-0.1906220	-1.1951050
H	-3.2304600	-0.8770170	-1.4064610
C	-6.0918470	0.8698870	-0.8333840
H	-6.1747850	2.9058240	-0.1330960
H	-5.6941350	-1.1305290	-1.5266480
H	-7.1704590	0.7581880	-0.8814680

55b

SCF energy: – 2363.35492938 hartree
Free energy correction: + 0.404494 hartree
imaginary frequency: none

C	0.3076340	0.6524900	1.3191440
C	-1.0126760	1.2748840	1.8107960
C	-2.0542920	0.1775630	2.1467590
C	-1.7269580	-1.1204380	1.3640110
C	1.5674230	1.1038260	2.0261710
O	2.5918730	0.3027110	1.5005490
C	3.8869700	0.8320860	1.6962850
C	-3.4903430	0.6249550	1.8771860
C	-1.8901940	-0.1524270	3.6429200
N	-3.5239450	-2.5461000	-1.4584570
C	-2.9886510	-2.4610940	-0.1990020
N	-2.2692690	-1.2763610	0.0430600
C	-2.0861730	-0.3333310	-0.9606200
C	-2.6043310	-0.4701690	-2.1909530
C	-3.4079400	-1.6370940	-2.5214630
O	-3.9459840	-1.8692980	-3.5850090
O	-3.1237650	-3.3291210	0.6402810
O	-1.4911190	2.1390210	0.7861610
C	-1.0327110	3.4234340	0.8187400
C	-1.5288070	4.1940580	-0.3673360
O	-0.3764350	3.8557460	1.7305820
H	0.4261390	0.8649700	0.2532190
H	-0.8310690	1.8801770	2.7028360
H	-2.0597330	-1.9936040	1.9246870
H	1.4922840	0.9934650	3.1197570
H	1.7060900	2.1646230	1.7782320
H	4.5862540	0.1185760	1.2575310

H	3.9857290	1.7999480	1.1896690
H	4.1081130	0.9545590	2.7644250
H	-4.1882780	-0.1667880	2.1660910
H	-3.7101050	1.5132080	2.4762560
H	-3.6537520	0.8762060	0.8277050
H	-2.5492400	-0.9761860	3.9300060
H	-0.8622620	-0.4325100	3.9028370
H	-2.1537760	0.7241010	4.2402000
H	-4.0555810	-3.3915660	-1.6420350
H	-1.4748160	0.5260840	-0.7099090
H	-2.4170600	0.2719400	-2.9542260
H	-2.5082310	4.6215170	-0.1310060
H	-0.8271280	4.9986020	-0.5827780
H	-1.6318350	3.5342430	-1.2294570
O	0.4674460	0.9342540	-1.8985660
S	1.4260550	2.0778590	-1.8340050
O	2.8267490	1.6726760	-2.0657810
O	1.2304610	2.8875510	-0.6062970
C	0.9833800	3.1369280	-3.2035240
H	-0.0523630	3.4568070	-3.0835990
H	1.6472820	4.0023690	-3.2018680
H	1.0995930	2.5733730	-4.1299870
S	0.1497970	-1.1824000	1.4305980
C	0.6365810	-1.8323900	-0.2303710
H	-0.0468550	-2.6743970	-0.3800420
H	0.4409470	-1.0384380	-0.9586490
C	2.0741690	-2.2655410	-0.2417980
C	2.4573670	-3.4728140	0.3465740
C	3.0260020	-1.4558630	-0.8638570
C	3.7900930	-3.8714380	0.3152210
H	1.7112560	-4.1033430	0.8250030
C	4.3576070	-1.8627060	-0.8995090
H	2.7290360	-0.5087650	-1.3094470
C	4.7415010	-3.0656590	-0.3092540
H	4.0844820	-4.8112900	0.7711510
H	5.0952830	-1.2336140	-1.3881840
H	5.7807480	-3.3782690	-0.3382040

58

SCF energy: – 2363.37590039 hartree
Free energy correction: + 0.409645 hartree
imaginary frequency: none

C	-3.0217080	-0.3876530	-0.1391600
C	-1.5024370	-0.4126390	0.0979440
C	-0.7609030	-1.7580110	-0.0990050
C	0.7272200	-1.4127030	-0.4470960
C	-3.8993050	-1.1611920	0.8428270
O	-4.0248570	-2.4856790	0.3881160
C	-4.7711350	-3.2802210	1.2868610
C	-1.3247990	-2.4739640	-1.3376200
C	-0.9043350	-2.6671940	1.1289610
N	2.2627880	1.8607510	0.1487580
C	1.5719540	0.8790250	-0.5198680
N	1.2843320	-0.2494520	0.2522320

C	1.6305390	-0.3070910	1.5812330
C	2.2420410	0.7030260	2.2279970
C	2.6344890	1.8946290	1.4915470
O	3.2394270	2.8513850	1.9436880
O	1.2522360	0.9813620	-1.6899620
O	-1.3094230	0.0442290	1.4449010
C	-0.9697540	1.3398640	1.6410030
C	-0.9862070	1.6794930	3.1044800
O	-0.7035240	2.1042530	0.7467470
H	-3.2520660	-0.7130000	-1.1582390
H	-1.0785860	0.3248510	-0.5912160
H	0.7476190	-1.1200000	-1.4984730
H	-4.8846050	-0.6746830	0.8757300
H	-3.4657250	-1.1239230	1.8530000
H	-4.8369010	-4.2797930	0.8563580
H	-5.7817170	-2.8736200	1.4235410
H	-4.2767290	-3.3366670	2.2661850
H	-2.3273800	-2.8567640	-1.1368080
H	-1.3671890	-1.7952440	-2.1978760
H	-0.6800860	-3.3170910	-1.6016820
H	-0.3785840	-3.6127400	0.9653830
H	-0.5230870	-2.2037440	2.0411740
H	-1.9589200	-2.9068830	1.2825930
H	2.5000440	2.6732450	-0.4102860
H	1.3836160	-1.2388060	2.0758920
H	2.4981240	0.6323130	3.2759880
H	-0.3985750	2.5814400	3.2722630
H	-2.0224180	1.8628000	3.4057020
H	-0.5977030	0.8501230	3.6972930
O	-2.4674630	1.4882130	-2.2187900
S	-3.4470970	1.9621110	-1.2566190
O	-3.3815880	3.3069970	-0.7289270
O	-3.4236980	0.9964740	0.0392300
C	-5.0778430	1.6524680	-1.8892130
H	-5.2066930	2.2747680	-2.7758320
H	-5.1592300	0.5972590	-2.1540940
H	-5.7992090	1.9222560	-1.1182610
S	1.8304290	-2.8648080	-0.2758840
C	3.1603740	-2.3847580	-1.4379460
H	3.8079670	-3.2677570	-1.4552100
H	2.7249700	-2.2812460	-2.4358680
C	3.9595710	-1.1519050	-1.0812170
C	4.5499440	-1.0022870	0.1775490
C	4.1520170	-0.1534810	-2.0383580
C	5.3152210	0.1219080	0.4719710
H	4.3922940	-1.7663590	0.9353120
C	4.9234300	0.9712450	-1.7479630
H	3.6834310	-0.2504120	-3.0139540
C	5.5066970	1.1122280	-0.4919180
H	5.7576650	0.2295740	1.4578200
H	5.0587260	1.7397690	-2.5028790
H	6.0984730	1.9918140	-0.2585120

TS C2

SCF energy: − 2363.31768046 hartree

Free energy correction: + 0.402781 hartree
imaginary frequency: − 558.1i cm–1

C	0.8964290	-0.8750120	0.3578970
C	-0.4010730	-1.6363000	0.2074390
C	-1.2124020	-1.2150080	-1.0269890
C	-1.5108640	0.3245150	-0.9664330
C	1.4709760	-0.6137980	1.7223610
O	2.5388840	0.2752110	1.5487040
C	3.0787500	0.7327770	2.7706370
C	-2.5107490	-2.0272760	-1.0941270
C	-0.3885420	-1.5088690	-2.2902900
N	-5.0677520	1.1507470	-1.1210800
C	-3.7977410	0.8560510	-1.5576410
N	-2.8633660	0.6797970	-0.5360610
C	-3.2327760	0.7635480	0.7897860
C	-4.4898790	1.0327400	1.1871430
C	-5.5314800	1.2546530	0.1963510
O	-6.6994480	1.5085370	0.4201660
O	-3.5110910	0.7498230	-2.7360100
O	-1.1748610	-1.3983380	1.3917290
C	-1.7225790	-2.4785710	2.0184520
C	-2.5514830	-2.0384070	3.1899610
O	-1.5585670	-3.6085130	1.6419720
H	1.4371480	-0.5073160	-0.5005460
H	-0.1659510	-2.7011150	0.1468560
H	-1.4254960	0.7294730	-1.9762060
H	1.7971780	-1.5731920	2.1491610
H	0.7050820	-0.1927060	2.3862350
H	3.8585860	1.4527840	2.5177310
H	2.3088910	1.2285520	3.3764320
H	3.5112310	-0.0946300	3.3484360
H	-3.0792930	-1.7477890	-1.9857010
H	-2.2715510	-3.0923470	-1.1582700
H	-3.1481750	-1.8730370	-0.2184350
H	-0.9857820	-1.2646630	-3.1738750
H	0.5494300	-0.9493070	-2.3413590
H	-0.1305520	-2.5716100	-2.3306650
H	-5.7552750	1.2825750	-1.8564570
H	-2.4347810	0.5936410	1.5028190
H	-4.7468000	1.1028740	2.2348500
H	-2.8408330	-2.9081920	3.7769450
H	-1.9928780	-1.3286080	3.8038630
H	-3.4497070	-1.5332920	2.8199390
O	2.9744990	-1.0206350	-1.7986350
S	3.2287210	-2.2379800	-0.9954310
O	3.5617810	-3.4409900	-1.7602220
O	2.0694200	-2.4979630	-0.0414670
C	4.6068390	-1.8605550	0.0661930
H	4.3299930	-0.9887980	0.6625500
H	4.8024520	-2.7236570	0.7035400
H	5.4734520	-1.6455990	-0.5604130
S	-0.2664300	1.2051980	0.0592740
C	0.7972150	1.9557350	-1.2434210
H	1.1811710	1.1554710	-1.8828700
H	0.1496340	2.6114410	-1.8309880

C	1.9259080	2.7088510	-0.5933270
C	3.2170940	2.1777120	-0.6092880
C	1.6883510	3.9328880	0.0372110
C	4.2633010	2.8745610	-0.0095230
H	3.3934360	1.2133270	-1.0811050
C	2.7333620	4.6239330	0.6432990
H	0.6822830	4.3459340	0.0489160
C	4.0235180	4.0957270	0.6183300
H	5.2670970	2.4608340	-0.0303390
H	2.5427440	5.5762020	1.1279570
H	4.8404770	4.6370140	1.0854800

59

SCF energy: – 2363.35474492 hartree
Free energy correction: + 0.402351 hartree
imaginary frequency: none

C	0.5931520	-0.7010880	0.4827980
C	-0.4686250	-1.7693130	0.2810690
C	-1.2842810	-1.4168030	-0.9770200
C	-1.5355540	0.1307240	-0.9724460
C	1.2666560	-0.6680180	1.8375320
O	2.1048950	0.4585200	1.8140780
C	2.7935170	0.6768540	3.0282660
C	-2.6016080	-2.1889390	-1.0565890
C	-0.4309360	-1.7460810	-2.2135730
N	-4.8095380	1.7101320	-1.2378860
C	-3.5616000	1.2866490	-1.6231030
N	-2.8515360	0.6104800	-0.6196420
C	-3.3862270	0.4368540	0.6461010
C	-4.6089480	0.8724810	0.9889260
C	-5.4343480	1.5650260	0.0081840
O	-6.5531940	2.0008620	0.1894470
O	-3.1092830	1.4742690	-2.7346500
O	-1.3026600	-1.7770660	1.4471710
C	-1.7463690	-2.9870270	1.8929740
C	-2.6475340	-2.8148590	3.0812020
O	-1.4492600	-4.0256730	1.3653190
H	1.3479620	-0.7810710	-0.3010180
H	0.0261930	-2.7380340	0.1732920
H	-1.3409640	0.5350900	-1.9675200
H	1.8397780	-1.6016680	1.9438530
H	0.5345880	-0.5996200	2.6539510
H	3.3943730	1.5766910	2.8872200
H	2.0903210	0.8275060	3.8573220
H	3.4499460	-0.1707230	3.2640430
H	-3.1514100	-1.8986530	-1.9571820
H	-2.3865760	-3.2590740	-1.1126000
H	-3.2487920	-2.0150880	-0.1925090
H	-0.9853440	-1.4850500	-3.1200410
H	0.5325450	-1.2262640	-2.2251640
H	-0.2167780	-2.8181260	-2.2360700
H	-5.3401850	2.1923420	-1.9569620
H	-2.7413620	-0.0849720	1.3451980
H	-5.0015220	0.7252880	1.9852630

H	-2.8393310	-3.7865850	3.5327130
H	-2.1953660	-2.1360140	3.8068680
H	-3.5948500	-2.3768510	2.7499160
O	2.7822920	-0.7093510	-1.8625980
S	3.4414420	-1.9040410	-1.2512110
O	4.2763000	-2.6586270	-2.2000240
O	2.4701080	-2.7356890	-0.4969920
C	4.5446160	-1.2244660	-0.0174790
H	3.9525270	-0.6107520	0.6672820
H	5.0257830	-2.0451950	0.5165200
H	5.2956140	-0.6092400	-0.5168710
S	-0.2647130	0.8969100	0.1587130
C	0.8263530	1.7796170	-1.0526630
H	1.2583830	1.0107470	-1.7003680
H	0.1222950	2.4072330	-1.6072270
C	1.8796700	2.5826820	-0.3464560
C	3.2122040	2.1744440	-0.4187530
C	1.5373010	3.7248250	0.3808140
C	4.1980360	2.9102240	0.2338890
H	3.4588100	1.2790930	-0.9839950
C	2.5234000	4.4536350	1.0381280
H	0.4992640	4.0457220	0.4321960
C	3.8554680	4.0463510	0.9650800
H	5.2347590	2.5932120	0.1732180
H	2.2544830	5.3415800	1.6011650
H	4.6250630	4.6176800	1.4748690

60

SCF energy: – 1428.95319126 hartree
Free energy correction: + 0.259313 hartree
imaginary frequency: none

C	1.9911180	-1.1300630	-0.7107660
C	1.4493160	-0.6311650	0.6298900
C	0.2035460	-1.4524750	0.9881760
C	-0.6467460	-1.5471970	-0.3138560
C	2.8353750	-0.0906620	-1.4324500
O	3.8618650	0.2836140	-0.5459650
C	4.6774030	1.3022290	-1.0816300
C	-0.5678450	-0.8215210	2.1484340
C	0.6322520	-2.8751670	1.3749640
N	-3.8433960	0.2296440	-0.0618040
C	-2.9274880	-0.7940090	0.0334630
N	-1.6633950	-0.4892480	-0.4697380
C	-1.3999390	0.7475690	-1.0109730
C	-2.3130790	1.7366830	-1.0785330
C	-3.6565850	1.5182690	-0.5755930
O	-4.5737570	2.3198990	-0.5743750
O	-3.2054550	-1.8734030	0.5257310
O	1.0829240	0.7421560	0.4609450
C	1.4768200	1.6317140	1.4000190
C	1.0634090	3.0204360	1.0006410
O	2.0647930	1.3250870	2.4061750
H	2.6012480	-2.0189340	-0.5307470
H	2.2132060	-0.6962930	1.4073990
H	-1.2189060	-2.4738290	-0.3019020
H	2.2176200	0.7788710	-1.6993360

H	3.2516590	-0.5111070	-2.3605250
H	5.4403950	1.5330820	-0.3372540
H	5.1636230	0.9746250	-2.0104430
H	4.0909430	2.2073240	-1.2925800
H	-1.4431810	-1.4277390	2.3957970
H	0.0798210	-0.7649060	3.0286460
H	-0.9052770	0.1913940	1.9092910
H	-0.2454120	-3.4522260	1.6810710
H	1.1101470	-3.4042730	0.5449150
H	1.3314650	-2.8460760	2.2160520
H	-4.7655030	0.0146710	0.3036800
H	-0.3951900	0.8696690	-1.3922240
H	-2.0733140	2.6926210	-1.5227290
H	-0.0110570	3.0379850	0.7974930
H	1.3109270	3.7212440	1.7959130
H	1.5816520	3.3018350	0.0794390
S	0.5290220	-1.6235850	-1.7126220

^{1}H-NMR (500 MHz, $CDCl_3$)

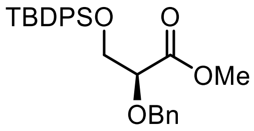

S2

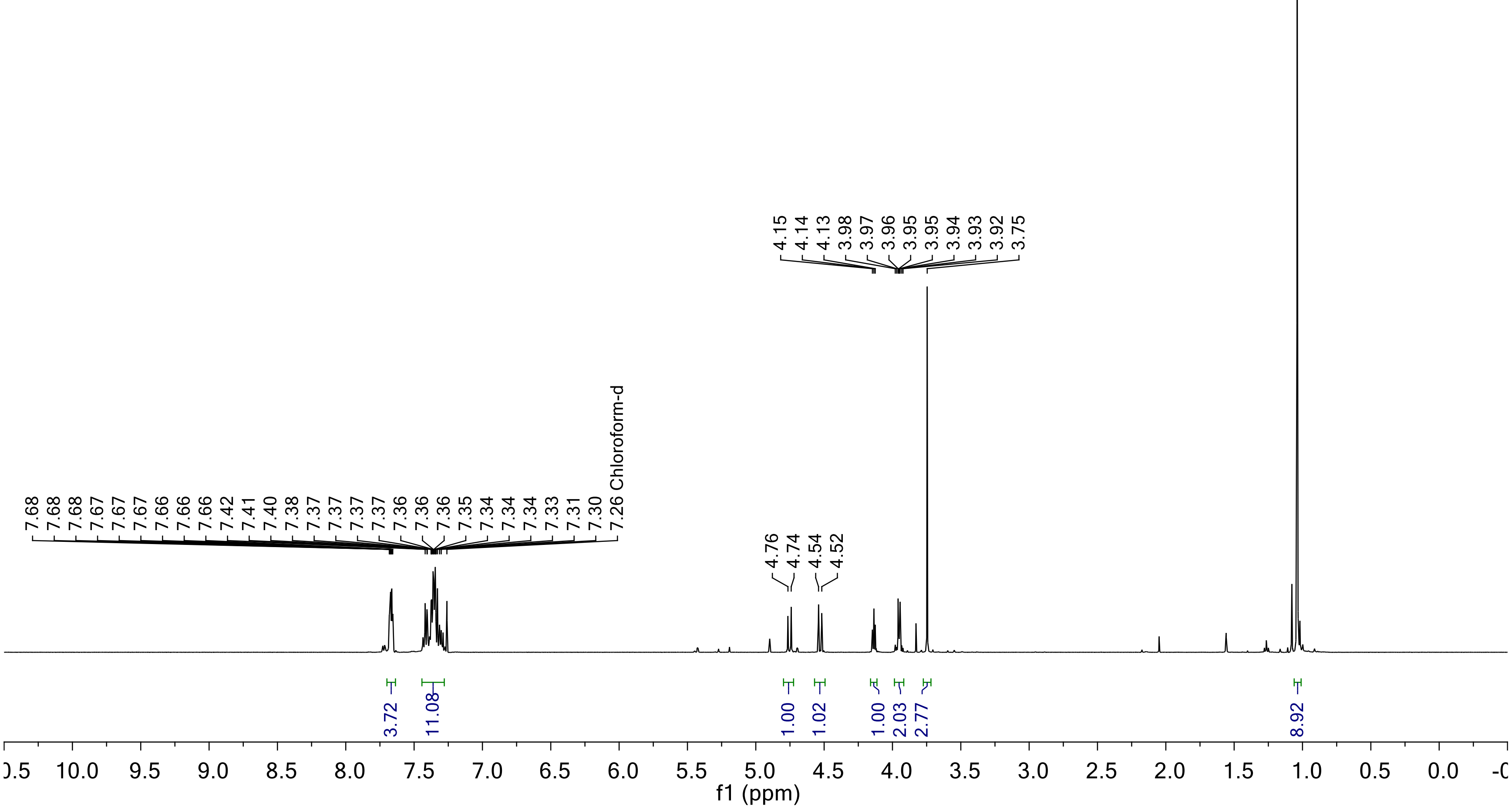

^{1}H-NMR (500 MHz, $CDCl_3$)

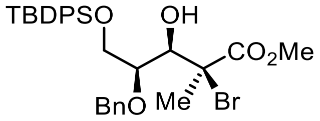

34a

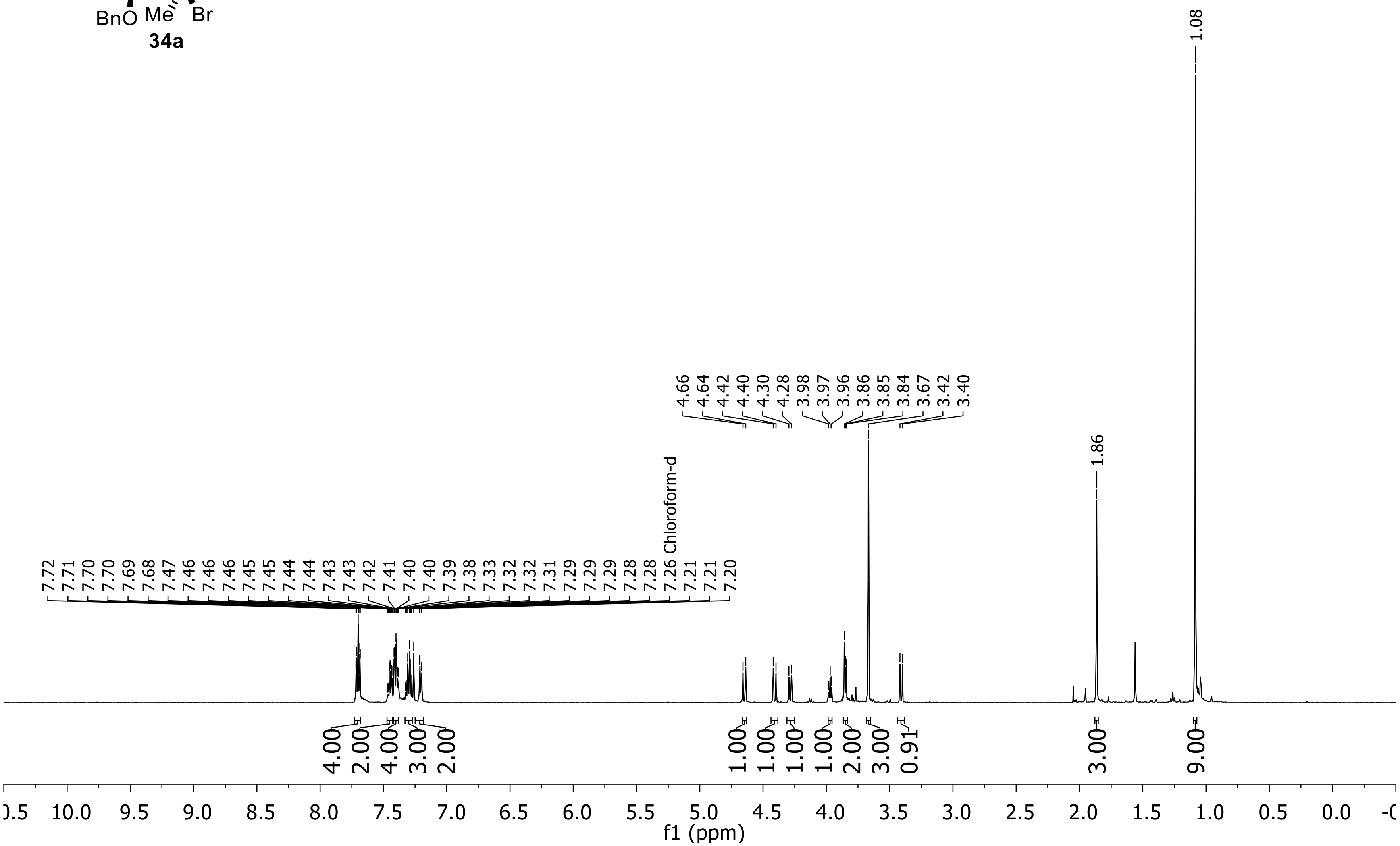

$^{13}C\{^1H\}$-NMR (126 MHz, $CDCl_3$)

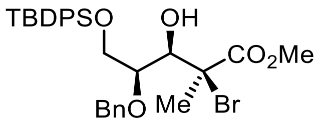

34a

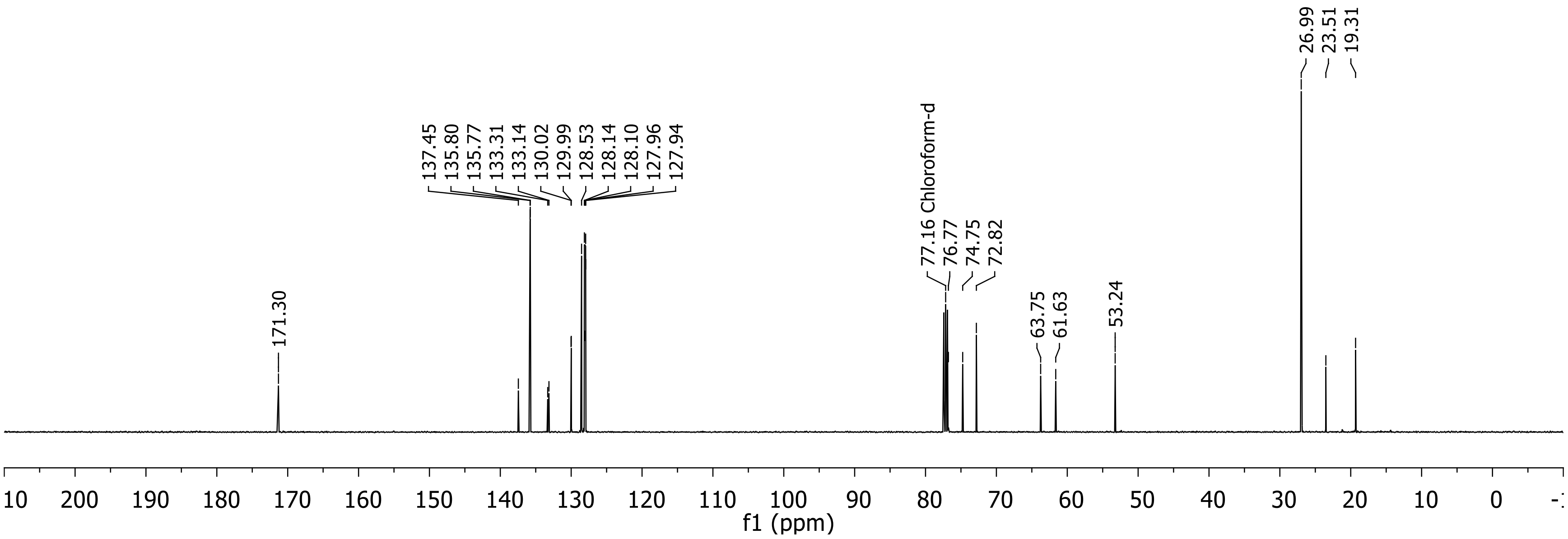

^{1}H-NMR (500 MHz, $CDCl_3$)

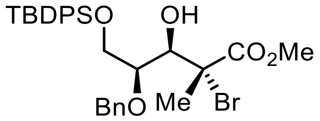

34b

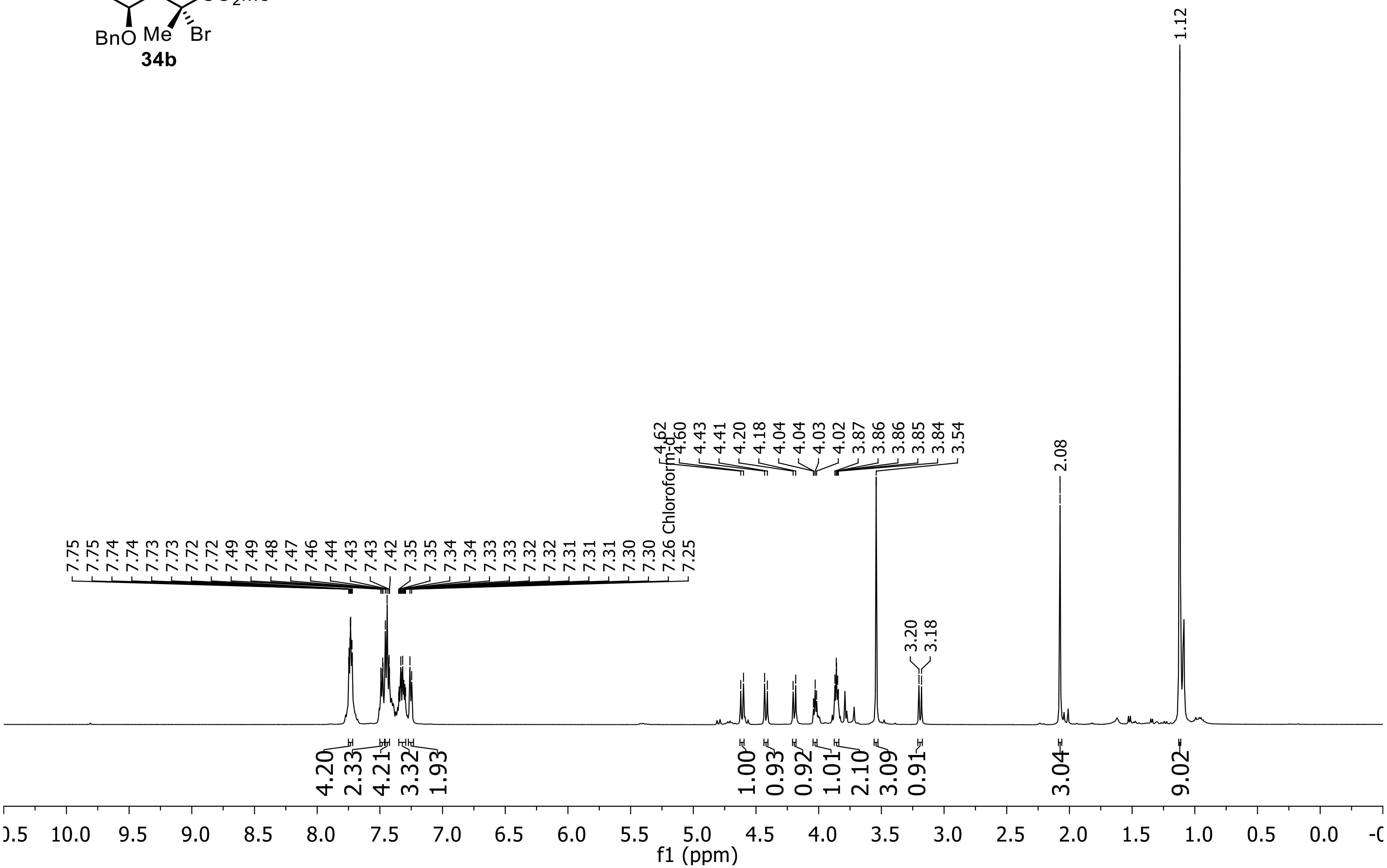

$^{13}C\{^{1}H\}$-NMR (126 MHz, $CDCl_3$)

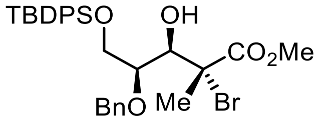

34b

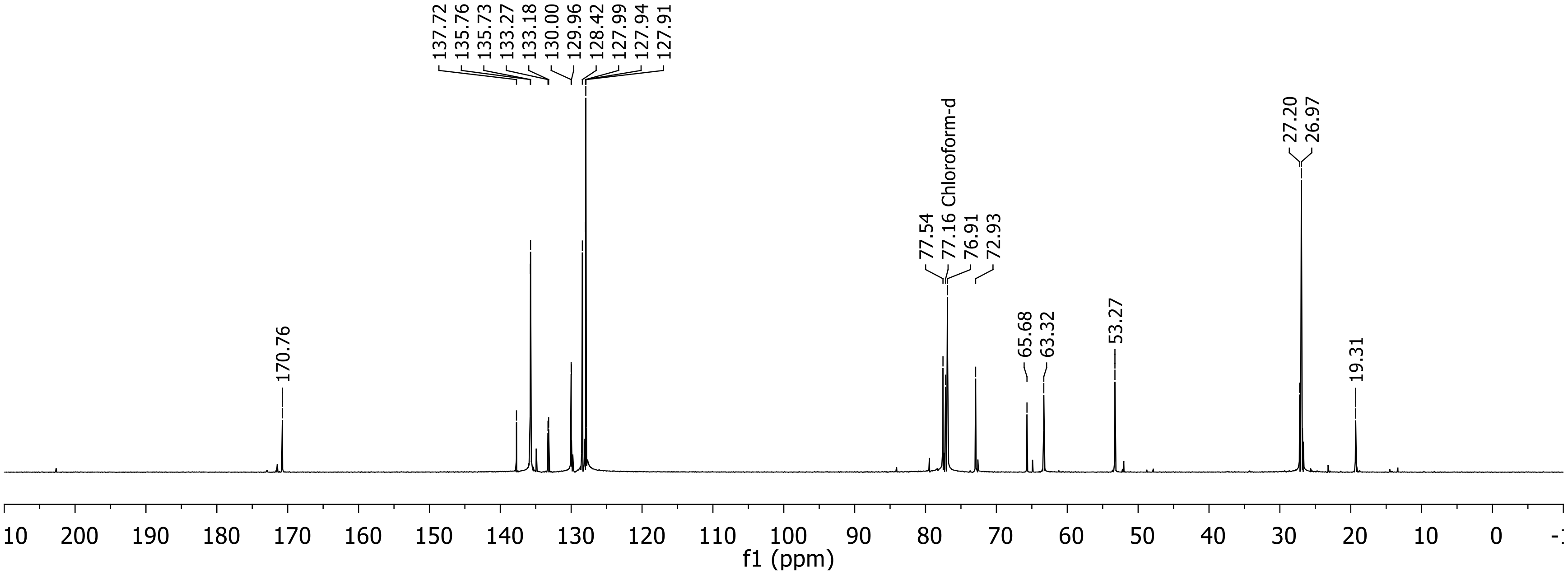

^{1}H-NMR (500 MHz, $CDCl_3$)

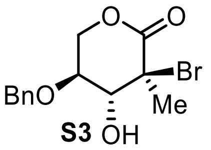

S3

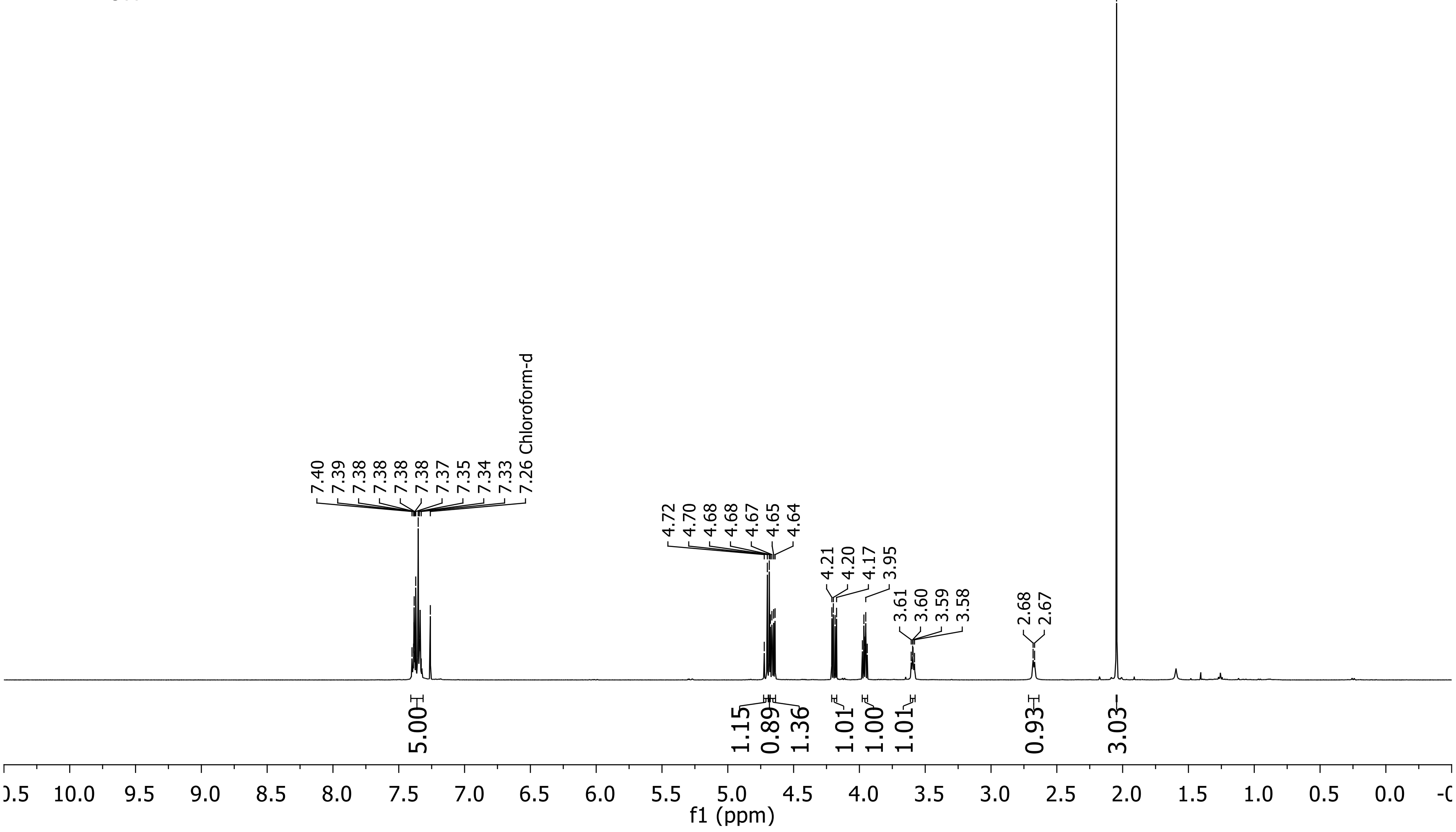

$^{13}C\{^1H\}$-NMR (126 MHz, $CDCl_3$)

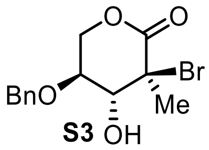

S3

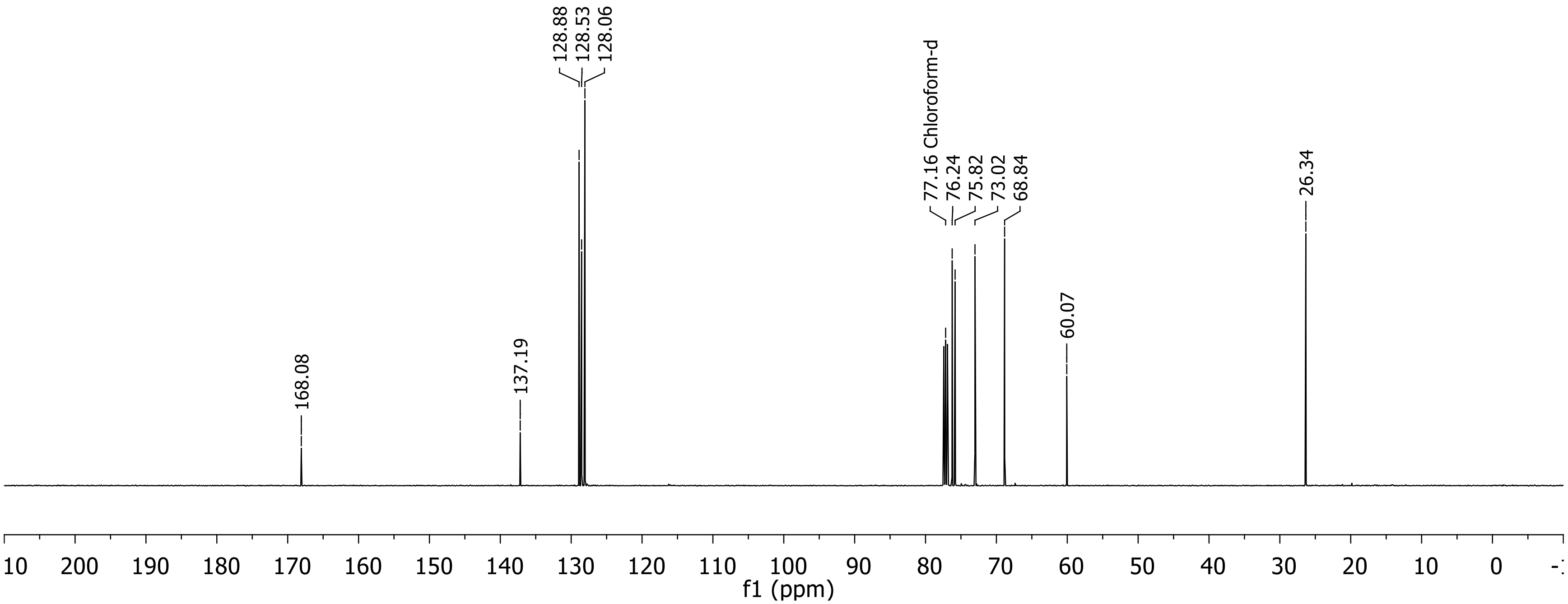

COSY (500 MHz, $CDCl_3$)

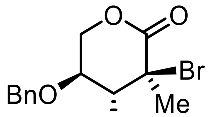

S3

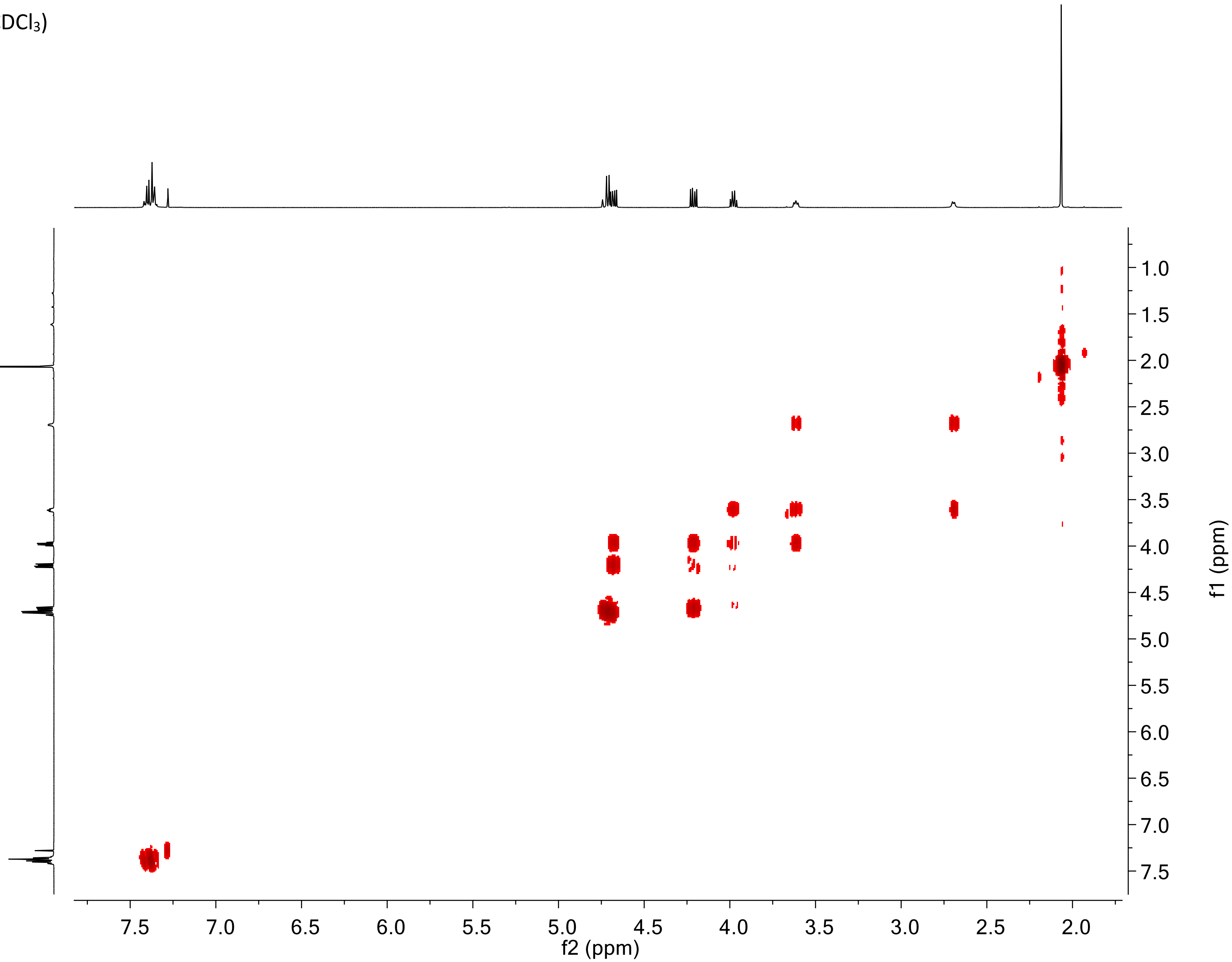

NOESY (500 MHz, $CDCl_3$)

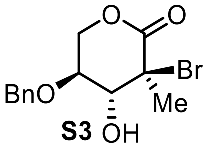

S3

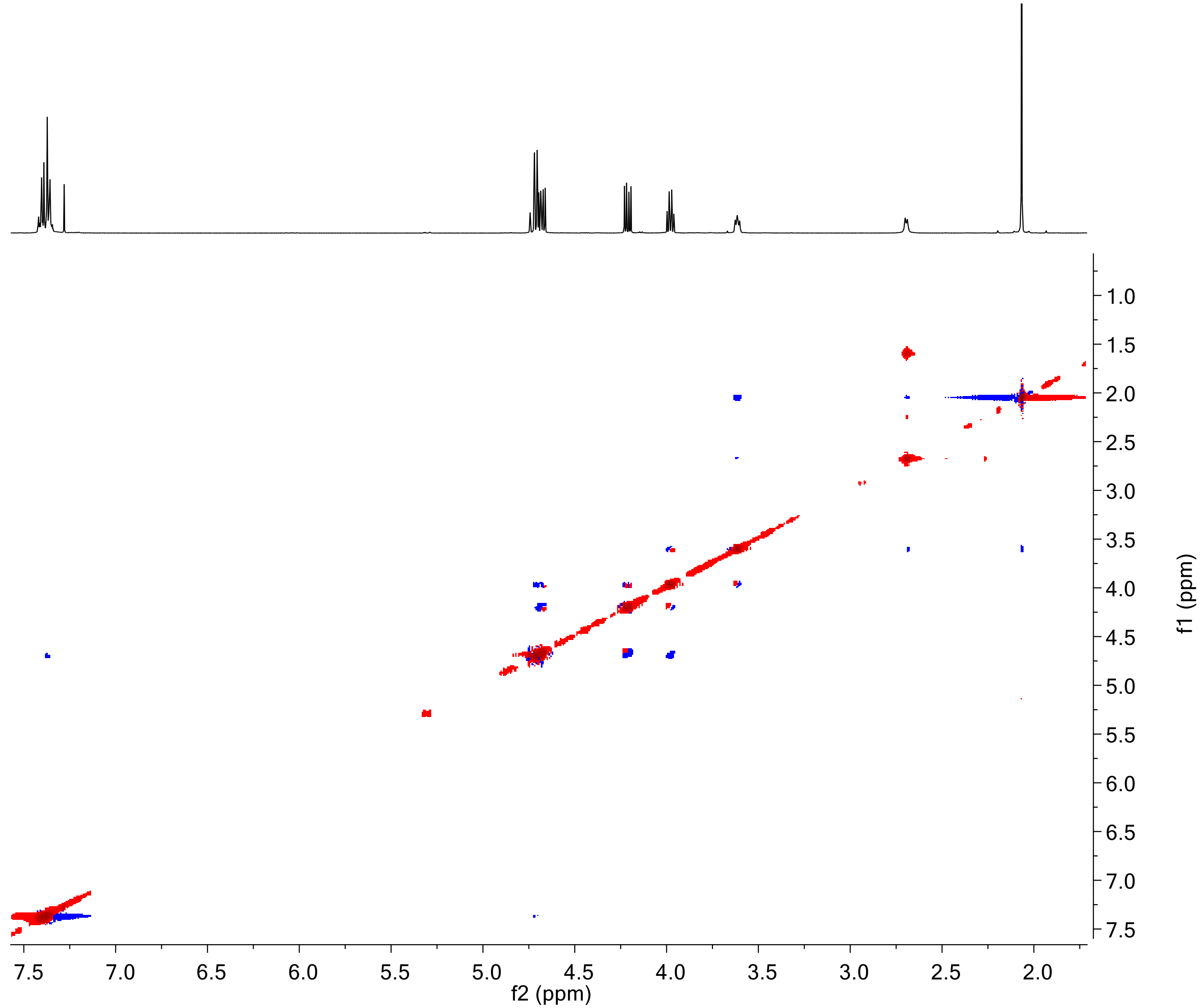

^{1}H-NMR (500 MHz, $CDCl_3$)

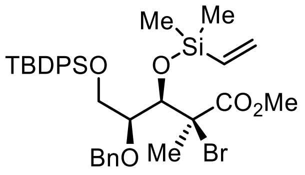

35a

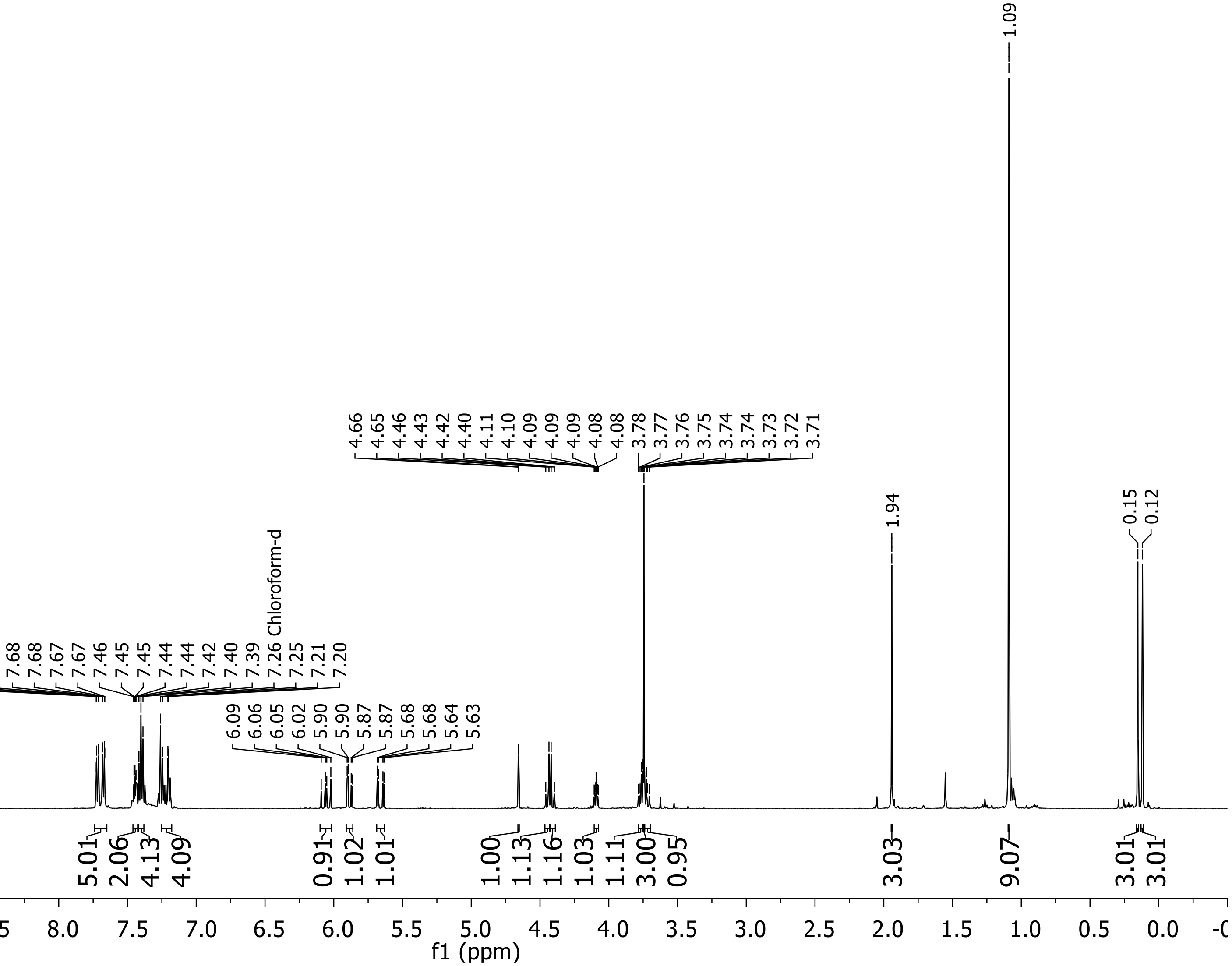

$^{13}C\{^{1}H\}$-NMR (126 MHz, $CDCl_3$)

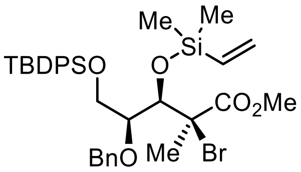

35a

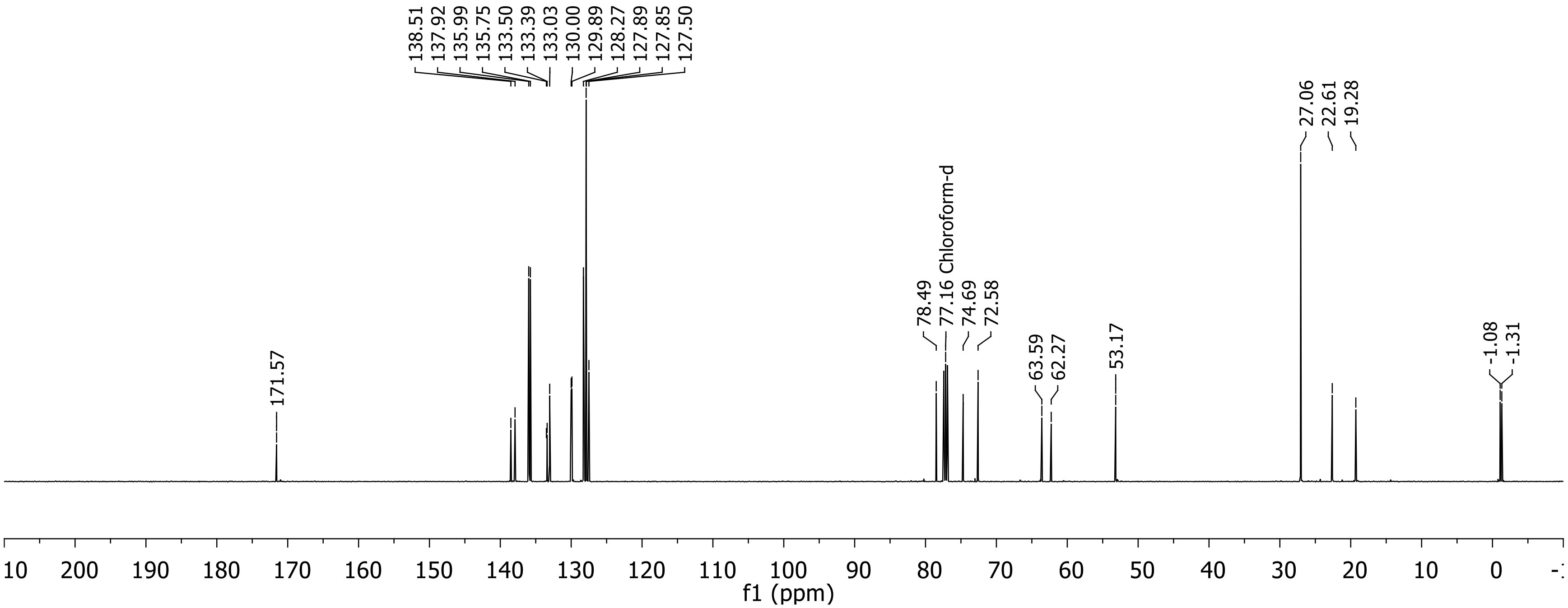

^{1}H-NMR (500 MHz, $CDCl_3$)

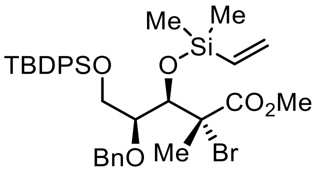

35b

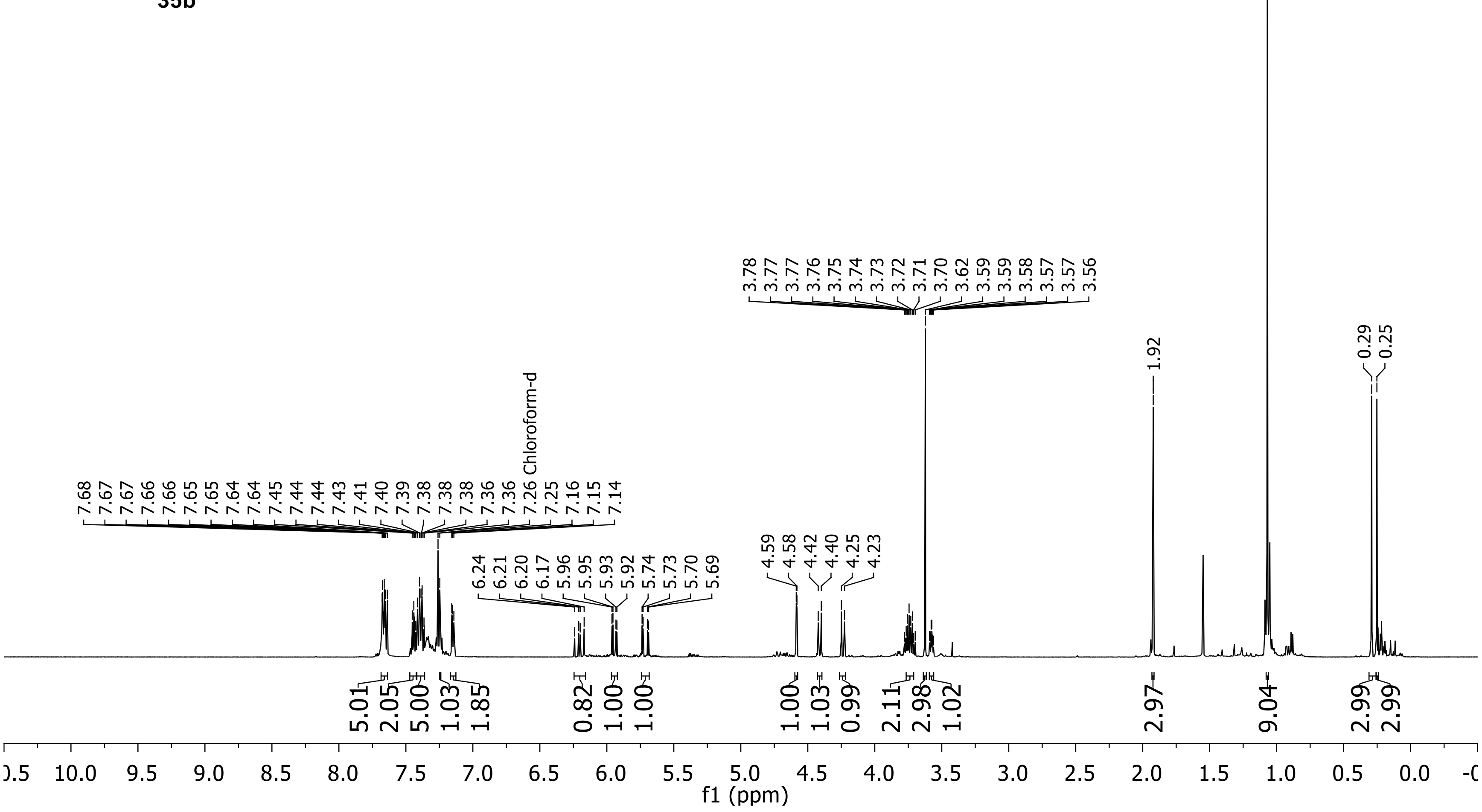

$^{13}C\{^1H\}$-NMR (126 MHz, $CDCl_3$)

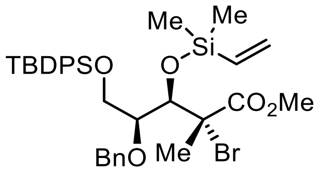

35b

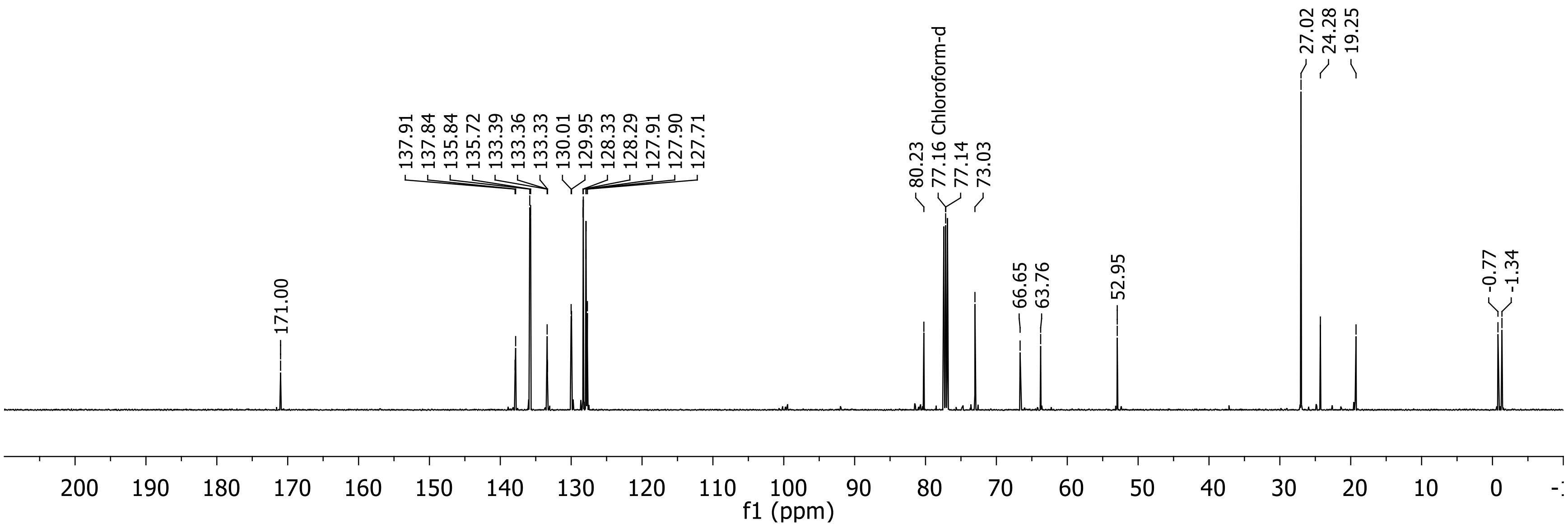

^{1}H-NMR (500 MHz, $CDCl_3$)

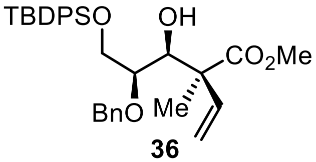

36

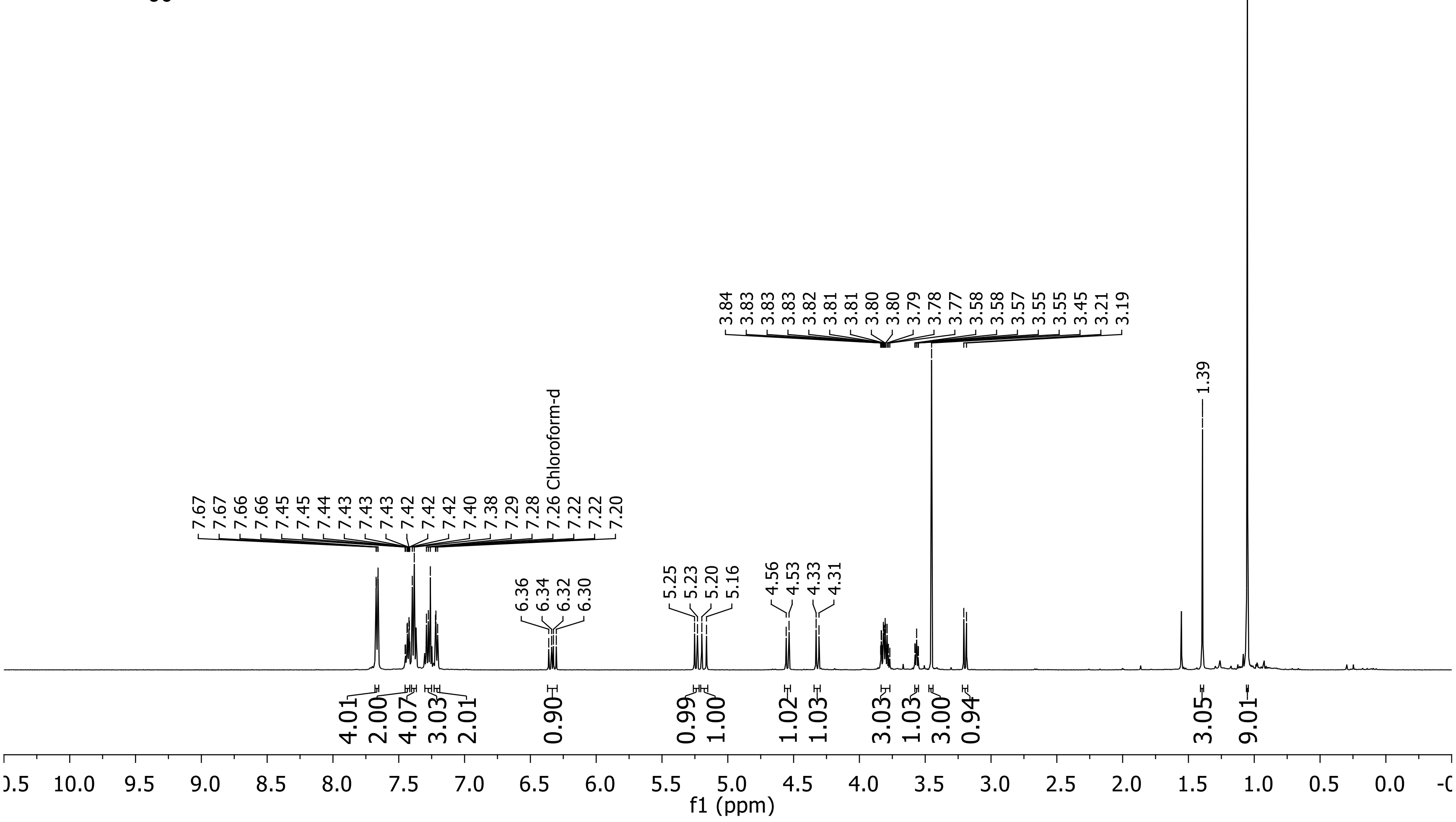

$^{13}C\{^{1}H\}$-NMR (126 MHz, $CDCl_3$)

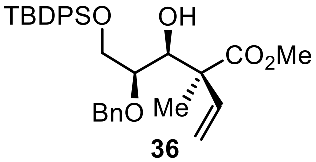

36

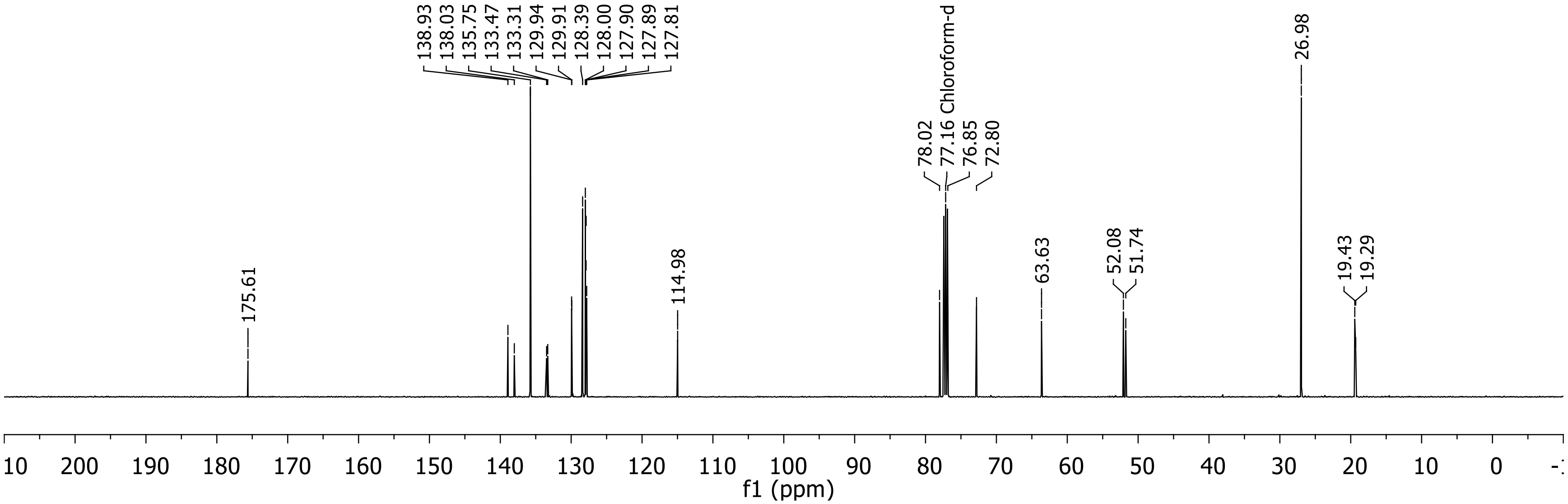

^{1}H-NMR (500 MHz, $CDCl_3$)

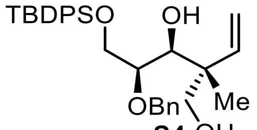

S4

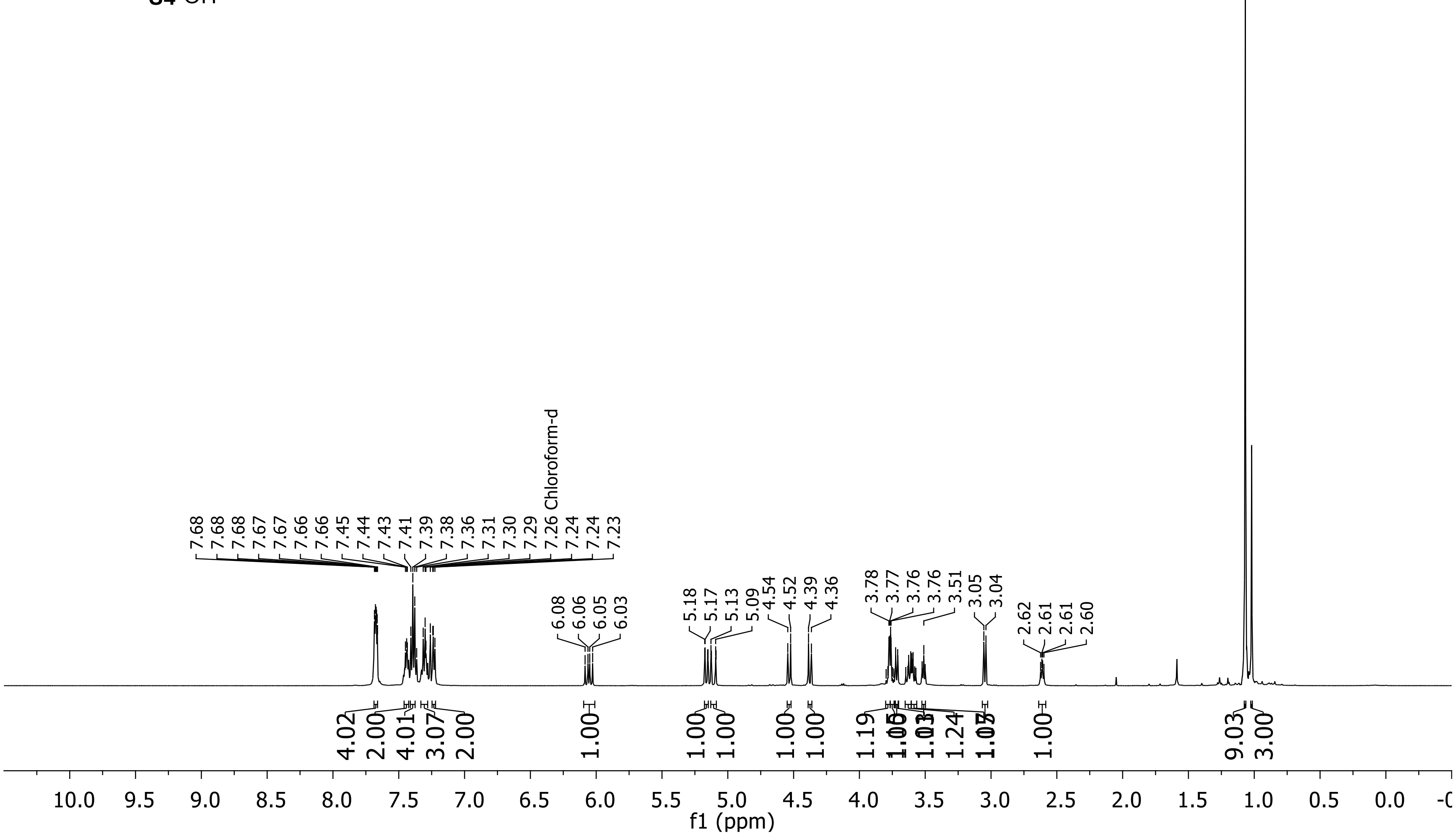

$^{13}C\{^1H\}$-NMR (126 MHz, $CDCl_3$)

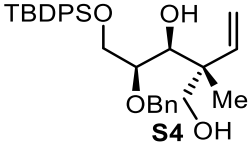

S4

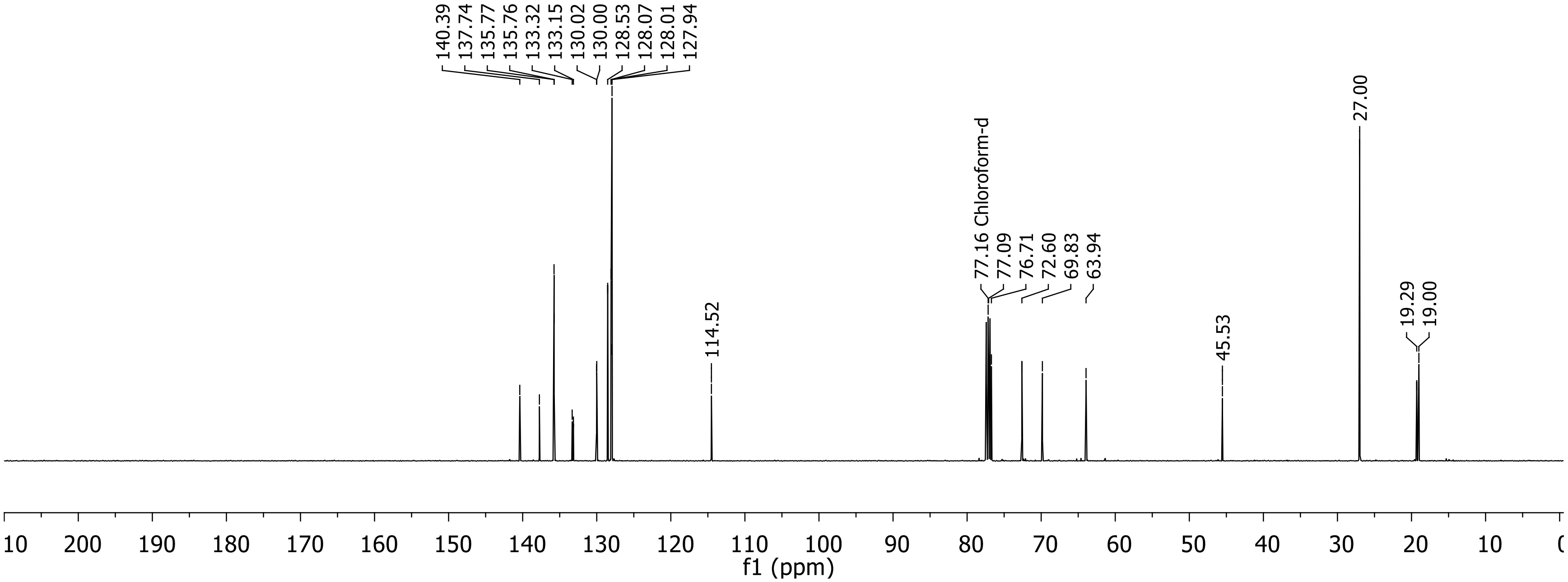

^{1}H-NMR (500 MHz, $CDCl_3$)

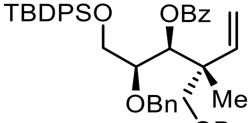

37

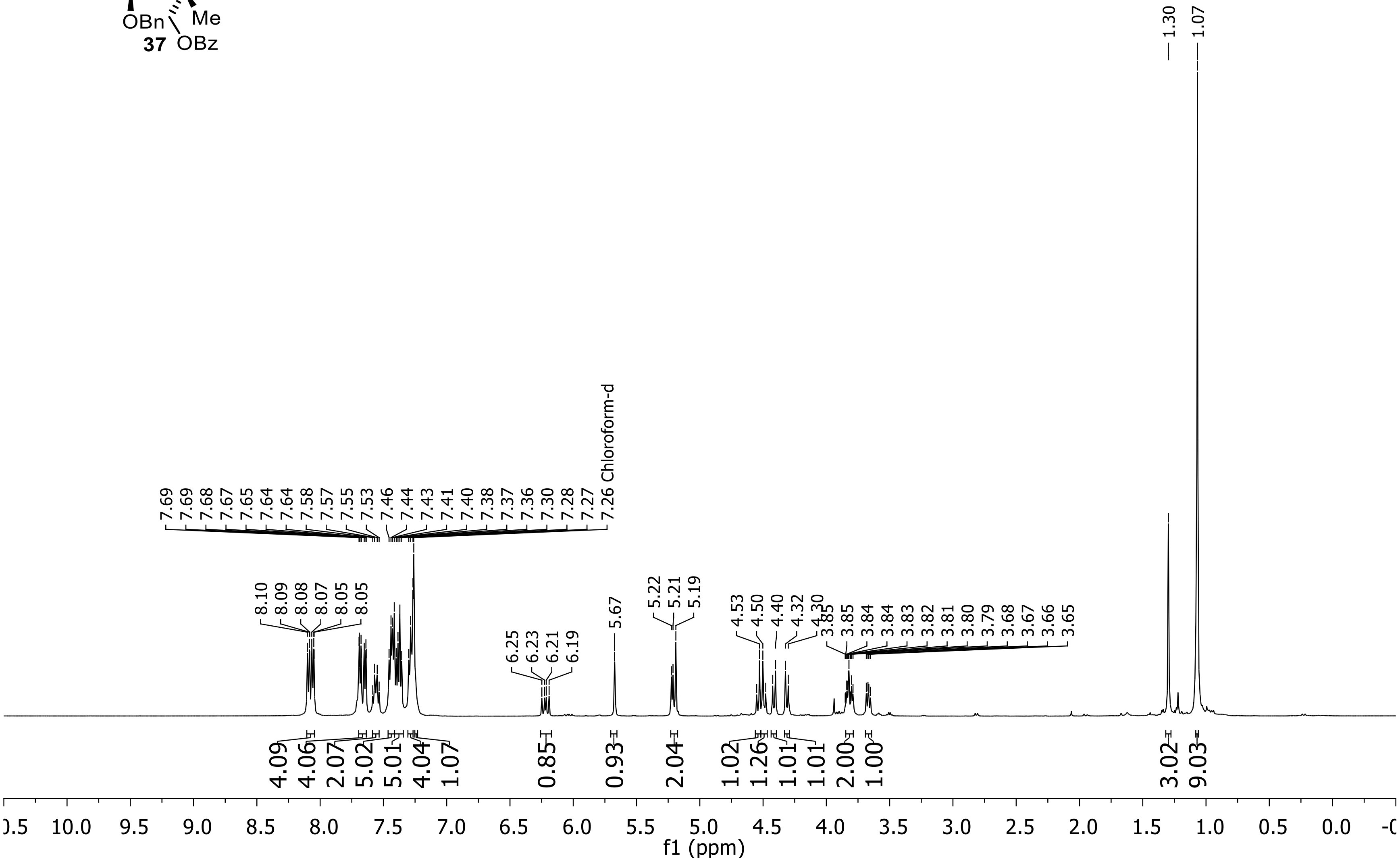

$^{13}C\{^{1}H\}$-NMR (126 MHz, $CDCl_3$)

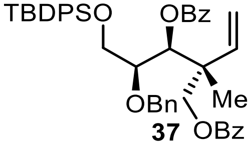

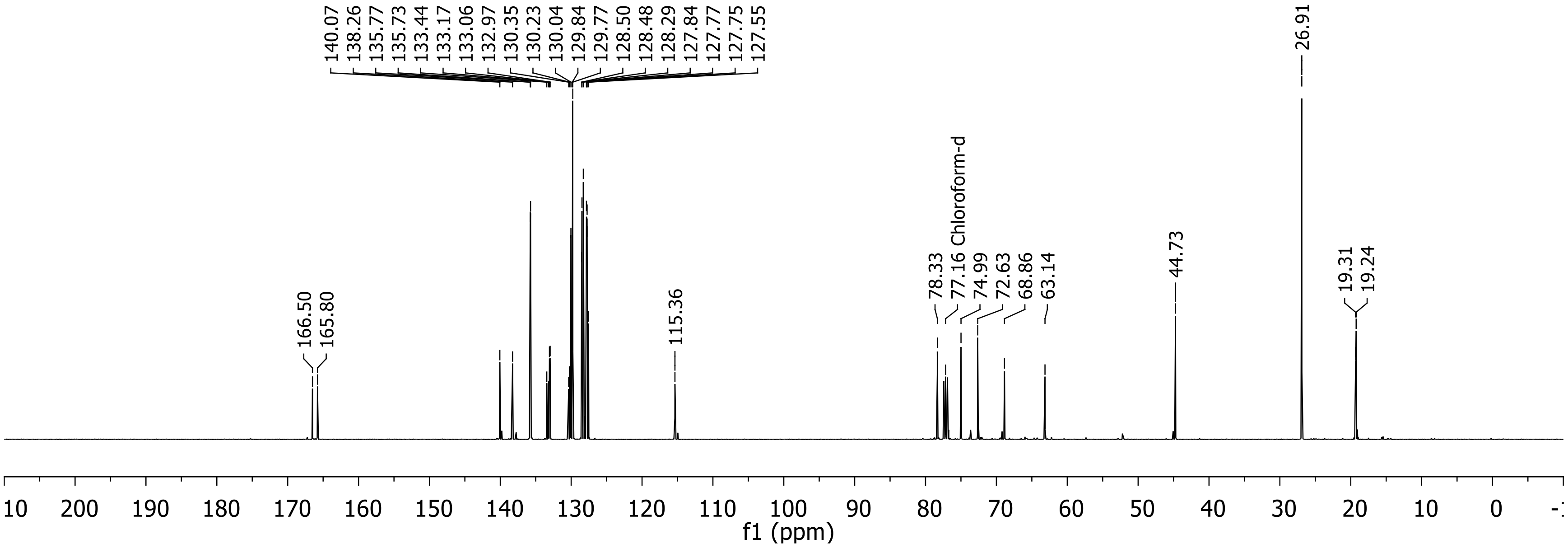

^{1}H-NMR (500 MHz, $CDCl_3$)

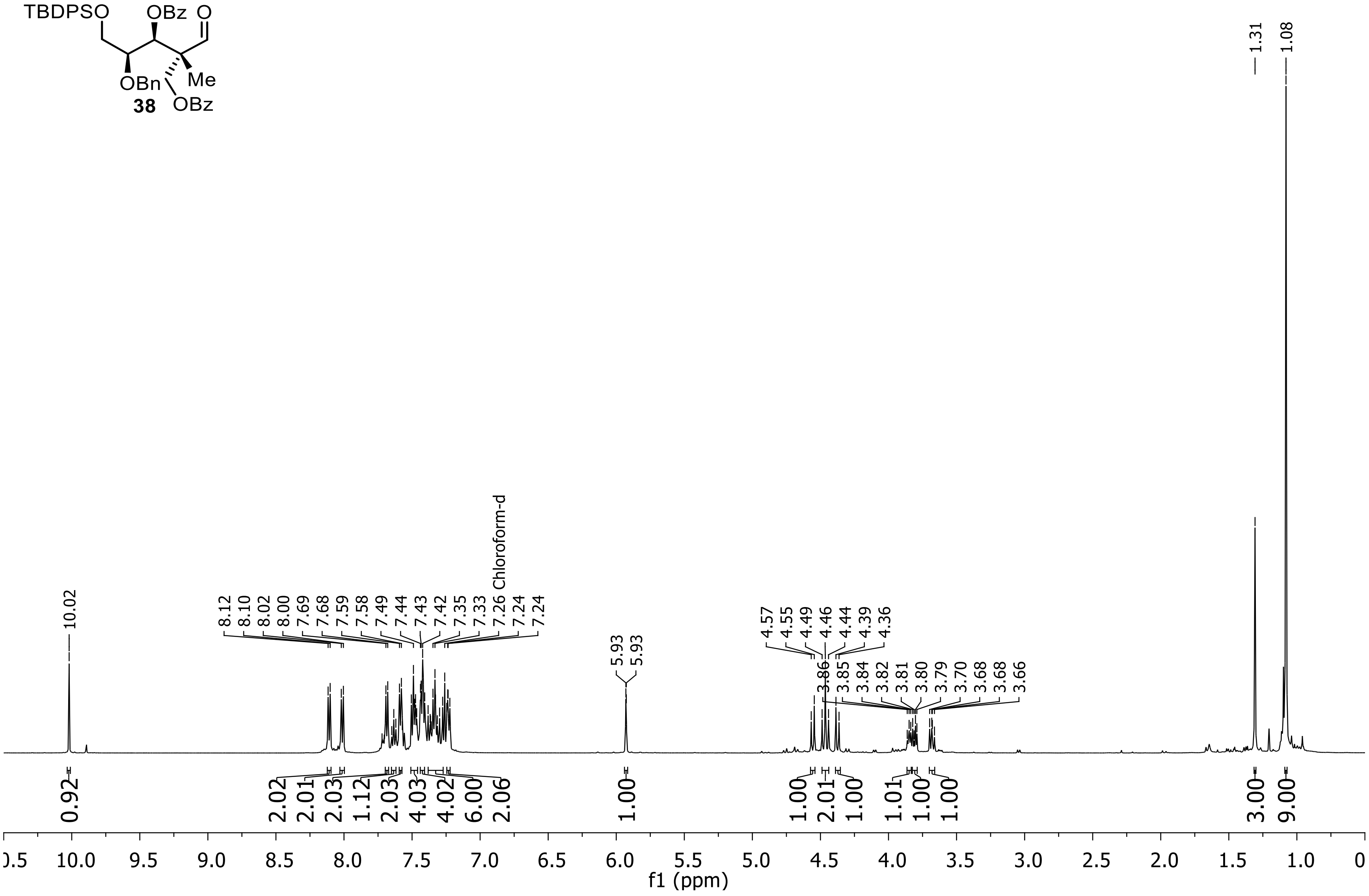

$^{13}C\{^{1}H\}$-NMR (126 MHz, $CDCl_3$)

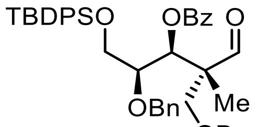

38

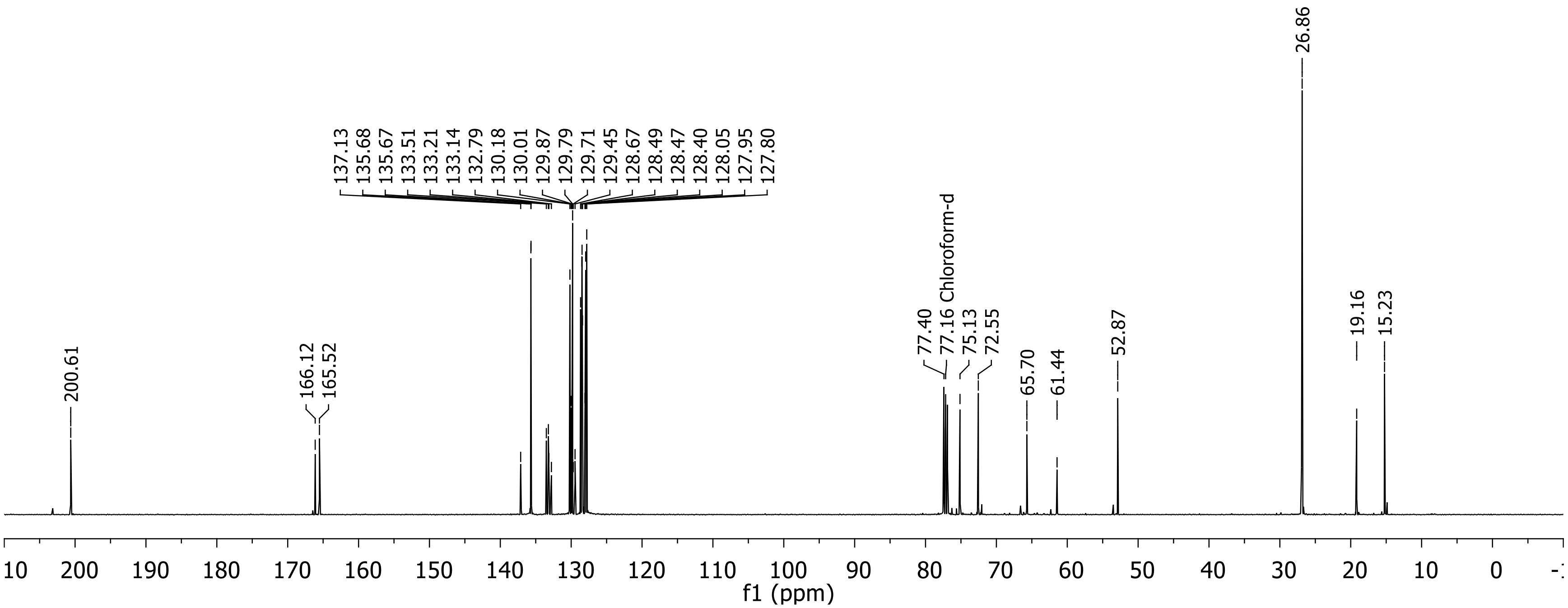

^{1}H-NMR (500 MHz, $CDCl_3$)

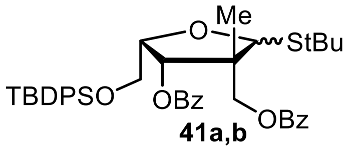

41a,b

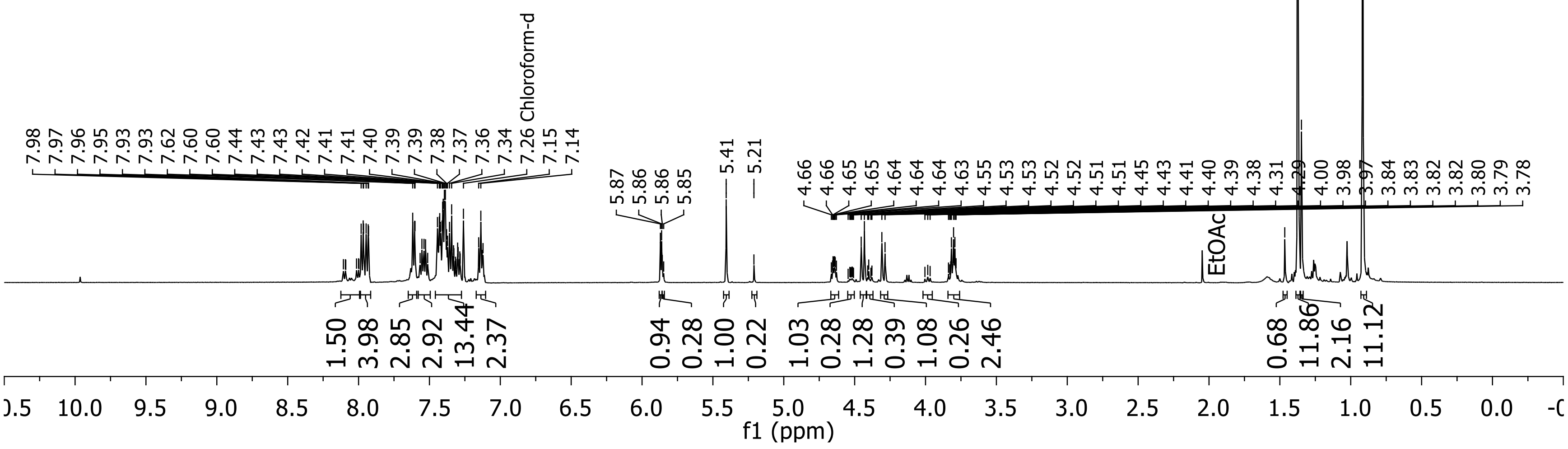

$^{13}C\{^{1}H\}$-NMR (126 MHz, $CDCl_3$)

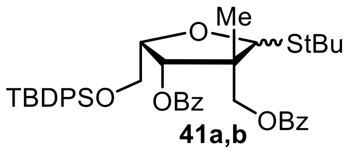

41a,b

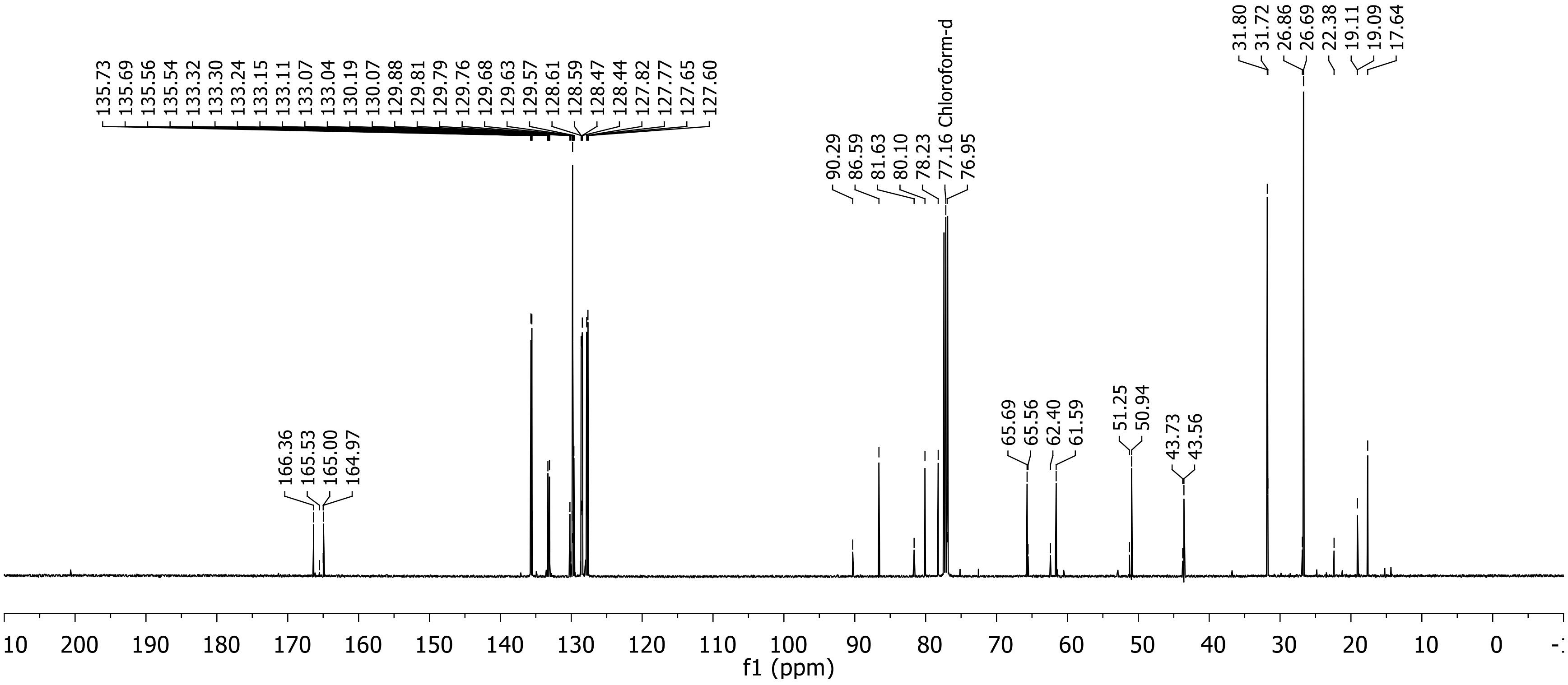

COSY (500 MHz, $CDCl_3$)

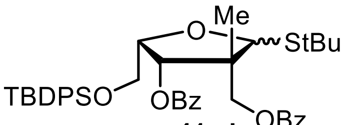

41a,b

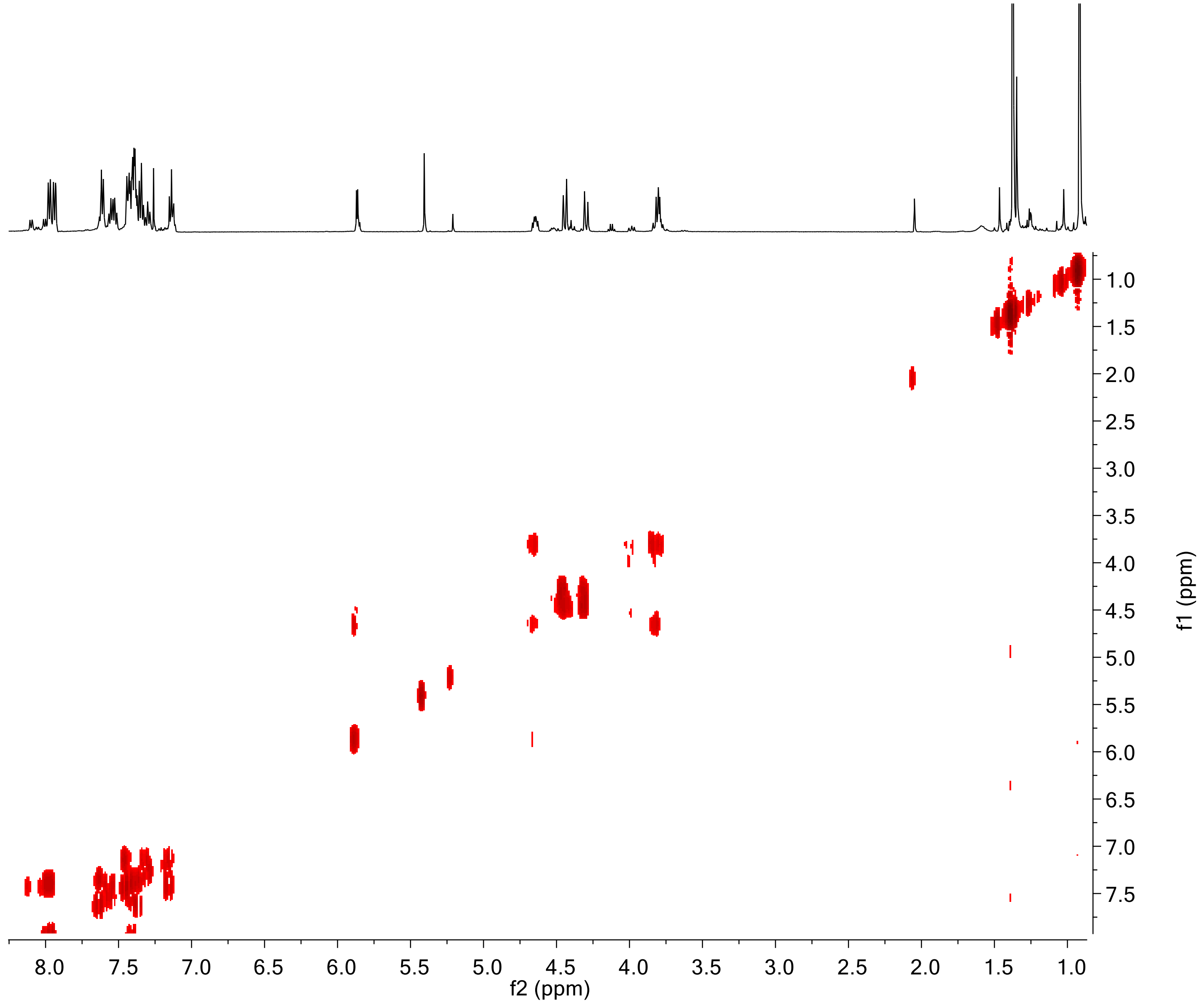

NOESY (500 MHz, $CDCl_3$)

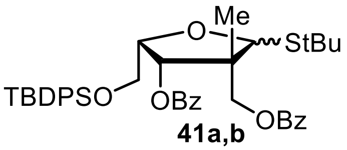

41a,b

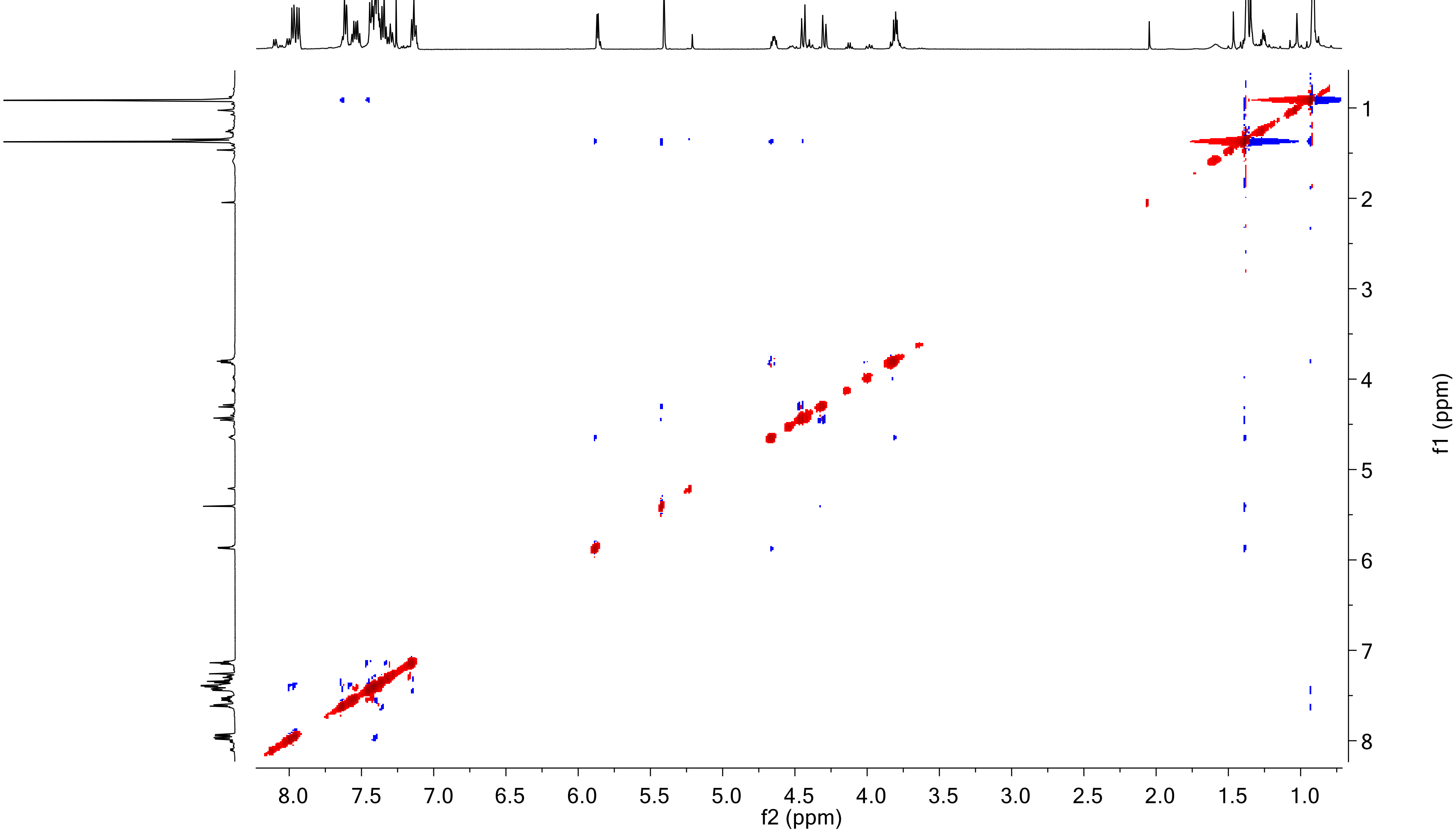

^{1}H-NMR (500 MHz, $CDCl_3$)

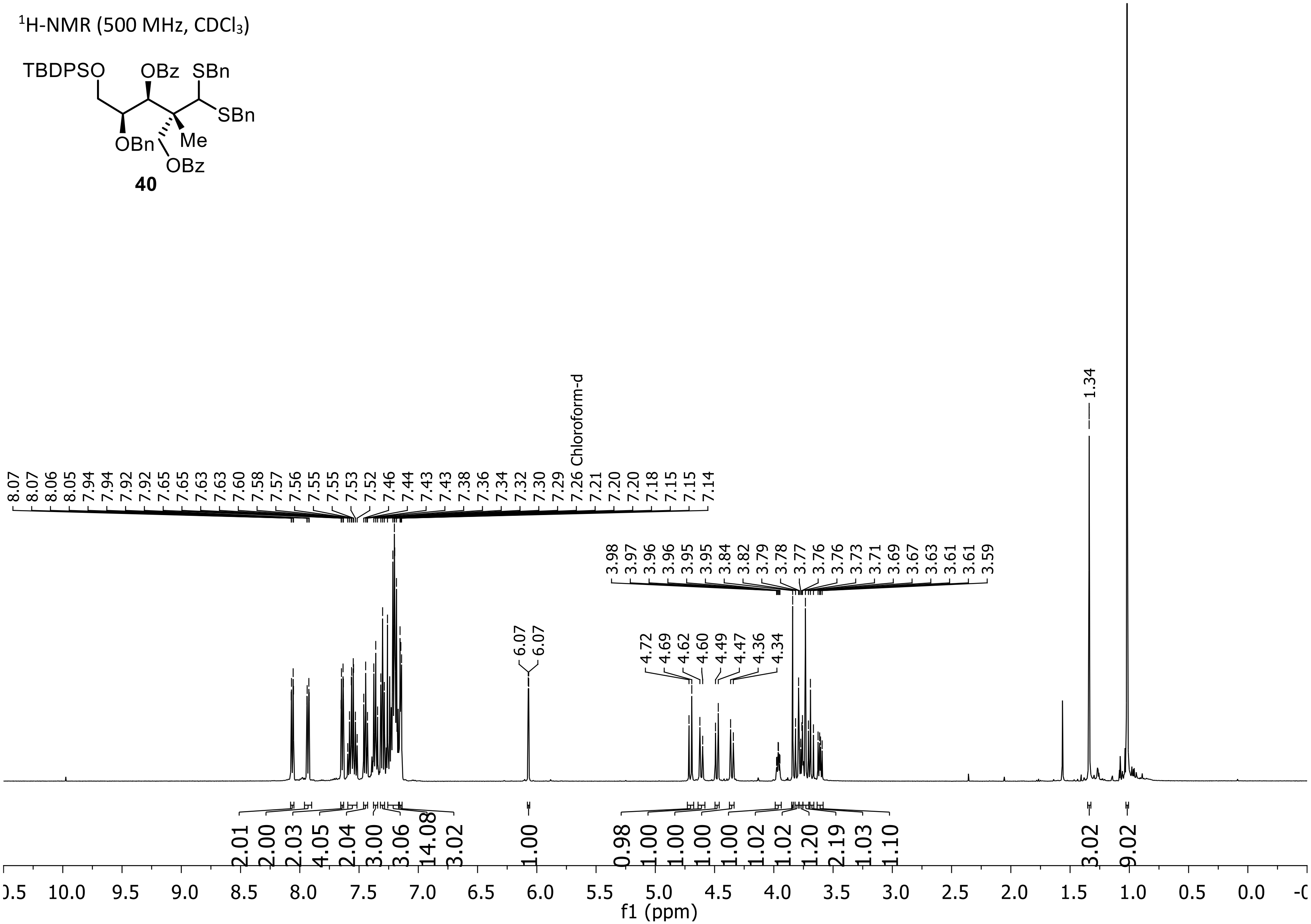

40

$^{13}C\{^{1}H\}$-NMR (126 MHz, $CDCl_3$)

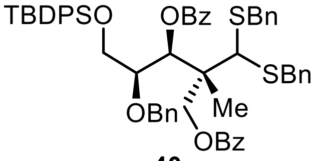

40

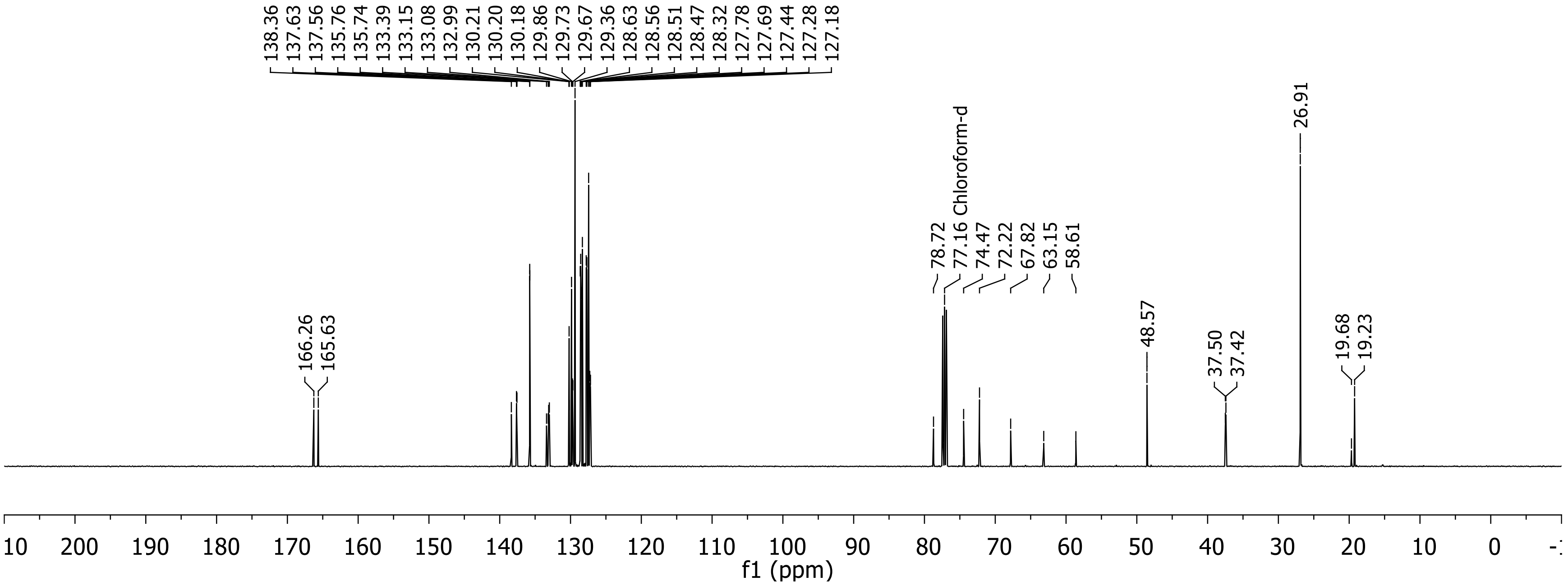

^{1}H-NMR (500 MHz, $CDCl_3$)

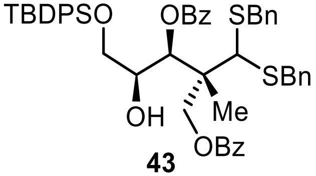

43

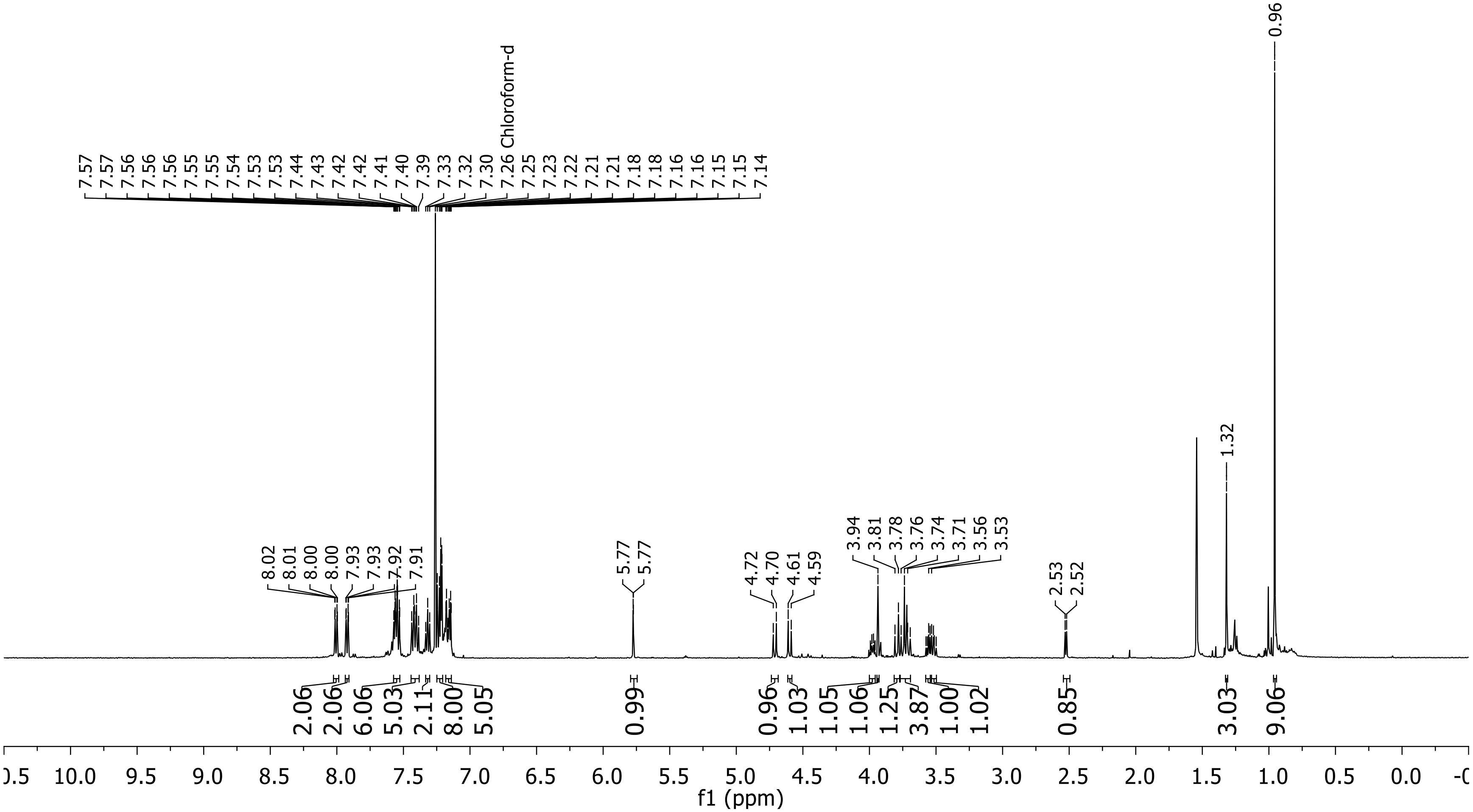

$^{13}C\{^1H\}$-NMR (126 MHz, $CDCl_3$)

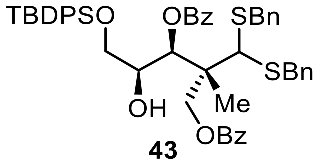

43

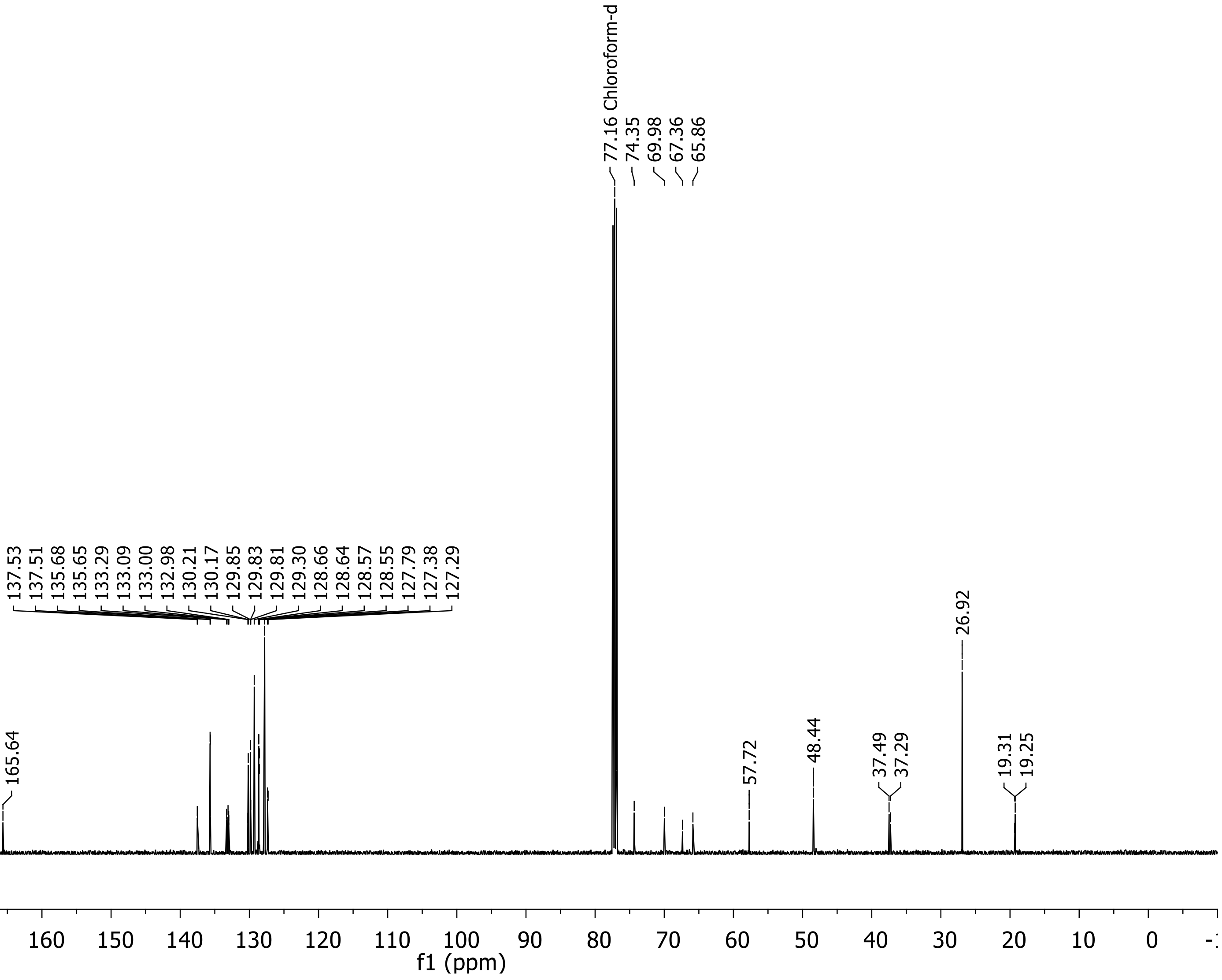

^{1}H-NMR (500 MHz, $CDCl_3$)

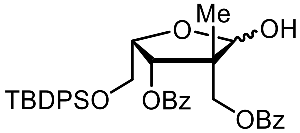

42a,b

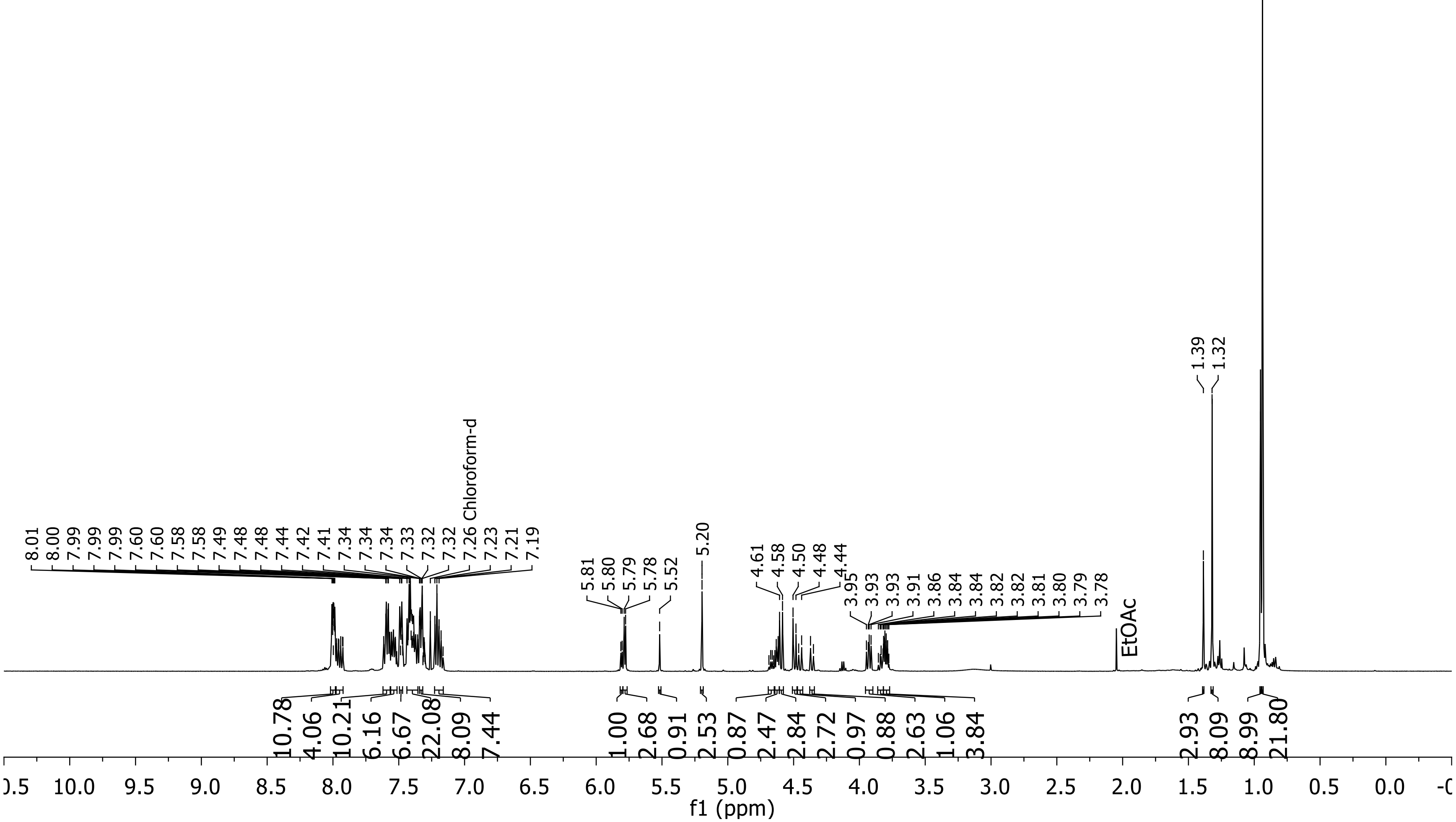

$^{13}C\{^{1}H\}$-NMR (126 MHz, $CDCl_3$)

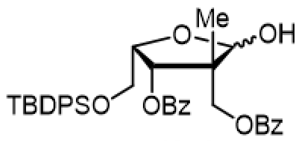

42a,b

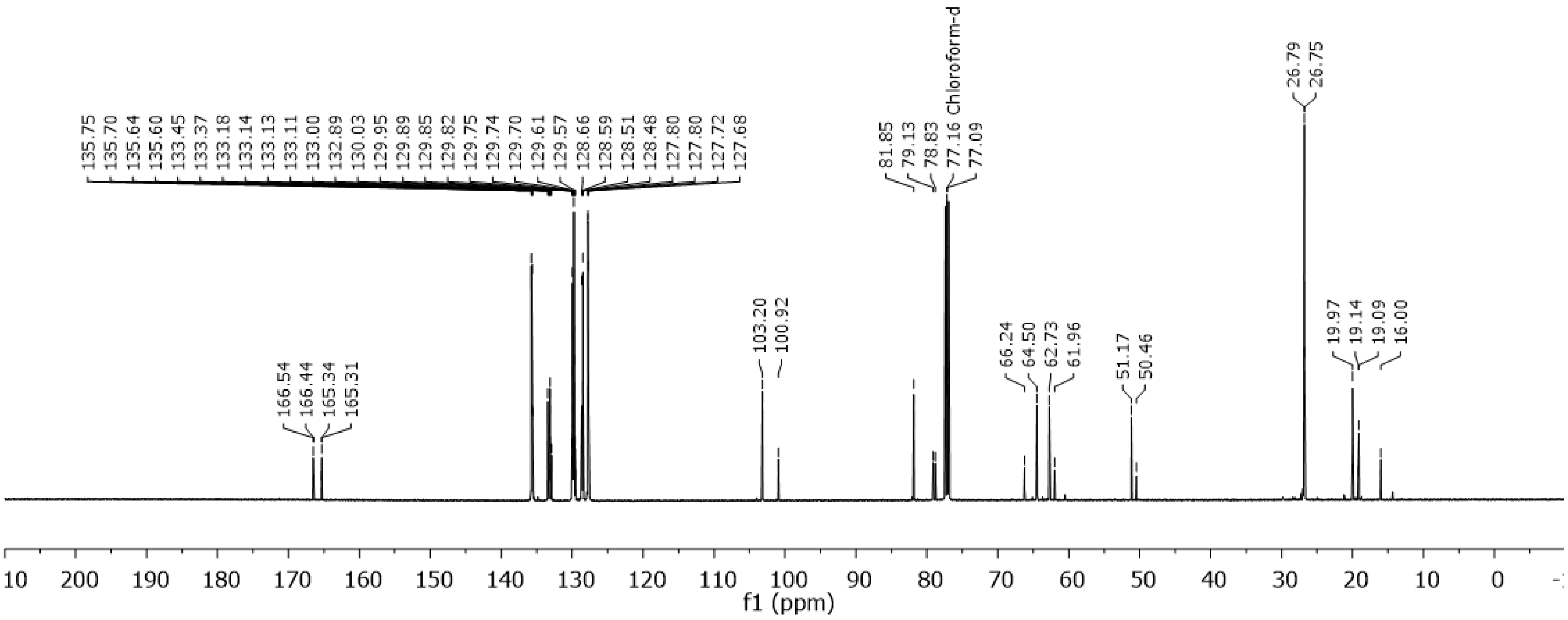

HSQC (500 MHz, $CDCl_3$)

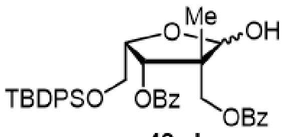

42a,b

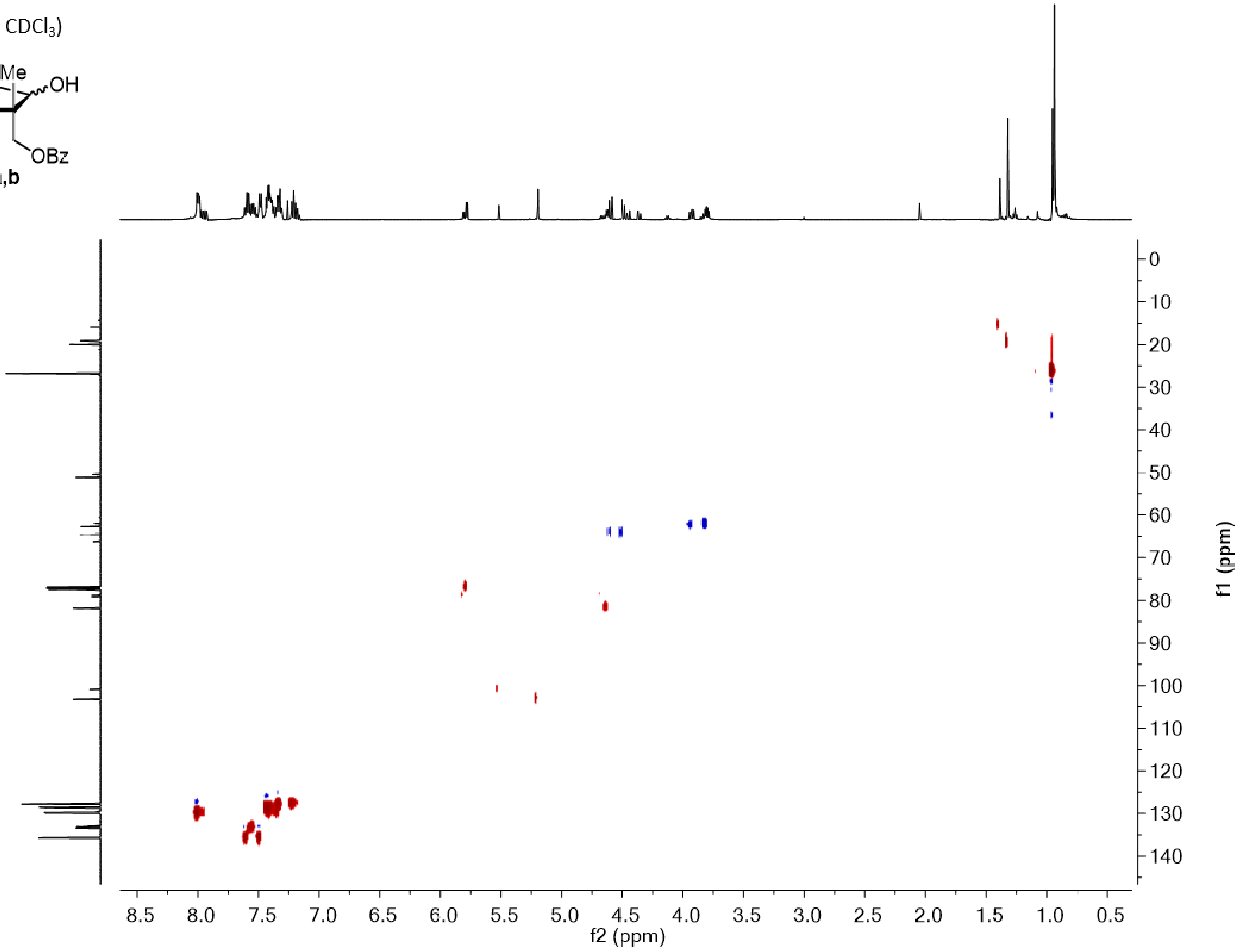

NOESY (500 MHz, $CDCl_3$)

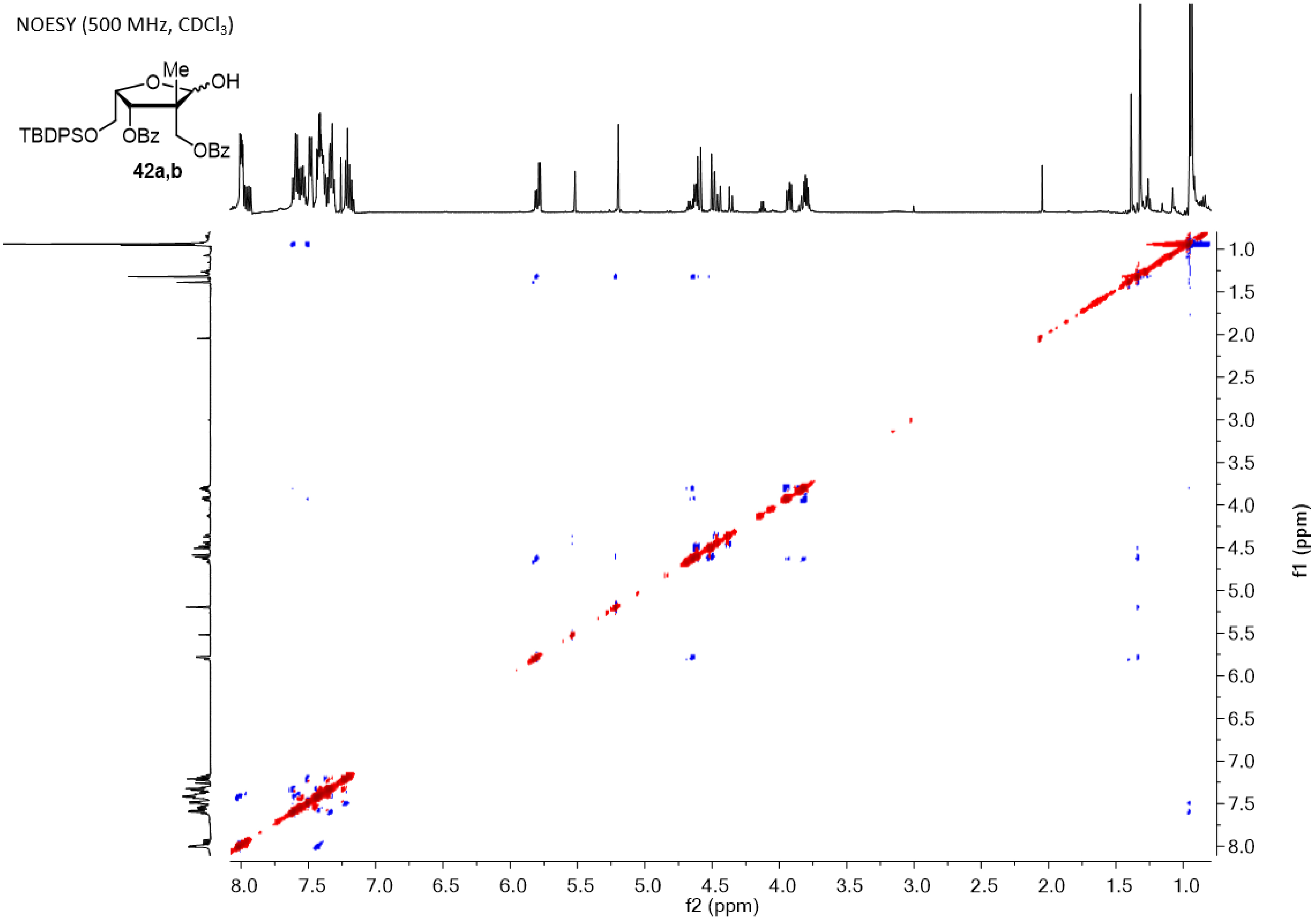

^{1}H-NMR (500 MHz, $CDCl_3$)

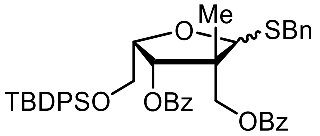

44a,b

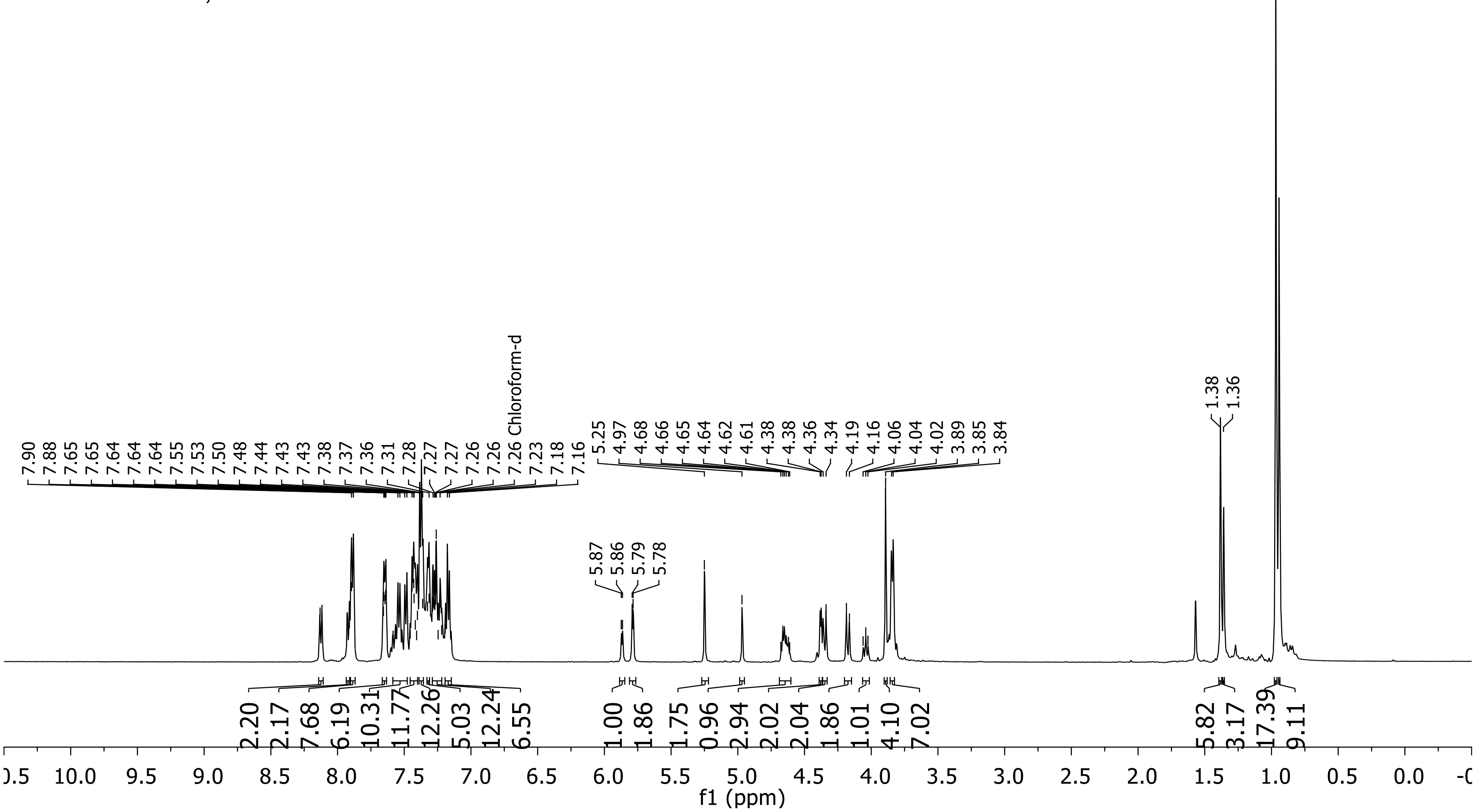

$^{13}C\{^{1}H\}$-NMR (126 MHz, $CDCl_3$)

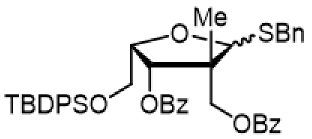

44a,b

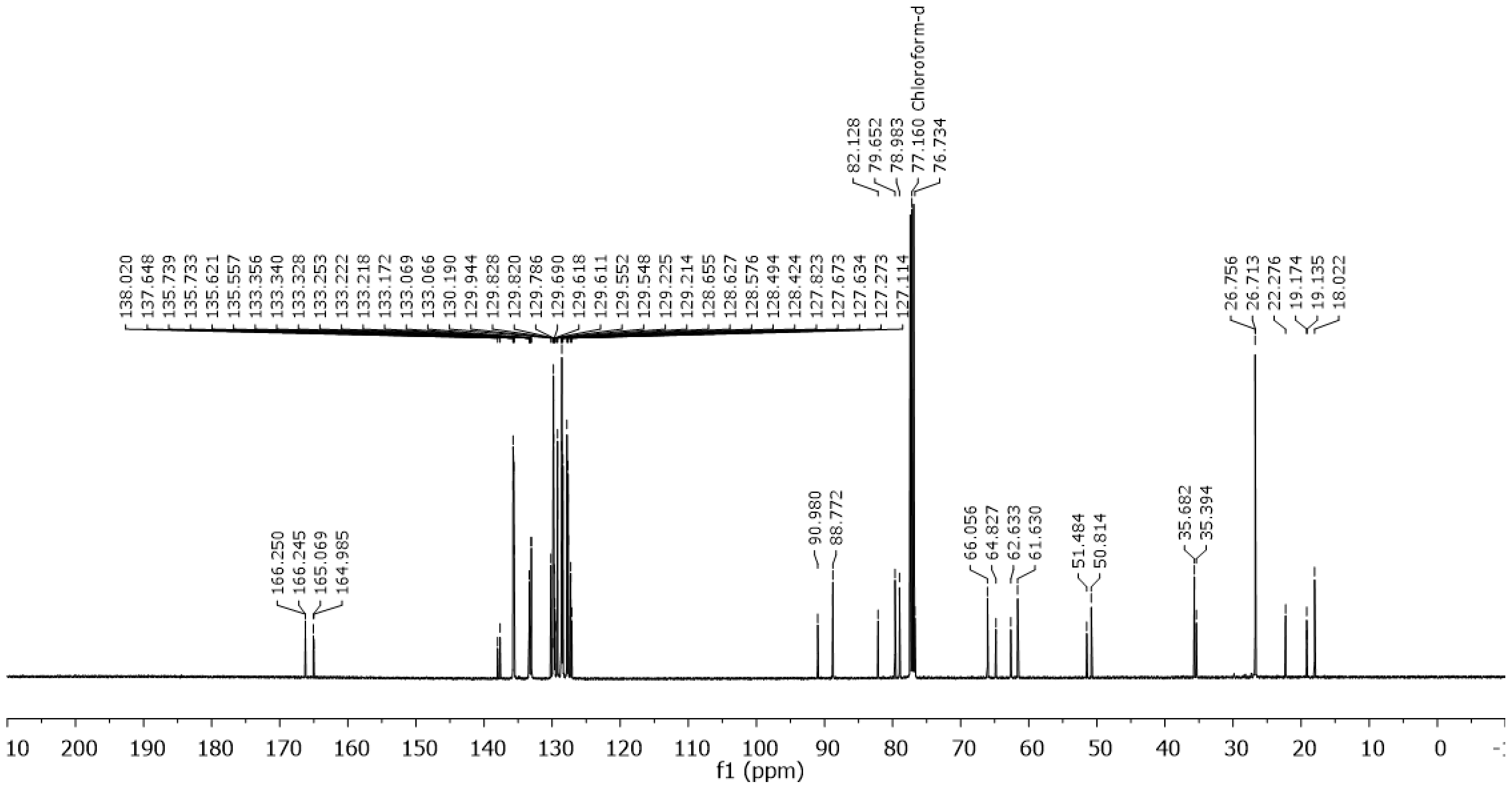

HSQC (500 MHz, $CDCl_3$)

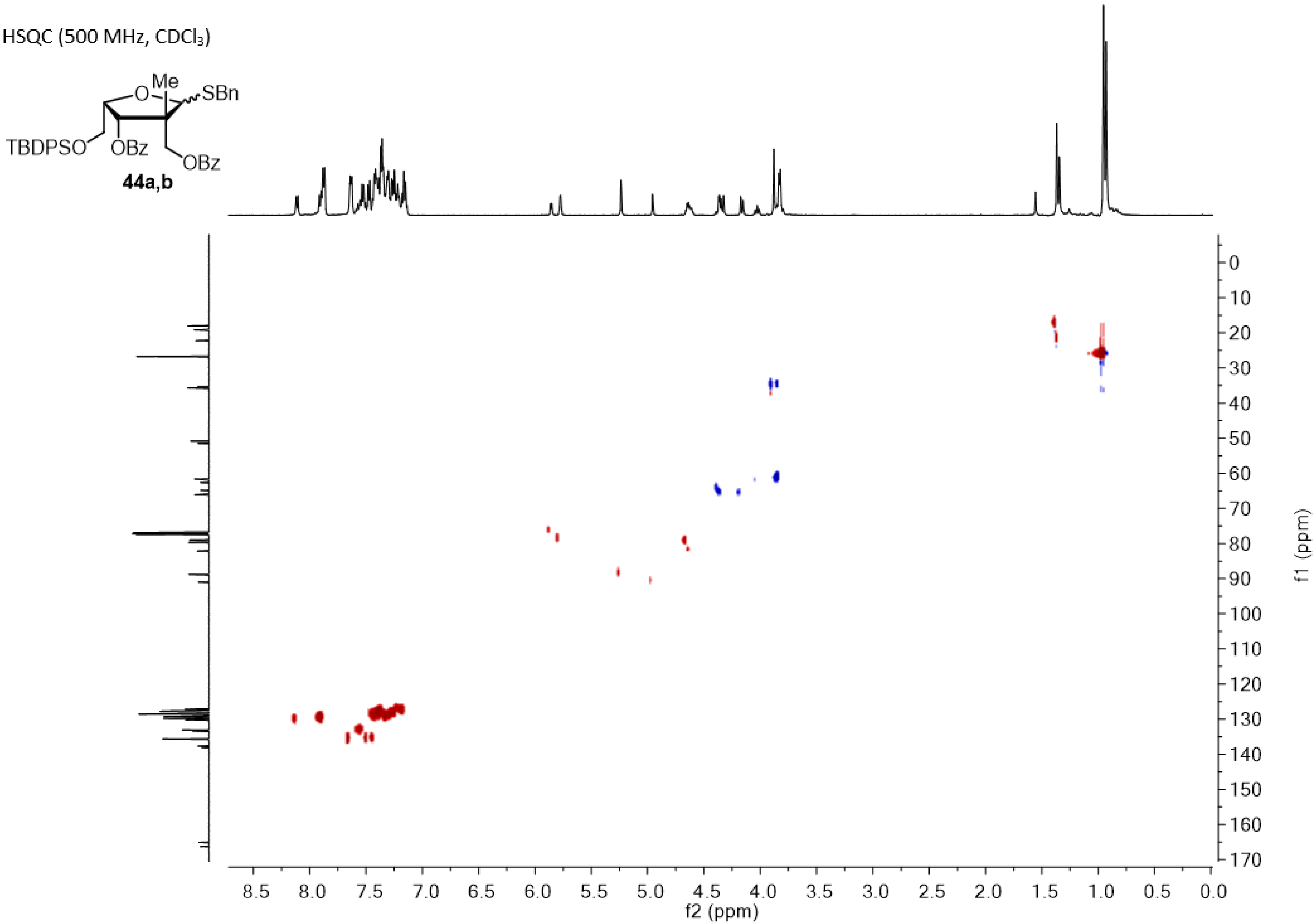

44a,b

NOESY (500 MHz, $CDCl_3$)

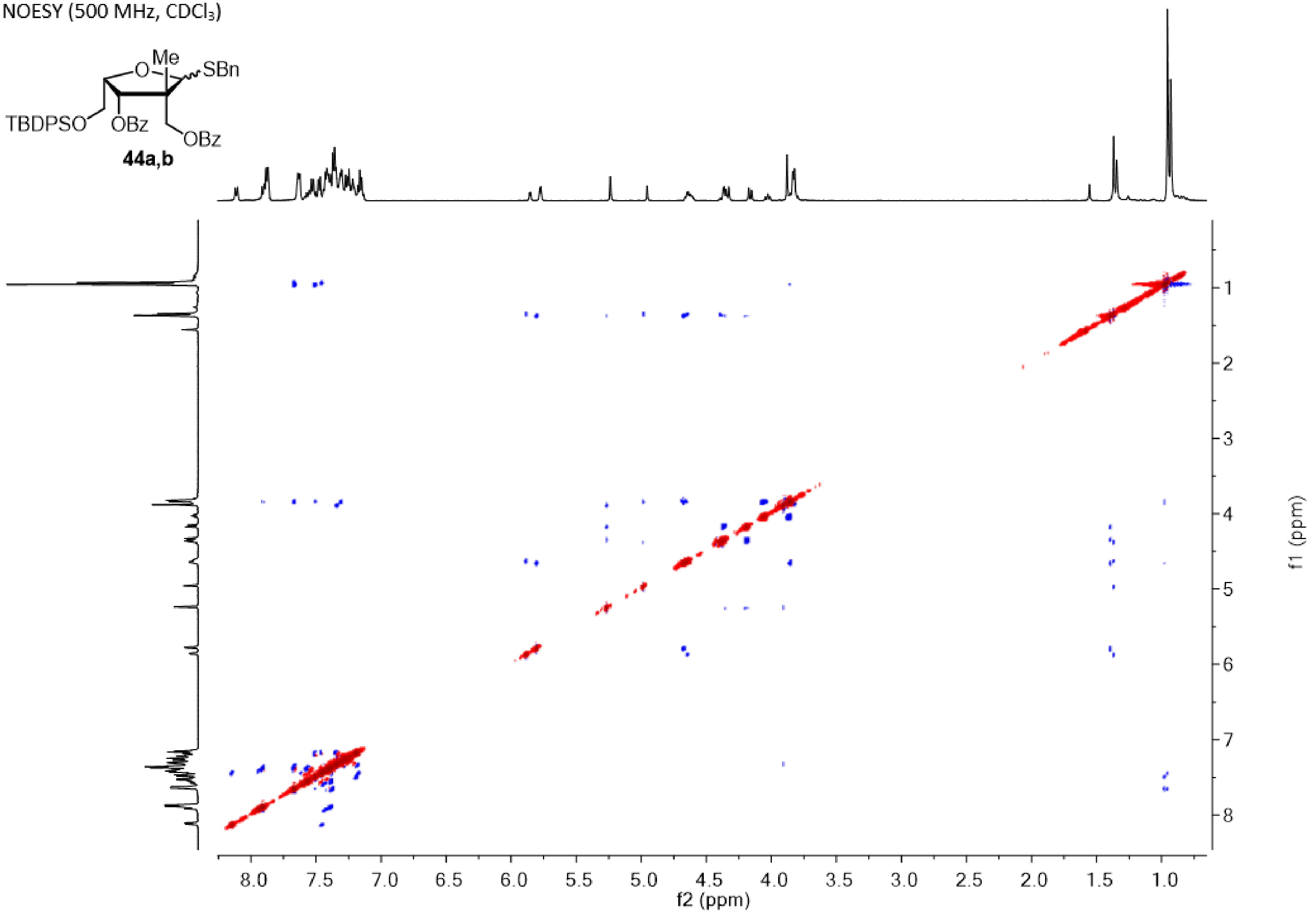

^{1}H-NMR (500 MHz, $CDCl_3$)

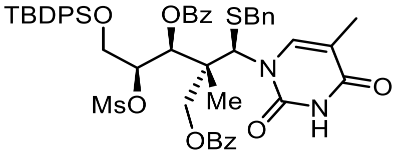

46a, 1',2'-*anti*

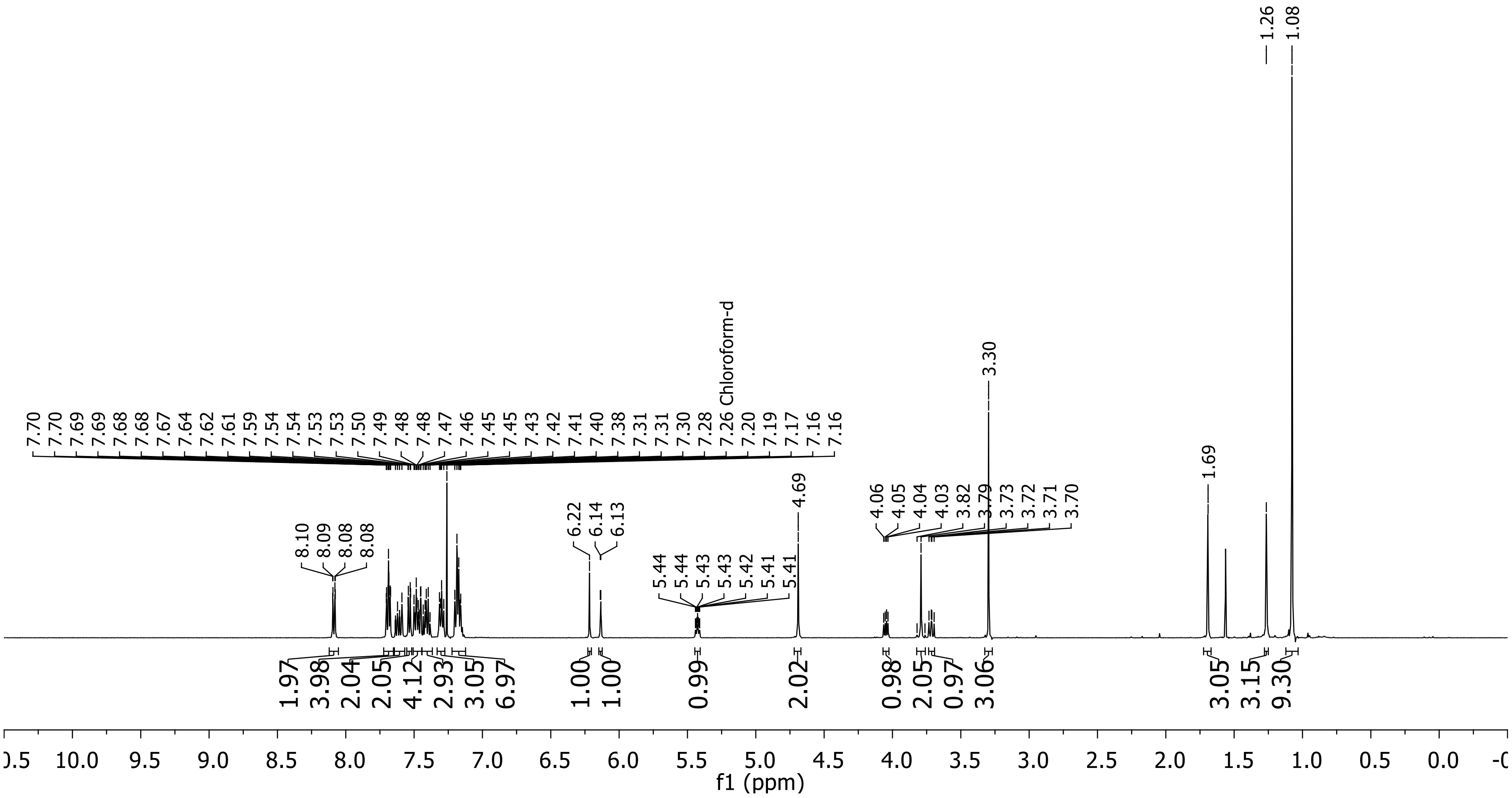

$^{13}C\{^{1}H\}$-NMR (126 MHz, $CDCl_3$)

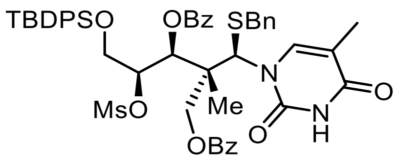

46a, 1',2'-*anti*

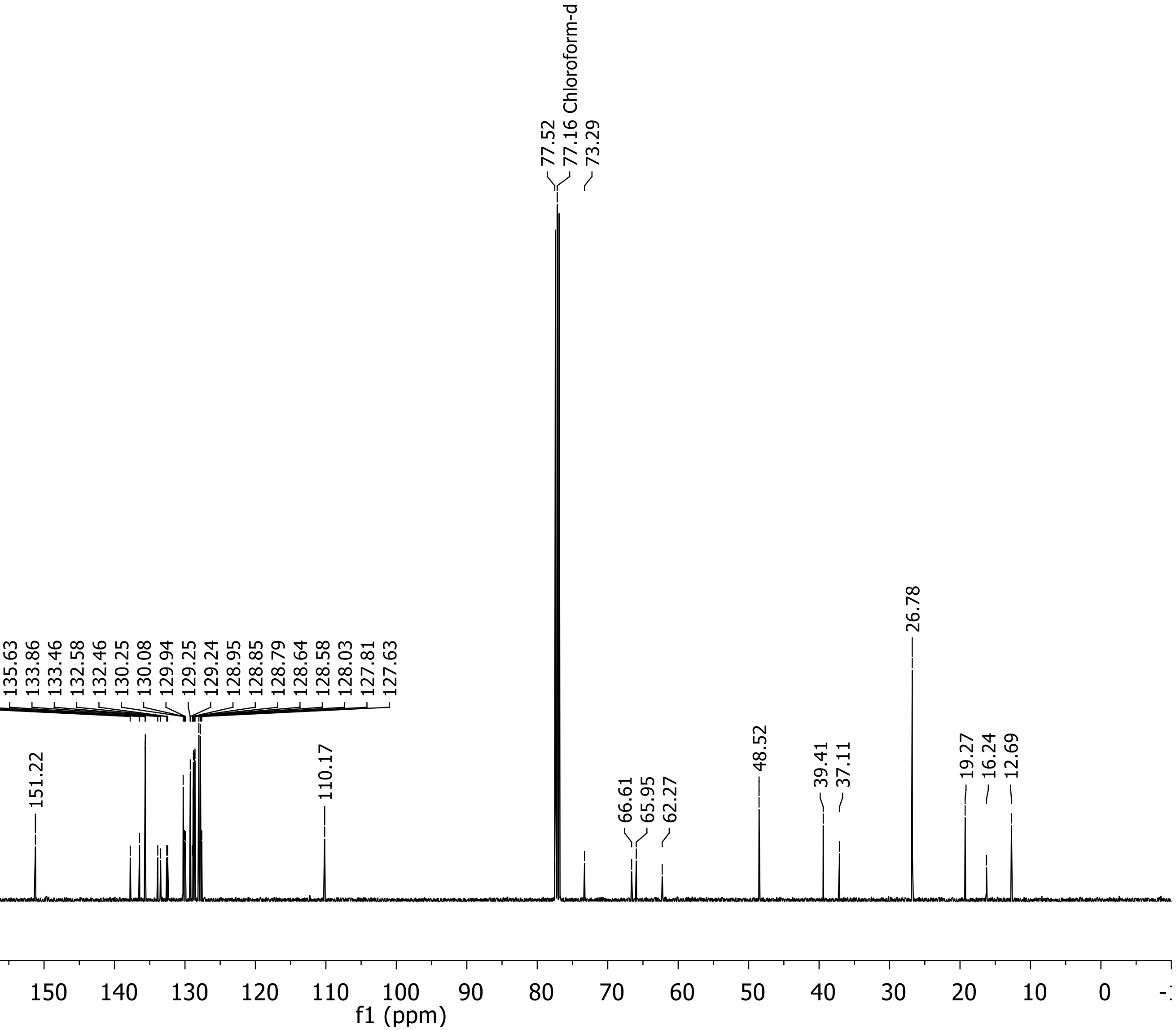

^{1}H-NMR (500 MHz, $CDCl_3$)

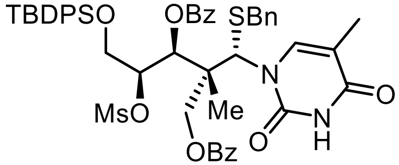

46b, 1',2'-*syn*

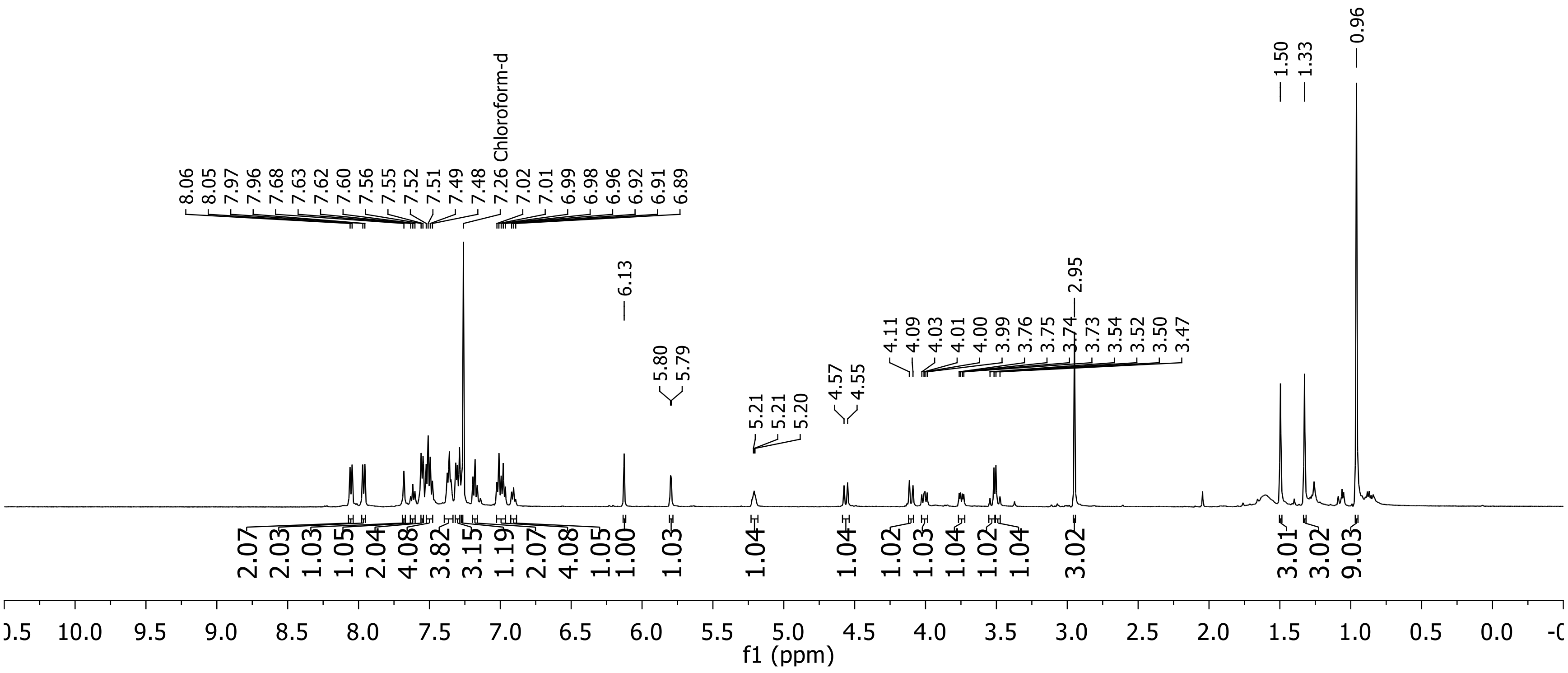

$^{13}C\{^1H\}$-NMR (126 MHz, $CDCl_3$)

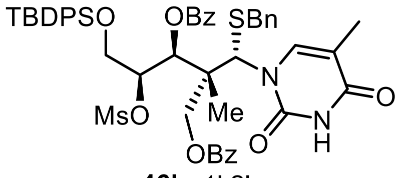

46b, 1',2'-*syn*

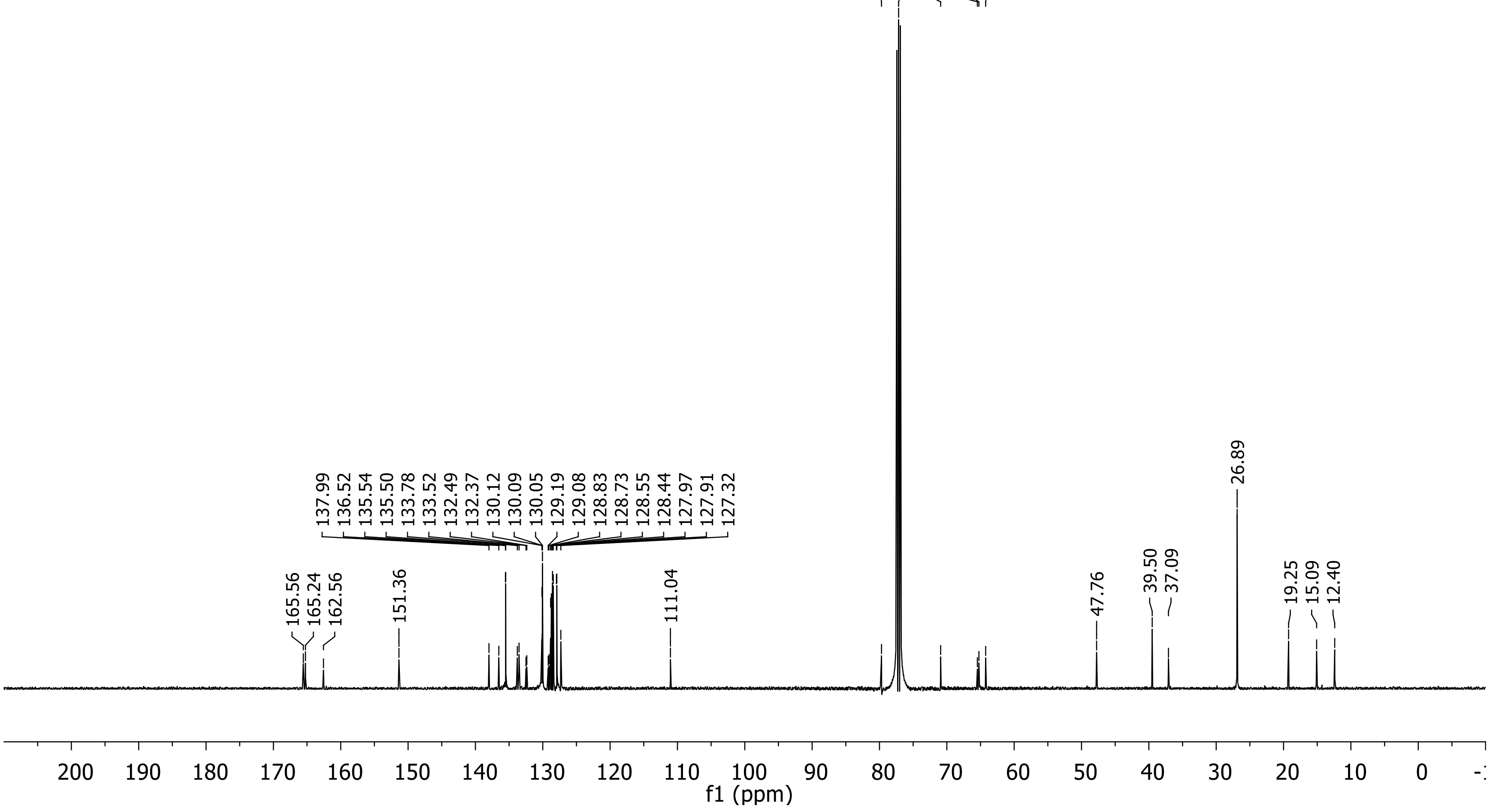

^{1}H-NMR (500 MHz, $CDCl_3$)

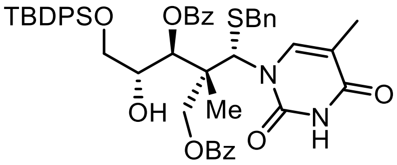

48, 1',2'-*syn*

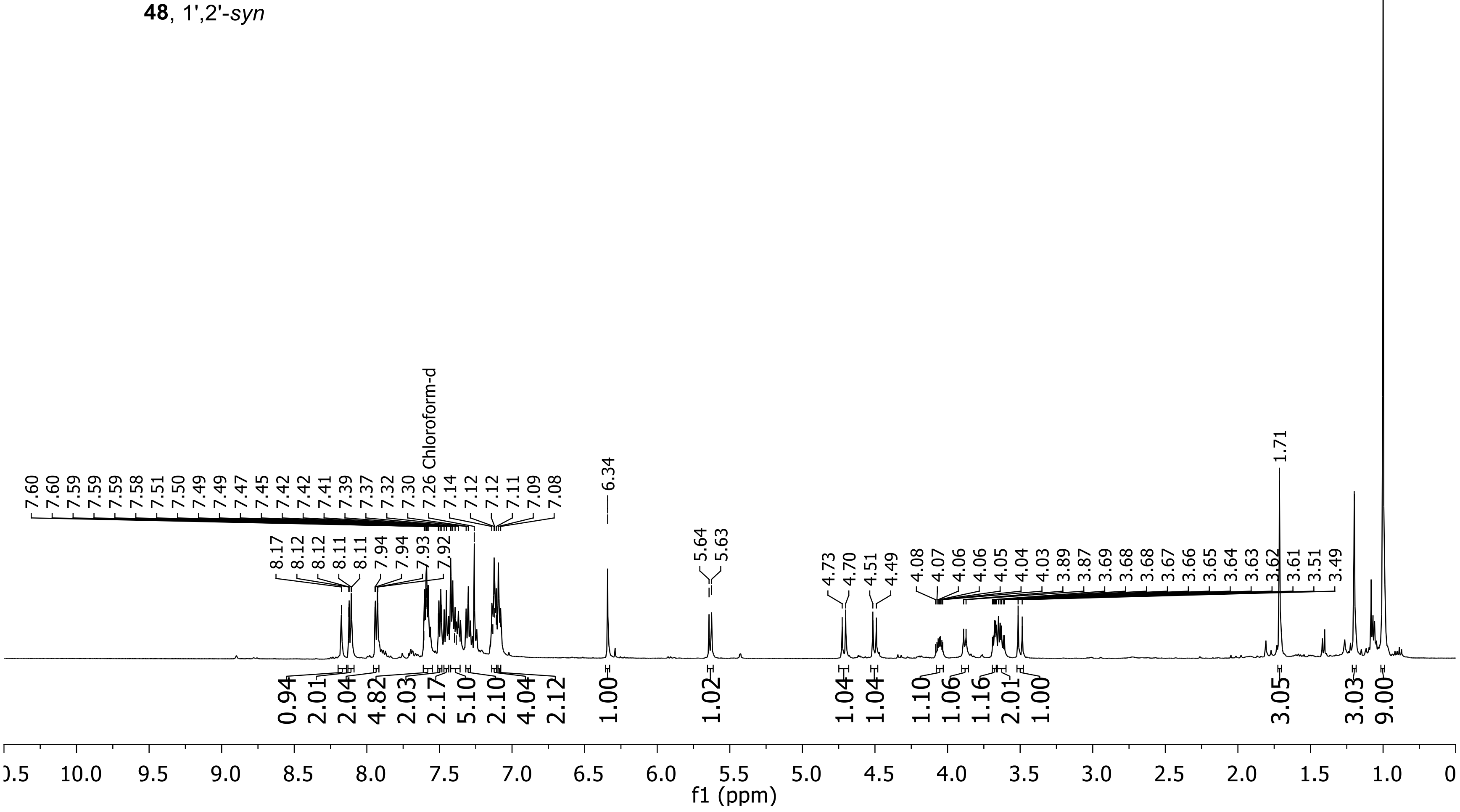

$^{13}C\{^{1}H\}$-NMR (126 MHz, $CDCl_3$)

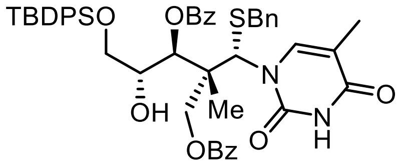

48, 1',2'-*syn*

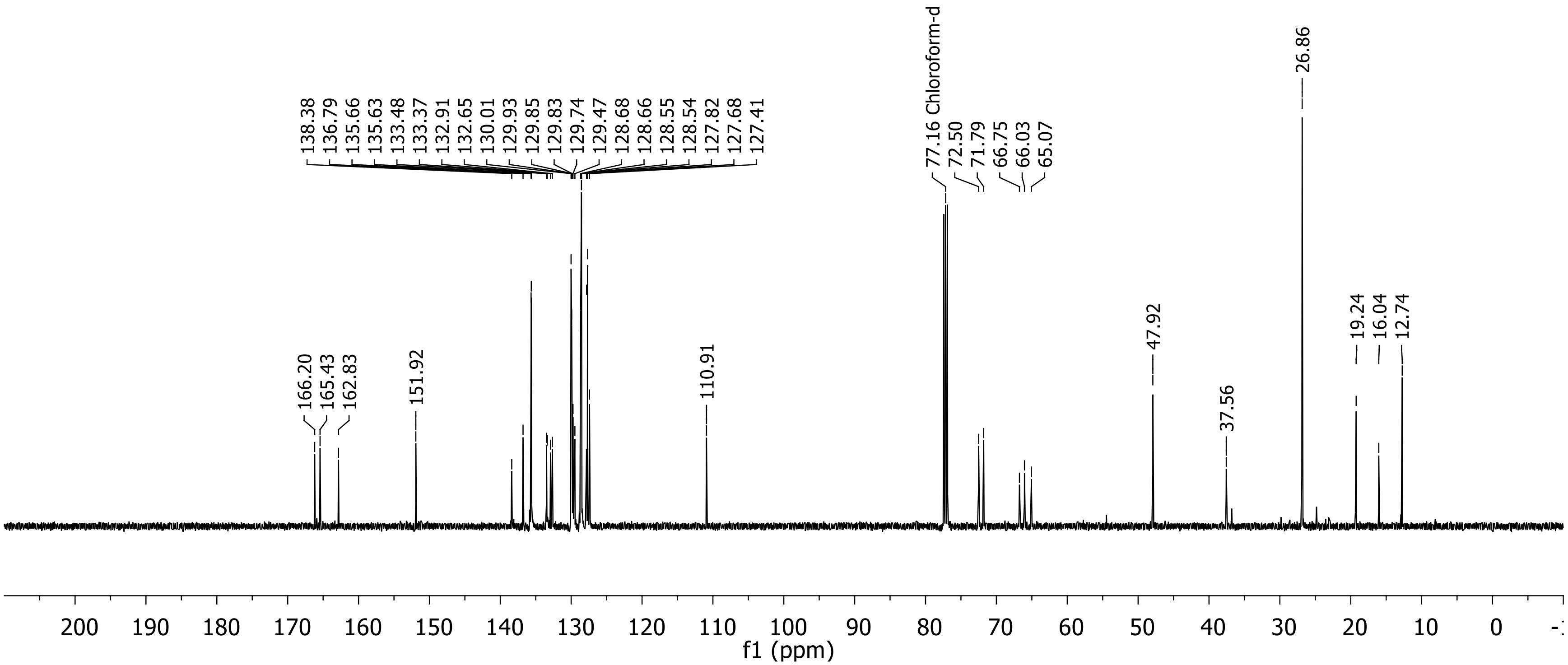

^{1}H-NMR (500 MHz, $CDCl_3$)

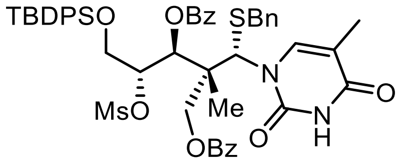

49

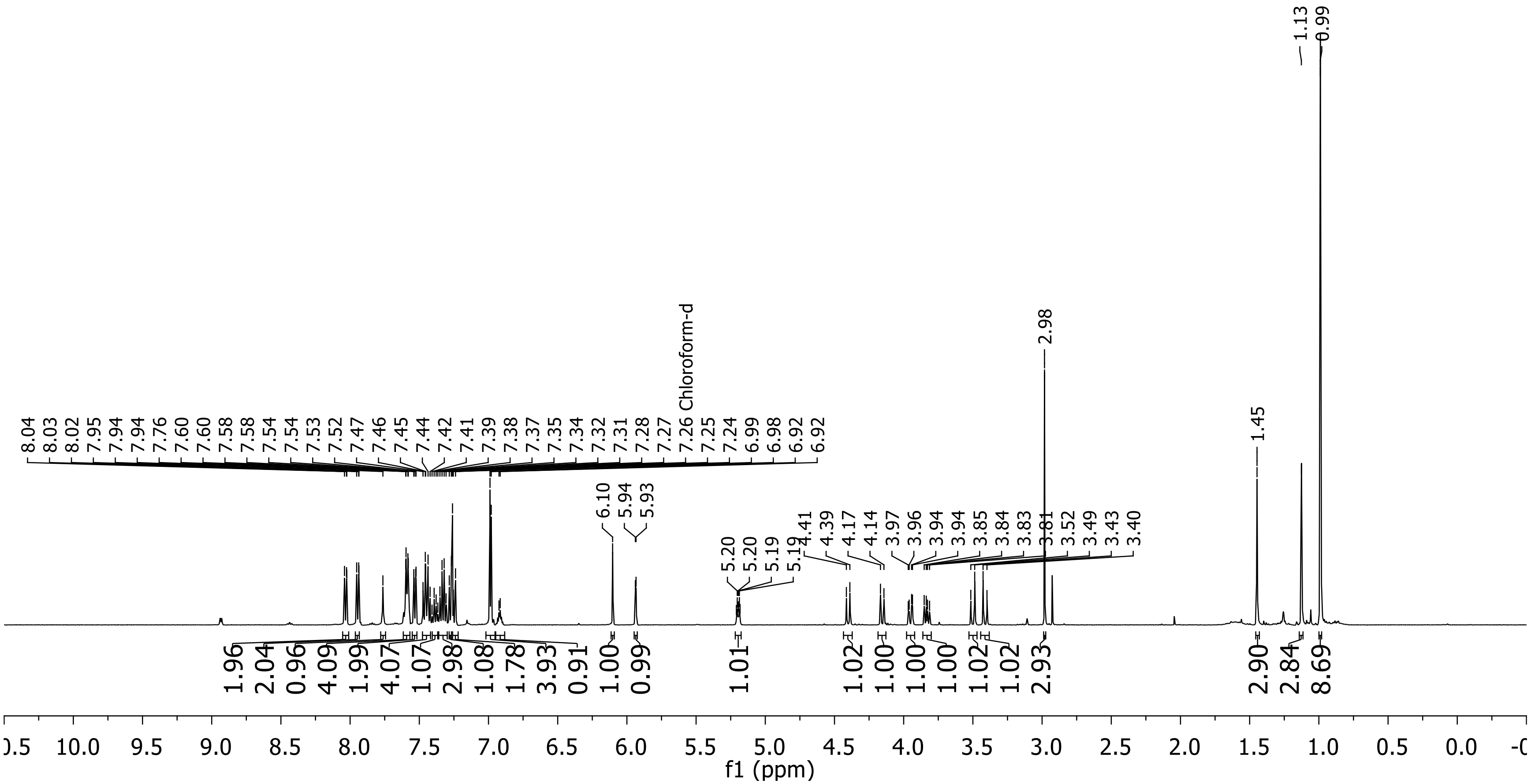

$^{13}C\{^{1}H\}$-NMR (126 MHz, $CDCl_3$)

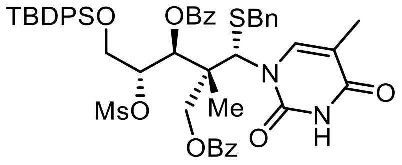

49

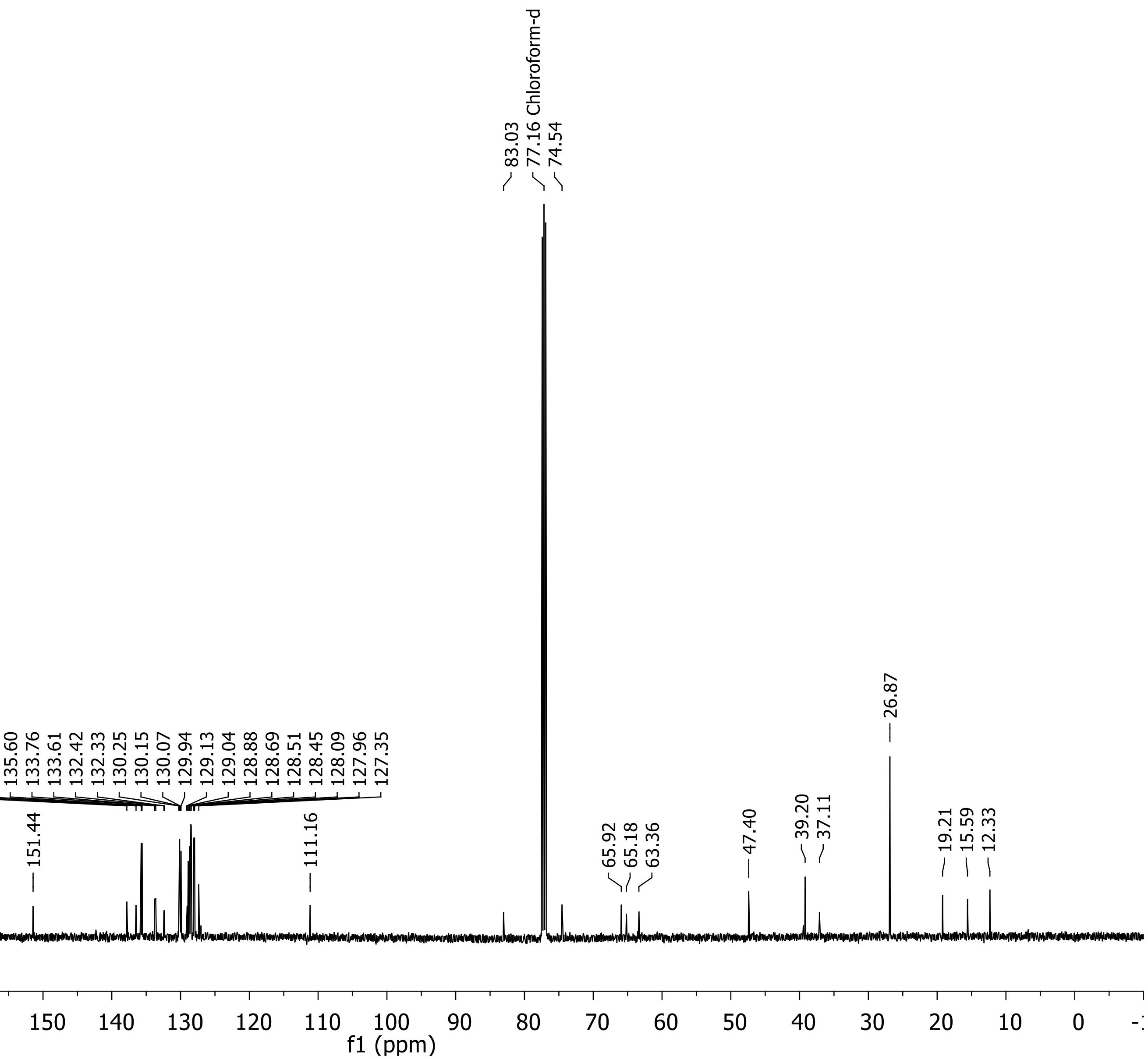

^{1}H-NMR (500 MHz, $CDCl_3$)

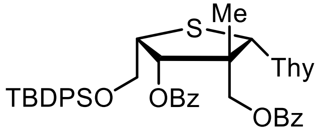

50

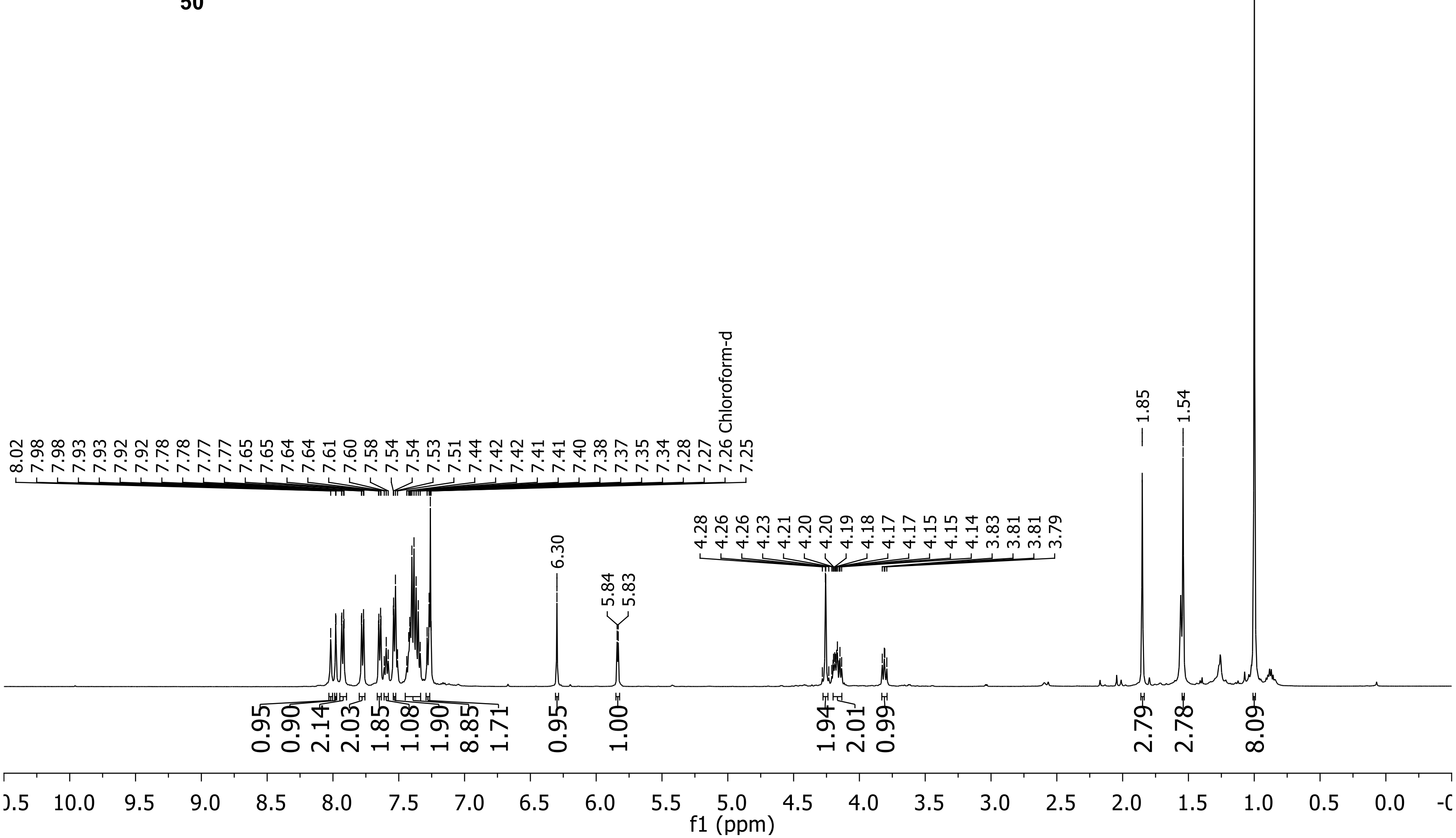

$^{13}C\{^{1}H\}$-NMR (126 MHz, $CDCl_3$)

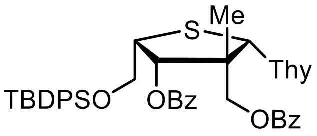

50

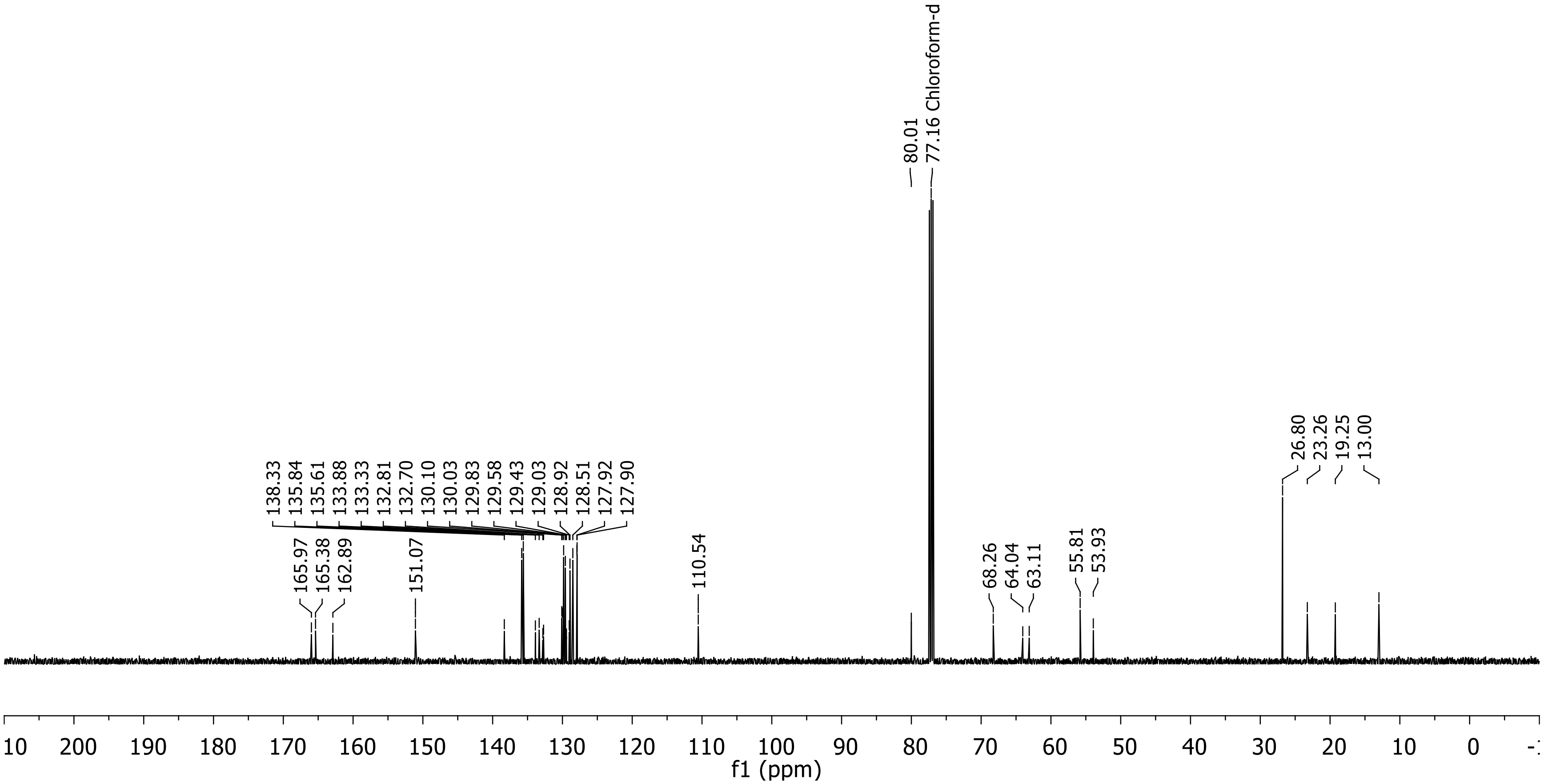

COSY (500 MHz, $CDCl_3$)

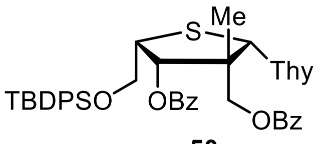

50

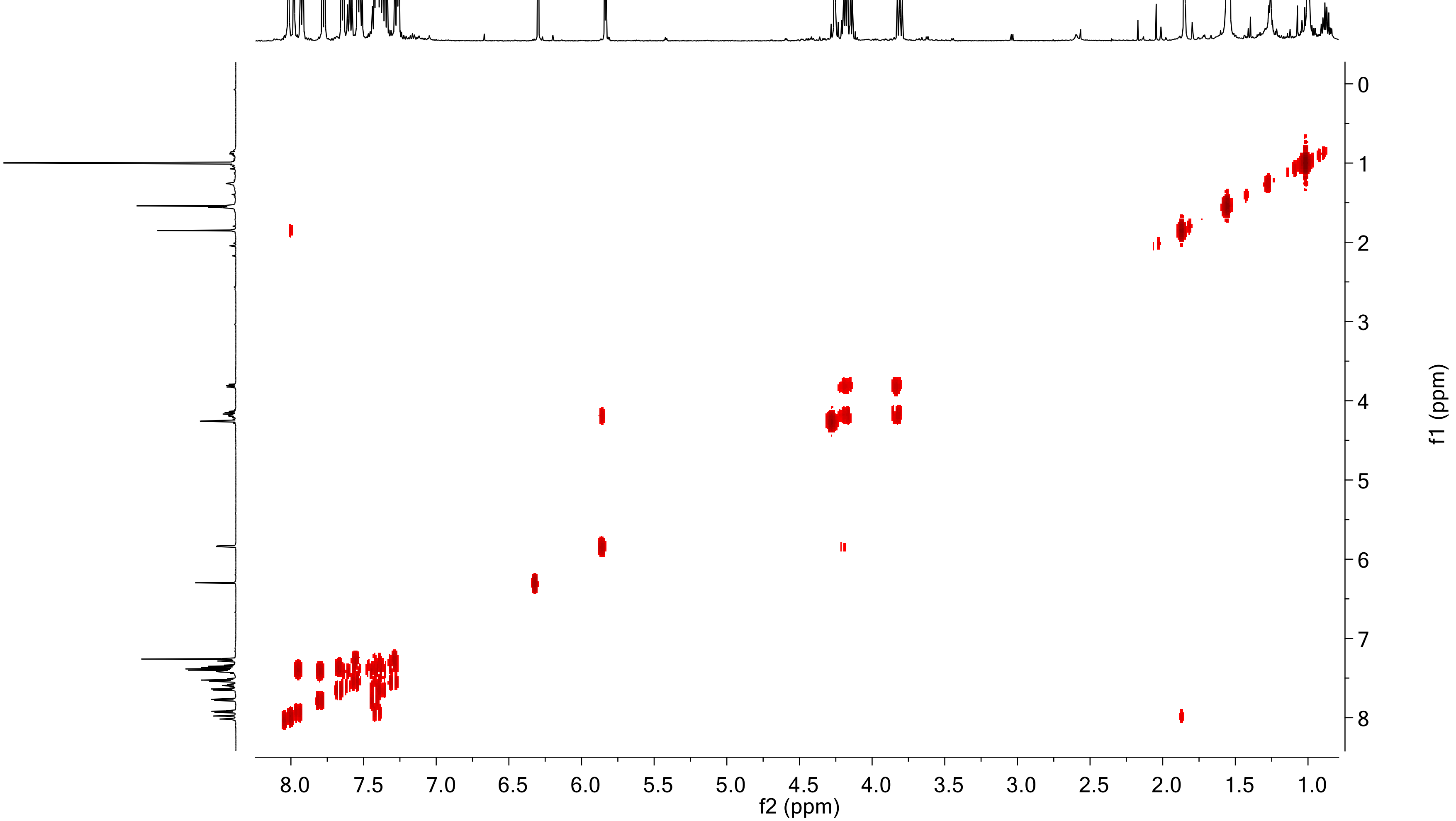

HSQC (500 MHz, $CDCl_3$)

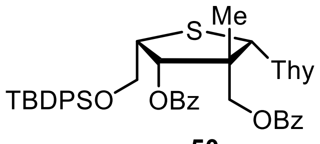

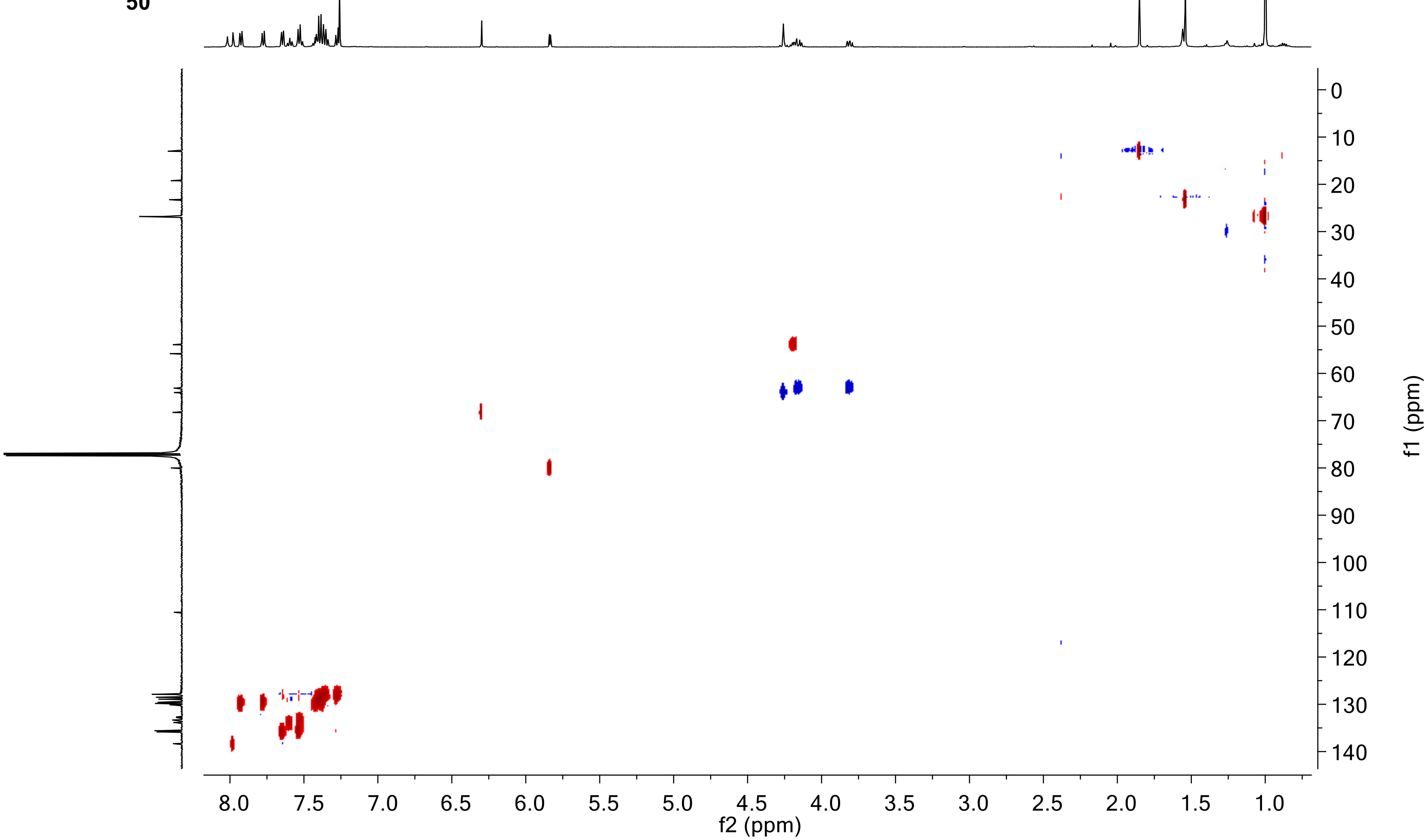

NOESY (500 MHz, $CDCl_3$)

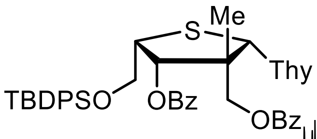

50

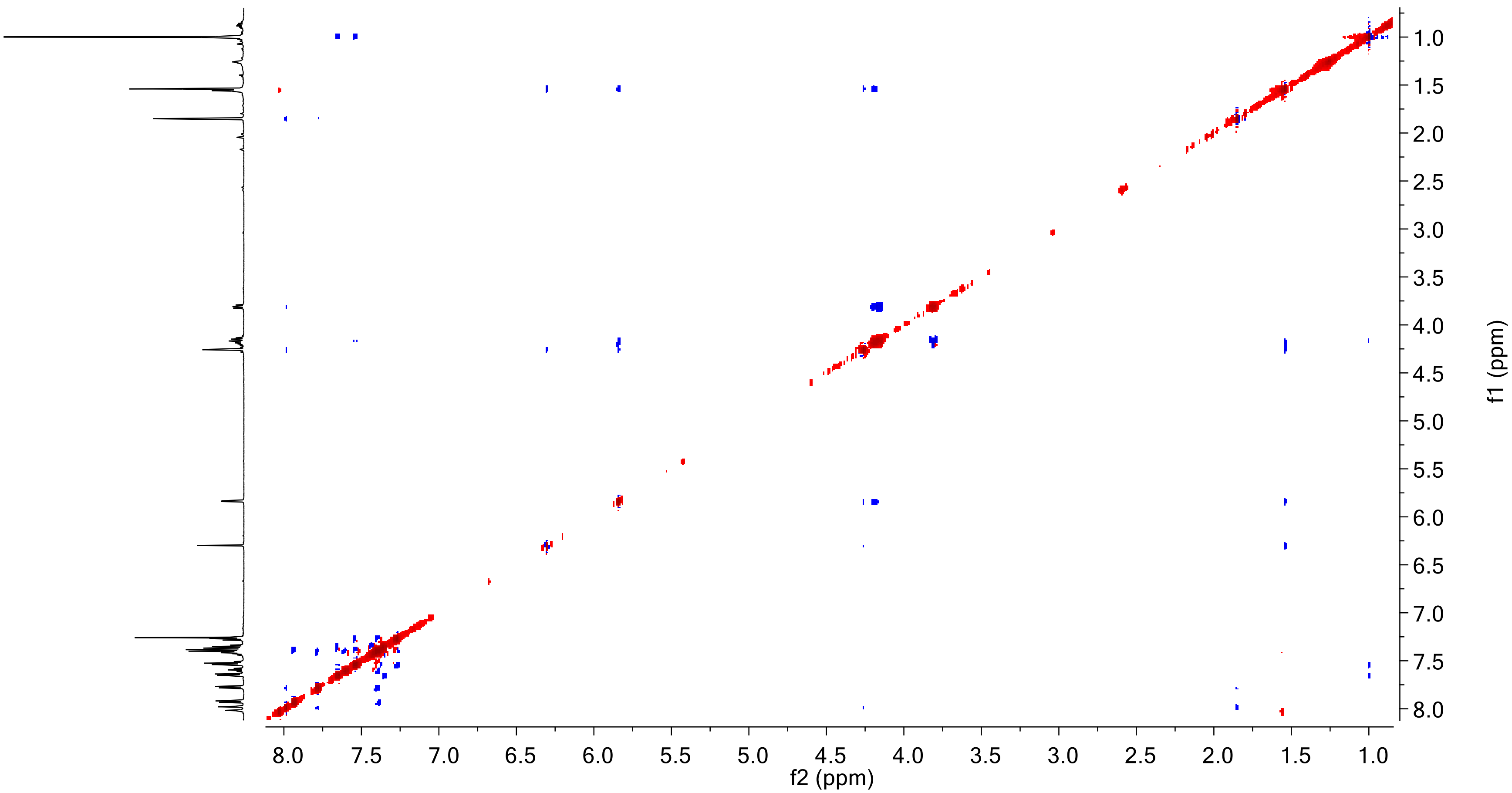

^{1}H-NMR (500 MHz, $CDCl_3$)

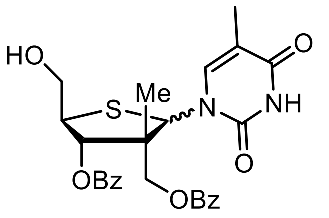

61a,b

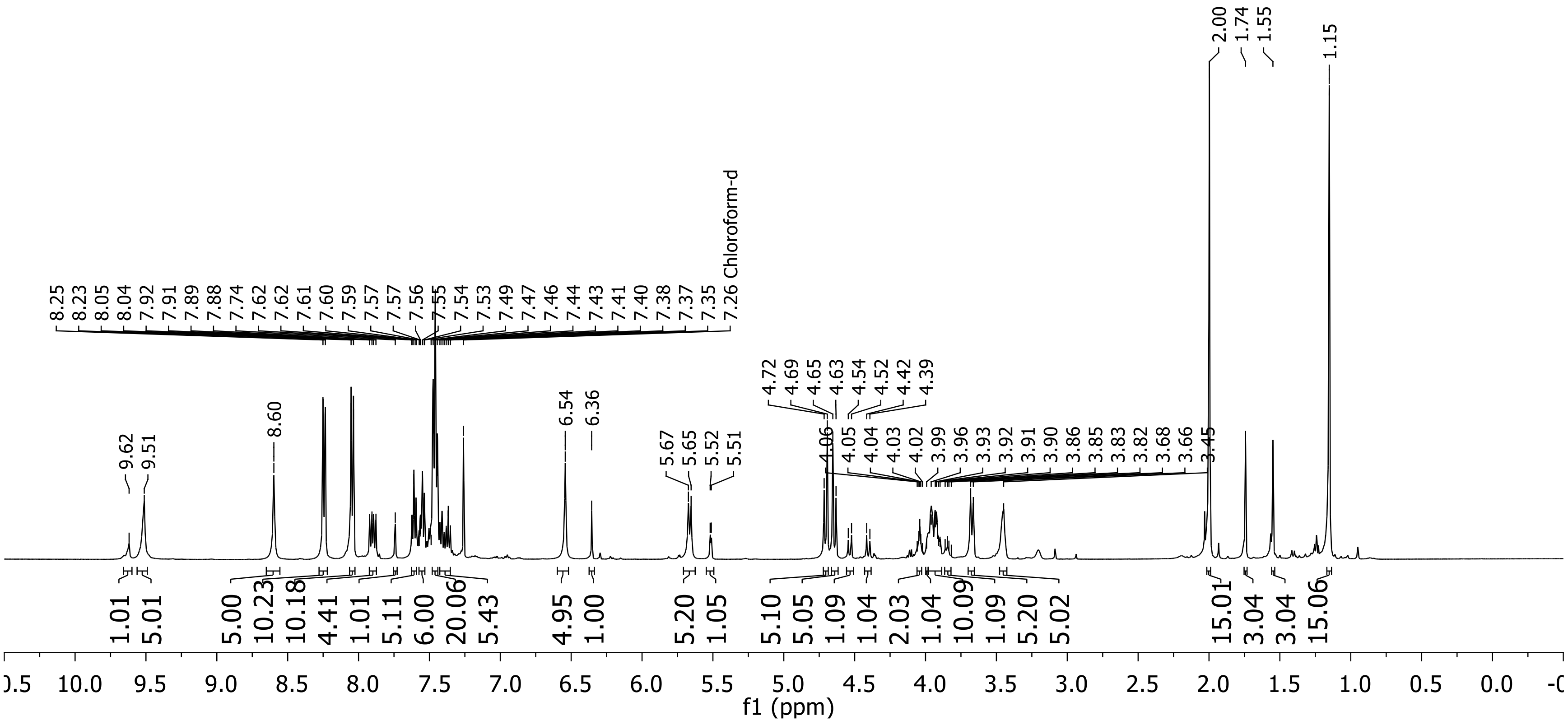

$^{13}C\{^1H\}$-NMR (126 MHz, $CDCl_3$)

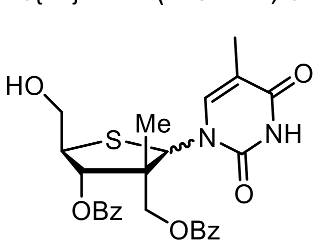

61a,b

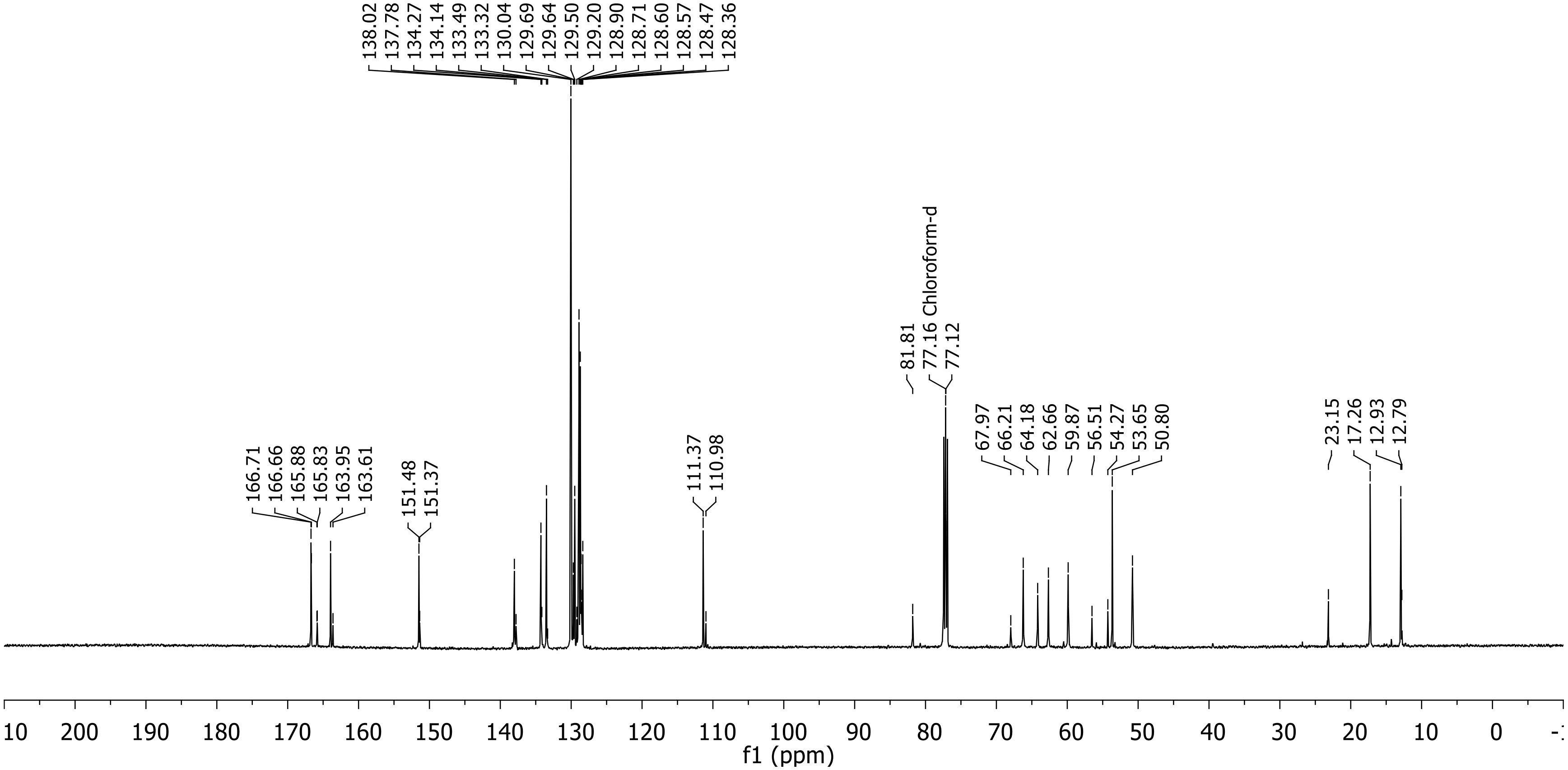

COSY (500 MHz, $CDCl_3$)

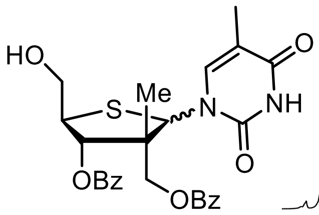

61a,b

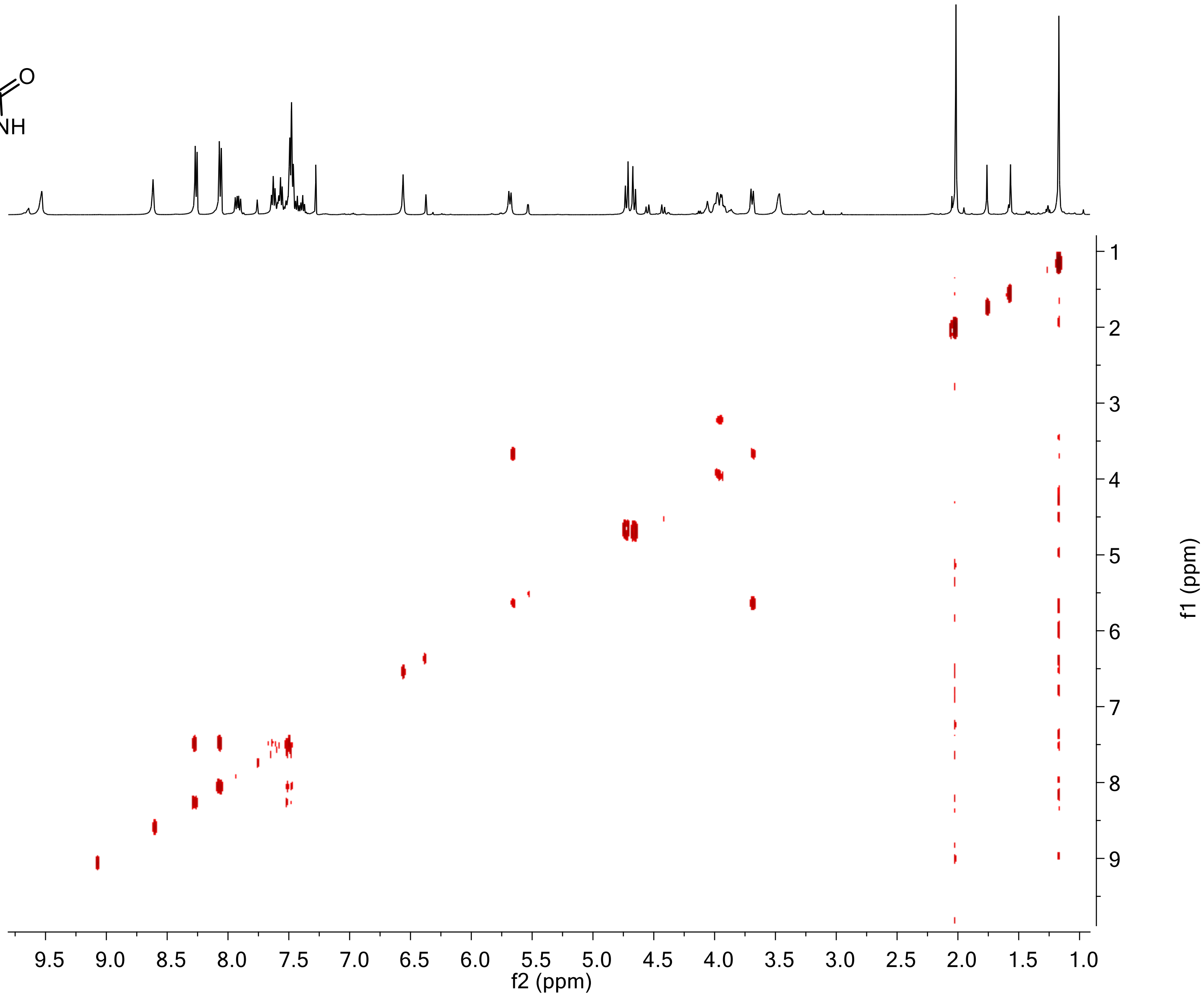

HSQC (500 MHz, $CDCl_3$)

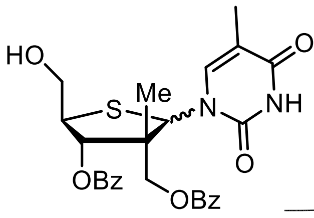

61a,b

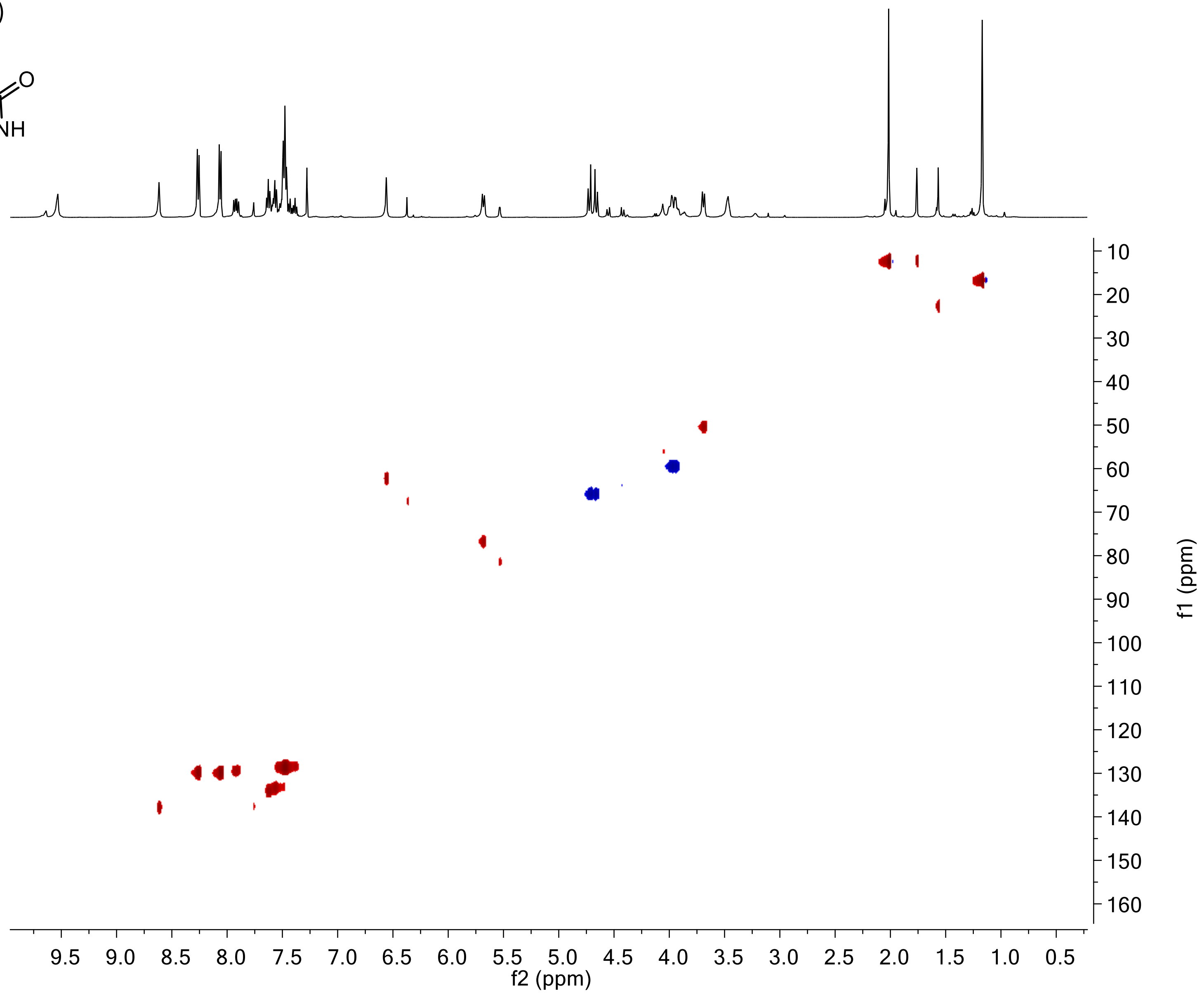

NOESY (500 MHz, $CDCl_3$)

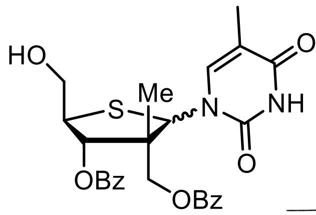

61a,b

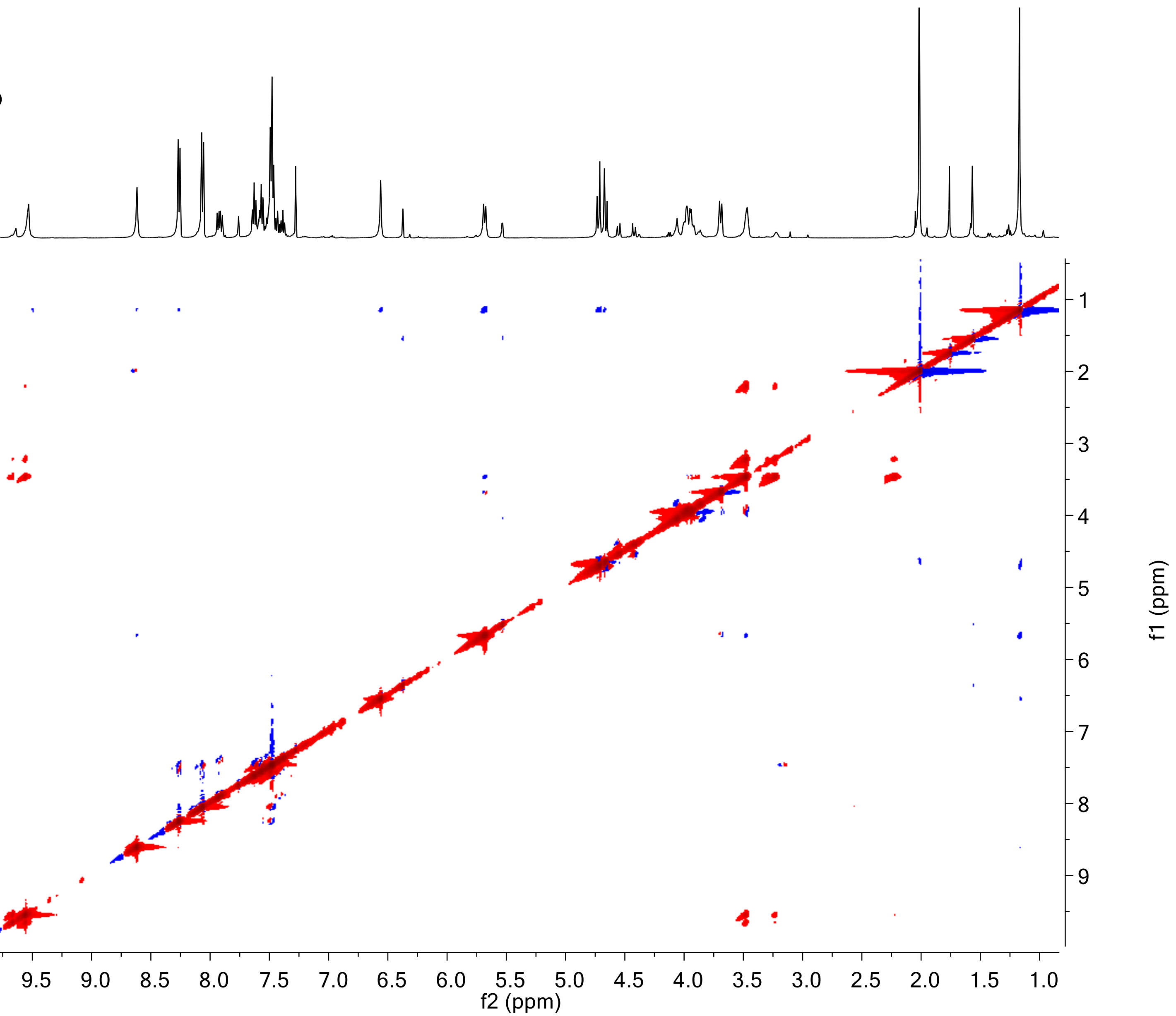

^{1}H-NMR (500 MHz, CD_3OD)

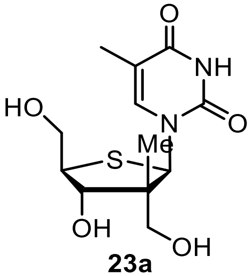

23a

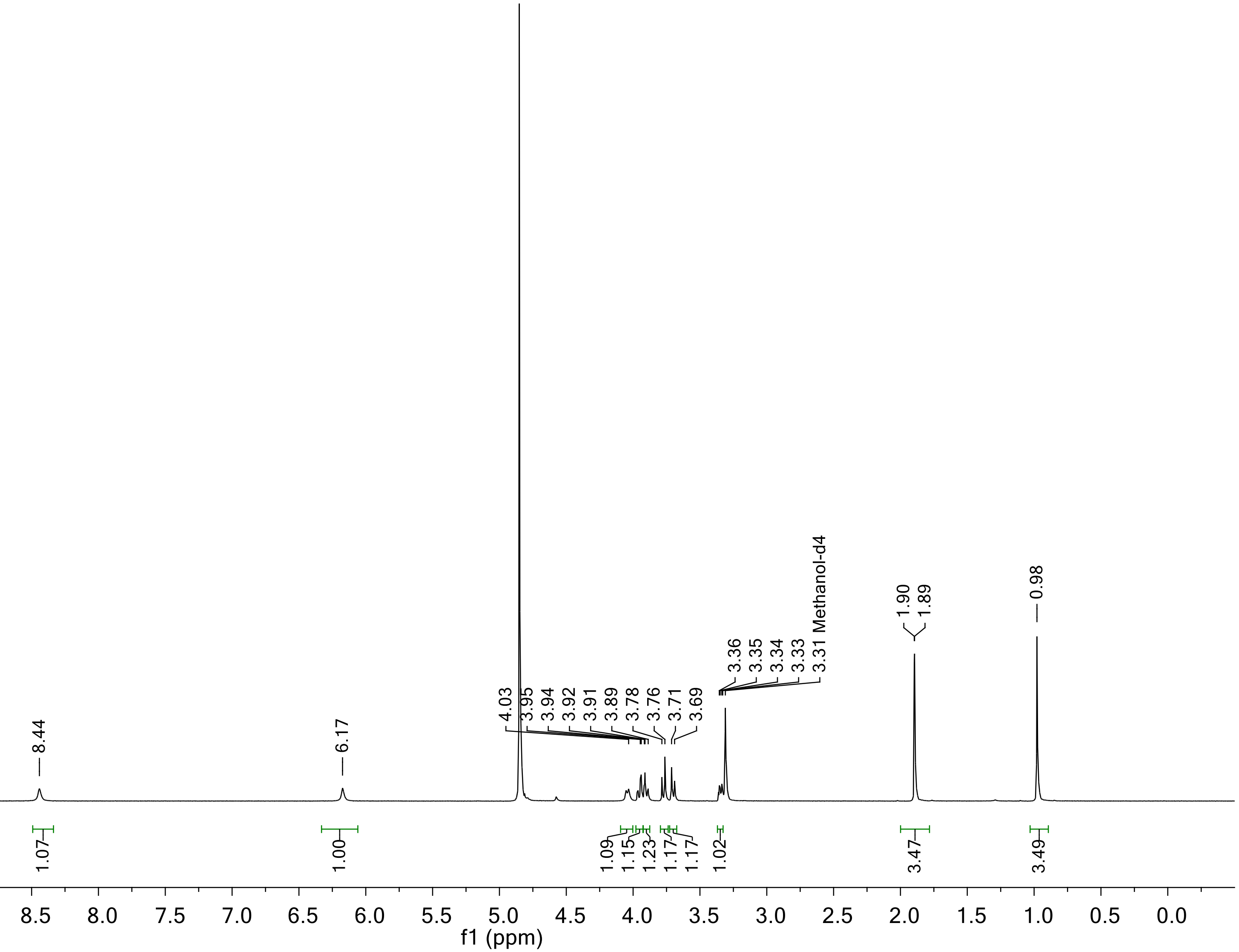

$^{13}C\{^{1}H\}$-NMR (126 MHz, CD_3OD)

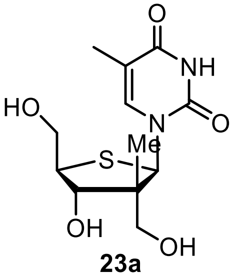

23a

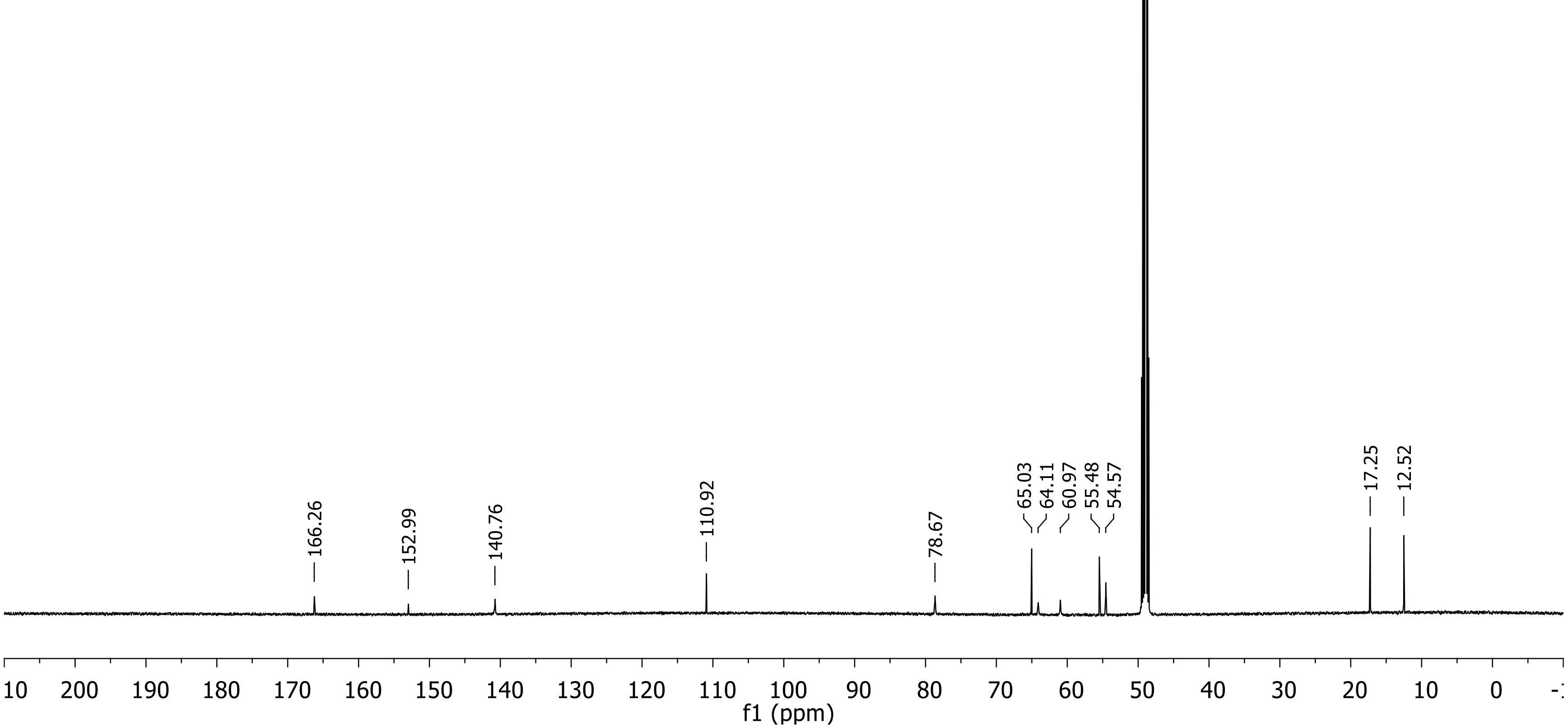

COSY (500 MHz, CD_3OD)

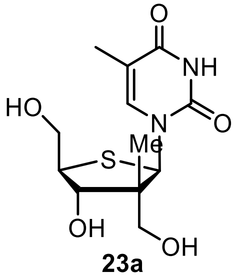

23a

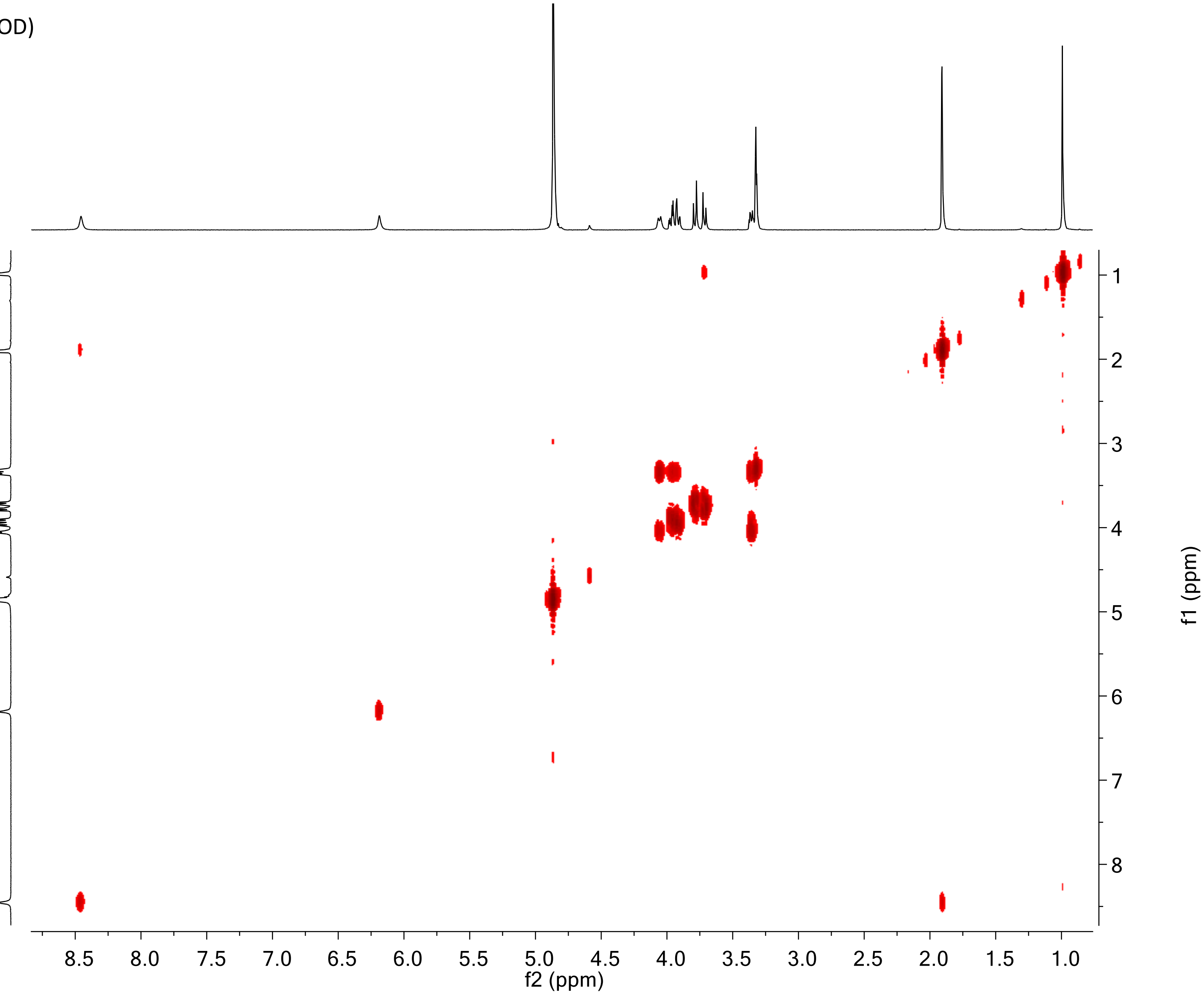

NOESY (500 MHz, CD_3OD)

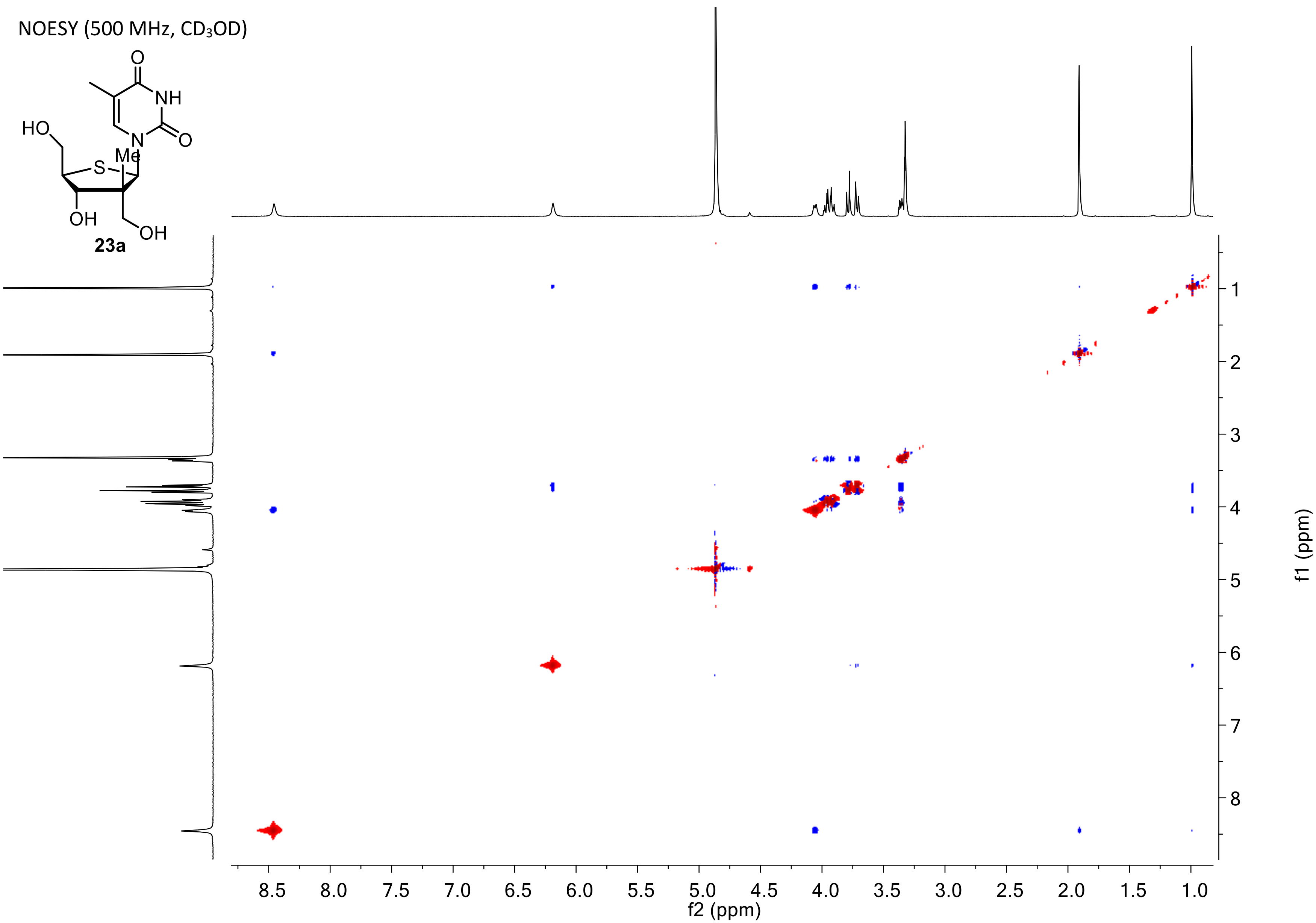

^{1}H-NMR (500 MHz, CD_3OD)

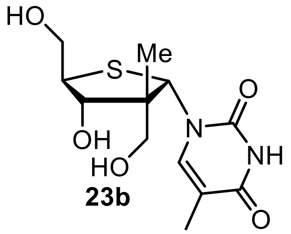

23b

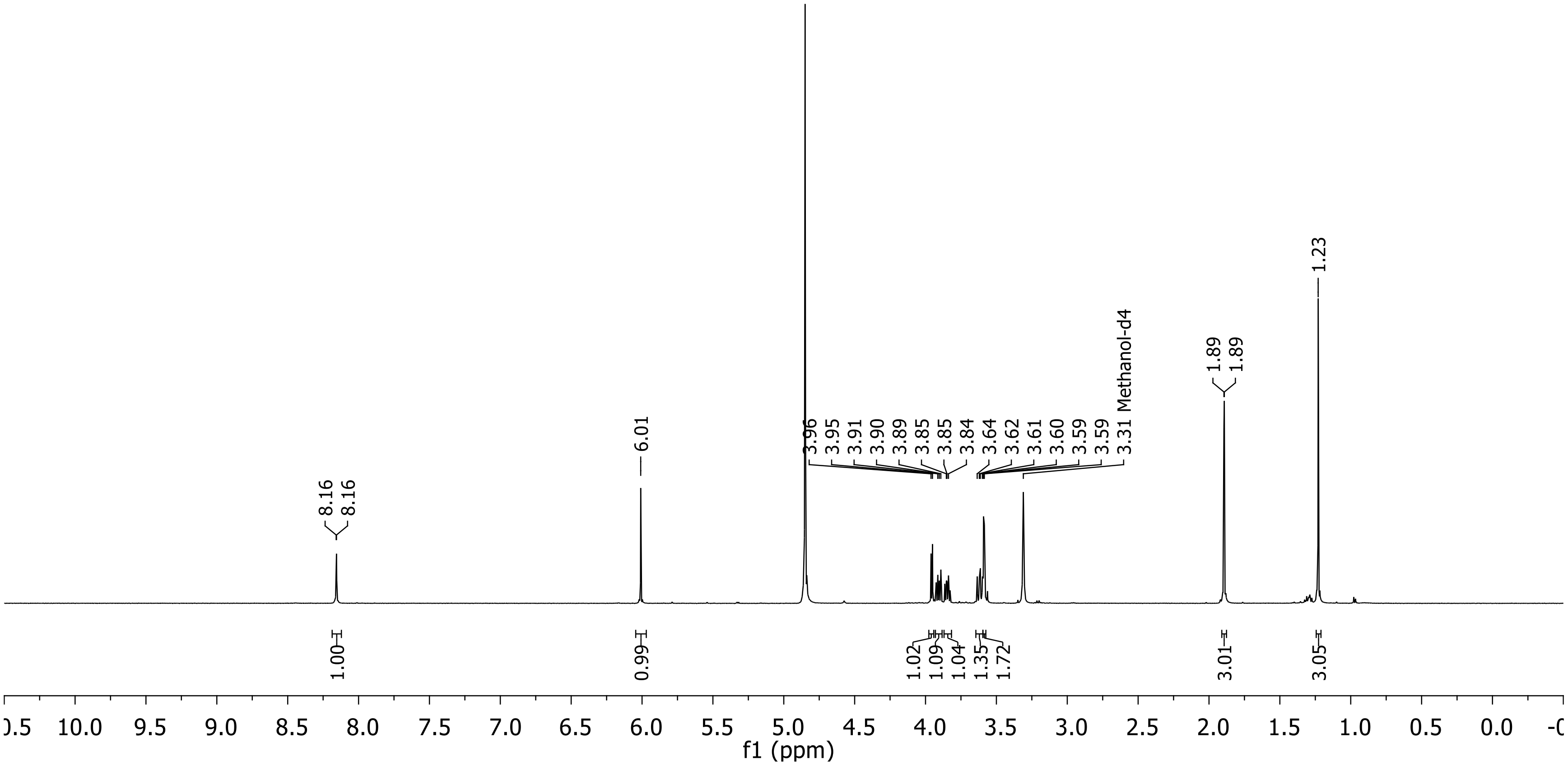

$^{13}C\{^{1}H\}$-NMR (126 MHz, CD_3OD)

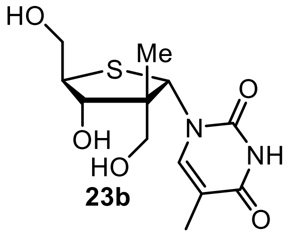

23b

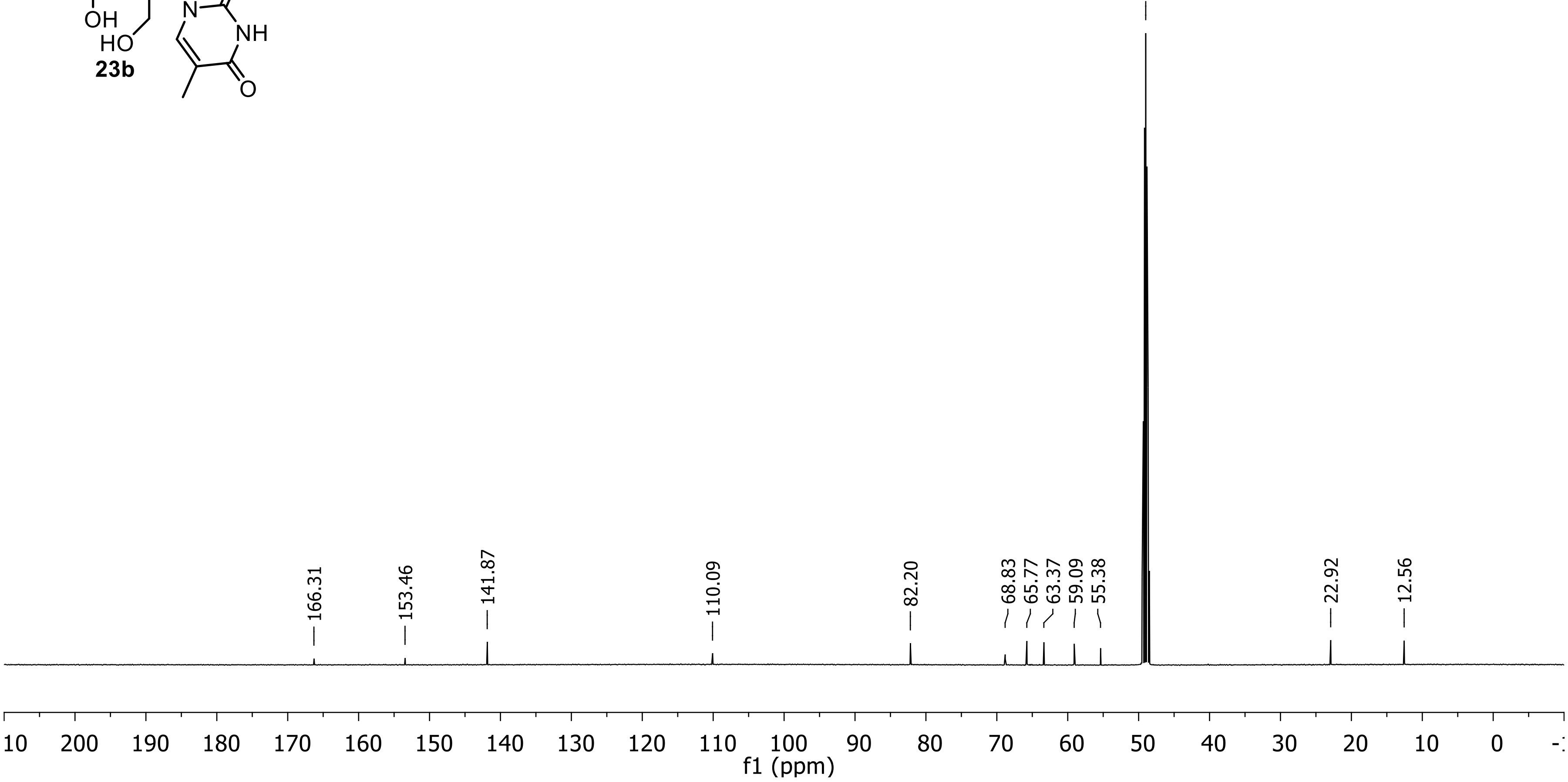

COSY (500 MHz, CD_3OD)

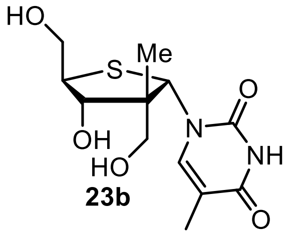

23b

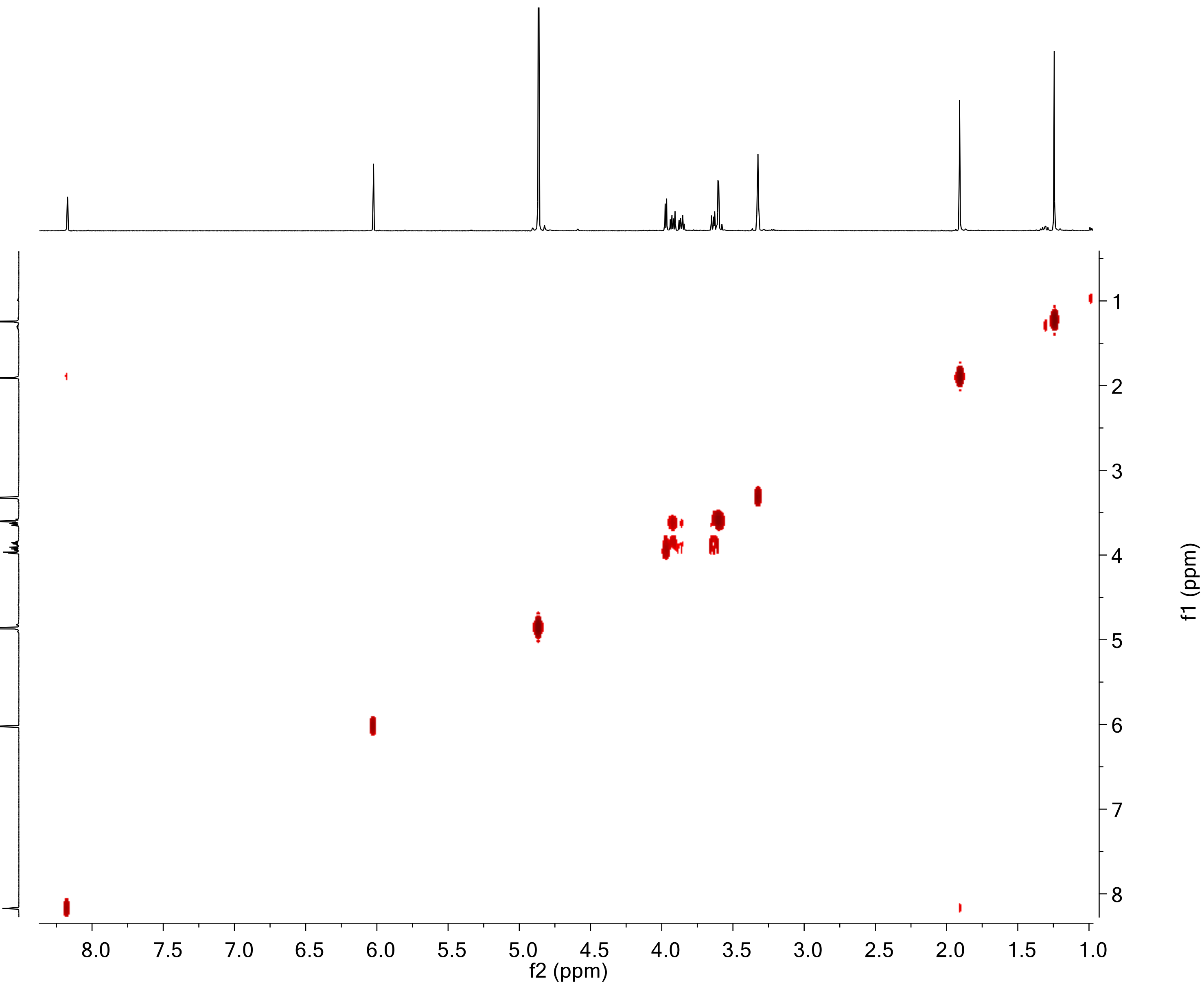

NOESY (500 MHz, CD_3OD)

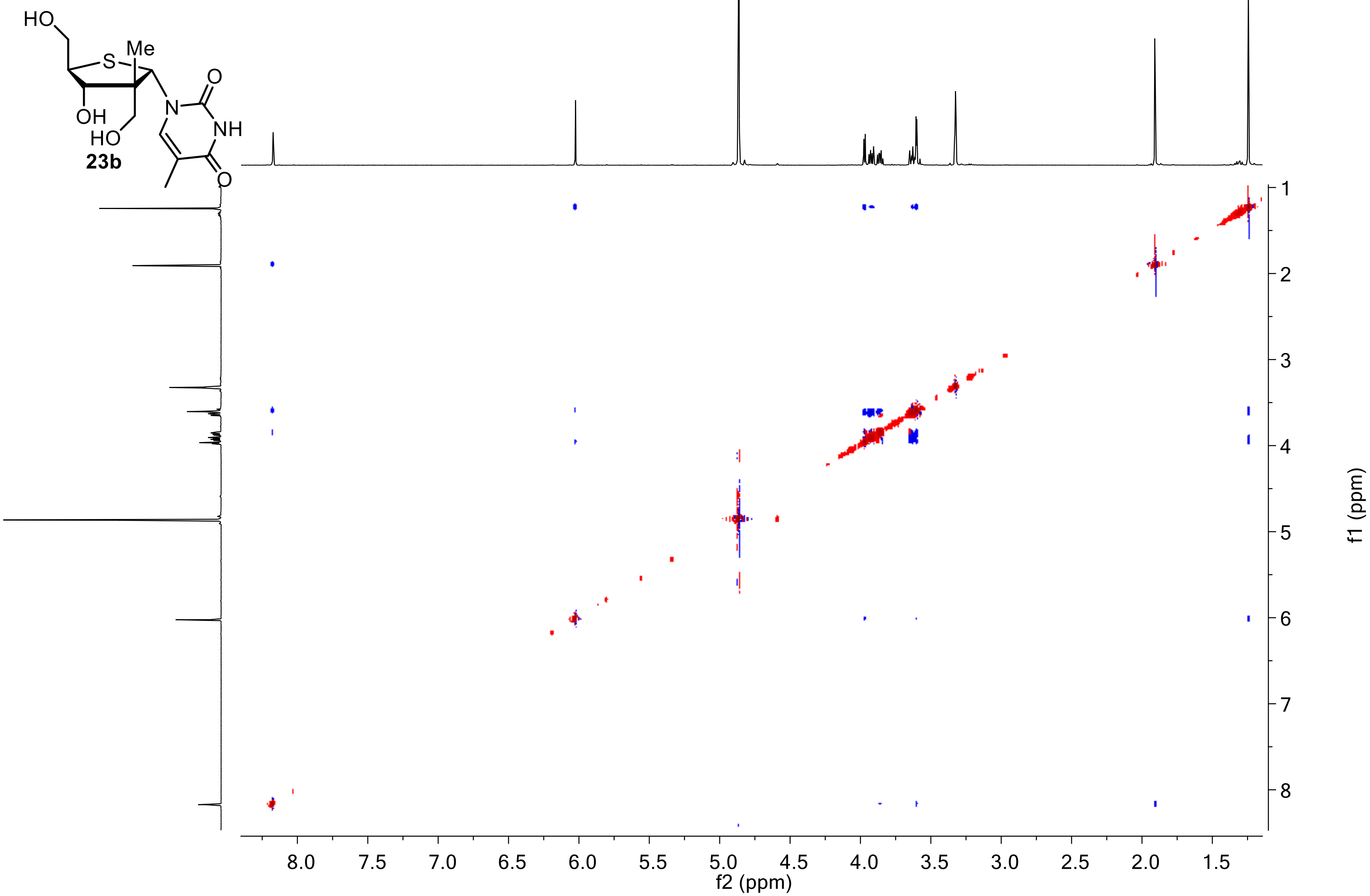

^{1}H-NMR (500 MHz, $CDCl_3$)

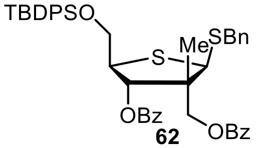

62

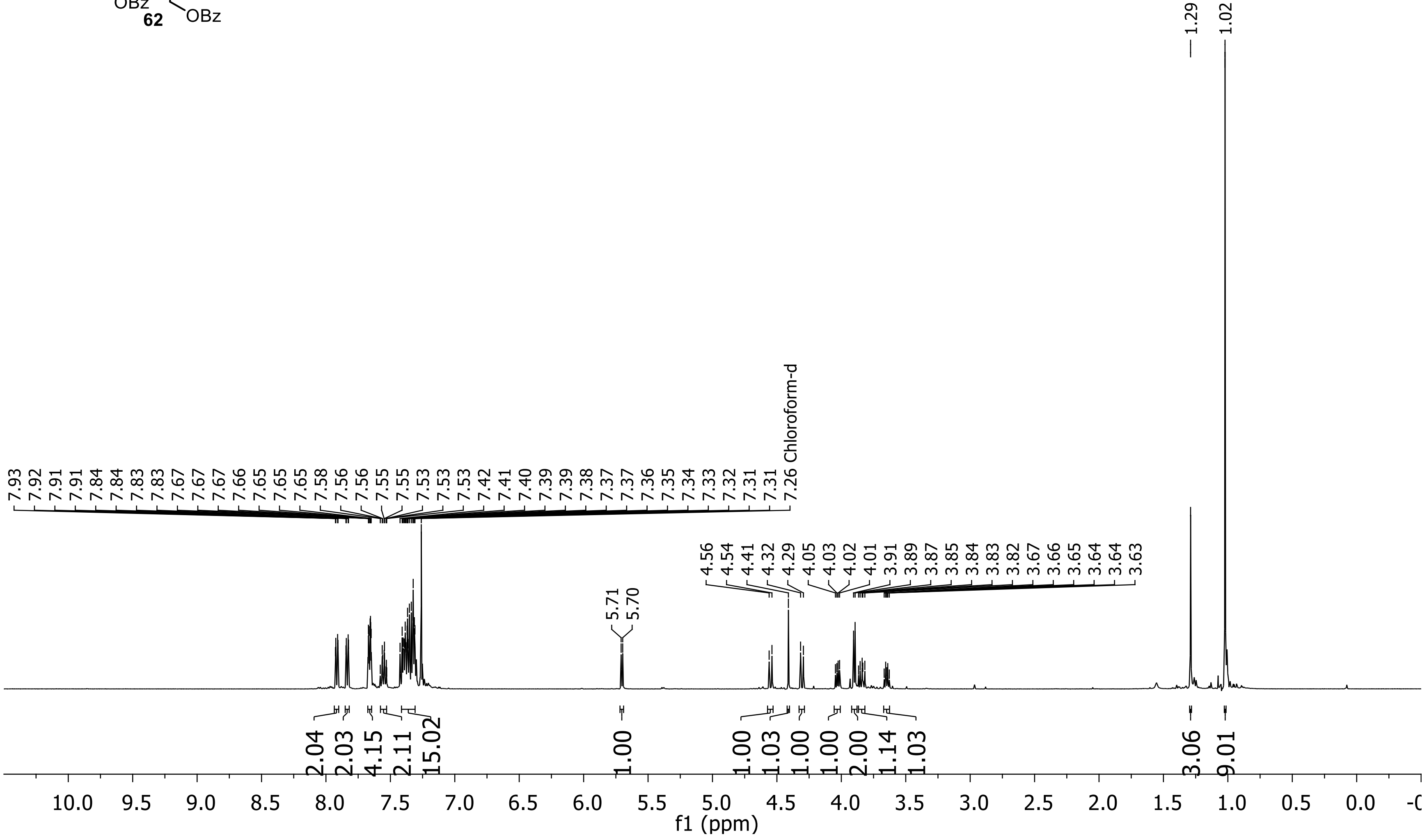

$^{13}C\{^{1}H\}$-NMR (126 MHz, $CDCl_3$)

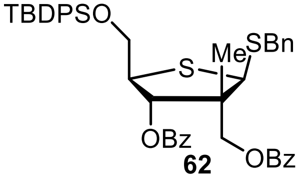

62

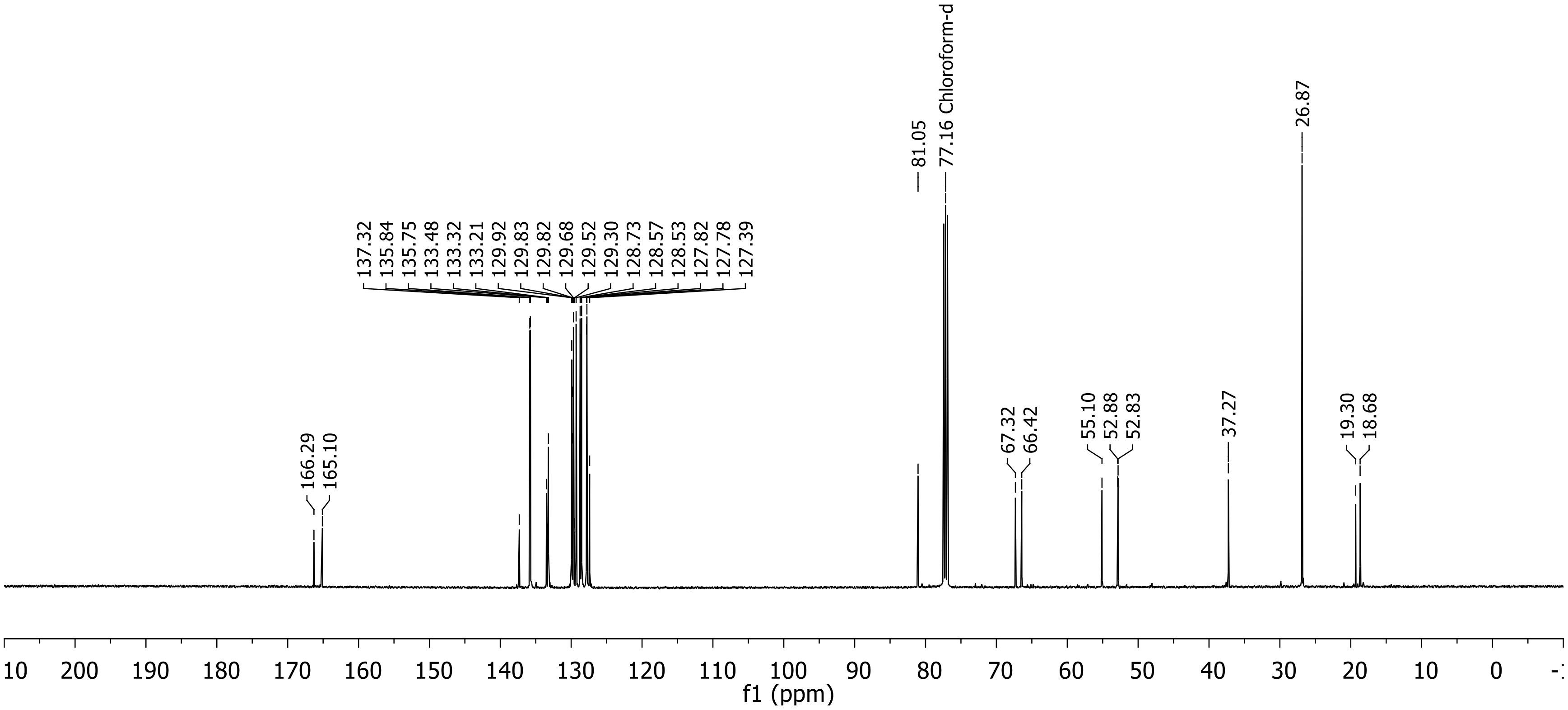

HSQC (500 MHz, $CDCl_3$)

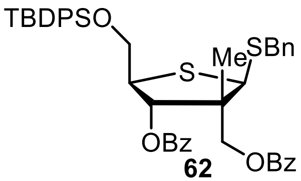

62

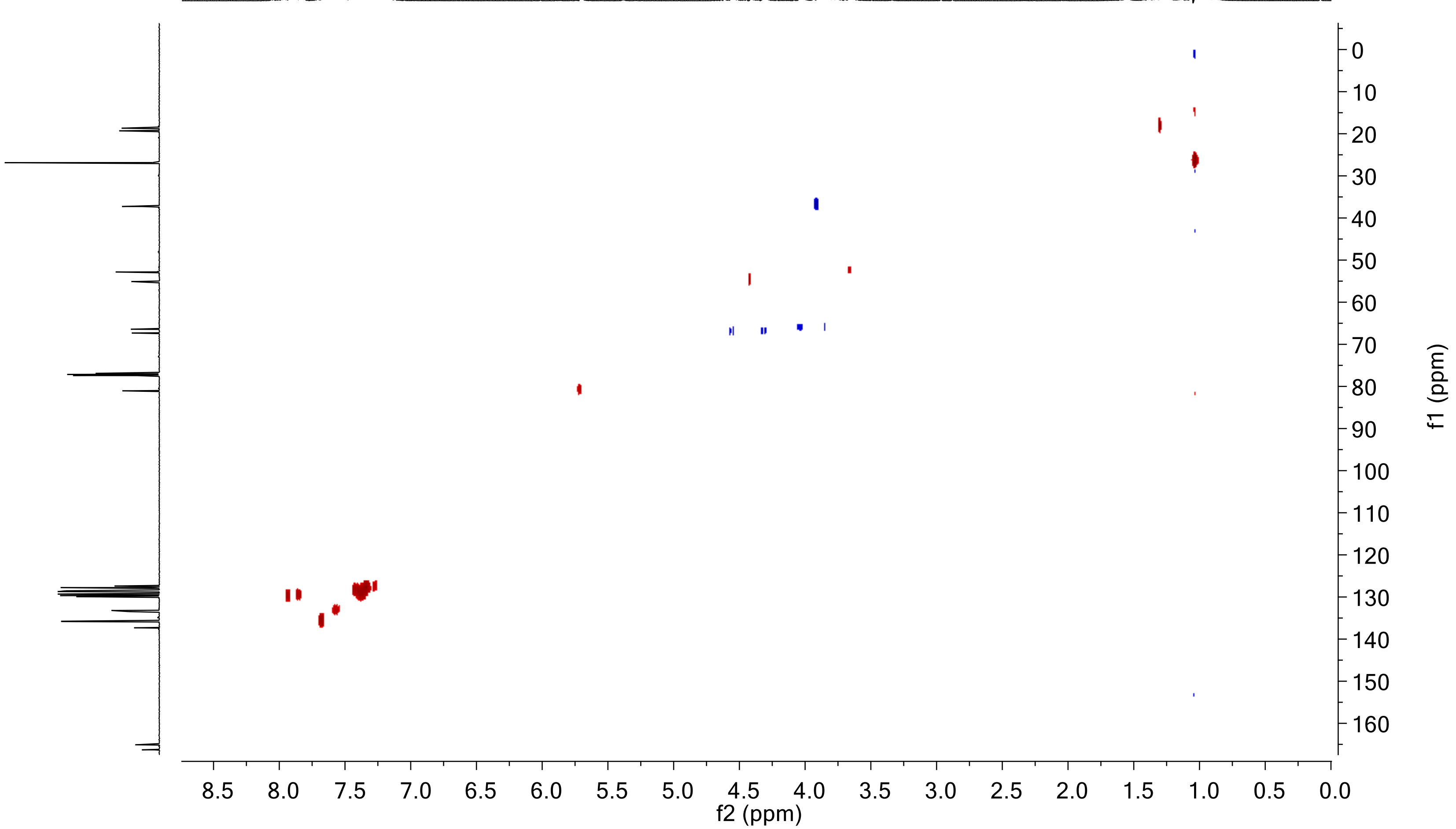

NOESY (500 MHz, $CDCl_3$)

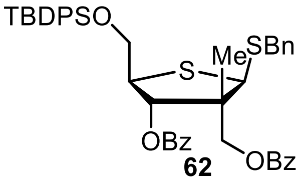

62

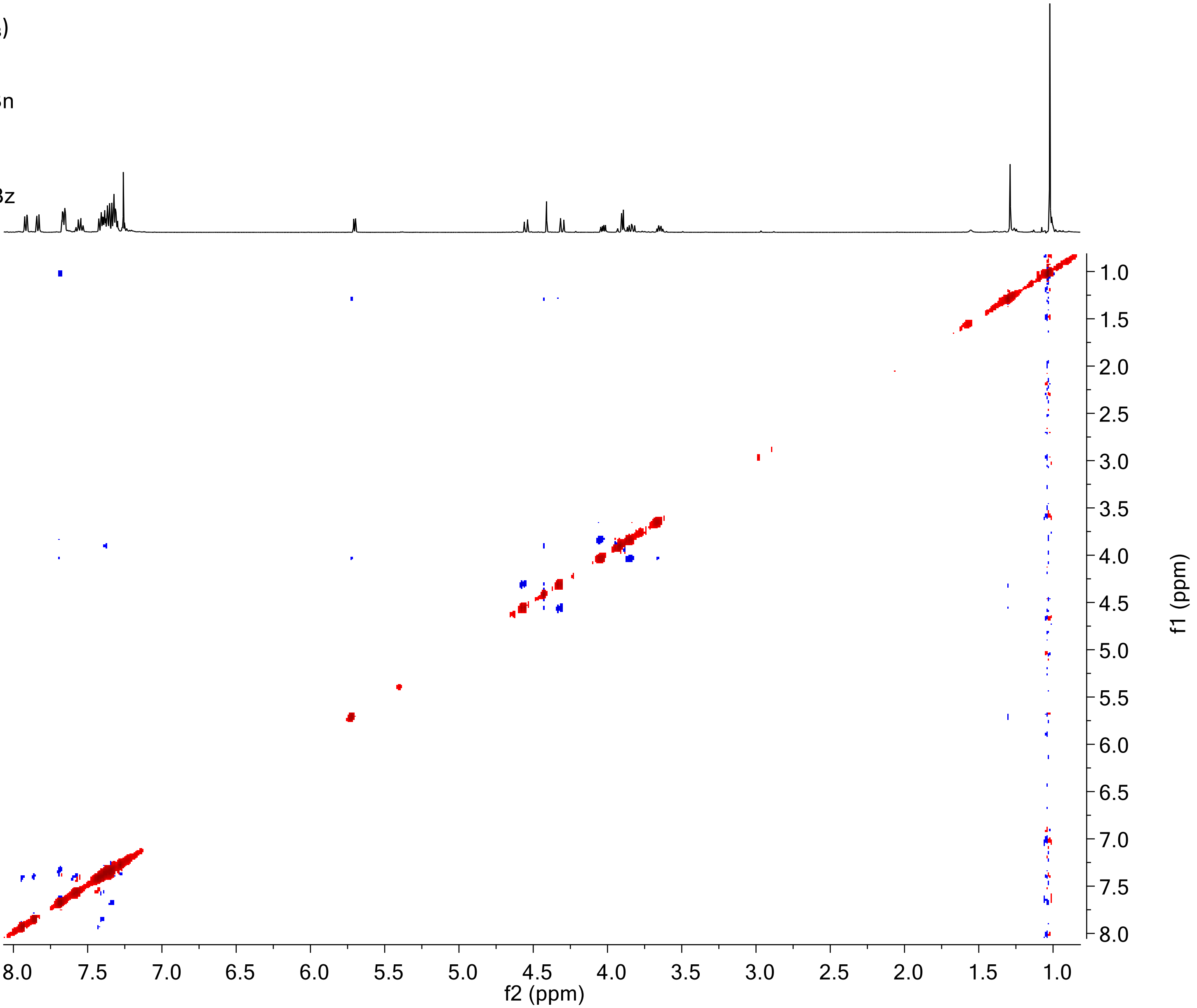

^{1}H-NMR (500 MHz, $CDCl_3$)

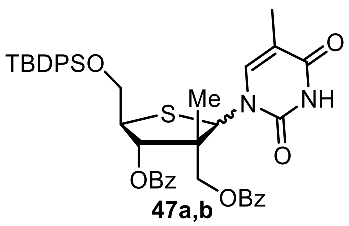

47a,b

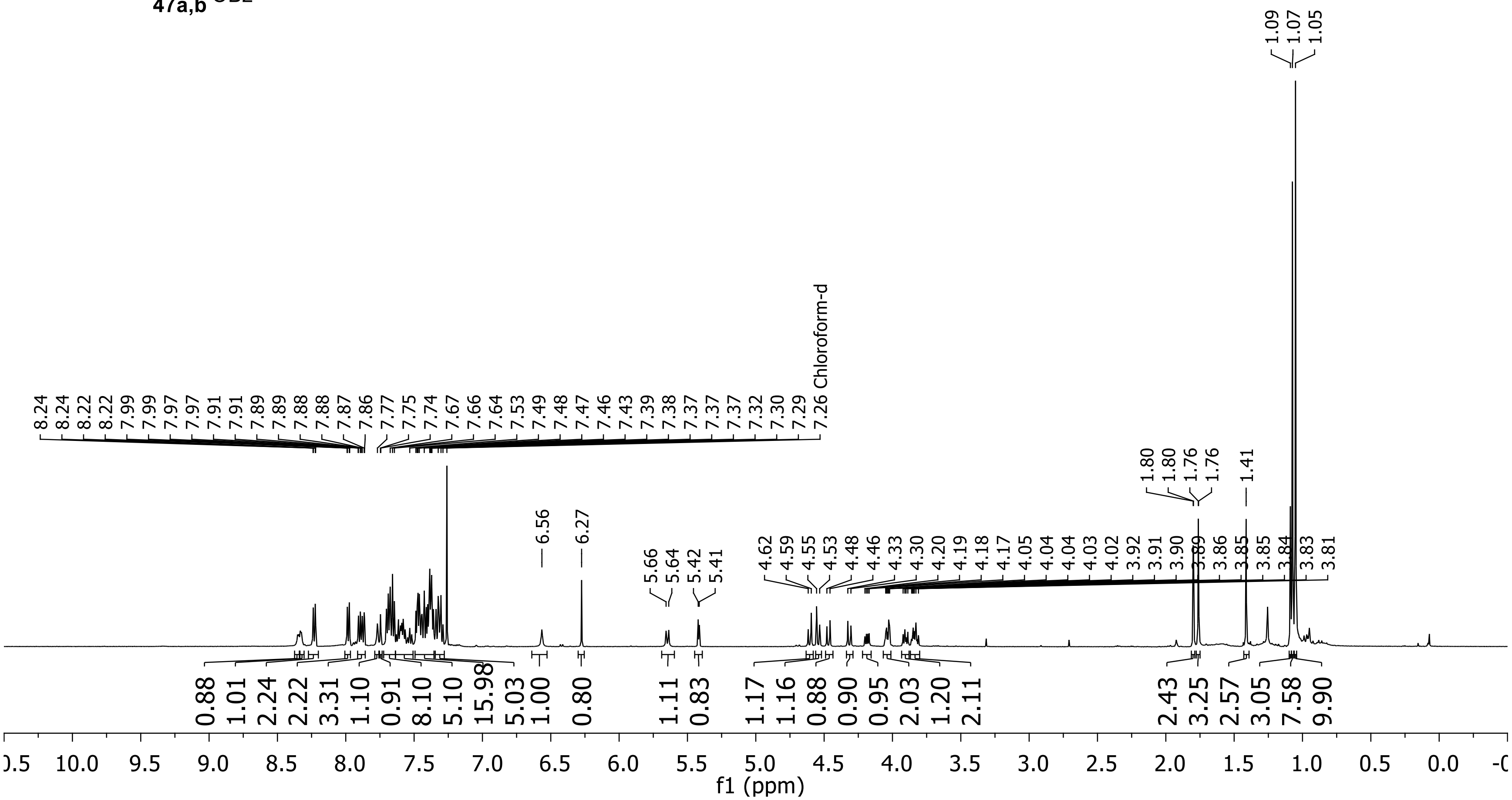

$^{13}C\{^{1}H\}$-NMR (126 MHz, $CDCl_3$)

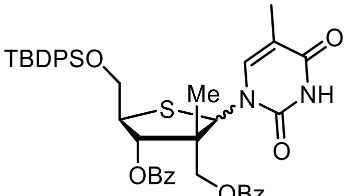

47a,b

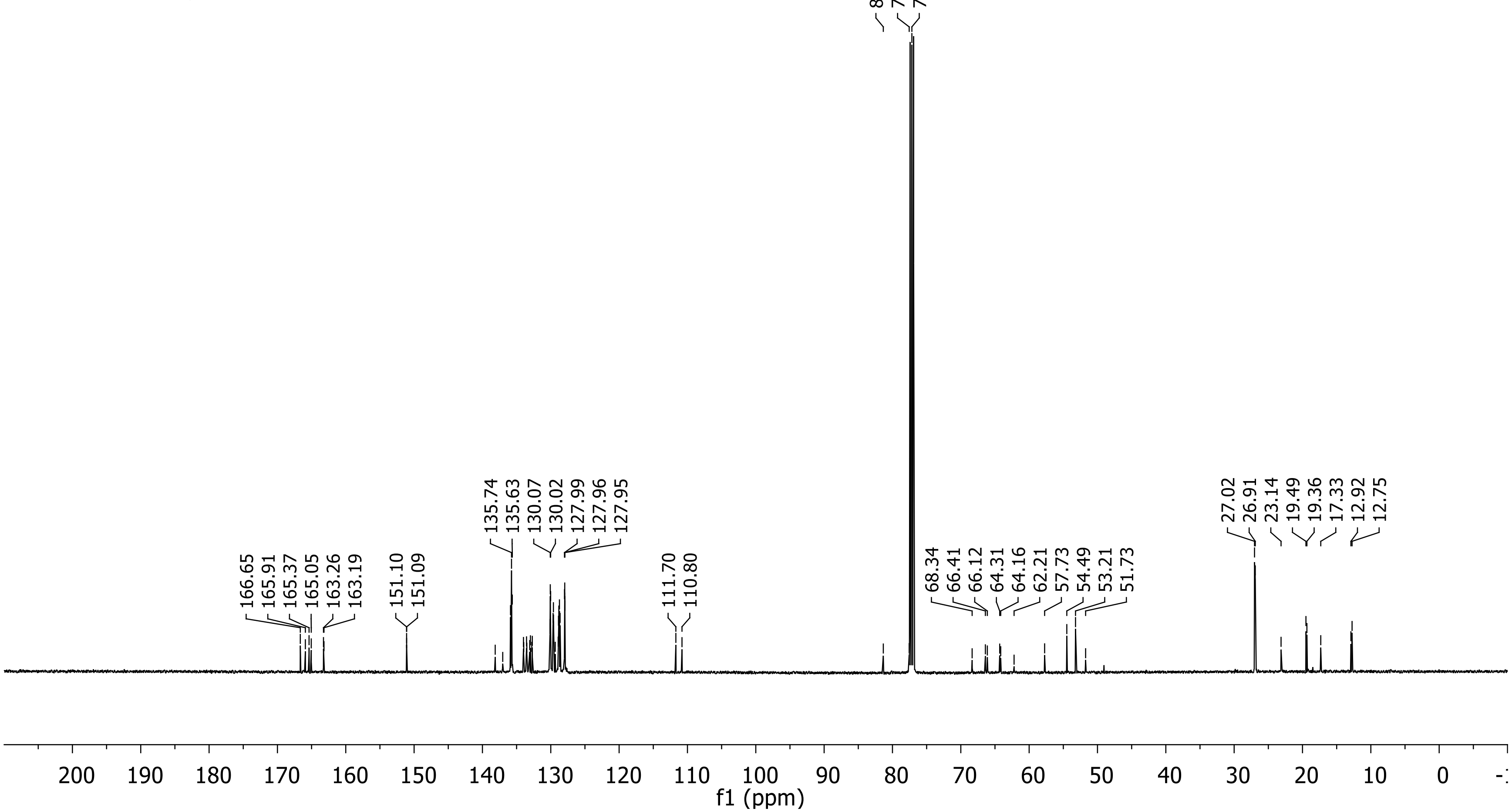

COSY (500 MHz, $CDCl_3$)

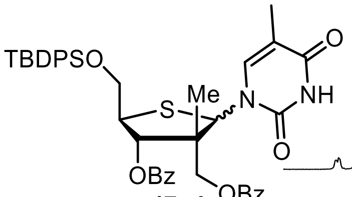

47a,b

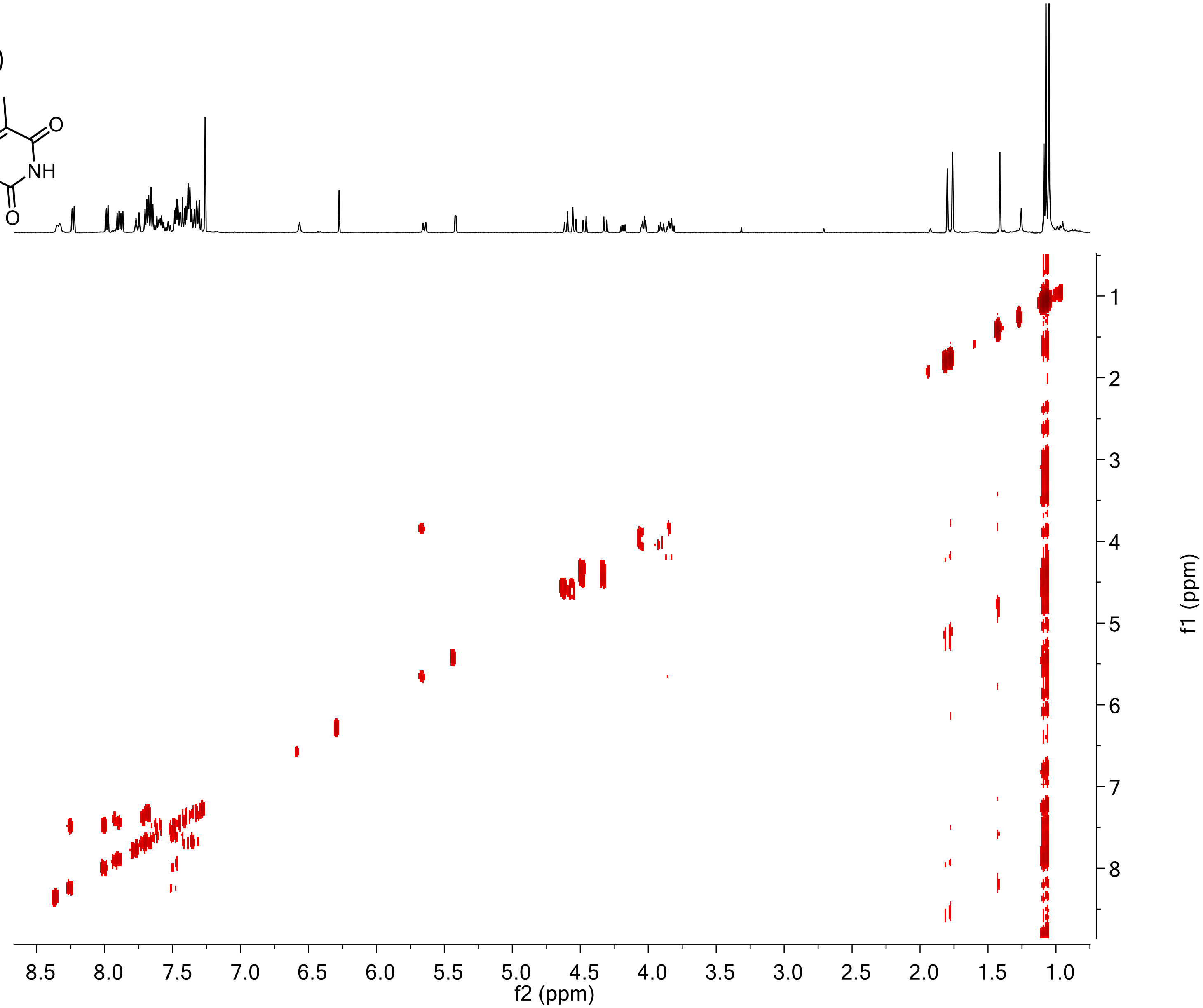

HSQC (500 MHz, $CDCl_3$)

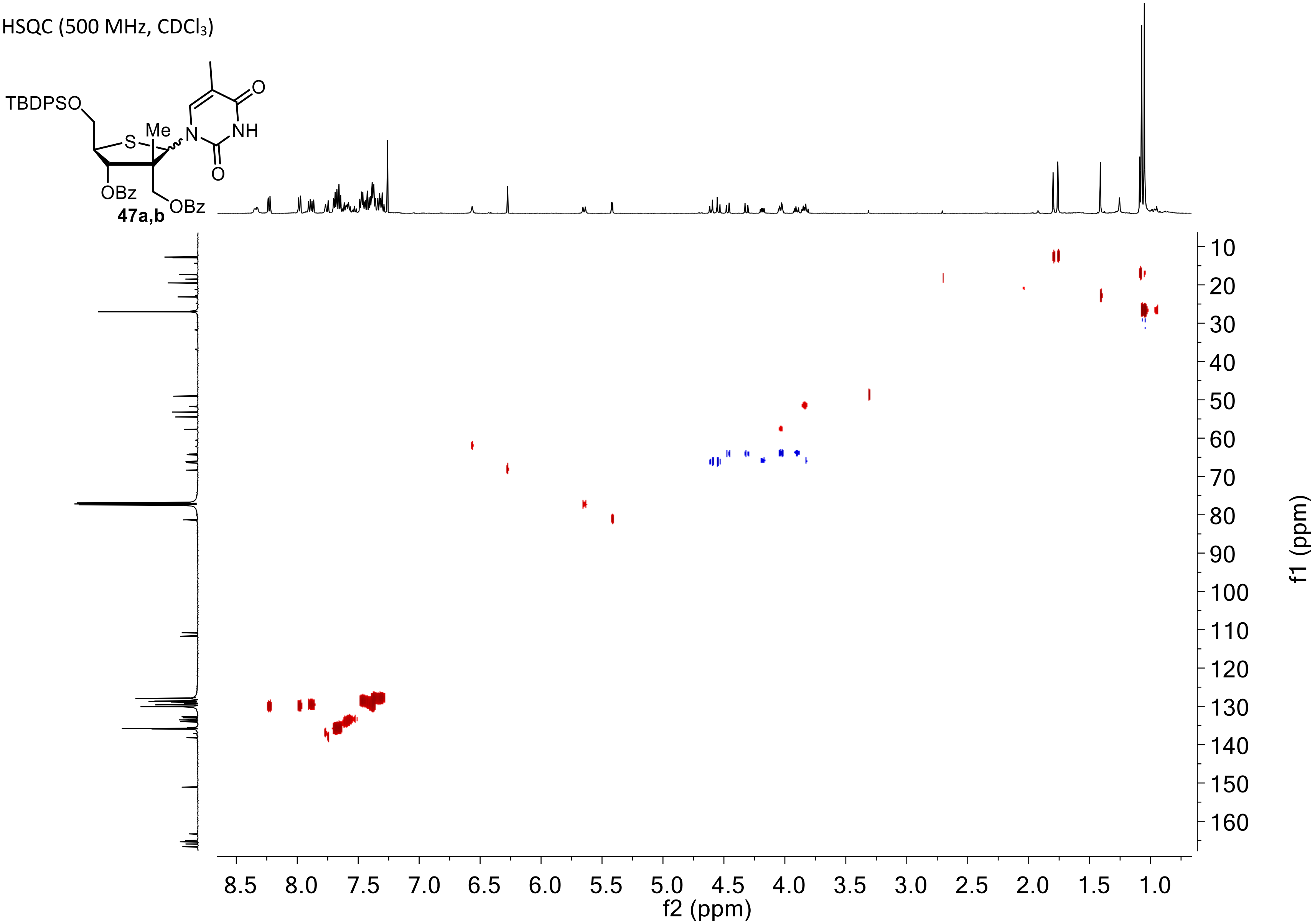

47a,b

HMBC (500 MHz, $CDCl_3$)

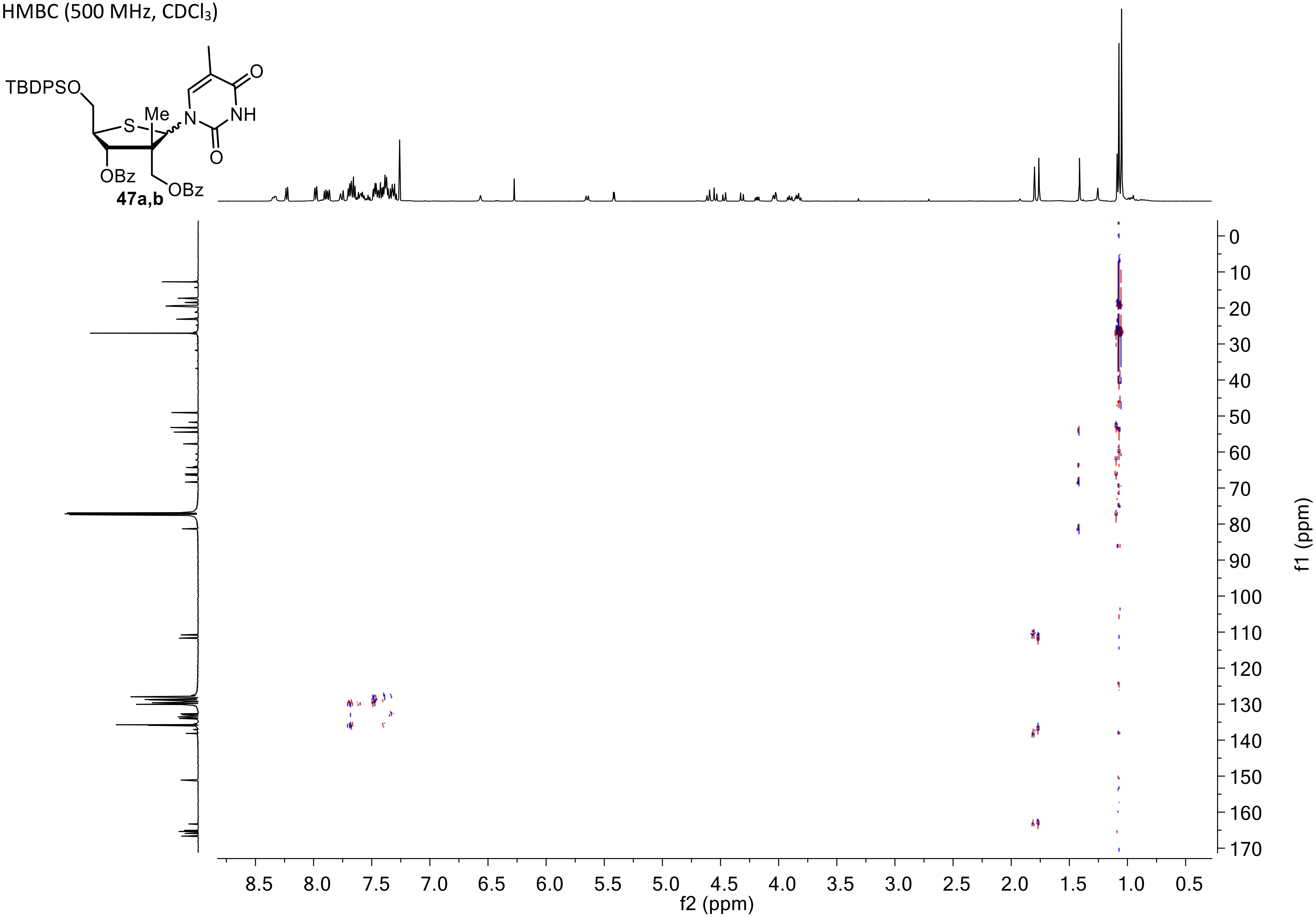

NOESY (500 MHz, $CDCl_3$)

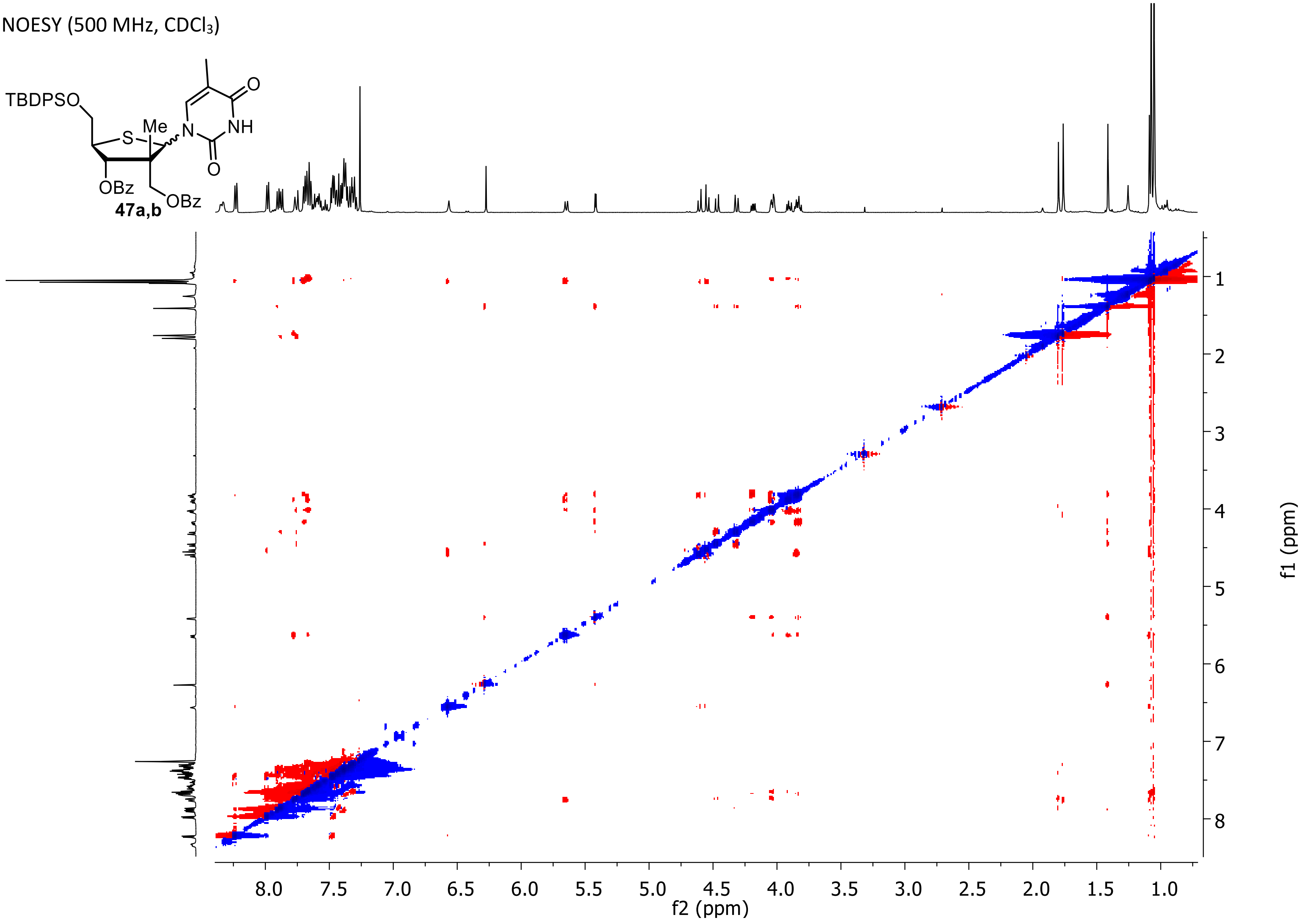

^{1}H-NMR (500 MHz, $CDCl_3$)

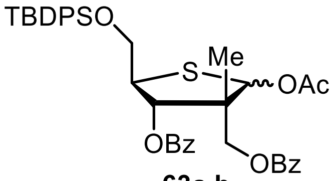

63a,b

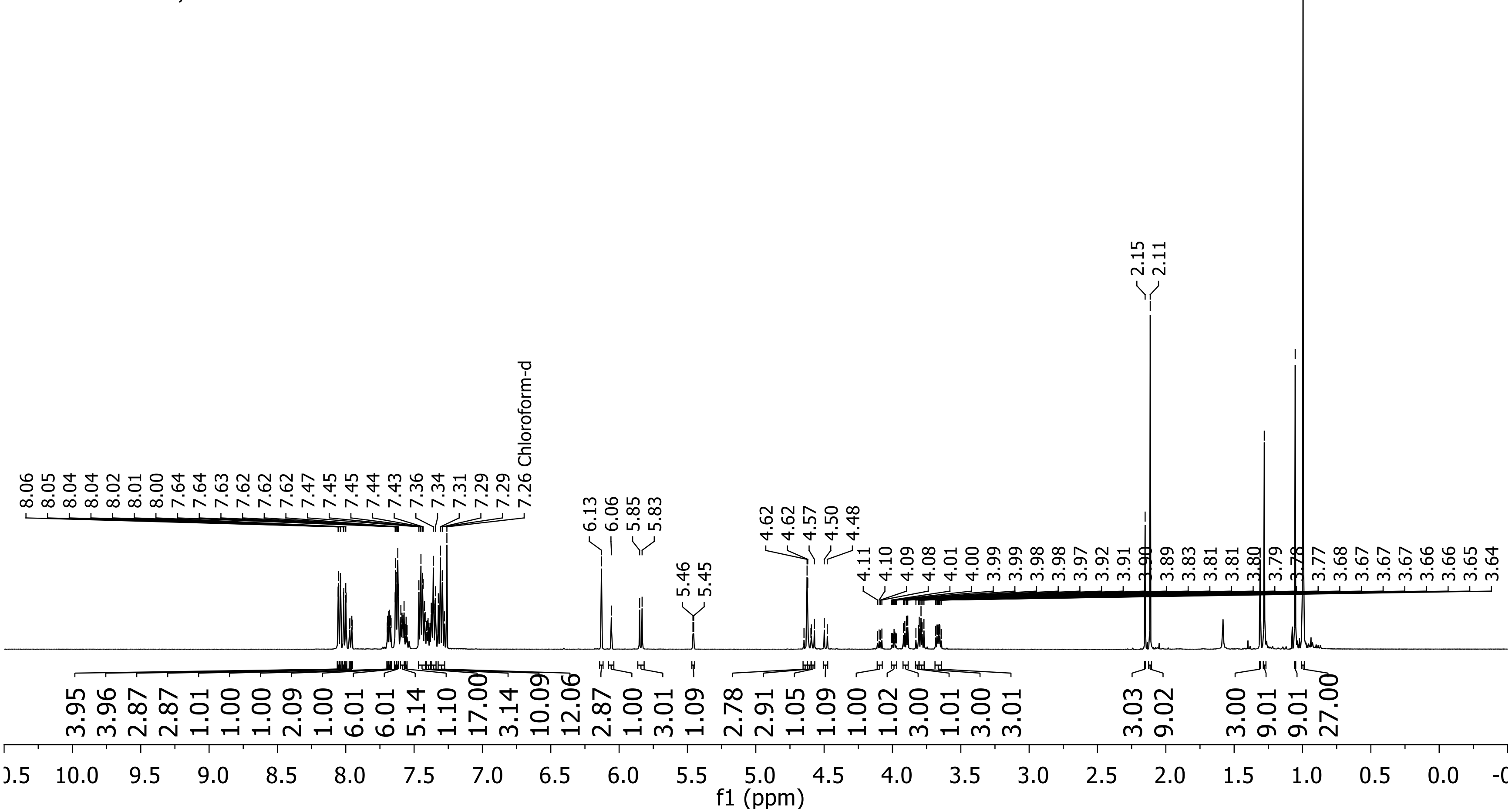

$^{13}C\{^1H\}$-NMR (126 MHz, $CDCl_3$)

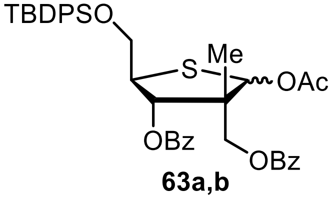

63a,b

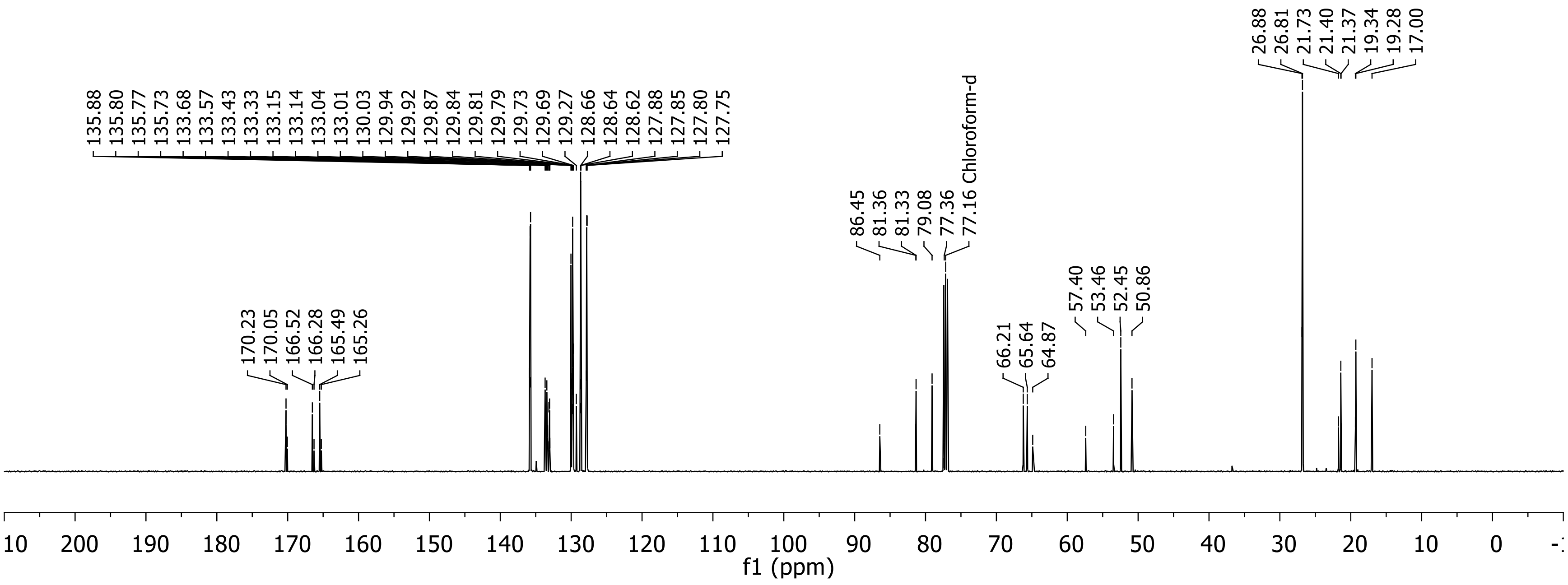

COSY (500 MHz, $CDCl_3$)

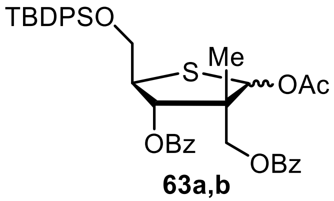

63a,b

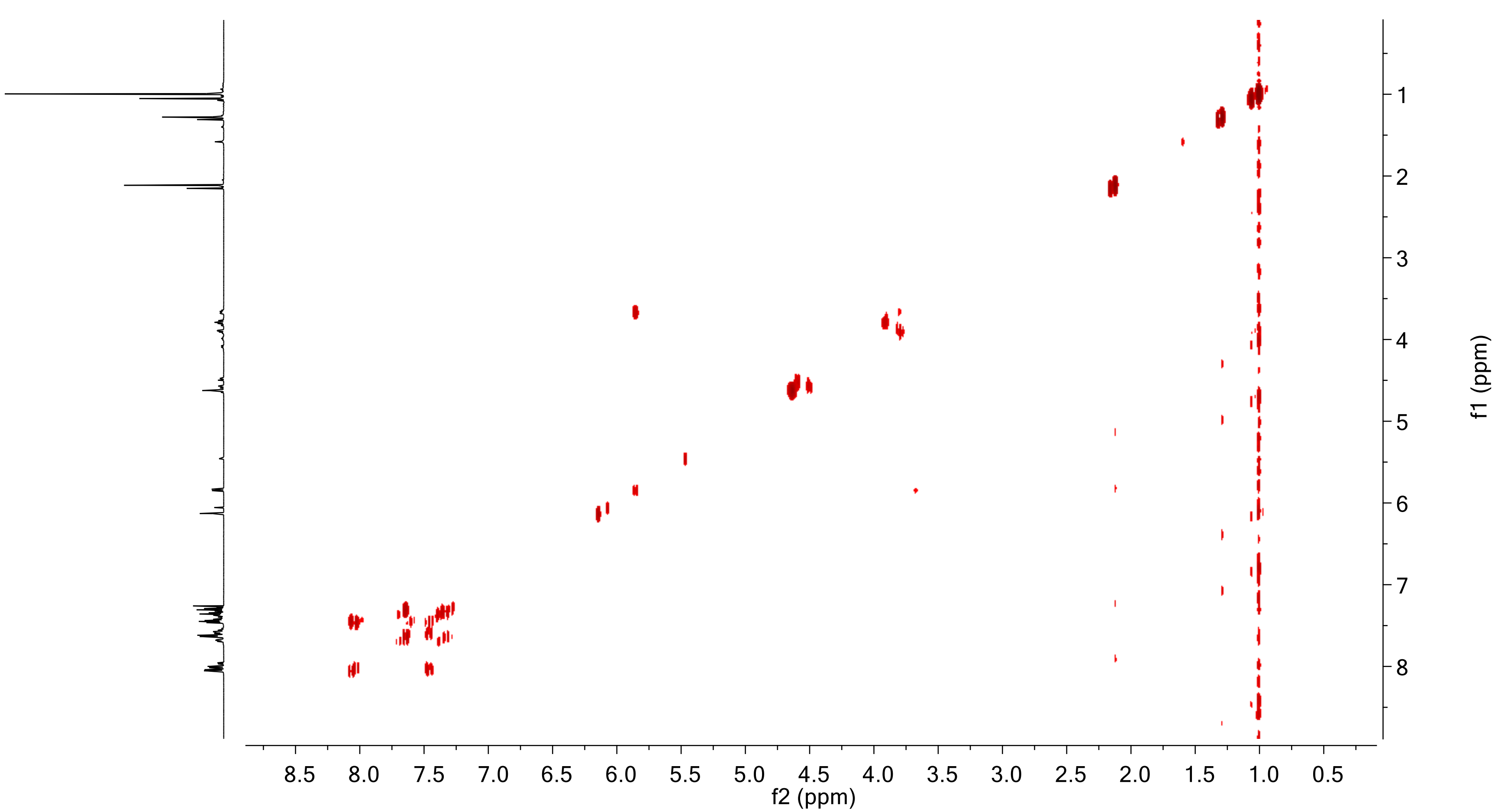

NOESY (500 MHz, $CDCl_3$)

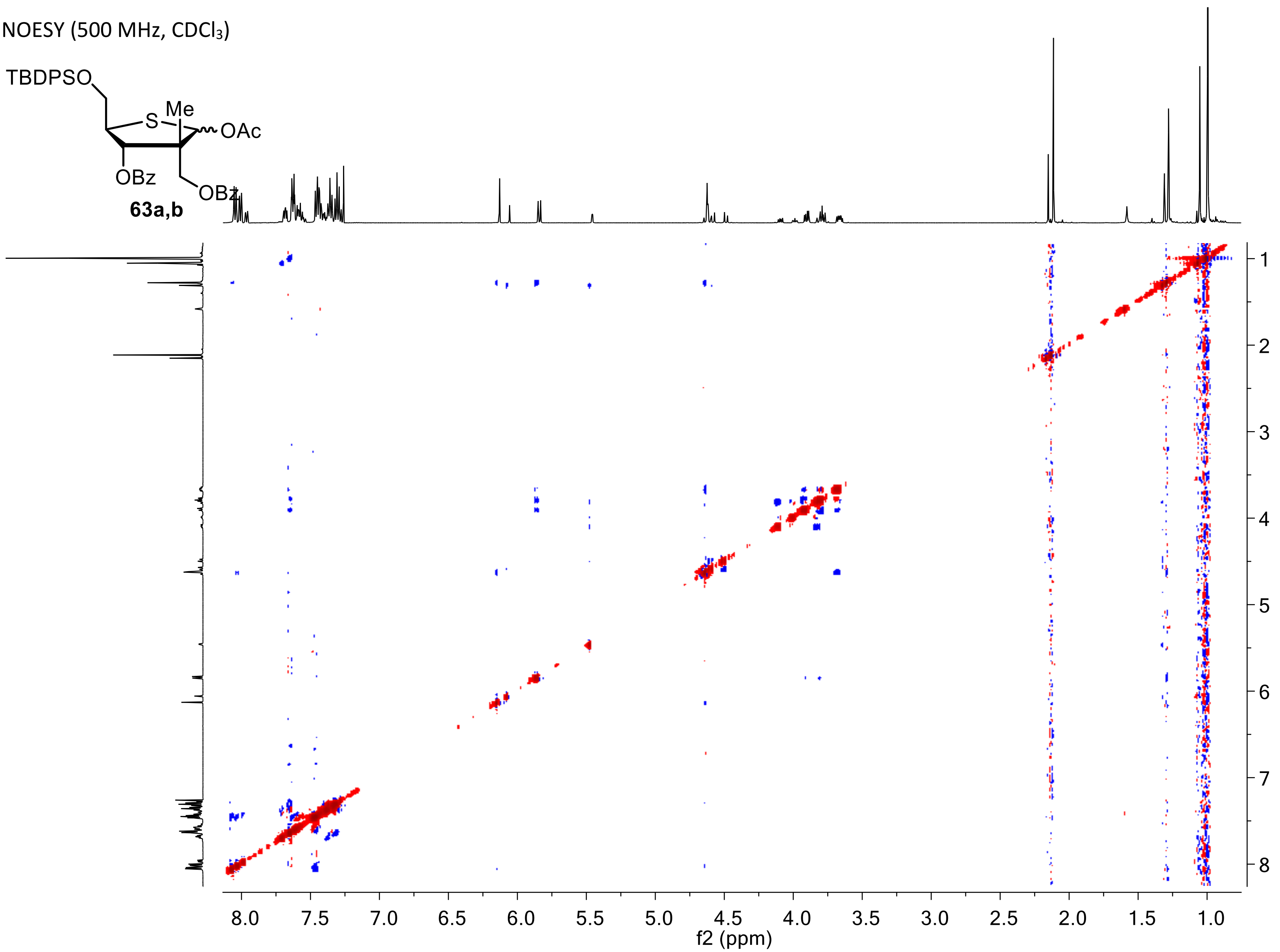

^{1}H-NMR (500 MHz, $CDCl_3$)

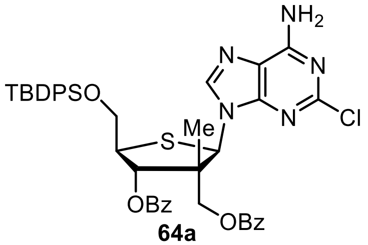

64a

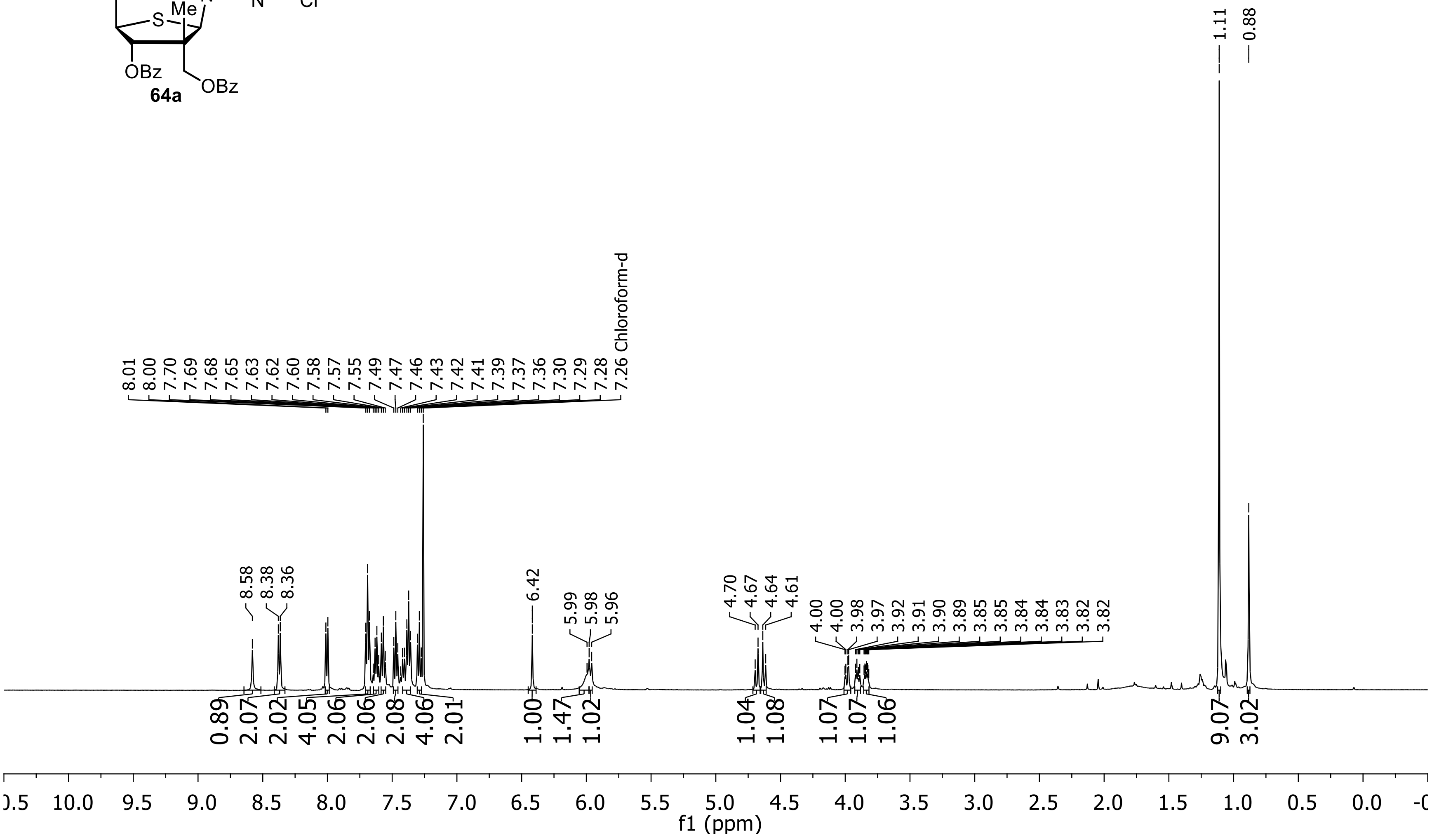

$^{13}C\{^{1}H\}$-NMR (126 MHz, $CDCl_3$)

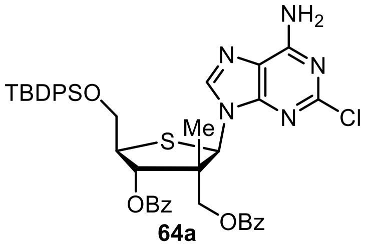

64a

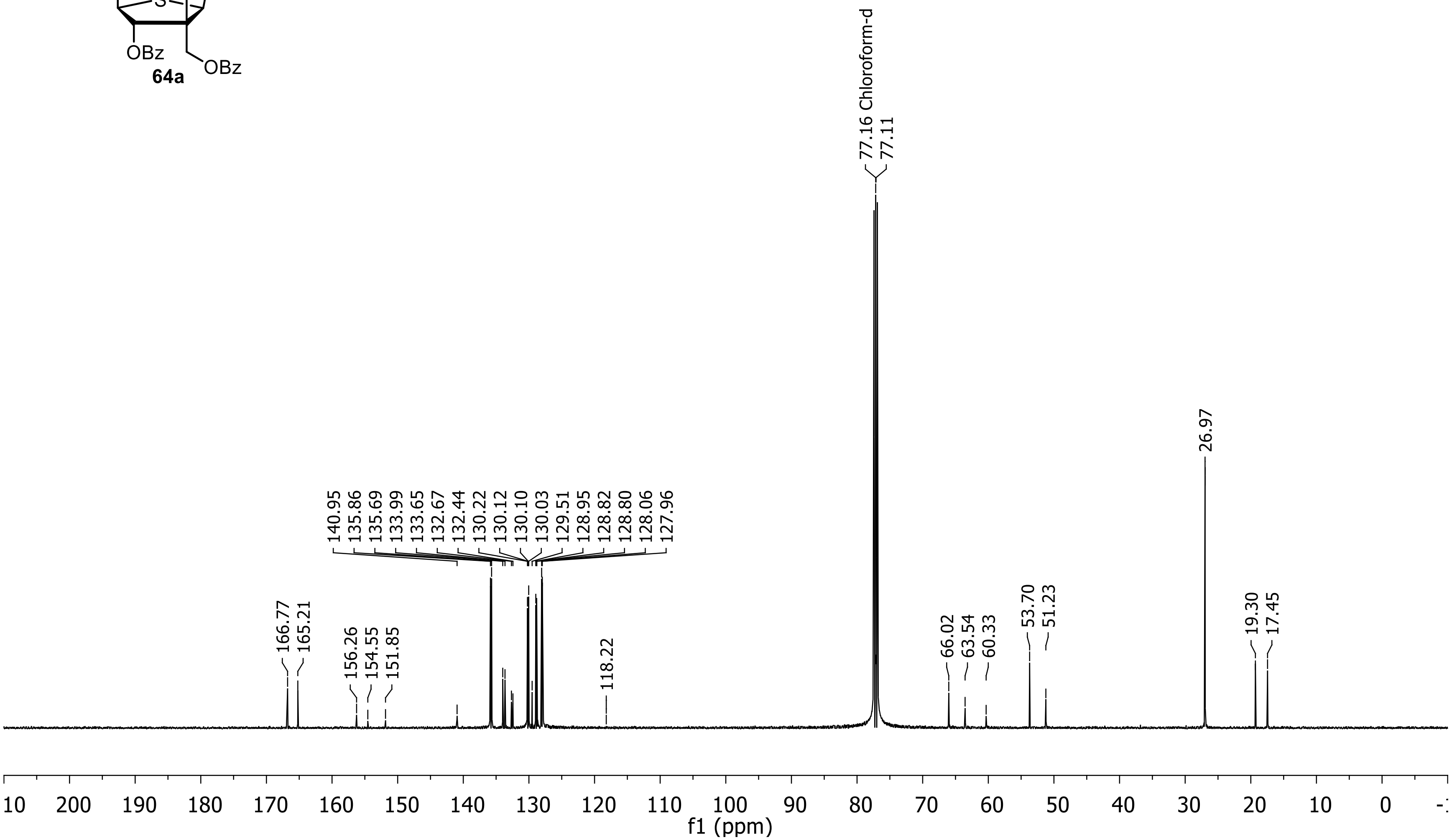

HSQC (500 MHz, $CDCl_3$)

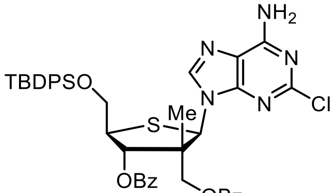

64a

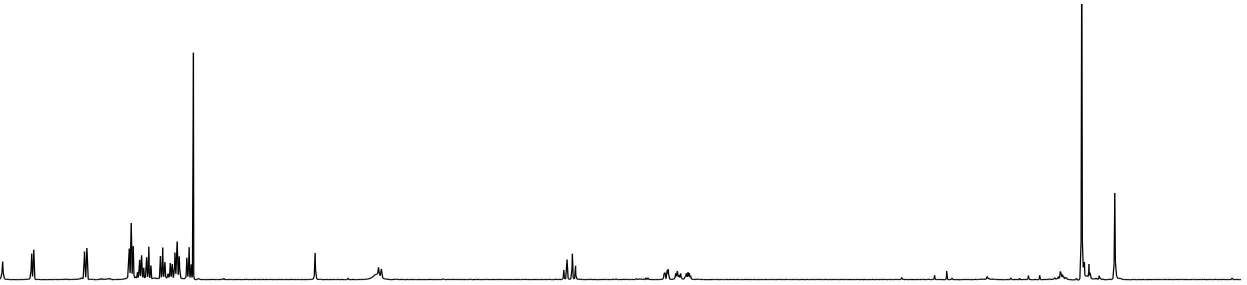

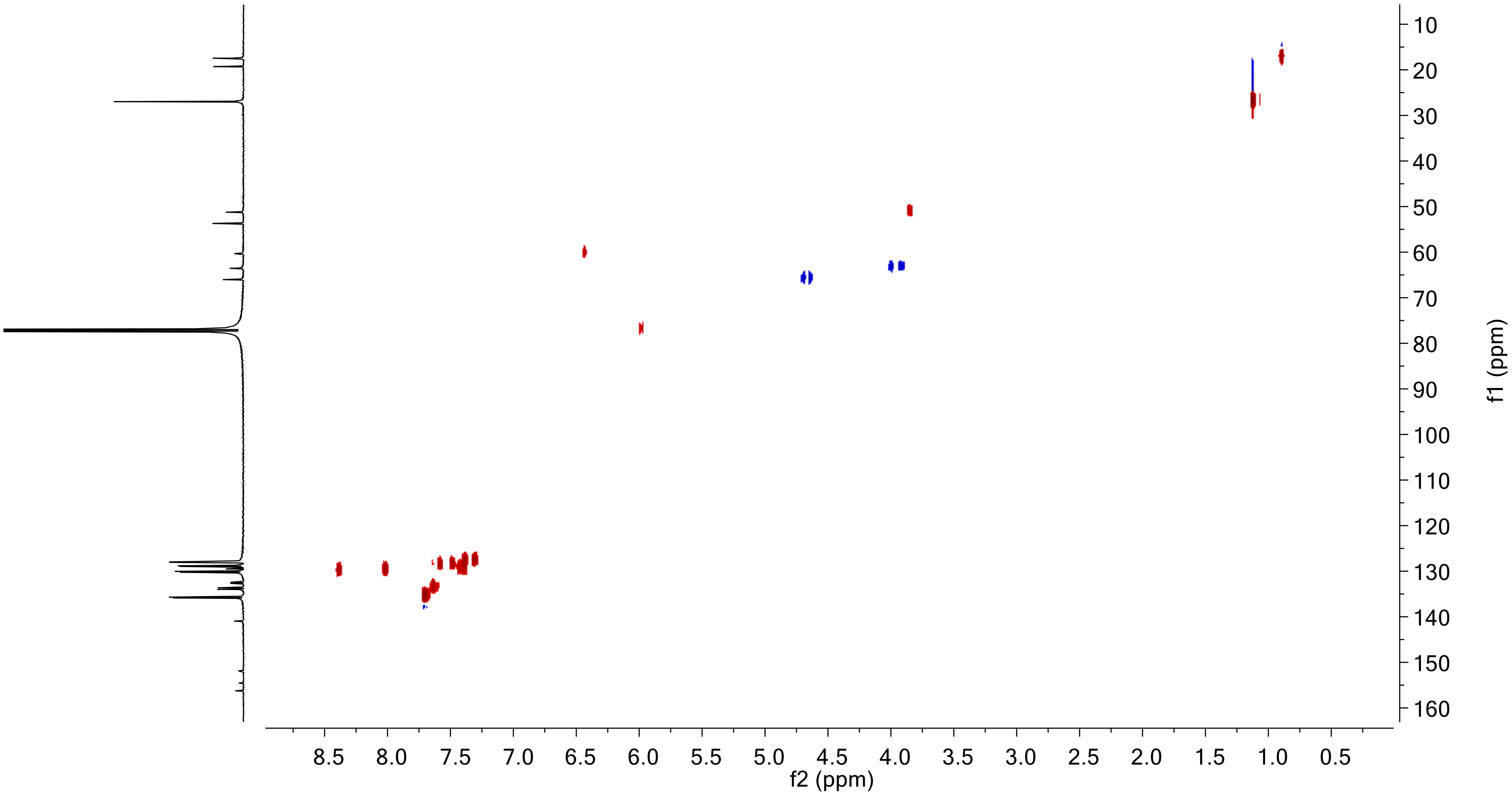

HMBC (500 MHz, $CDCl_3$)

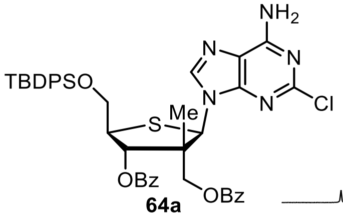

64a

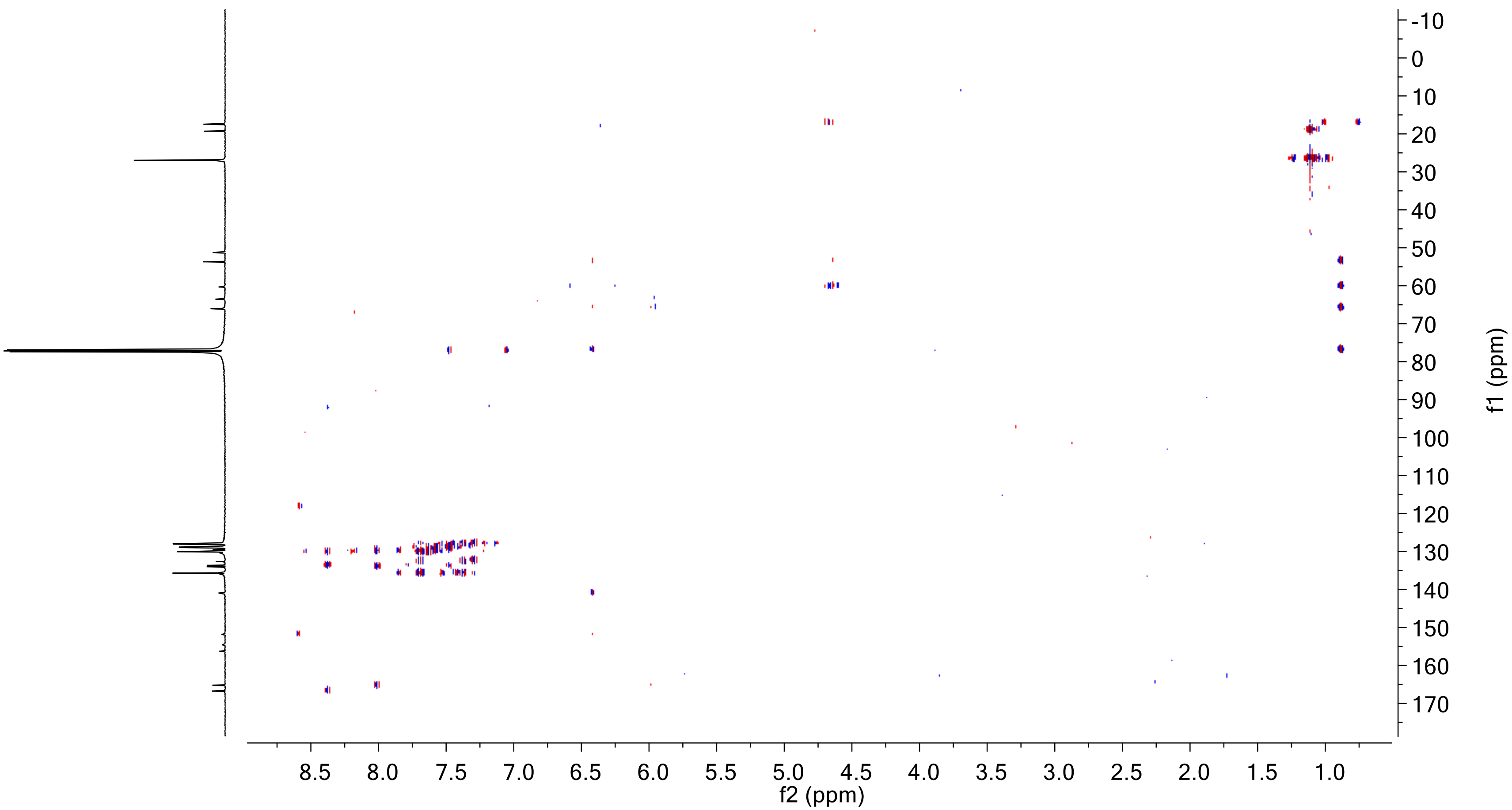

NOESY (500 MHz, $CDCl_3$)

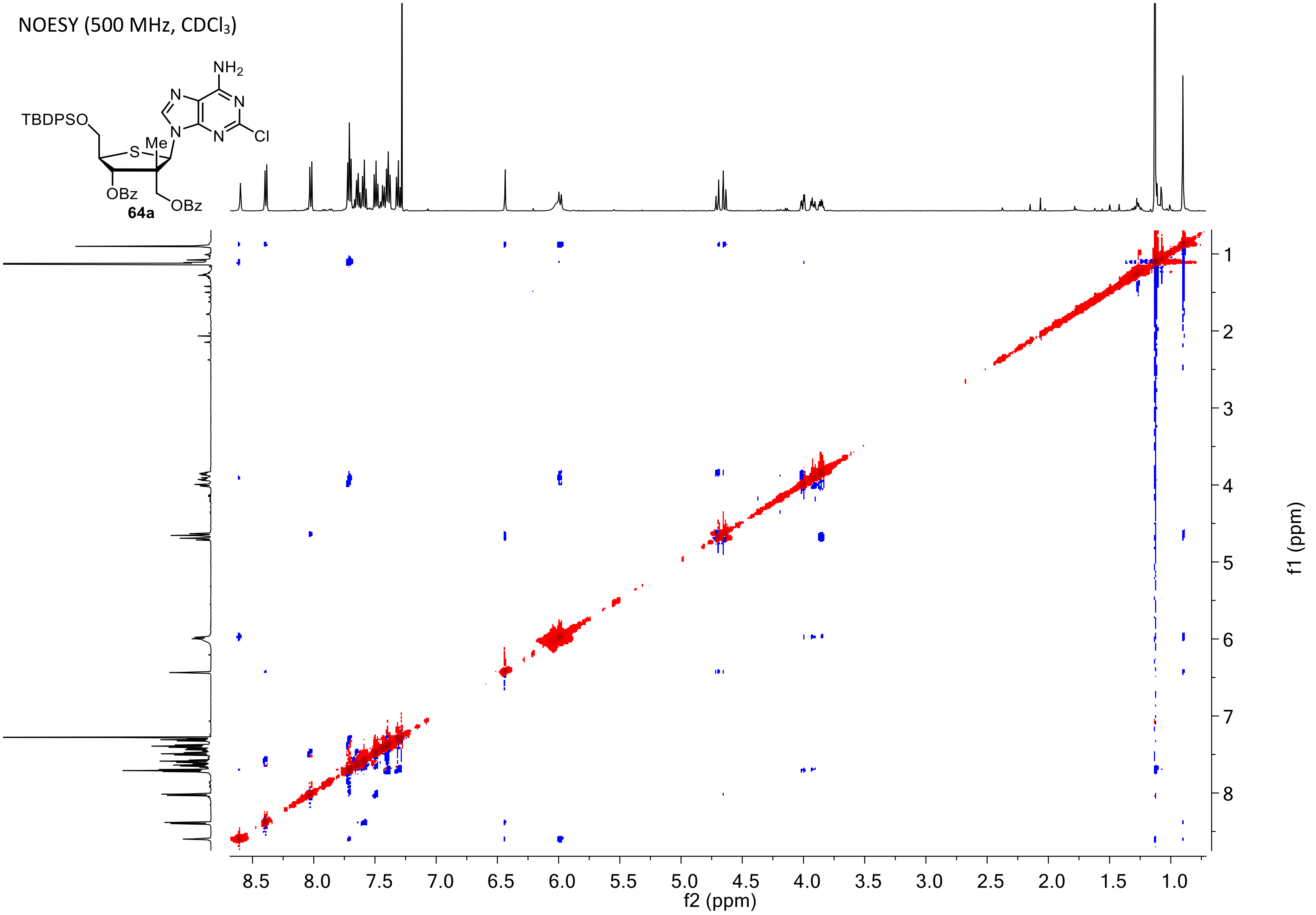

^{1}H-NMR (500 MHz, $CDCl_3$)

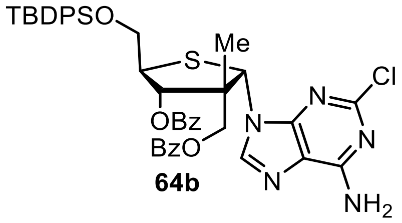

64b

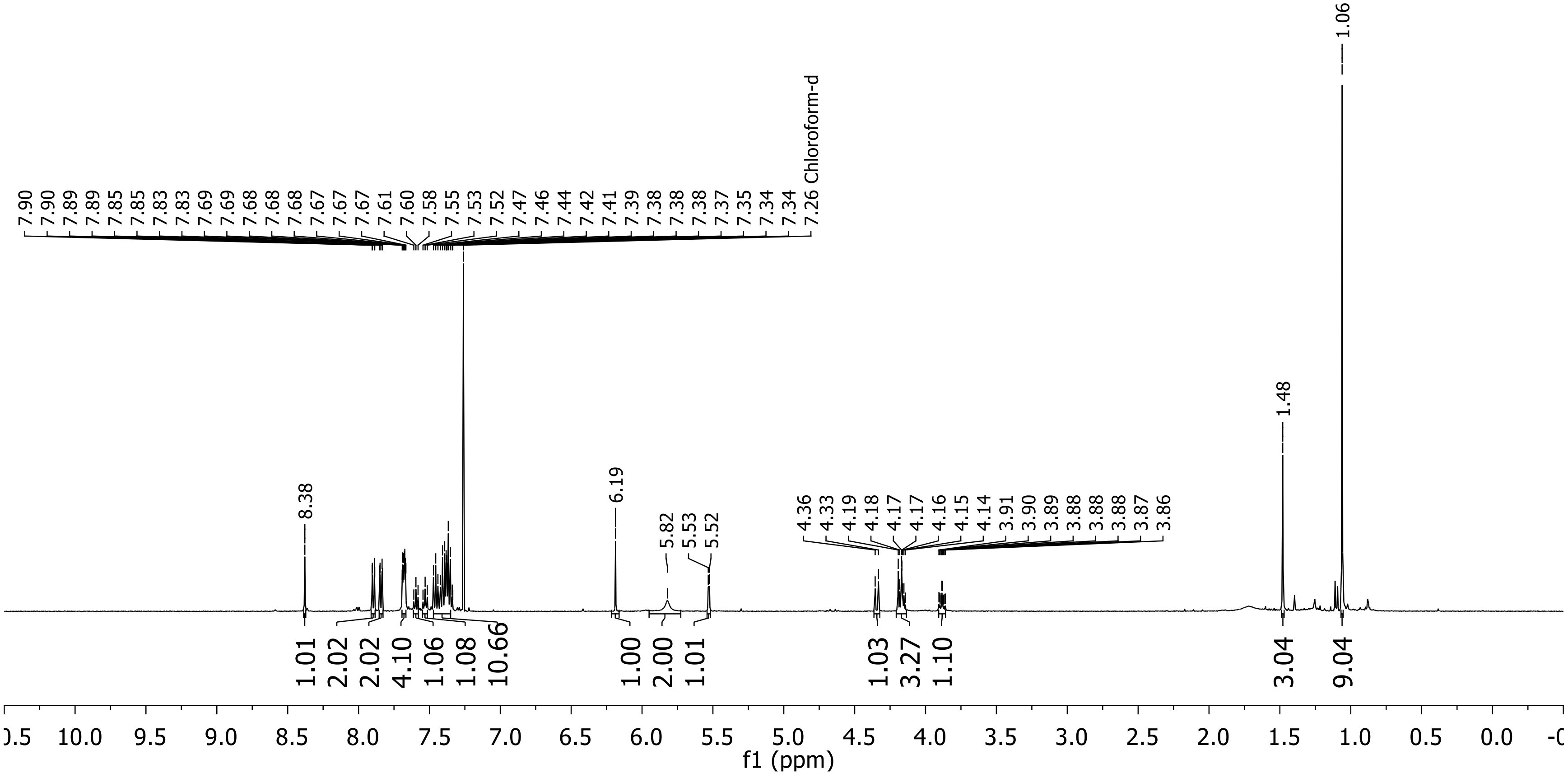

$^{13}C\{^{1}H\}$-NMR (126 MHz, $CDCl_3$)

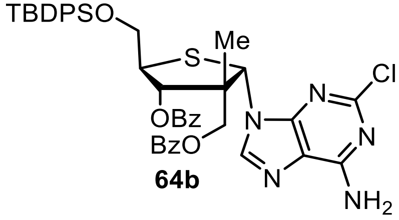

64b

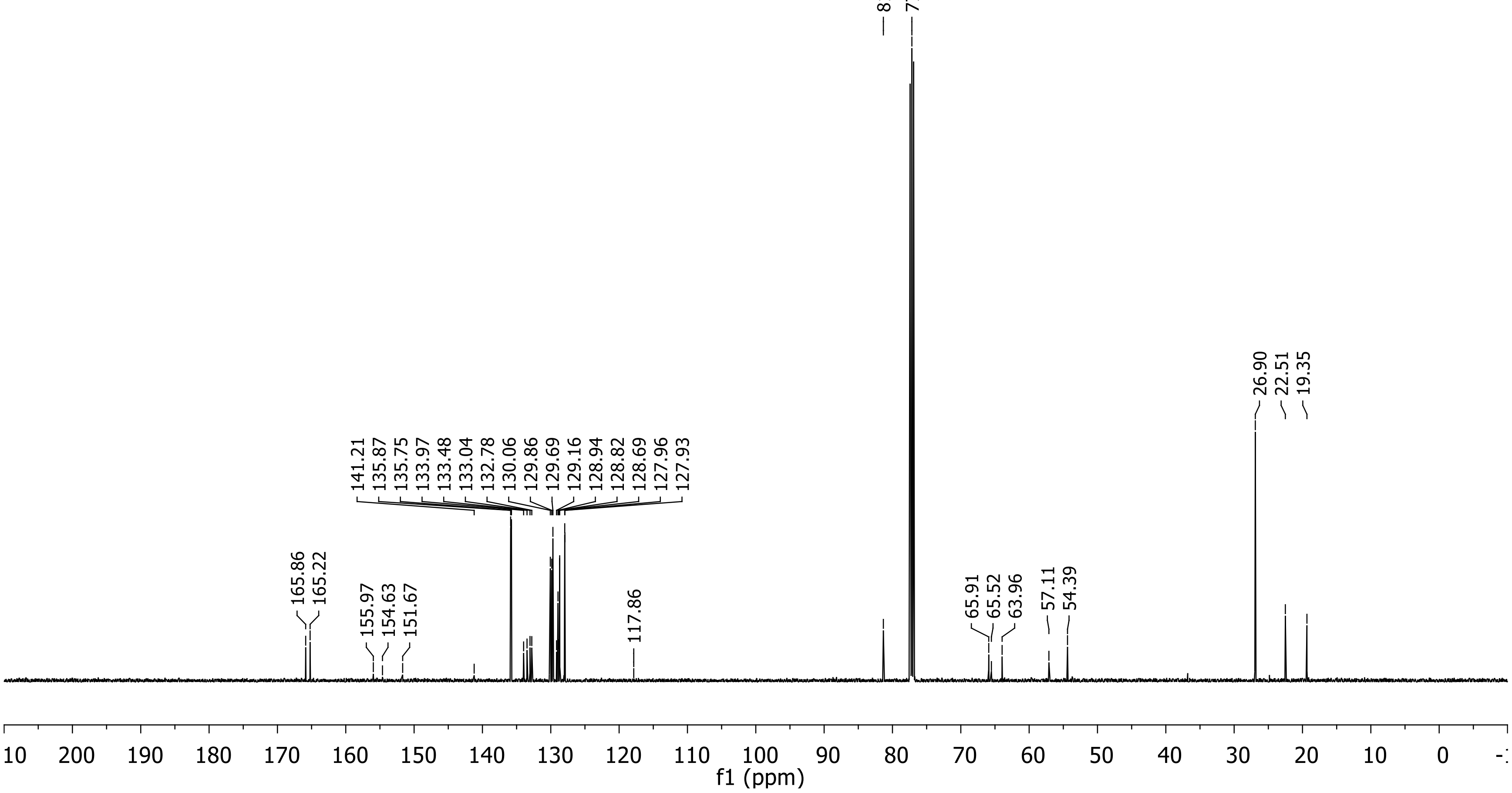

HSQC (500 MHz, $CDCl_3$)

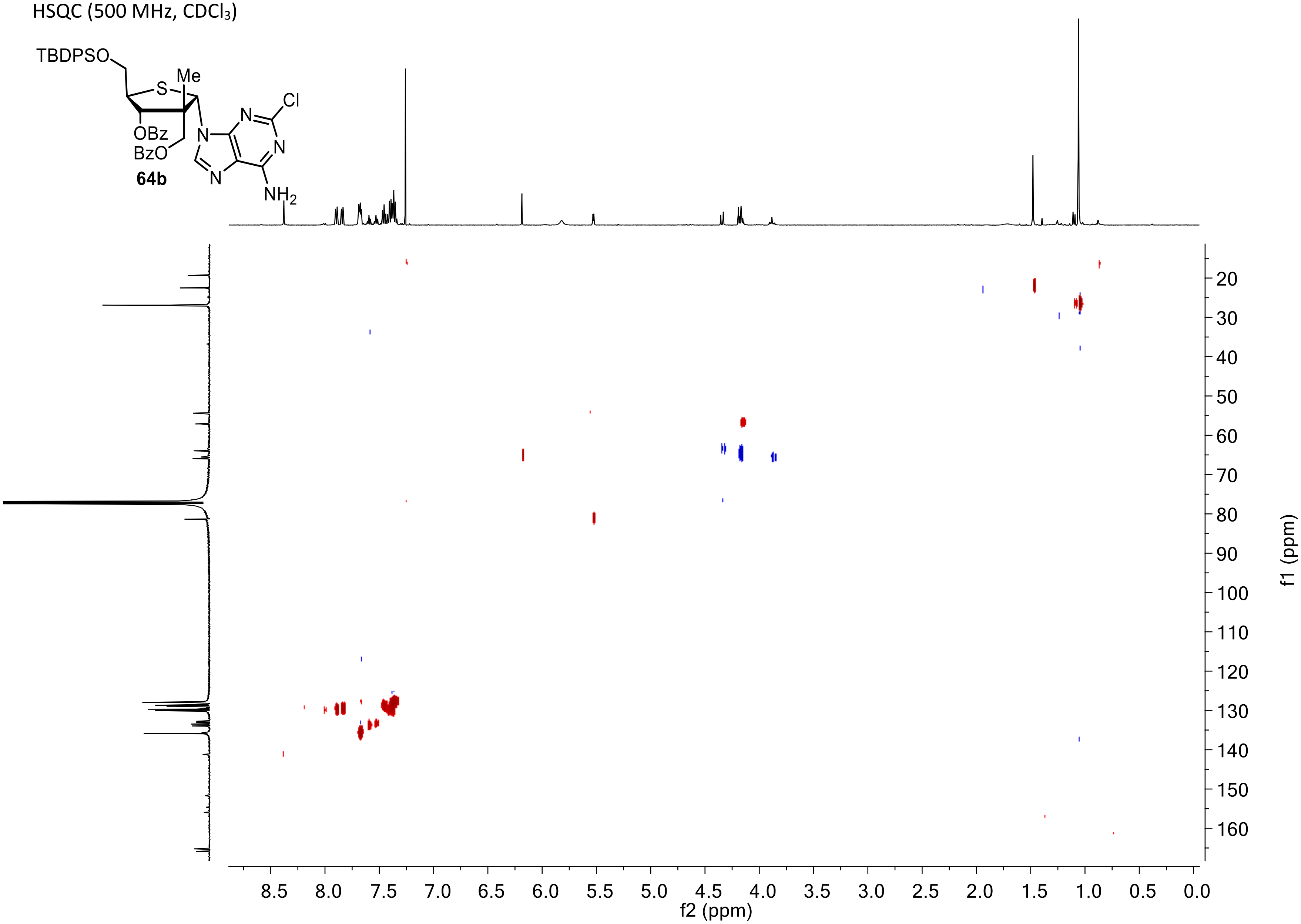

HMBC (500 MHz, $CDCl_3$)

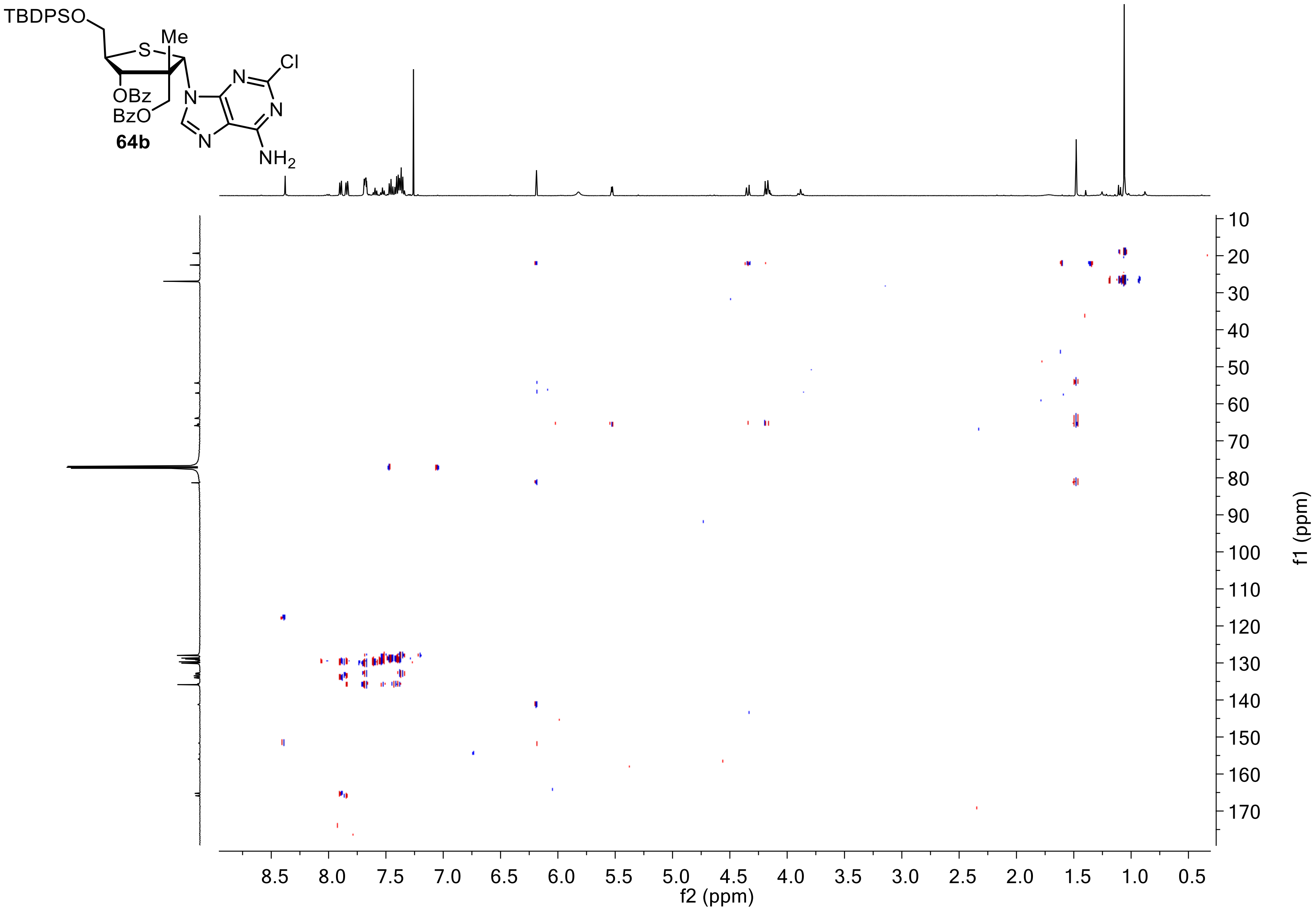

64b

NOESY (500 MHz, $CDCl_3$)

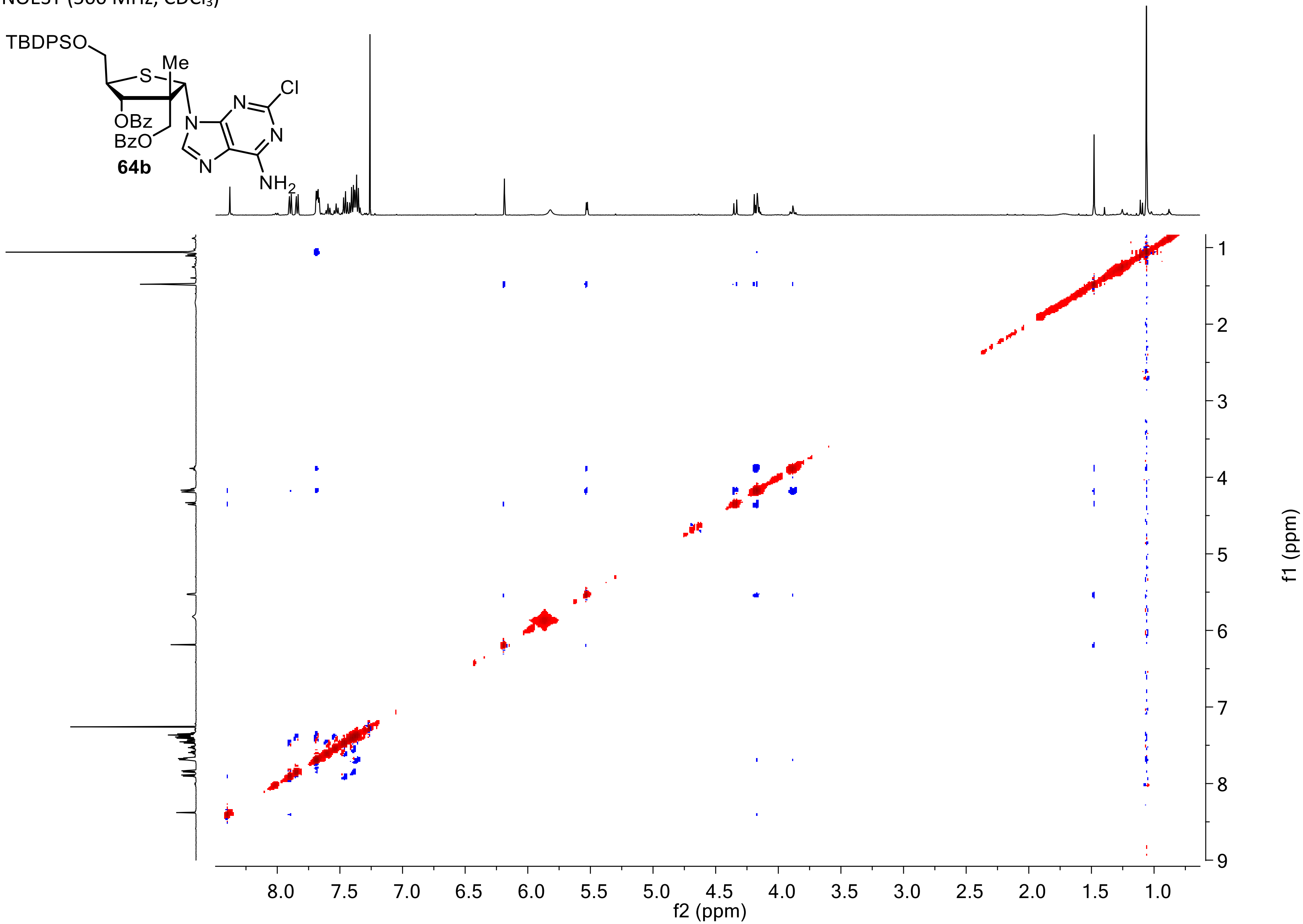

^{1}H-NMR (500 MHz, $CDCl_3$)

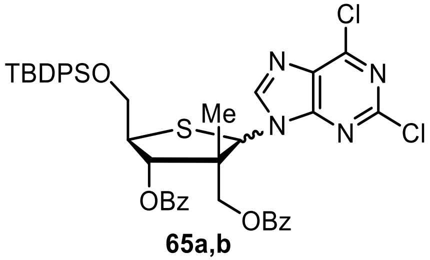

65a,b

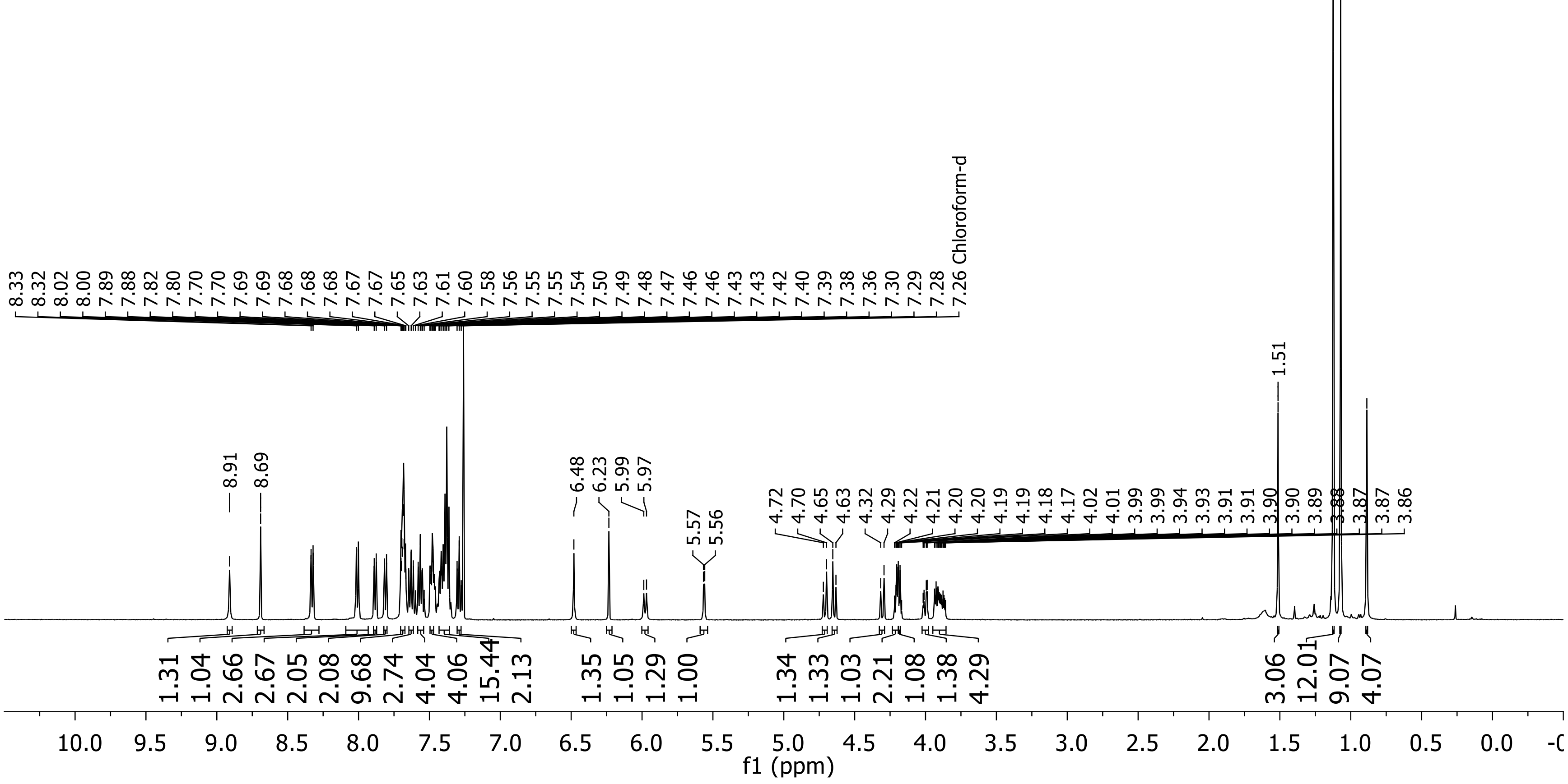

$^{13}C\{^1H\}$-NMR (126 MHz, $CDCl_3$)

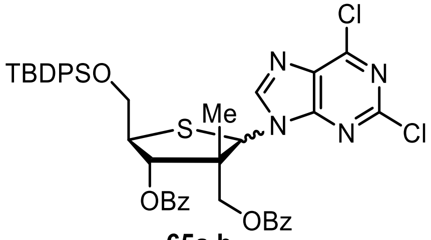

65a,b

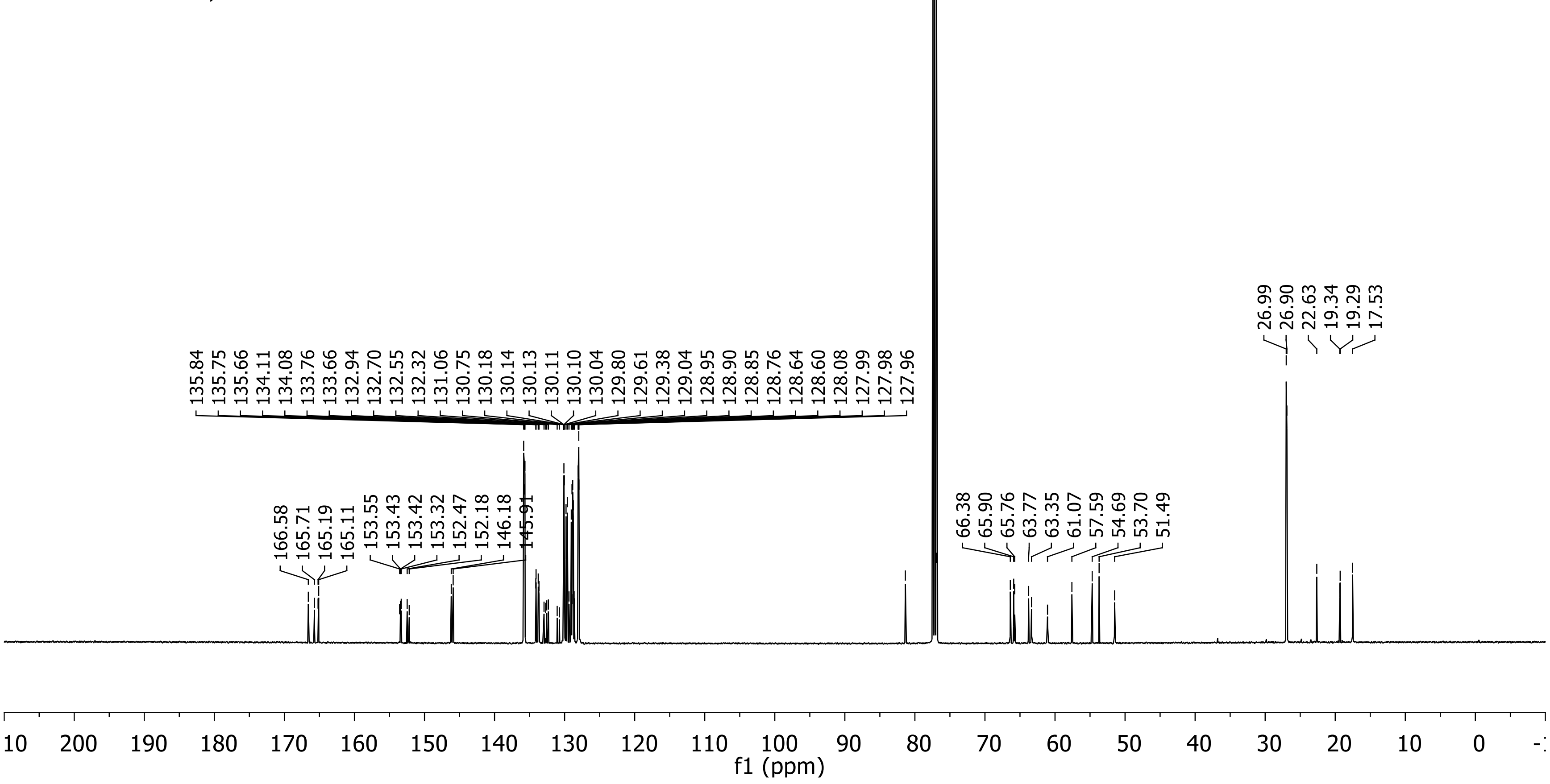

COSY (500 MHz, $CDCl_3$)

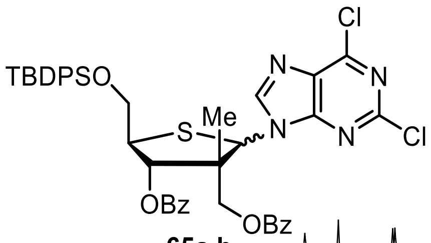

65a,b

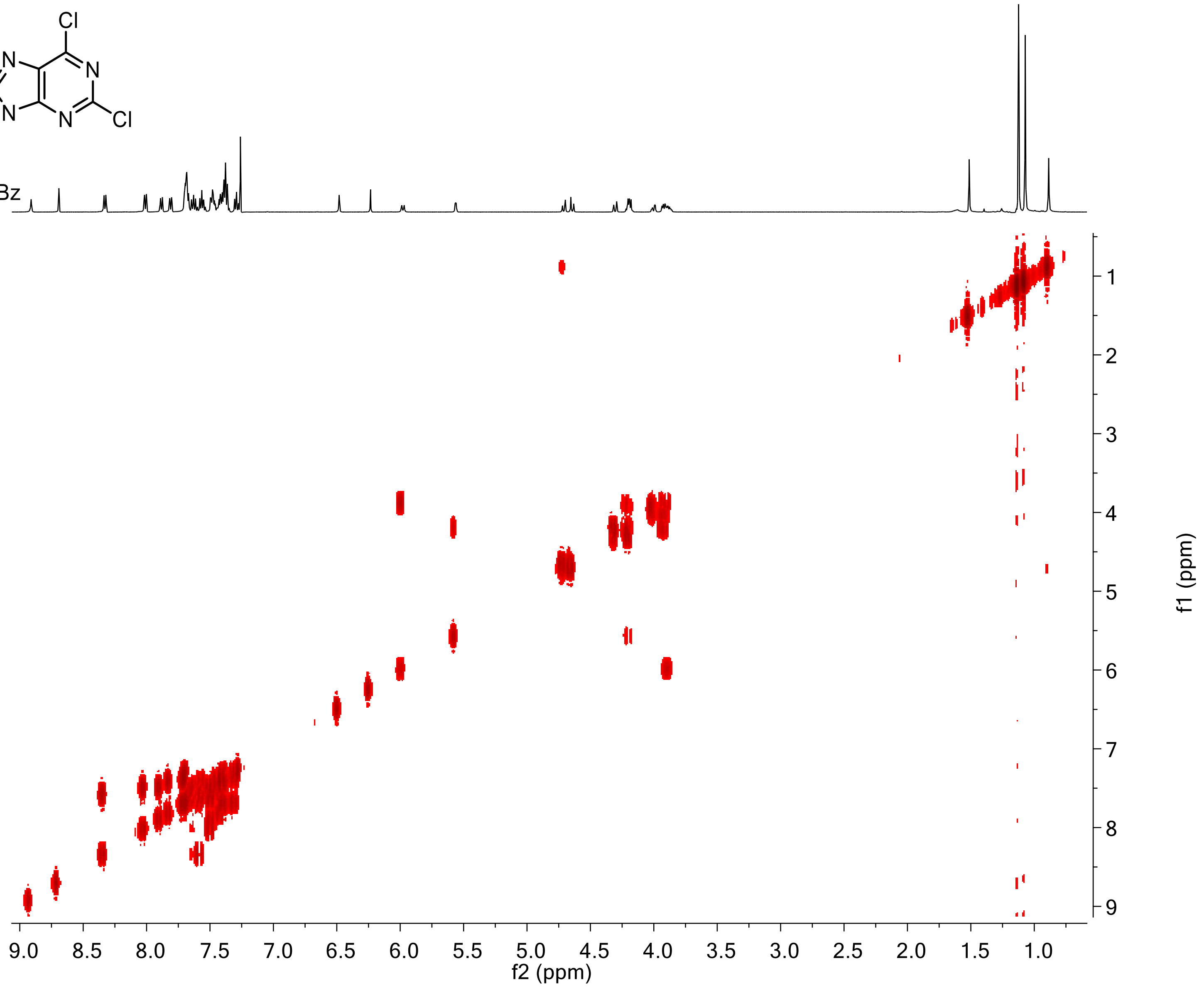

HSQC (500 MHz, $CDCl_3$)

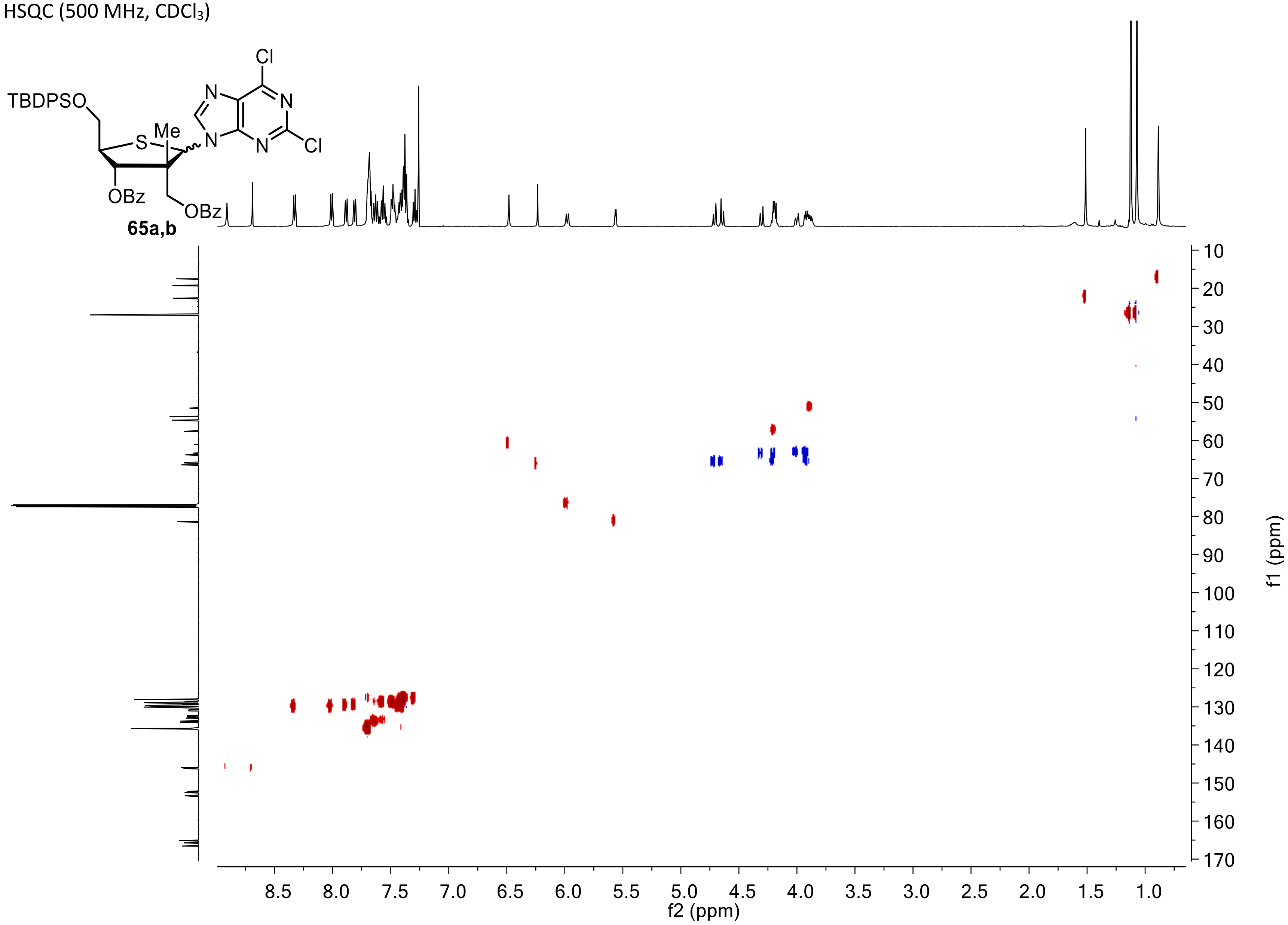

65a,b

HMBC (500 MHz, $CDCl_3$)

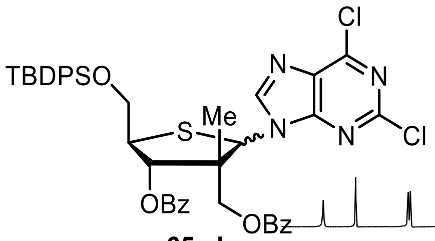

65a,b

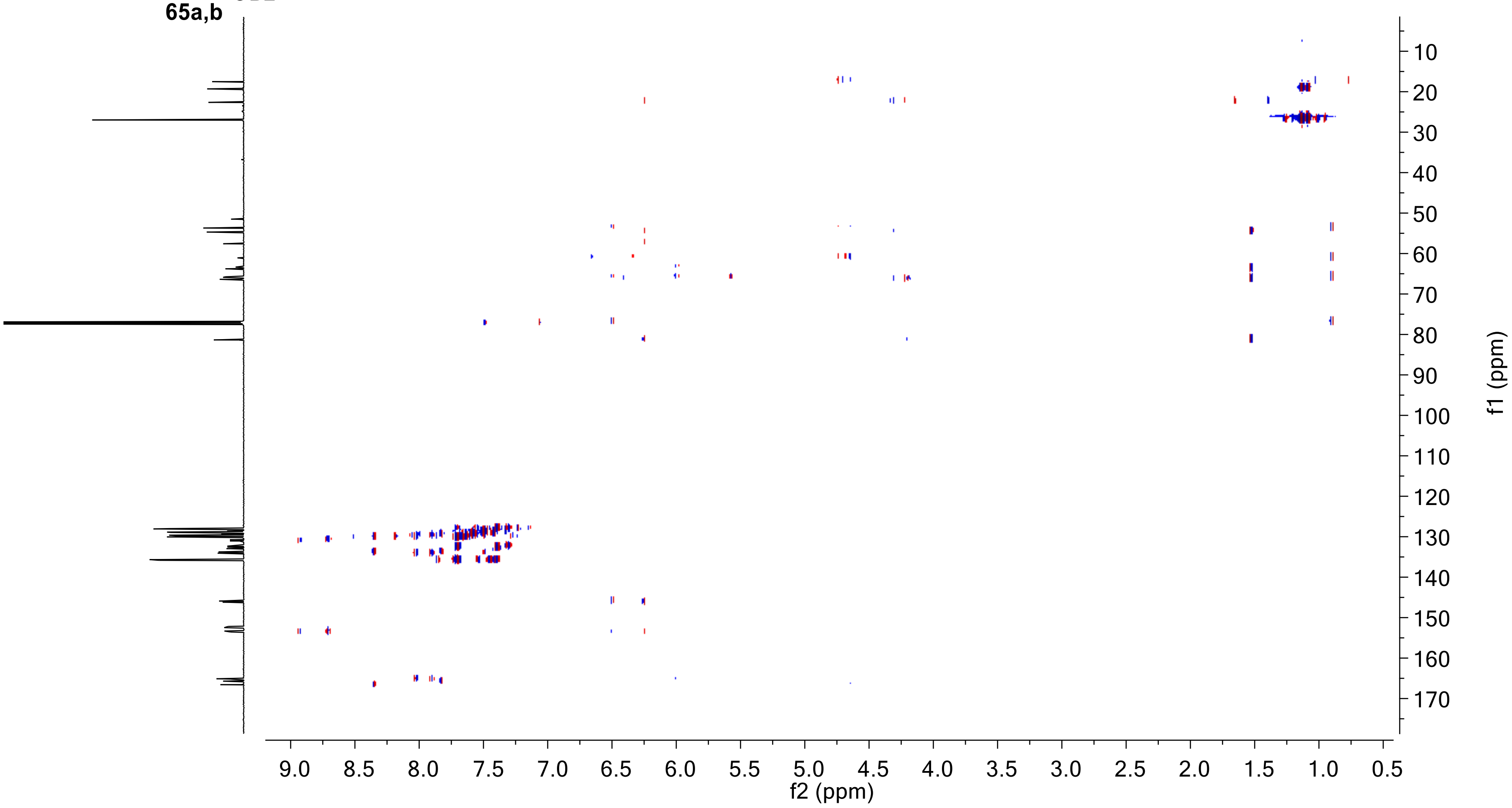

NOESY (500 MHz, CDCl₃)

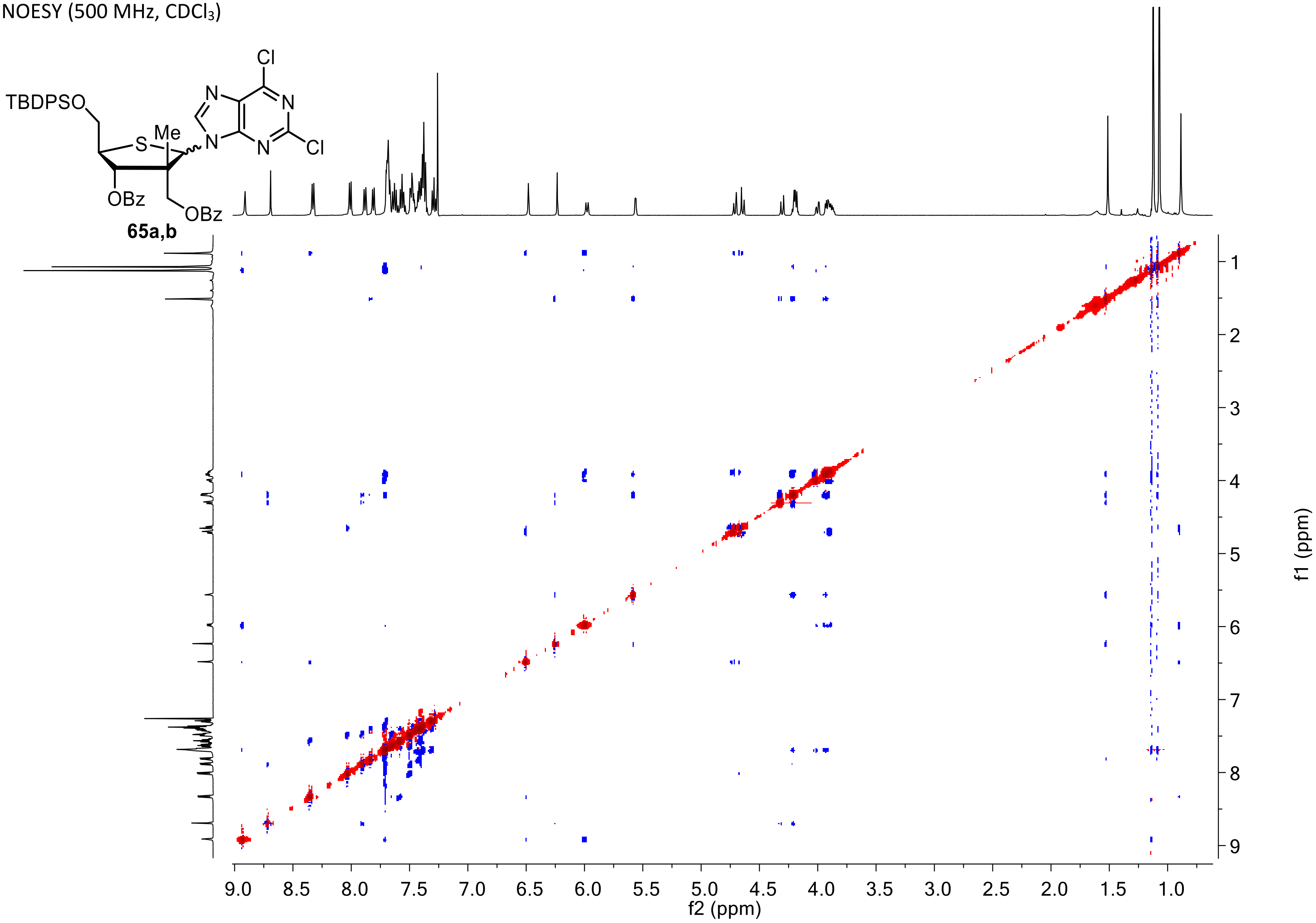

^{1}H-NMR (500 MHz, CD_3OD)

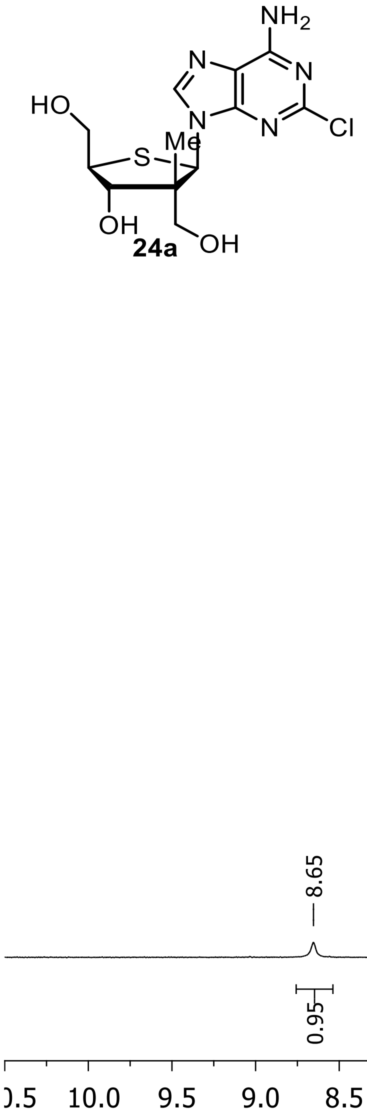

24a

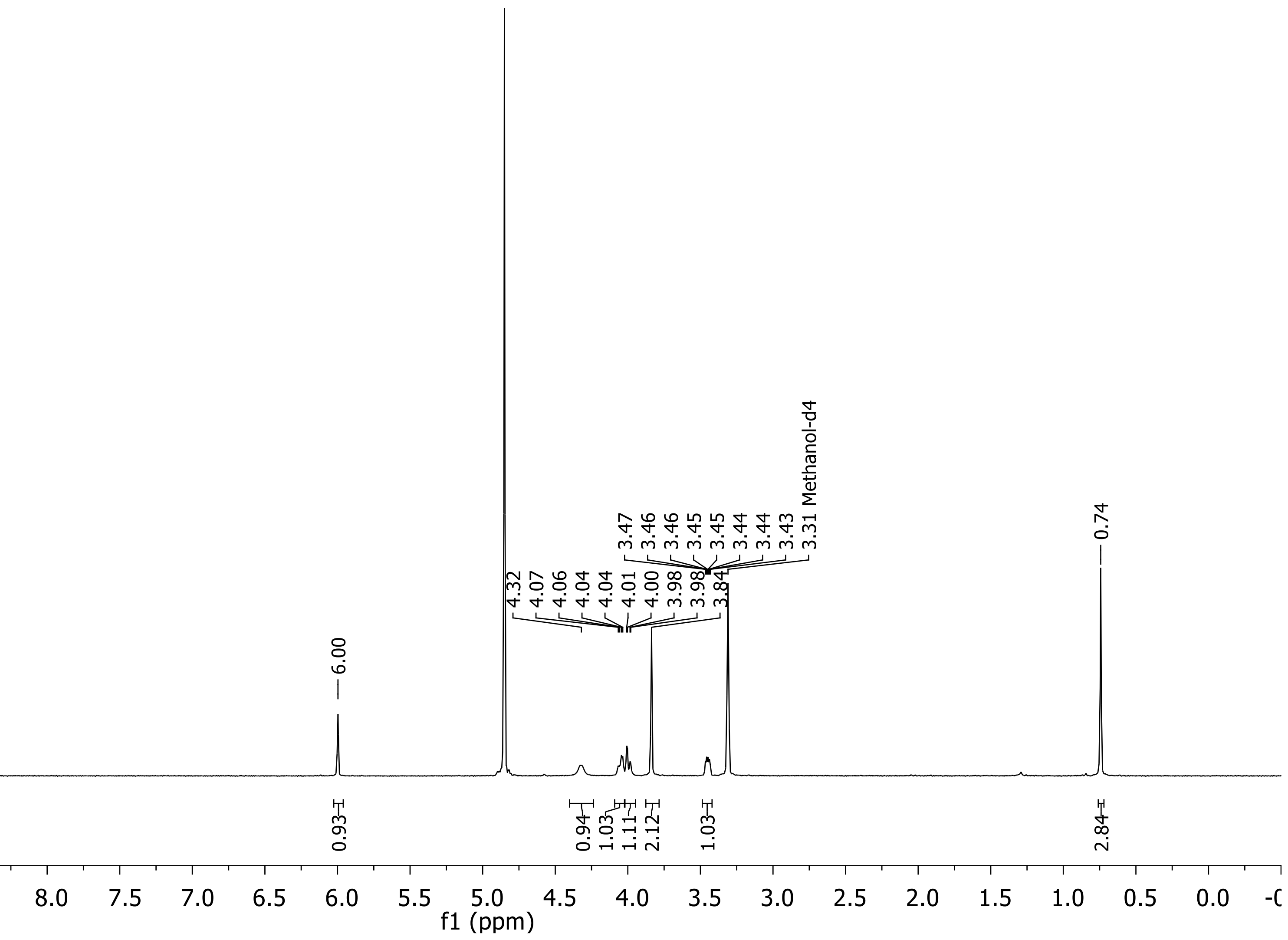

$^{13}C\{^{1}H\}$-NMR (126 MHz, CD_3OD)

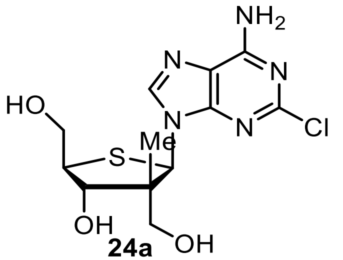

24a

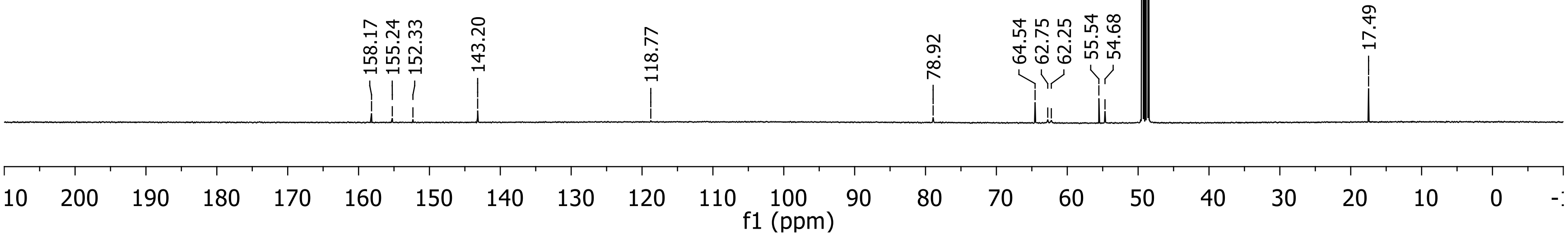

HMBC (500 MHz, CD_3OD)

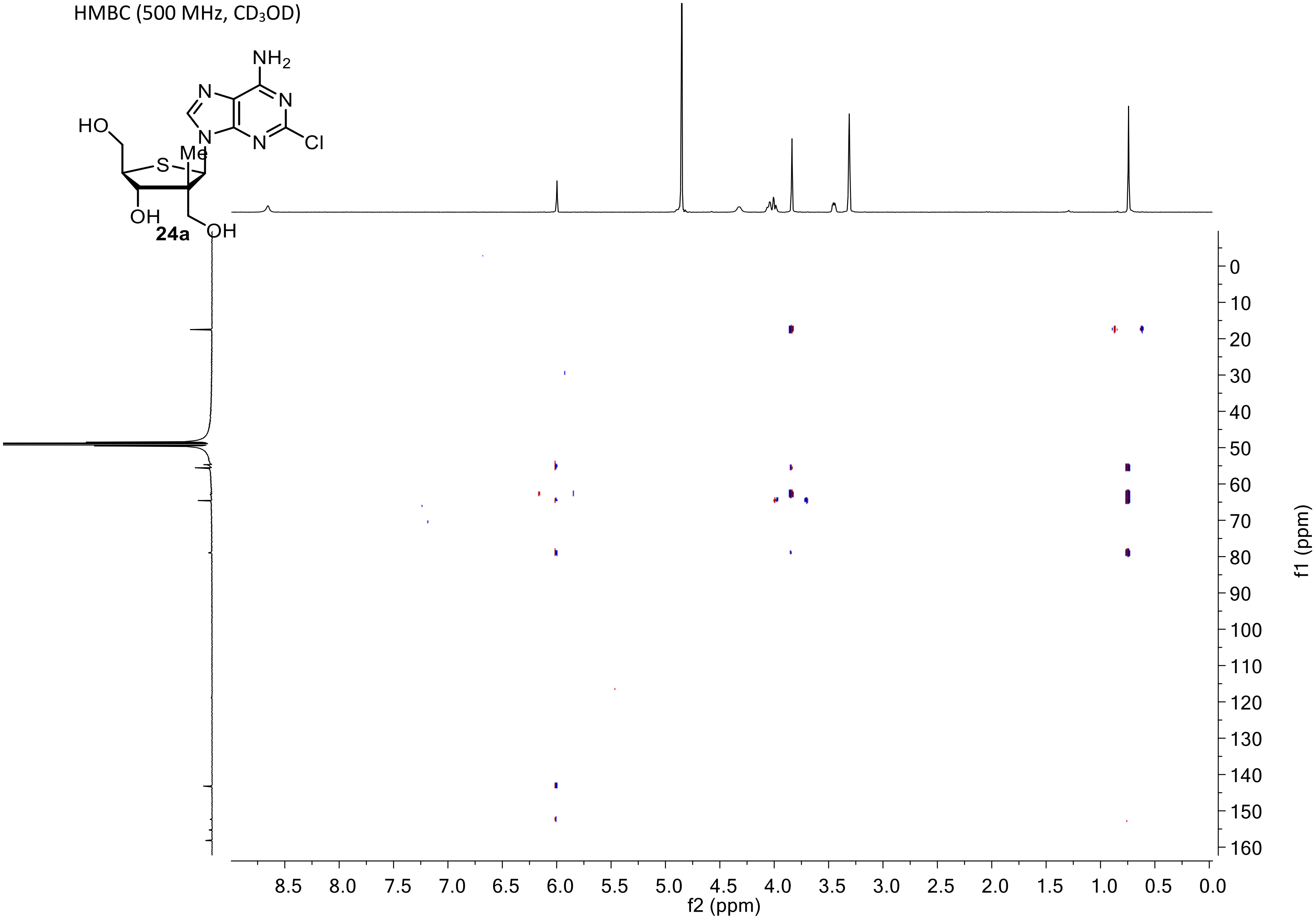

24a

NOESY (500 MHz, CD_3OD)

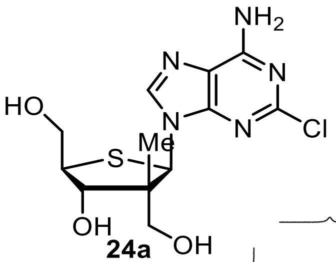

24a

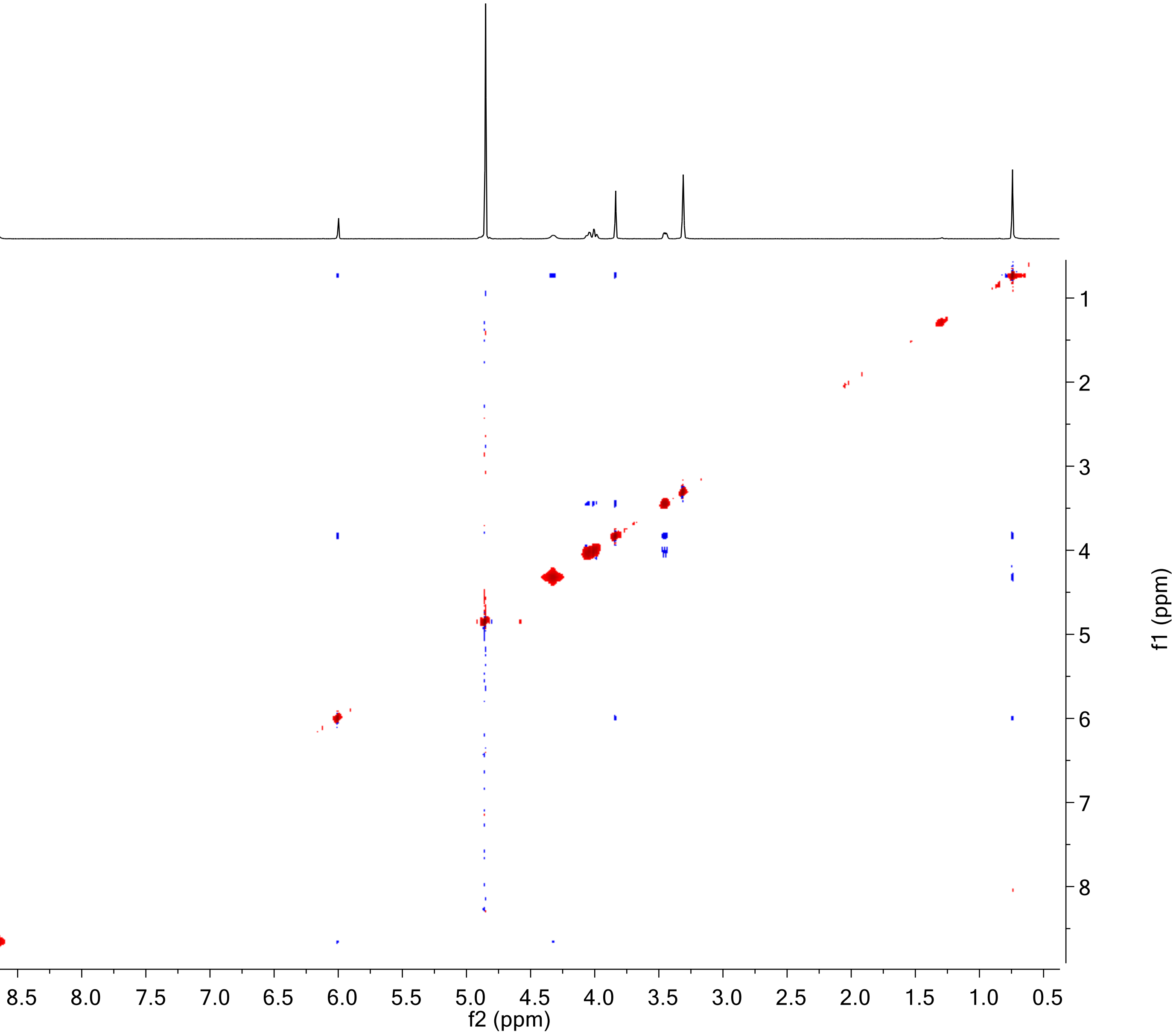

^{1}H-NMR (500 MHz, CD_3OD)

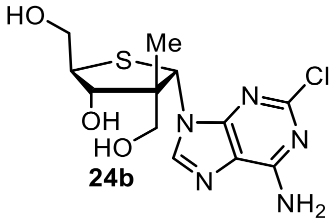

24b

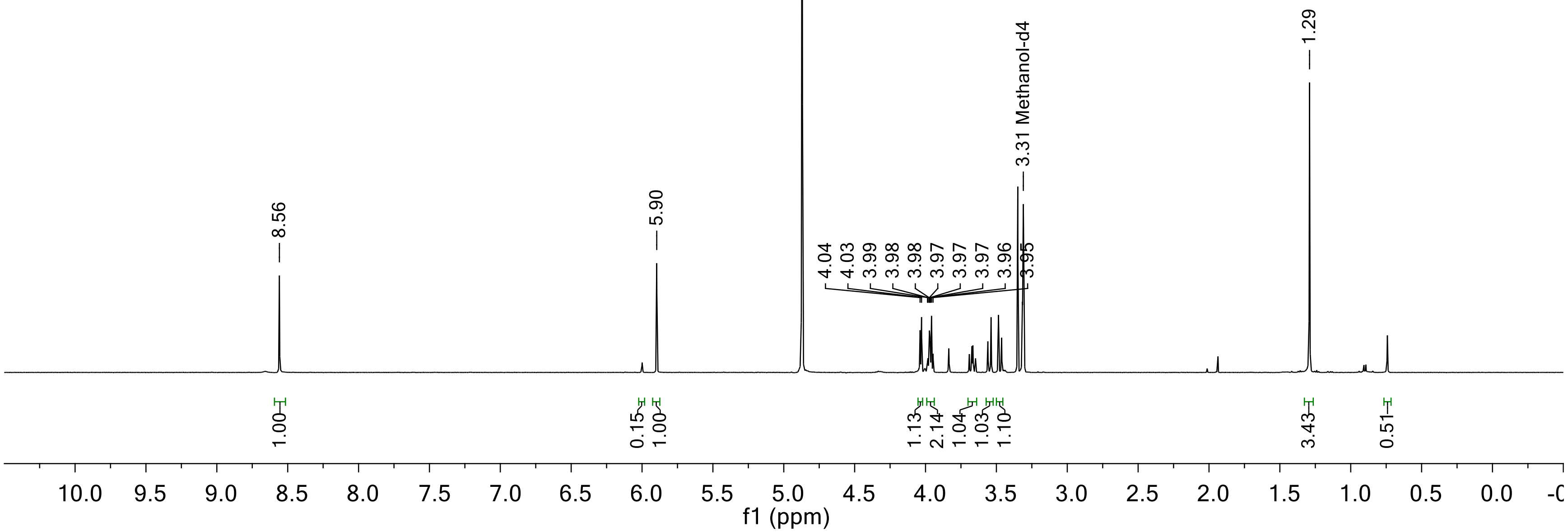

$^{13}C\{^1H\}$-NMR (126 MHz, CD_3OD)

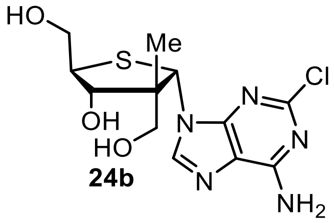

24b

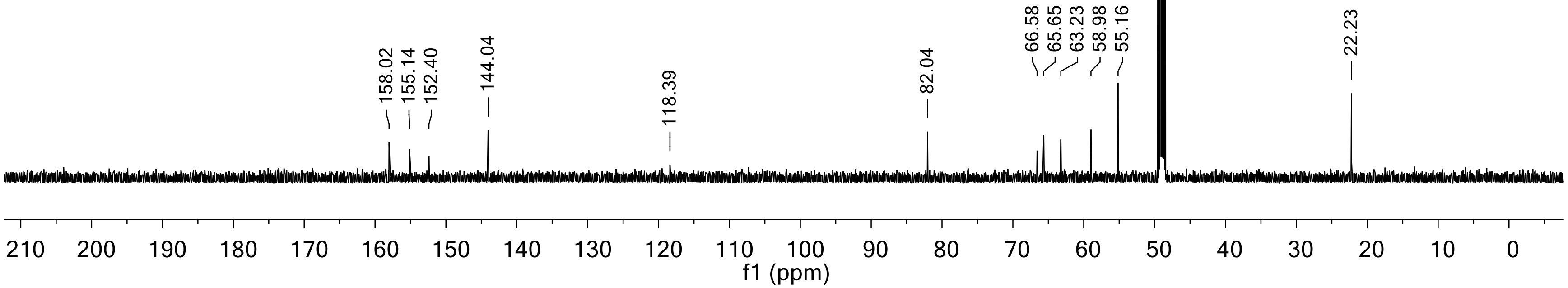

HSQC (500 MHz, CD_3OD)

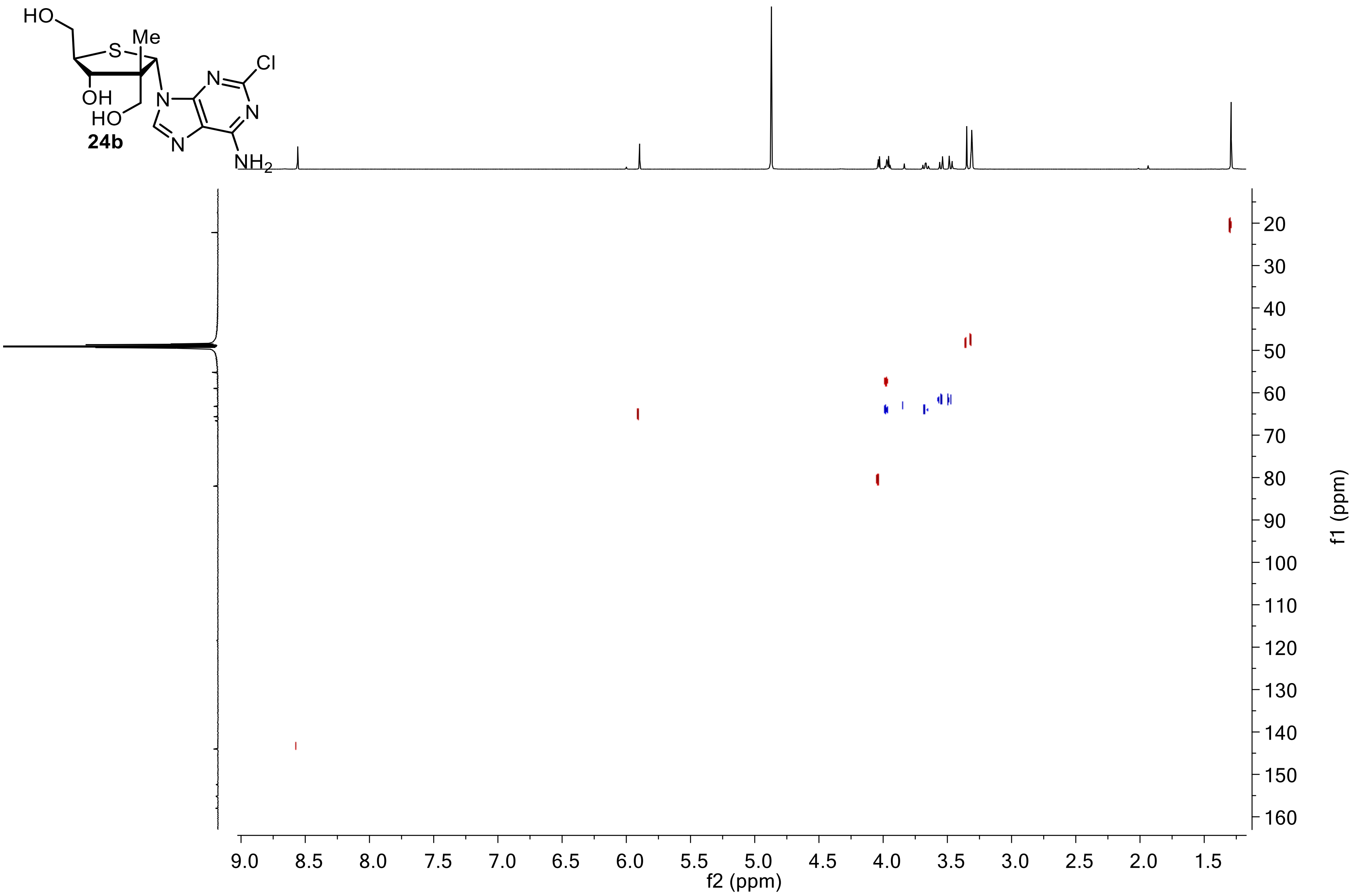

NOESY (500 MHz, CD_3OD)

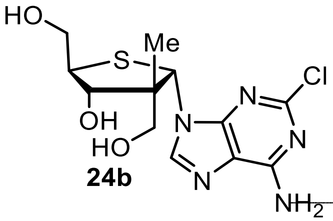

24b

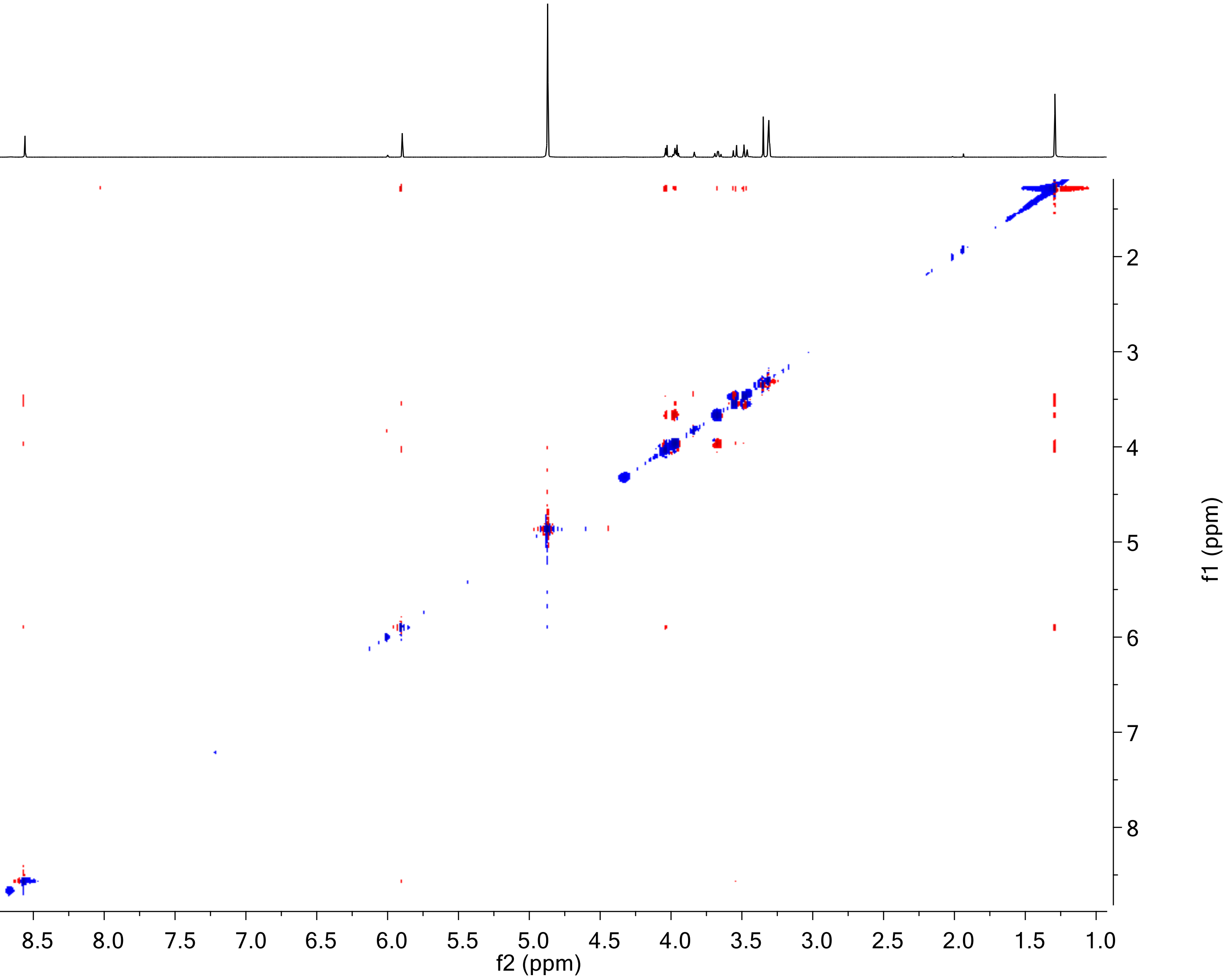

Supplement: Supplementary file 1 [file molecules-29-01647-s001.zip › molecules-2952096-supplementary.pdf]
